# Spurious Oscillations Caused by Density Functional Approximations: Who is to Blame? Exchange or Correlation?

**DOI:** 10.1021/acs.jctc.3c01339

**Published:** 2024-04-03

**Authors:** Sebastian P. Sitkiewicz, Rubén R. Ferradás, Eloy Ramos-Cordoba, Robert Zaleśny, Eduard Matito, Josep M. Luis

**Affiliations:** † Donostia International Physics Center (DIPC), Donostia 20018, Euskadi, Spain; ‡ Wrocław Centre for Networking and Supercomputing, Wrocław University of Science and Technology, Wyb. Wyspiańskiego 27, Wrocław PL-50370, Poland; § Polimero eta Material Aurreratuak: Fisika, Kimika eta Teknologia, Kimika Fakultatea, Euskal Herriko Unibertsitatea UPV/EHU, P.K. 1072, Donostia 20080, Euskadi, Spain; ∥ Ikerbasque Foundation for Science, Plaza Euskadi 5, Bilbao 48009, Euskadi, Spain; ⊥ Institute for Advanced Chemistry of Catalonia (IQAC), CSIC, Jordi Girona 18-26, Barcelona 08034, Spain; # Faculty of Chemistry, Wrocław University of Science and Technology, Wyb. Wyspiańskiego 27, Wrocław PL-50370, Poland; ¶ Institut de Química Computacional i Catàlisi (IQCC) and Departament de Química, Universitat de Girona, Girona 17003, Catalonia, Spain

## Abstract

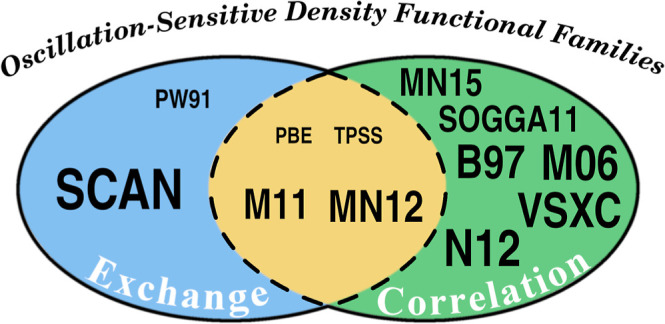

We analyze the varying susceptibilities of different density functional approximations (DFAs) to present spurious oscillations on the profiles of several vibrational properties. Among other problems, these spurious oscillations cause significant errors in harmonic and anharmonic IR and Raman frequencies and intensities. This work hinges on a judicious strategy to dissect the exchange and correlation components of DFAs and pinpoint the origins of these oscillations. We identify spurious oscillations in derivatives of all energy components with respect to nuclear displacements, including those energy terms that do not involve numerical integrations. These *indirect* spurious oscillations are attributed to suboptimal electron densities resulting from a self-consistent field procedure using a DFA that exhibits *direct* spurious oscillations. *Direct* oscillations stem from inaccurate numerical integration of the exchange and correlation energy density functionals. A thorough analysis of *direct* spurious oscillations reveals that only a handful of exchange and correlation components are insensitive to spurious oscillations, giving rise to three families of functionals, BH&H, LSDA, and BLYP. Among the functionals in these families, we encounter four widespread DFAs: BLYP, B3LYP, LC-BLYP, and CAM-B3LYP. Certain DFAs like PBE appear less sensitive to spurious oscillations due to compensatory cancellations between their energy components. Additionally, we found non-negligible but small oscillations in PBE and TPSS, which could be safely employed provided a sufficiently large integration grid is used in the calculations. These findings hint at the key components of current approximations to be improved and emphasize the necessity to develop accurate DFAs suitable for studying molecular spectroscopies.

## 1 Introduction

The applications of density functional theory (DFT) are widespread, including solid-state physics, materials chemistry, and spectroscopy, to name a few active areas of research.^1−3^ The success of DFT in every area is strongly dependent on the inherent accuracy of a given density functional approximation (DFA) in the description of the physical/chemical phenomenon under study.^4−9^ As far as chemistry is concerned, the development of DFAs resulted in quite successful predictions of structural and thermochemical properties of molecules, and nowadays, DFT is the most popular choice for characterizing potential energy hypersurfaces of electronic ground states and modeling the course of reactions. In contrast, the performance of DFAs in predicting excited-state properties is still far from expectations, as highlighted in recent reviews on this subject.^7,8^

A recent work of the present authors demonstrated that even electronic-ground-state vibrational spectroscopies might be challenging to model using DFAs.^10^ This is due to the presence of spurious oscillations in property profiles along nuclear coordinates, which might lead to substantial errors in properties involving molecular vibrations.^11^ These include vibrational spectroscopies like IR or Raman, and the vibrational contributions to molecular properties, for example, nonlinear optical properties. We showed that DFAs from all Jacob’s ladder rungs suffer from these spurious oscillations and the corresponding errors may reach hundreds of percent for the high-order derivatives required to compute the anharmonic corrections to the vibrational IR and Raman spectra.^11^ DFAs from the B97, M06, and SCAN families (see Table S2 for DFA families) were the most impacted by spurious oscillations, while BLYP and PBE were the least affected. In particular, large errors in the high-order derivatives of the energy, dipole moment, and polarizability with respect to the nuclear coordinates were reported, rendering many DFAs unsuitable for the computation of anharmonic corrections to vibrational spectra and molecular properties.^11^ This limitation is associated with the numerical integration that yields the exchange–correlation energy,^11^ which has been also studied in the recent works of Lehtola and Marques.^12−15^ Consequently, it is logical to anticipate that this issue might not be confined solely to ground-state vibrational spectroscopy. Indeed, our recent findings confirm its impact on excited states properties as well.^16^

Certain DFAs exhibit enhanced resistance to spurious oscillations compared to others.^10,11,16^ This suggests that when designing new DFAs, emphasis could be placed on formulations less prone to these oscillations. To support this initiative, the primary aim of this study is to analyze the exchange and correlation components within DFAs to understand the origins of this erratic behavior in specific DFAs. To this end, the first part of this work is devoted to the refinement and streamlining of the algorithm for detecting spurious oscillations, facilitating a comprehensive evaluation of a large collection of functionals. Namely, it deals with the study of the basis-set dependence and the dependence of spurious oscillations on the accuracy of molecular orbitals. These insights will be important in refining the process of quantifying such oscillations and analyzing how they affect different energy components. Finally, an analysis of both exchange and correlation functionals will permit identification of the part(s) of the DFA that cause spurious oscillations. In doing so, we will gain knowledge that would guide developers in designing more robust DFAs.

## 2 Methodology

In Kohn–Sham DFT (KS-DFT), the electronic energy is expressed as
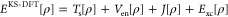
1where *T*_s_ is the Kohn–Sham kinetic energy, *V*_en_ is the electron-nuclei attractive potential, *J* is the Coulomb interaction energy, and *E*_xc_ is the exchange–correlation energy, which defines the DFA and it is typically divided into exchange (*E*_x_) and correlation (*E*_c_) energies. In practice, *E*_xc_ is the only part of the energy expression that is susceptible to numerical integration errors. *E*_xc_, as well as other elements involving the exchange–correlation kernel, such as elements of the Fock matrix or terms occurring in the response equations, are evaluated using a numerical quadrature. The numerical integration usually adopts Becke’s multicenter integration scheme, which represents the integral as a sum of atom-centered components.^17^ Becke’s atom-centered integrals are computed with quadratures in spherical polar coordinates and are split into angular and radial parts. The former one is most commonly represented with the Lebedev^18−20^ angular grid, whereas the sampling of the radial space is more challenging. In QCHEM 5.1,^21^ the Handy or Murray radial integration grids^22^ (adapting the Euler–Maclaurin quadrature) combined with Becke’s weighting scheme is normally used as default. We note that the use of other integration grids^23−26^ might reduce or increase the appearance of spurious oscillations. For instance, we find that Ochsenfeld’s extension^23^ of Becke’s nuclear weighting scheme reduces the spurious oscillations, although they are eliminated in none of the cases studied. Unfortunately, this integration scheme is not available in most computational packages, Psi4 being an exception.^27−29^ For the effect of this and other radial integration grids and weighting schemes, we refer to Section S4 of the Supporting Information. The grids are commonly represented as (*N*_*r*_, *N*_Ω_), where *N*_*r*_ and *N*_Ω_ are the number of radial and angular points of each atom-centered grid, respectively. In our previous study,^11^ we have found that the magnitude of spurious oscillations varies with grid size, typically diminishing significantly only when using exceptionally large grids. Being the cost of calculation proportional to the grid size is in the interest of widespread applicability making the grid size as small as possible (the proportionality is slightly reduced in the presence of exact exchange). For illustration, a substantial reduction in spurious oscillations required at least the use of an unpruned grid (750, 974). For nonhybrid DFAs, this grid demands over 12 times the computational time compared to the largest default grid in many computational software packages.^11,16^

In this study, we assess the sensitivity of DFA energy components to spurious oscillations, following the method outlined in our recent publication.^11^ Specifically, for a designated DFA, we calculate the energy (*E*), the dipole moment (μ_*z*_), the polarizability (α_*zz*_), and their derivatives with respect to nuclear displacements (labeled as ξ); this set of attributes is referred to as *P*^DFA^, which includes the former values computed at various values of the nuclear coordinate, forming a *property profile*. The calculation of a profile rather than a single value at equilibrium (or any other relevant geometry) is necessary, considering the oscillatory shape of the property along the nuclear coordinates. We also investigate the spurious oscillations in the derivatives of the energy components present in eq 1, namely d^*m*^*T*_s_/dξ^*m*^, d^*m*^*V*_en_/dξ^*m*^, d^*m*^*J*/dξ^*m*^, d^*m*^*E*_x_/dξ^*m*^, and d^*m*^*E*_c_/dξ^*m*^, for orders of derivatives up to *m* = 4.

In order to quantify the spurious oscillations in *P*^DFA^, we designed an algorithm in ref (11). The first step of the algorithm is finding a proper reference to compare with that of *P*^DFA^. Typically, we repeat the calculation with a large grid, *P*^DFA(LG)^ to have a profile the closest to an oscillation-free version of the target profile, *P*^DFA^. However, there is no guarantee that this profile is entirely free of spurious oscillations, so we require a filtering process. The algorithm transforms *P*^DFA(LG)^ to the reciprocal (frequency) space by using a discrete Fourier transform. In the frequency spectrum, spurious oscillations of *P*^DFA(LG)^ correspond to high-frequency bands that are identified by comparison with the profile of some ab initio method that does not involve numerical integrations (e.g., HF or post-HF methods). The bands causing the spurious oscillations are filtered out using an automatically designed low-pass finite impulse response filter, generating a *clean* profile, *P*_filt_^DFA(LG)^. We quantify spurious oscillations through the root-mean-square error (RMSE)
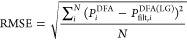
2where the summation runs over the set of *N* points in the property profile along a given nuclear displacement coordinate.

## 3 Computational Details

We test the stability of 45 DFAs, which represent different families and rungs of the DFT Jacob’s ladder (see the list in Tables S1 and S2), on these complexes: HCN·HF, HCN·HCl, N_2_·HF, OC·HF, HCN·BrF, and Ar_2_. All of them are noncovalently bonded systems, which previously proved to be prone to show spurious oscillations on property profiles along nuclear displacement coordinates.^11^ For each studied DFA, the geometries were optimized using the aug-cc-pVTZ basis set and the (250, 974) grid. Examining all possible atomic displacements entails a substantial computational cost, which can be significantly reduced by considering only the most relevant displacement. The selection of the nuclear displacement coordinate is a key step of the algorithm, for which we employ the first-order field induced coordinate (FIC) along the main internuclear axis *z*, χ_1,*z*_.^30−32^ This effectively represents the overall response of the equilibrium geometry to an external electric field (see eq S1 for further details). For the noncovalently bound complexes studied, χ_1,*z*_ is mainly composed of the low-frequency intermolecular stretching mode. Subsequently, property profiles were built from the optimized geometry by implementing a series of displacements along the χ_1,*z*_ coordinate. For SVWN5 and SPW92, the optimization procedure is omitted, and the B3LYP geometries are adopted instead.

The calculation of RMSE involves geometries within a displacement range that we have selected following these criteria: (i) for hydrogen- and halogen-bonded systems, 33 points in the range 0 ± 0.32χ_1,*z*_ (i.e., displacements along the normalized FIC coordinate, starting from the optimized geometry), (ii) 129 points in the range 7.2 ± 0.32 au for Ar_2_. The range is marked with dashed vertical lines for HCN·HF in the following Figures and Figures S9–S17.

In this study, integration grids (99, 590), (250, 974), (750, 974), and (1500, 974) were examined, with the latter being utilized as the large grid (LG) to procure the filtered property profiles. For the integration of the nonlocal VV10 correlation in ωB97M-V, ωB97X-V, and B97M-V, a pruned SG3 grid is employed.^33^ Except for the optimization procedures and tests of basis-set dependence in this study, the 6-31+G* basis set is applied for all electronic structure calculations. In the KS-DFT SCF calculations, the DIIS convergence criterion is set to 10^–11^ and nearly all of the two-electron integrals are included (cutoff set to 10^–30^). Static polarizabilities are obtained analytically from the CPKS equation. All HF and KS-DFT calculations were performed with QCHEM 5.1^21^ and all graphs were generated with the Matplotlib package.^34^

## 4 Results and Discussion

### 4.1 Basis Set Dependence

**Figure 1:**
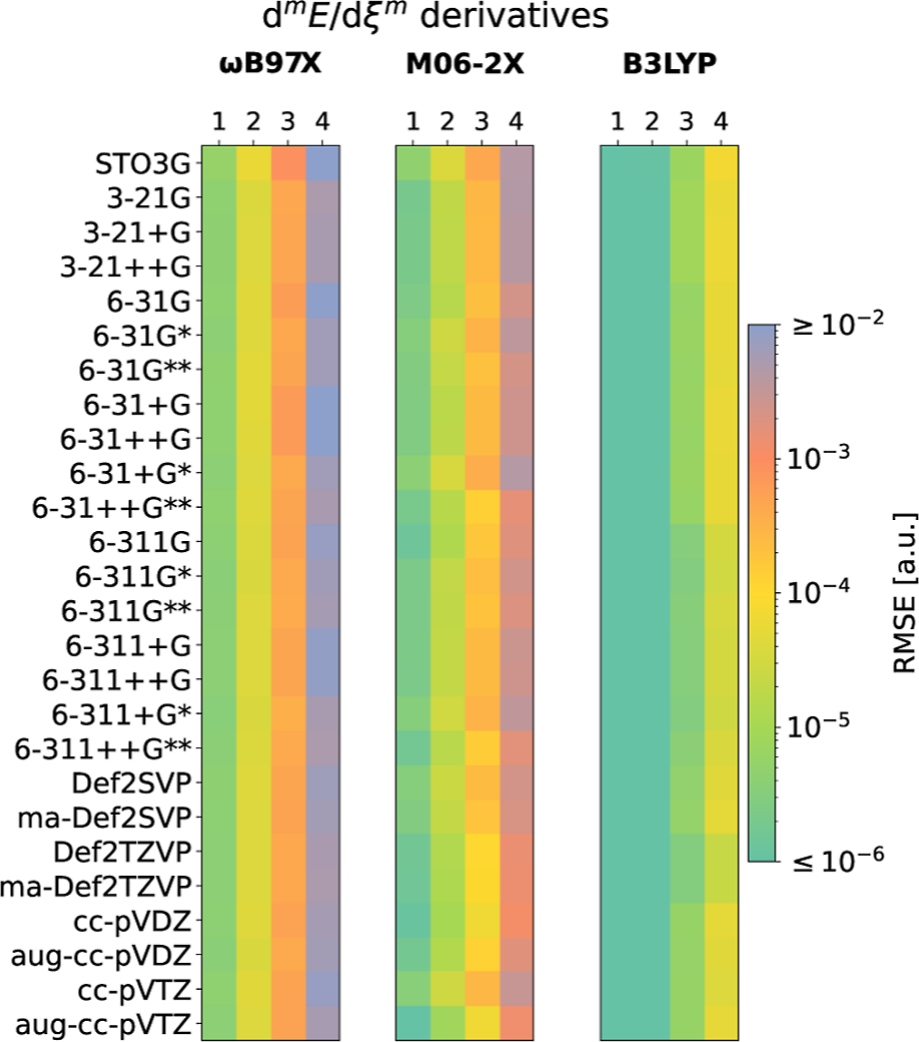
RMSE values of d^*m*^*E*/dχ_1,*z*_^*m*^ derivatives (*m* = 1–4) of HCN·HF using ωB97X, M06-2X, and B3LYP combined with the (99, 590) integration grid for a large collection of basis sets. Colors reflect the magnitude of the RMSE. Raw data is available in Tables S3–S8.

First of all, we will analyze the extent to which the spurious oscillations depend on the quality of the basis set employed. We tested 26 basis sets of different sizes and families (see Figure 1). For three representative DFAs, ωB97X,^35^ M06-2X,^36^ and B3LYP,^37,38^ we calculated the RMSE of the derivatives of the energy, dipole moment, and polarizability of the HCN·HF system. The results of d^*m*^*E*/dξ^*m*^ for the (99, 590) grid are depicted in Figure 1 (for other properties and grids see Figures S2 and S3). The errors coming from spurious oscillations in the derivatives exhibit minimal dependency on the basis set across all of the examined properties. In nearly all instances, the extent of the spurious oscillation error discerned with aug-cc-pVTZ can be anticipated using a substantially smaller basis set such as Def2-SVP, 6-31+G*, or even 6-31G. This holds true across all properties and DFAs, including high-order derivatives like d^4^*E*/dξ^4^. Consequently, the actual computational expense of pinpointing spurious oscillations can be significantly mitigated by utilizing a small basis set. Therefore, in the rest of this paper, we will employ the 6-31+G* basis set. Notice that the use of small basis sets is solely to quantify spurious oscillations, and we recommend bigger basis sets for studies of spectroscopic properties.

### 4.2 Direct and Indirect Errors

During the self-consistent field procedure to converge the Kohn–Sham equations, the errors committed by an inaccurate numerical integration of the exchange–correlation energy density (hereafter *direct errors*) can extend to the molecular orbitals and, consequently, to the electron density. As a result, energy components that do not require numerical integration can be affected by spurious oscillations, giving rise to *indirect errors*. In this section, we analyze how important these *direct* and *indirect errors* are in the presence of spurious oscillations. In order to analyze the part of the exchange–correlation functional that suffers from direct errors, we define  and , which exclude exact exchange.

In this section, the derivative profiles of every component of the KS-DFT electronic energy (d^*m*^*T*_s_/dξ^*m*^, d^*m*^*V*_en_/dξ^*m*^, d^*m*^*J*/dξ^*m*^, , , , and ) are computed using B3LYP, VSXC, B97, ωB97M-V, M06-2X, and MN15. These calculations are performed on two integration grids, (99, 590) and (1500, 974), with the latter being considered in this case an oscillation-free benchmark that does not require filtering.

**Figure 2:**
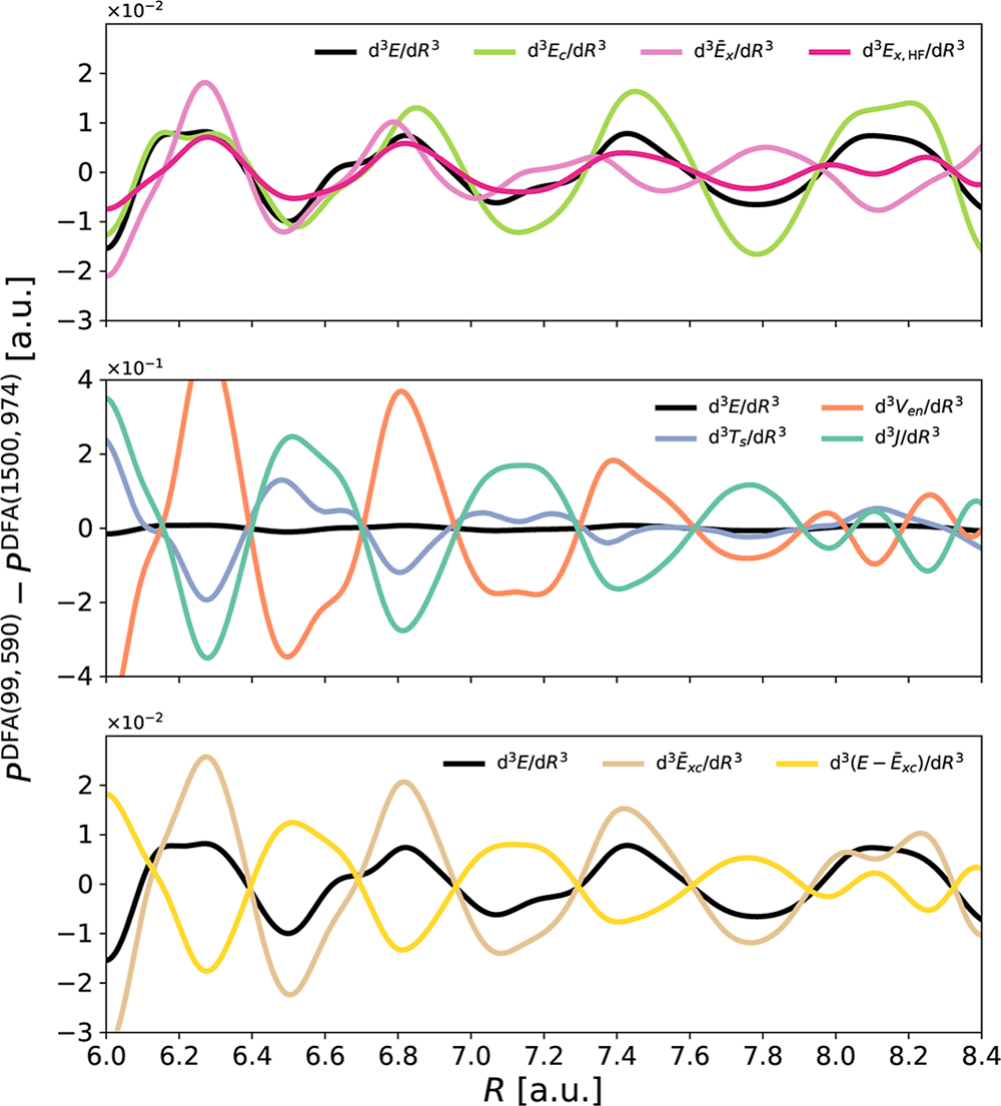
Spurious oscillations in the third derivatives of energy components of Ar_2_: total electronic (*E*), kinetic (*T*_s_), nuclei attraction (*V*_en_), Coulomb repulsion (*J*), exact HF-like exchange (*E*_x,HF_), pure exchange , and correlation (*E*_c_), as well as their sum . Obtained with MN15/6-31+G* as the difference between (99, 590) and (1500, 974) integration grids. See Figures S4–S8 for other DFAs.

The focus of our analysis is on the third derivatives of the electronic energy obtained with the MN15 functional (Figure 2), albeit analogous conclusions are expected for other properties and DFAs (Figures S4–S8). Spurious oscillations afflict every energy component, evidencing that numerical errors propagate among energy components during the self-consistent field procedure. The oscillation magnitude in the derivatives of *T*_s_, *V*_en_, and *J* is an order of magnitude larger than in the derivatives of *E*_x,HF_, , and *E*_c_. The oscillations in d^3^*J*/dξ^3^ are out-of-phase compared to those in d^3^*V*_en_/dξ^3^, giving a partial compensation for errors in the total energy. Some DFAs also exhibit cancellations between d^3^*T*_s_/dξ^3^ and d^3^*V*_en_/dξ^3^ (d^3^*E*_c_/dξ^3^); see Figures S4–S8. Interestingly, the oscillation magnitude of the aggregated terms subjected to the *indirect* grid error, that is, d^3^(*T*_s_ + *V*_en_ + *J* + *E*_x,HF_)/dξ^3^ = , aligns with those generated by , which is the source of the *direct* grid error.

These findings indicate that the total RMSE values represent a minimal estimate of the actual errors incurred in calculating the exchange–correlation energy term due to numerical integration errors. Hence, a thorough evaluation of DFAs should be based on separate scrutiny of the exchange and correlation energy terms to ensure a comprehensive understanding and accurate assessment of spurious oscillations and their implications. Such detailed scrutiny is vital for DFA developers who aim to create more robust and accurate approximations.

### 4.3 Role of the Electron Density

In this section, we analyze the effect of using different types of densities on the oscillations of the property profiles of d^*m*^*E*/dξ^*m*^. The purpose of this study is to further reduce the computational cost involved in calculating *P*^DFA^ and *P*_filt_^DFA(LG)^. The density obtained through the self-consistent field process inherent to solving the Kohn–Sham equations—and which has been employed thus far for assessing spurious oscillations—will be referred to as SCF density. We will be examining three additional input densities: (i) the densities obtained from the superposition of atomic densities (SAD),^39^ (ii) its *purified* version, SADMO,^12,21^ obtained from an initial diagonalization of the SAD density matrix, followed by the Aufbau occupation of the corresponding natural orbitals employed to construct the density, and (iii) the SCF density for the equilibrium geometry applied across the entire property profile (frozen molecular orbitals or FMO). FMO calculations employ the same molecular orbital coefficients of the equilibrium geometry but are reorthonormalized to adapt to the new geometry. All SCF densities will be computed using a (1500, 974) grid, but not necessarily the properties derived from them, to circumvent *indirect errors*, ensuring a fair comparison of all densities. This is crucial as other densities that are not procured through a self-consistent field process are devoid of *indirect errors* (see the previous subsection).

**Figure 3:**
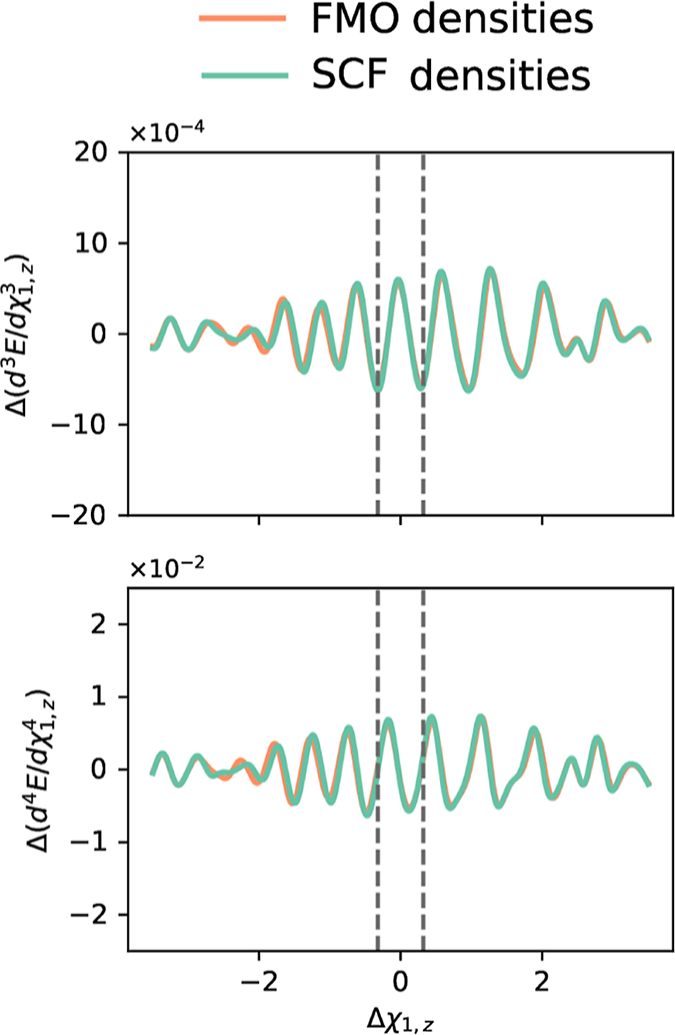
Spurious oscillations in d^3^*E*/dχ_1,*z*_^3^ and d^4^*E*/dχ_1,*z*_^4^ of HCN·HF using the FMO density (orange curves) and the SCF density (green curves) at the B97/6-31+G* level, where the oscillations are defined as Δ*P* = *P*^DFA(99, 590)^ – *P*^DFA(1500, 974)^. Vertical dashed lines mark the displacement range of the property curve for which RMSE, and RRMSE are calculated (see Computational Details section).

We tested HCN·HF and Ar_2_, a collection of 20 DFAs (see Table S25) employing the four aforementioned densities and the three integration grids. For the sake of brevity, we will only compare SCF and FMO densities (see Figures S9–S17 for SAD and SADMO densities assessment). Figure 3 shows that the spurious oscillations of  and d^4^*E*_c_/dξ^4^ for HCN·HF obtained with B97 and the (99, 590) grid using the SCF and FMO densities are barely distinguishable. Figures S9–S17 corroborate these conclusions across various DFAs. The FMO and SCF densities align closely, displaying excellent agreement, while the accord between SAD and SADMO densities is lesser in comparison.

**Figure 4:**
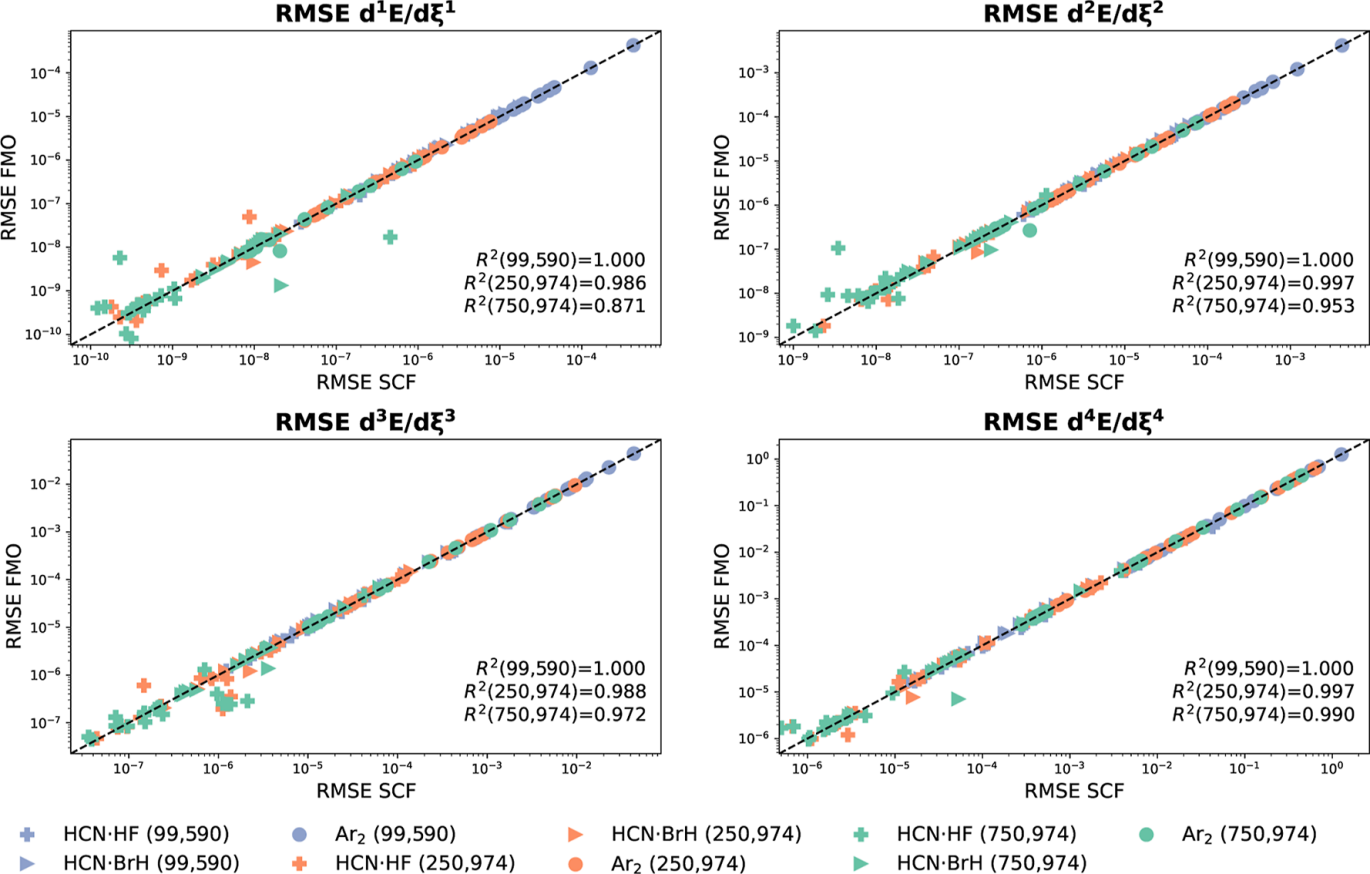
Correlation between RMSE in d^*m*^*E*/dξ^*m*^ obtained using the SCF and FMO densities for 20 DFAs (see Table S25) combined with the 6-31+G* basis set. Two complexes (HCN·HF and Ar_2_) and three numerical grids [(99, 590), (250, 974), and (750, 974)] are tested.

In Figure 4, we present the correlation plots of the RMSE values of HCN·HF and Ar_2_ profiles of d^*m*^*E*/dχ_1,*z*_^*m*^ for various grids obtained using FMO and SCF densities (see Figures S18 and S19 for SAD and SADMO densities, respectively). These graphs confirm the excellent agreement between the RMSE obtained from FMO and SCF densities. Given that profiles created from FMO densities necessitate only the SCF molecular orbital coefficients of the molecule at equilibrium, the computational cost for the entire profile is reduced by a factor of *N*, where *N* represents the number of points in the profile.^a^ FMO densities are thus an ideal choice for examining spurious oscillations in  and *E*_c_, as they explicitly eliminate *indirect errors* and significantly reduce computational costs.

### 4.4 Assessment of Exchange and Correlation Functionals

In this section, we assess the extent of the *direct errors* in d^*m*^*E*/dξ^*m*^ by separately analyzing  and d^*m*^*E*_c_/dξ^*m*^. Our objective is to determine whether it is the exchange, the correlation, or both components that contribute to spurious oscillations. For this purpose, we will utilize FMO densities and a modest basis set, 6-31+G*, following the conclusions drawn in the previous sections. The results obtained in this section will assist DFA developers in identifying which components of the exchange–correlation DFA need to be refined to prevent spurious oscillations. Throughout this process, we will also pinpoint exchange and correlation functionals that are not sensitive to spurious oscillations, making them reliable choices for computing ground- and excited-state vibrational spectroscopies.

**Figure 5:**
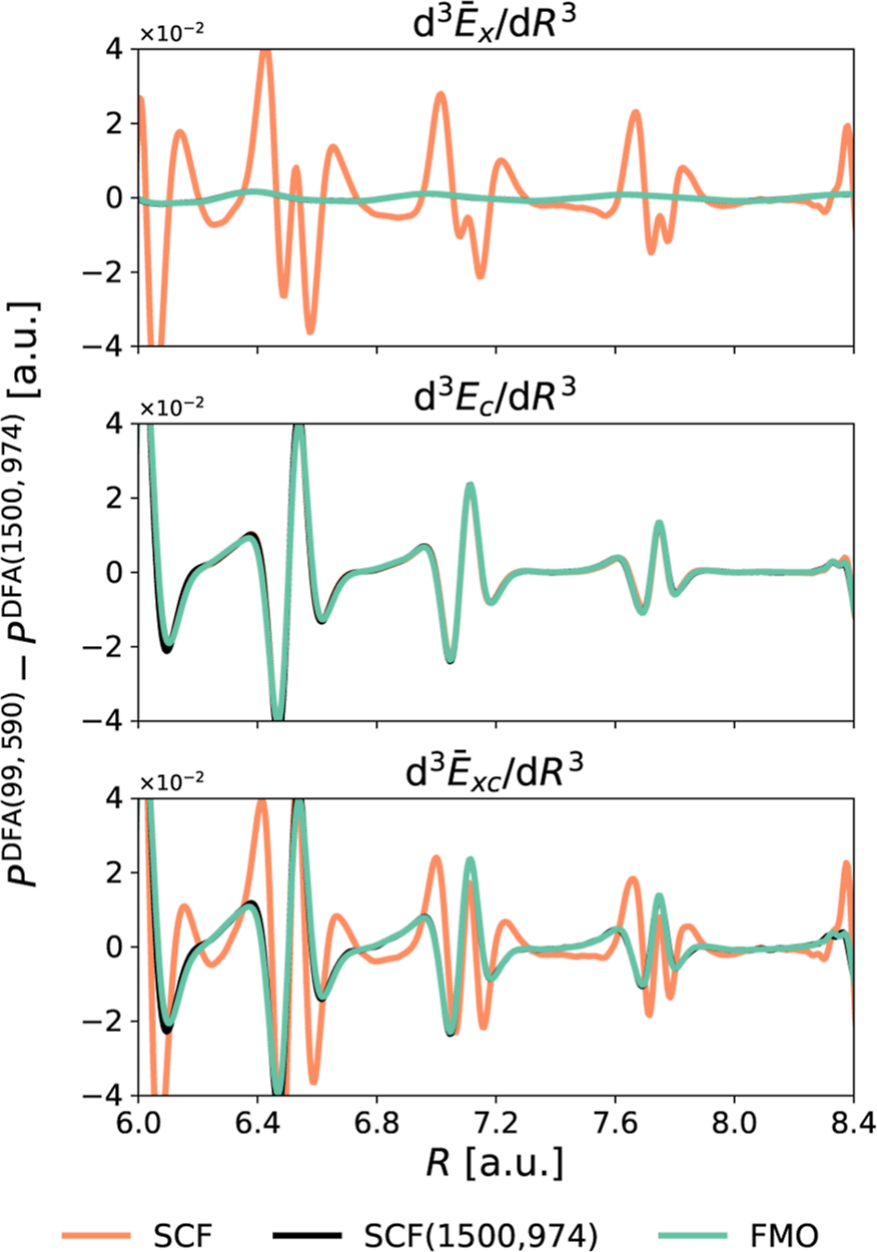
Spurious oscillations in the third derivatives of pure exchange  and correlation (*E*_c_) energy components of Ar_2_, as well as their sum, obtained using B97/6-31+G* and the (99, 590) and (1500, 974) integration grids. Three different densities are employed: (i) SCF density (SCF), (ii) SCF(1500, 974) —obtained using the (1500, 974) grid, and (iii) FMO, constructed from the reorthonormalized molecular orbital coefficients obtained from the SCF density of one geometry (*R* = 7.2 au) using the (250, 974) integration grid.

Figure 5 illustrates the importance of using a density devoid of *indirect errors* to clearly identify which part of the functional is responsible for the spurious oscillations (see also Figures S20–S24). Here, we depict  and d^3^*E*_c_/d*R*^3^ for Ar_2_ using B97 and the (99, 590) grid. These calculations employ three distinct densities: the SCF density (which is contaminated with *indirect errors*), the SCF density paired with a large grid, and the FMO density. The profiles derived from the latter two densities are essentially equivalent, thereby validating the use of the FMO density. An inspection of  reveals that the spurious oscillations of this term are attributable to the *indirect errors* imbued in the SCF density. On the other hand, the genuine oscillations caused by the *direct errors* arising from inaccurate numerical integration of the exchange term are notably smaller. Hence, within B97, the spurious oscillations stem from numerical integration inaccuracies of the correlation functional.

To conduct a similar analysis for the 45 DFAs referenced in Table S1, we calculated the root-mean-square error (RMSE) of up to the fourth derivative of *E̅*_x_ and *E*_c_ using FMO densities. These calculations were performed on two integration grids, (99,590) and (250,974), and involved various chemical systems, including HCN·HF, HCN·HCl, OC·HF, N_2_·HF, HCN·BrF, and Ar_2_. The corresponding RMSE values of the exchange and correlation energies (/dξ^*m*^ and d^*m*^*E*_c_/dξ^*m*^) are presented in Tables S9–S20, whereas the maximum deviations for each functional are reported in Table 1.

**Table 1:** Maximum RMSE for Each Functional among All the Systems, Grids and Derivative Orders, Sorted from Lowest to Highest^a^^,^^b^

DFA	*E*_c_	DFA	*E̅*_x_	DFA	*E̅*_xc_^abs^
BH&H	6.92 × 10^–^^4^	SOGGA11-X	2.99 × 10^–^^3^	BH&H	5.29 × 10^–^^3^
LC-BLYP	6.93 × 10^–^^4^	BH&H	4.59 × 10^–^^3^	LC-BLYP	7.65 × 10^–^^3^
BLYP	6.96 × 10^–^^4^	ωB97X-V	5.80 × 10^–^^3^	BH&HLYP	7.73 × 10^–^^3^
B1LYP	6.96 × 10^–^^4^	PBE50	5.99 × 10^–^^3^	CAMB3LYP	8.59 × 10^–^^3^
CAMB3LYP	6.97 × 10^–^^4^	B97	6.36 × 10^–^^3^	SPW92	9.94 × 10^–^^3^
BH&HLYP	6.99 × 10^–^^4^	LC-BLYP	6.95 × 10^–^^3^	SVWN5	9.94 × 10^–^^3^
B3LYP	7.35 × 10^–^^4^	BH&HLYP	7.03 × 10^–^^3^	B1LYP	1.10 × 10^–^^2^
SPW92	7.67 × 10^–^^4^	ωB97X	7.49 × 10^–^^3^	B3LYP	1.12 × 10^–^^2^
SVWN5	7.72 × 10^–^^4^	ωB97X-D3	7.82 × 10^–^^3^	BLYP	1.40 × 10^–^^2^
RevTPSS	7.57 × 10^–^^3^	ωB97	7.84 × 10^–^^3^	RevTPSS	1.66 × 10^–^^2^
PW91	9.75 × 10^–^^3^	CAMB3LYP	7.90 × 10^–^^3^	PBE50	1.84 × 10^–^^2^
PBE	9.80 × 10^–^^3^	LC-wPBE	7.93 × 10^–^^3^	LC-wPBE	1.91 × 10^–^^2^
mPW91	1.02 × 10^–^^2^	PBE0	8.63 × 10^–^^3^	PBE0	1.98 × 10^–^^2^
LC-wPBE	1.11 × 10^–^^2^	RevTPSS	9.00 × 10^–^^3^	PBE	2.09 × 10^–^^2^
PBE0	1.12 × 10^–^^2^	ωB97X-D	9.03 × 10^–^^3^	B1PW91	2.23 × 10^–^^2^
B1PW91	1.16 × 10^–^^2^	SPW92	9.17 × 10^–^^3^	TPSSh	2.33 × 10^–^^2^
TPSS	1.17 × 10^–^^2^	SVWN5	9.17 × 10^–^^3^	TPSS	2.39 × 10^–^^2^
TPSSh	1.22 × 10^–^^2^	B1LYP	1.03 × 10^–^^2^	PW91	3.44 × 10^–^^2^
PBE50	1.24 × 10^–^^2^	B3LYP	1.05 × 10^–^^2^	mPW91	3.56 × 10^–^^2^
B97M-V	1.51 × 10^–^^2^	N12-SX	1.06 × 10^–^^2^	B97M-V	3.65 × 10^–^^2^
ωB97X-V	3.56 × 10^–^^2^	B1PW91	1.07 × 10^–^^2^	ωB97X-V	4.14 × 10^–^^2^
SOGGA11-X	4.06 × 10^–^^2^	PBE	1.11 × 10^–^^2^	SOGGA11-X	4.36 × 10^–^^2^
M11	4.62 × 10^–^^2^	TPSSh	1.12 × 10^–^^2^	ωB97M-V	1.11 × 10^–^^1^
MN15	8.16 × 10^–^^2^	ωB97M-V	1.19 × 10^–^^2^	MN15	1.21 × 10^–^^1^
ωB97M-V	9.94 × 10^–^^2^	TPSS	1.22 × 10^–^^2^	M11	1.27 × 10^–^^1^
SOGGA11	1.15 × 10^–^^1^	BLYP	1.33 × 10^–^^2^	MN15-L	1.66 × 10^–^^1^
MN12-SX	1.26 × 10^–^^1^	B97-D	2.12 × 10^–^^2^	SOGGA11	1.72 × 10^–^^1^
M11-L	1.36 × 10^–^^1^	B97M-V	2.13 × 10^–^^2^	N12-SX	1.98 × 10^–^^1^
MN15-L	1.40 × 10^–^^1^	VSXC	2.24 × 10^–^^2^	MN12-L	2.39 × 10^–^^1^
MN12-L	1.45 × 10^–^^1^	PW91	2.47 × 10^–^^2^	B97-D	2.63 × 10^–^^1^
M06-2X	1.86 × 10^–^^1^	mPW91	2.54 × 10^–^^2^	M06-2X	2.76 × 10^–^^1^
N12-SX	1.87 × 10^–^^1^	MN15-L	2.66 × 10^–^^2^	M11-L	3.15 × 10^–^^1^
B97-D	2.42 × 10^–^^1^	N12	3.94 × 10^–^^2^	MN12-SX	3.40 × 10^–^^1^
B97	3.44 × 10^–^^1^	MN15	3.94 × 10^–^^2^	B97	3.50 × 10^–^^1^
SCAN	3.59 × 10^–^^1^	SOGGA11	5.80 × 10^–^^2^	VSXC	4.75 × 10^–^^1^
SCAN0	3.63 × 10^–^^1^	M06	7.04 × 10^–^^2^	M06-L	5.81 × 10^–^^1^
VSXC	4.53 × 10^–^^1^	M11	8.07 × 10^–^^2^	ωB97	6.12 × 10^–^^1^
M06-HF	4.66 × 10^–^^1^	M06-2X	9.08 × 10^–^^2^	M06	6.93 × 10^–^^1^
M06-L	4.86 × 10^–^^1^	MN12-L	9.38 × 10^–^^2^	ωB97X	6.94 × 10^–^^1^
ωB97	6.04 × 10^–^^1^	M06-L	9.52 × 10^–^^2^	M06-HF	6.98 × 10^–^^1^
M06	6.22 × 10^–^^1^	M11-L	1.79 × 10^–^^1^	ωB97X-D3	8.31 × 10^–^^1^
ωB97X	6.86 × 10^–^^1^	MN12-SX	2.13 × 10^–^^1^	SCAN0	1.14 × 10^0^
ωB97X-D3	8.23 × 10^–^^1^	M06-HF	2.32 × 10^–^^1^	ωB97X-D	1.21 × 10^0^
ωB97X-D	1.20 × 10^0^	SCAN0	7.82 × 10^–^^1^	N12	1.29 × 10^0^
N12	1.25 × 10^0^	SCAN	1.04 × 10^0^	SCAN	1.40 × 10^0^

a In Tables S9 and S20, we collect individual data.

b The last column contains the cumulative absolute RMSE of pure exchange and correlation.

The data in Table 1 shows that DFAs consisting of certain exchange and correlation functionals are relatively insensitive to present spurious oscillations. For instance, B88, LSDA, TPSS, and PBE exchange functionals and LYP, LSDA, TPSS, PW91, and PBE correlation functionals present relatively small maximal RMSE values. In particular, LSDA exchange–correlation and LYP correlation functionals might be safely used to design DFAs resilient to spurious oscillations. When we consider the sum of absolute magnitudes of the exchange and correlation functionals that compose a given DFA, we find a short list of DFAs that are relatively insensitive to spurious oscillation errors and can be grouped into three DFA families (see Table S2 for DFA families). The list includes BH&H,^40^ the LSDA family (SVWN5^41,42^ and SPW92^41,43^), and the BLYP family (BLYP,^44,45^ B1LYP,^46^ B3LYP,^37,38^ BH&HLYP,^47^ LC-BLYP,^48^ and CAM-B3LYP^49^). Generally, for these DFAs, the RMSE value is larger for the exchange energy compared to the correlation energy. For instance, the B88 exchange functional^44^ presents larger errors than the LYP correlation functional in the BLYP functional and other members of the BLYP family.

**Figure 6:**
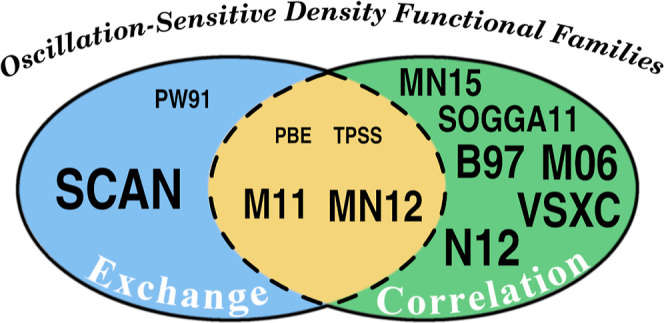
Sensitivity to spurious oscillations of DFA families (see Table S2), which are classified into those exhibiting a dominant sensitive component, exchange (blue) or correlation (green), and those in which both components contribute similarly to the spurious oscillations (yellow). The size of the font indicates the magnitude of the spurious oscillations. Based on the data from Table S26 (component dominance) and Table 1 (maximal RMSE magnitudes).

In Figure 6, we summarize the results of the DFA families that are more affected by the spurious oscillations (i.e., those showing a maximal RMSE value above 0.01, see Table S26), dividing them into three groups based on the dominant source of error, whether it be exchange, correlation, or a combination of both. Among the functionals that present a dominant RMSE in the exchange functional, we find the PW91 (PW91,^50^ mPW91,^51^ and B1PW91^46^) and SCAN (SCAN^52^ and SCAN0^53^) families. B1PW91 functional presents small (but non-negligible) oscillations in the correlation part, whereas the maximal deviations are twice larger in PW91 and mPW91. The SCAN family shows the highest maximal RMSE values for the exchange functional with the errors for the correlation functional being approximately half as large. In Section S5 of the Supporting Information, we present results obtained using renormalized versions of SCAN, namely rSCAN^54^ and r2SCAN.^55^ It is demonstrated that rSCAN leads to even larger oscillations than SCAN, while r2SCAN manages to partially reduce the oscillations in comparison to SCAN, although their magnitude remains considerably large.

The PBE (PBE,^56^ PBE0,^57^ PBE50,^21^ and LC-ωPBE^58^), and TPSS (TPSS,^59^ RevTPSS,^60^ and TPSSh^61^) families present small oscillations separately for both exchange and correlation functionals, always within the limit we have set (RMSE = 0.01), and the sum of their absolute values exceeds this boundary, resulting in noticeable spurious oscillations. M11 and MN12 families both present large RMSE values for both exchange and correlation functionals.

In the B97 family (B97,^62^ B97-D,^63^ ωB97,^35^ ωB97X,^35^ ωB97X-D,^64^ ωB97X-D3,^65^ ωB97X-V,^66^ ωB97M-V,^67^ and B97M-V^68^) small spurious oscillations are only observed for the exchange part of all range-separated and hybrid exchange functionals. In contrast, the GGA (B97-D^63^) and the meta-GGA (ωB97M-V^68^) exhibit larger RMSE values. This is due to hybridized functionals reducing the amount of pure GGA exchange, which is more prone to numerical integration errors. For the SOGGA (SOGGA11^69^ and SOGGA11-X^70^), N12 (N12^71^ and N12-SX^72^), MN15 (MN15^73^ and MN15-L^74^), M06 (M06,^36^ M06-2X,^36^ M06-L,^75^ and M06-HF^76^), M11 (M11,^77^ M11-L^78^), MN12 (MN12^79^ and MN12-SX^72^) and VSCX^80^ families, large RMSE values are noted for both correlation and exchange functionals. However, these values are also mitigated for the exact exchange component in their hybrid members, such as SOGGA11-X, N12-SX, M06-2X, M06-HF, and MN12-SX. However, the error in the exchange functional remains substantial in the last four hybrid DFAs.

Finally, in the case of Ar_2_, we identify that the correlation part of the DFA is more prone to present spurious oscillations than exchange (see Table S30) regardless of the DFA—we expect a similar result for other molecules bound by dispersion interactions. In contrast, the exchange part is dominant for the appearance of spurious oscillations in H-bonded complexes.

## 5 Conclusions

In this study, we analyzed the varying susceptibility of different DFAs to present spurious oscillations along nuclear displacement coordinates.^11^ These oscillations cause important errors in various vibrational spectroscopic properties,^10,11,16^ which are commonly masked as performance errors. As we demonstrated in ref (11), these spurious oscillations exhibit grid sensitivity and often diminish for large grids. We note on passing that the grid-sensitivity behavior has also been identified in the context of single-point energy calculations in atomic systems.^12−15^

The present work hinges on a judicious strategy to dissect the exchange and correlation components of DFAs to pinpoint the origins of these spurious oscillations. Initially, we refined and optimized an algorithm for detecting spurious oscillations, aiming to reduce computational costs and isolate the cause of these oscillations. Our findings indicate that spurious oscillations are insensitive to the choice of basis sets. Small basis sets such as 6-31G and Def2-SVP are adequate for assessing their magnitude along a property profile near equilibrium, greatly reducing the computational cost of the assessment. Furthermore, we identified spurious oscillations in derivatives of energy components that do not involve numerical integrations. These *indirect* spurious oscillations are caused by suboptimal electron densities from a self-consistent field procedure involving a DFA that presents *direct* spurious oscillations originating from numerical integration errors in *E*_x_ and *E*_c_. *Indirect* oscillations mask the true extent of *direct* spurious oscillations, which are more pronounced than the oscillations we observed on the total energy. We prove that using FMO from the equilibrium geometry is an excellent choice for examining spurious oscillations in *E̅*_x_ and *E*_c_ because it significantly decreases the computational cost and eliminates *indirect errors*.

These insights have significantly simplified the analysis process and, most importantly, enabled a distinct identification of the impacts of spurious oscillations from exchange and correlation components. Our results reveal that some DFAs, such as PBE, appeared to be relatively insensitive to spurious oscillations due to cancellations between energy components. A thorough analysis of *direct* spurious oscillations reveals that only a handful of exchange and correlation components are insensitive to spurious oscillations, giving rise to three families of functionals: BH&H, LSDA, and BLYP. Among the functionals in these families, we encounter four widespread DFAs: BLYP, B3LYP, LC-BLYP, and CAM-B3LYP. Considering the importance of the electron delocalization error^3,81^ for various applications in computational chemistry,^82−85^ the latter two are the only safe options in a wide context. However, other popular functionals, such as those in PBE and TPSS families, present small errors and could be safely employed provided that a sufficiently large integration grid is used in the calculations.

The results of this study will serve as a starting point for developing accurate DFAs suitable for studying molecular spectroscopies. We are actively pursuing research in this area in our laboratories.

## Supporting Information Available

Graphics complementing those presented in Figures 1 to 5, additionally, functionals used in this work and grid errors for various grids, systems, and derivatives. The classification in Figure 6 is grounded in the information presented in Table S26 and its accompanying notes (PDF)

## Footnotes

a Should the calculation of a property involve numerical derivatives, the equilibrium SCF molecular orbitals can be reorthonormalized for the *M* points mandated by the numerical integration expression, yielding a cumulative reduction in computational cost of a factor of *MN*.

## References

[ref1] Becke A. D. Perspective: Fifty years of density-functional theory in chemical physics. J. Chem. Phys. 2014, 140, 18A301 10.1063/1.4869598. 24832308

[ref2] Burke K. Perspective on density functional theory. J. Chem. Phys. 2012, 136, 150901 10.1063/1.4704546. 22519306

[ref3] Cohen A. J.; Mori-Sánchez P.; Yang W. Challenges for density functional theory. Chem. Rev. 2012, 112, 289–794. 10.1021/cr200107z. 22191548

[ref4] Quintal M. M.; Karton A.; Iron M. A.; Boese A. D.; Martin J. M. Benchmark study of DFT functionals for late-transition-metal reactions. J. Phys. Chem. A 2006, 110, 709–716. 10.1021/jp054449w. 16405344

[ref5] Suellen C.; Freitas R. G.; Loos P.-F.; Jacquemin D. Cross-comparisons between experiment, TD-DFT, CC, and ADC for transition energies. J. Chem. Theory Comput. 2019, 15, 4581–4590. 10.1021/acs.jctc.9b00446. 31265781

[ref6] Besalú-Sala P.; Sitkiewicz S. P.; Salvador P.; Matito E.; Luis J. M. A new tuned range-separated density functional for the accurate calculation of second hyperpolarizabilities. Phys. Chem. Chem. Phys. 2020, 22, 11871–11880. 10.1039/D0CP01291B. 32441724

[ref7] Loos P.-F.; Comin M.; Blase X.; Jacquemin D. Reference energies for intramolecular charge-transfer excitations. J. Chem. Theory Comput. 2021, 17, 3666–3686. 10.1021/acs.jctc.1c00226. 33955742

[ref8] Liang J.; Feng X.; Hait D.; Head-Gordon M. Revisiting the performance of time-dependent density functional theory for electronic excitations: Assessment of 43 popular and recently developed functionals from rungs one to four. J. Chem. Theory Comput. 2022, 18, 3460–3473. 10.1021/acs.jctc.2c00160. 35533317

[ref9] Chołuj M.; Alam M. M.; Beerepoot M. T.; Sitkiewicz S. P.; Matito E.; Ruud K.; Zaleśny R. Choosing bad versus worse: predictions of two-photon-absorption strengths based on popular density functional approximations. J. Chem. Theory Comput. 2022, 18, 1046–1060. 10.1021/acs.jctc.1c01056. 35080389 PMC8830054

[ref10] Zaleśny R.; Medved’ M.; Sitkiewicz S. P.; Matito E.; Luis J. M. Can density functional theory be trusted for high-order electric properties? The case of hydrogen-bonded complexes. J. Chem. Theory Comput. 2019, 15, 3570–3579. 10.1021/acs.jctc.9b00139. 31082215

[ref11] Sitkiewicz S. P.; Zaleśny R.; Ramos-Cordoba E.; Luis J. M.; Matito E. How reliable are modern density functional approximations to simulate vibrational spectroscopies?. J. Phys. Chem. Lett. 2022, 13, 5963–5968. 10.1021/acs.jpclett.2c01278. 35735354 PMC9251762

[ref12] Lehtola S.; Marques M. A. Many recent density functionals are numerically ill-behaved. J. Chem. Phys. 2022, 157, 174114 10.1063/5.0121187. 36347696

[ref13] Lehtola S.; Marques M. A. Reproducibility of density functional approximations: how new functionals should be reported. J. Chem. Phys. 2023, 159, 114116 10.1063/5.0167763. 37725491

[ref14] Lehtola S. Meta-GGA density functional calculations on atoms with spherically symmetric densities in the finite element formalism. J. Chem. Theory Comput. 2023, 19, 2502–2517. 10.1021/acs.jctc.3c00183. 37084260 PMC10173457

[ref15] Lehtola S. Atomic electronic structure calculations with Hermite interpolating polynomials. J. Phys. Chem. A 2023, 127, 4180–4193. 10.1021/acs.jpca.3c00729. 37129275 PMC10184118

[ref16] Sitkiewicz S. P.; Matito E.; Luis J. M.; Zaleśny R. Pitfall in simulations of vibronic TD-DFT spectra: Diagnosis and assessment. Phys. Chem. Chem. Phys. 2023, 25, 30193–30197. 10.1039/D3CP04276F. 37905423

[ref17] Becke A. D. A multicenter numerical integration scheme for polyatomic molecules. J. Chem. Phys. 1988, 88, 2547–2553. 10.1063/1.454033.

[ref18] Lebedev V. I. Values of the nodes and weights of ninth to seventeenth order gauss-markov quadrature formulae invariant under the octahedron group with inversion. USSR Comput. Math. & Math. Phys. 1975, 15, 44–51. 10.1016/0041-5553(75)90133-0.

[ref19] Lebedev V. I. Spherical quadrature formulas exact to orders 25–29. Sib. Math. J. 1977, 18, 99–107. 10.1007/BF00966954.

[ref20] Lebedev V. I.; Laikov D. N. A quadrature formula for the sphere of the 131st algebraic order of accuracy. Doklady Mathematics 1999, 59, 477–481.

[ref21] Epifanovsky E.; Gilbert A. T. B.; Feng X.; Lee J.; Mao Y.; Mardirossian N.; Pokhilko P.; White A. F.; Coons M. P.; Dempwolff A. L.; et al. Software for the frontiers of quantum chemistry: An overview of developments in the Q-Chem 5 package. J. Chem. Phys. 2021, 155, 084801 10.1063/5.0055522. 34470363 PMC9984241

[ref22] Murray C. W.; Handy N. C.; Laming G. J. Quadrature schemes for integrals of density functional theory. Mol. Phys. 1993, 78, 997–1014. 10.1080/00268979300100651.

[ref23] Laqua H.; Kussmann J.; Ochsenfeld C. An improved molecular partitioning scheme for numerical quadratures in density functional theory. J. Chem. Phys. 2018, 149, 204111 10.1063/1.5049435. 30501270

[ref24] Treutler O.; Ahlrichs R. Efficient molecular numerical integration schemes. J. Chem. Phys. 1995, 102, 346–354. 10.1063/1.469408.

[ref25] Mura M. E.; Knowles P. J. Improved radial grids for quadrature in molecular density-functional calculations. J. Chem. Phys. 1996, 104, 9848–9858. 10.1063/1.471749.

[ref26] Stratmann R. E.; Scuseria G. E.; Frisch M. J. Achieving linear scaling in exchange-correlation density functional quadratures. Chem. Phys. Lett. 1996, 257, 213–223. 10.1016/0009-2614(96)00600-8.

[ref27] Turney J. M.; Simmonett A. C.; Parrish R. M.; Hohenstein E. G.; Evangelista F. A.; Fermann J. T.; Mintz B. J.; Burns L. A.; Wilke J. J.; Abrams M. L.; et al. Psi4: an open-source ab initio electronic structure program. WIREs, Comput. Mol. Sci. 2012, 2, 556–565. 10.1002/wcms.93.

[ref28] Parrish R. M.; Burns L. A.; Smith D. G.; Simmonett A. C.; DePrince A. E.; Hohenstein E. G.; Bozkaya U.; Sokolov A. Y.; Di Remigio R.; Richard R. M.; et al. Psi4 1.1: an open-source electronic structure program emphasizing automation, advanced libraries, and interoperability. J. Chem. Theory Comput. 2017, 13, 3185–3197. 10.1021/acs.jctc.7b00174. 28489372 PMC7495355

[ref29] Smith D. G.; Burns L. A.; Sirianni D. A.; Nascimento D. R.; Kumar A.; James A. M.; Schriber J. B.; Zhang T.; Zhang B.; Abbott A. S.; et al. Psi4NumPy: an interactive quantum chemistry programming environment for reference implementations and rapid development. J. Chem. Theory Comput. 2018, 14, 3504–3511. 10.1021/acs.jctc.8b00286. 29771539

[ref30] Luis J. M.; Duran M.; Champagne B.; Kirtman B. Determination of vibrational polarizabilities and hyperpolarizabilities using field-induced coordinates. J. Chem. Phys. 2000, 113, 5203–5213. 10.1063/1.1290022.

[ref31] Luis J. M.; Duran M.; Kirtman B. Field-induced coordinates for the determination of dynamic vibrational nonlinear optical properties. J. Chem. Phys. 2001, 115, 4473–4483. 10.1063/1.1390525.

[ref32] Kirtman B.; Champagne B.; Luis J. M. Efficient treatment of the effect of vibrations on electrical, magnetic, and spectroscopic properties. J. Comput. Chem. 2000, 21, 1572–1588. 10.1002/1096-987X(200012)21:16<1572::AID-JCC14>3.0.CO;2-8.

[ref33] Dasgupta S.; Herbert J. M. Standard grids for high-precision integration of modern density functionals: SG-2 and SG-3. J. Comput. Chem. 2017, 38, 869–882. 10.1002/jcc.24761. 28233907

[ref34] Hunter J. D. Matplotlib: A 2D graphics environment. Comput. Sci. Eng. 2007, 9, 90–95. 10.1109/MCSE.2007.55.

[ref35] Chai J.-D.; Head-Gordon M. Systematic optimization of long-range corrected hybrid density functionals. J. Chem. Phys. 2008, 128, 084106 10.1063/1.2834918. 18315032

[ref36] Zhao Y.; Truhlar D. The M06 suite of density functionals for main group thermochemistry, thermochemical kinetics, noncovalent interactions, excited states, and transition elements: two new functionals and systematic testing of four M06-class functionals and 12 other functionals. Theor. Chem. Acc. 2008, 120, 215–241. 10.1007/s00214-007-0310-x.

[ref37] Becke A. D. Density-functional thermochemistry. III. The role of exact exchange. J. Chem. Phys. 1993, 98, 5648–5652. 10.1063/1.464913.

[ref38] Stephens P. J.; Devlin F. J.; Chabalowski C. F.; Frisch M. J. Ab initio calculation of vibrational absorption and circular dichroism spectra using density functional force fields. J. Phys. Chem. 1994, 98, 11623–11627. 10.1021/j100096a001.

[ref39] Van Lenthe J. H.; Zwaans R.; Van Dam H. J. J.; Guest M. F. Starting SCF calculations by superposition of atomic densities. J. Comput. Chem. 2006, 27, 926–932. 10.1002/jcc.20393. 16557519

[ref40] Becke A. D. A new mixing of Hartree-Fock and local density-functional theories. J. Chem. Phys. 1993, 98, 1372–1377. 10.1063/1.464304.

[ref41] Dirac P. A. M. Note on exchange phenomena in the Thomas atom. Math. Proc. Cambridge Philos. Soc. 1930, 26, 376–385. 10.1017/S0305004100016108.

[ref42] Vosko S. H.; Wilk L.; Nusair M. Accurate spin-dependent electron liquid correlation energies for local spin density calculations: a critical analysis. Can. J. Phys. 1980, 58, 1200–1211. 10.1139/p80-159.

[ref43] Perdew J. P.; Wang Y. Accurate and simple analytic representation of the electron-gas correlation energy. Phys. Rev. B 1992, 45, 13244–13249. 10.1103/PhysRevB.45.13244. 10001404

[ref44] Becke A. D. Density-functional exchange-energy approximation with correct asymptotic behavior. Phys. Rev. A 1988, 38, 3098–3100. 10.1103/PhysRevA.38.3098. 9900728

[ref45] Lee C.; Yang W.; Parr R. G. Development of the Colle-Salvetti correlation-energy formula into a functional of the electron density. Phys. Rev. B 1988, 37, 785–789. 10.1103/PhysRevB.37.785. 9944570

[ref46] Adamo C.; Barone V. Toward reliable adiabatic connection models free from adjustable parameters. Chem. Phys. Lett. 1997, 274, 242–250. 10.1016/S0009-2614(97)00651-9.

[ref47] Frisch M. J.; Trucks G. W.; Schlegel H. B.; Scuseria G. E.; Robb M. A.; Cheeseman J. R.; Scalmani G.; Barone V.; Petersson G. A.; Nakatsuji H.; Gaussian 16. Revision C.01; Gaussian Inc: Wallingford CT, 2016.

[ref48] Tawada Y.; Tsuneda T.; Yanagisawa S.; Yanai T.; Hirao K. A long-range-corrected time-dependent density functional theory. J. Chem. Phys. 2004, 120, 8425–8433. 10.1063/1.1688752. 15267767

[ref49] Yanai T.; Tew D. P.; Handy N. C. A new hybrid exchange–correlation functional using the Coulomb-attenuating method (CAM-B3LYP). Chem. Phys. Lett. 2004, 393, 51–57. 10.1016/j.cplett.2004.06.011.

[ref50] Perdew J. P.; Chevary J. A.; Vosko S. H.; Jackson K. A.; Pederson M. R.; Singh D. J.; Fiolhais C. Atoms, molecules, solids, and surfaces: applications of the generalized gradient approximation for exchange and correlation. Phys. Rev. B 1992, 46, 6671–6687. 10.1103/PhysRevB.46.6671. 10002368

[ref51] Adamo C.; Barone V. Exchange functionals with improved long-range behavior and adiabatic connection methods without adjustable parameters: the mPW and mPW1PW models. J. Chem. Phys. 1998, 108, 664–675. 10.1063/1.475428.

[ref52] Sun J.; Ruzsinszky A.; Perdew J. P. Strongly constrained and appropriately normed semilocal density functional. Phys. Rev. Lett. 2015, 115, 036402 10.1103/PhysRevLett.115.036402. 26230809

[ref53] Hui K.; Chai J.-D. SCAN-based hybrid and double-hybrid density functionals from models without fitted parameters. J. Chem. Phys. 2016, 144, 044114 10.1063/1.4940734. 26827209

[ref54] Bartók A. P.; Yates J. R. Regularized SCAN functional. J. Chem. Phys. 2019, 150, 161101 10.1063/1.5094646. 31042928

[ref55] Furness J. W.; Kaplan A. D.; Ning J.; Perdew J. P.; Sun J. Accurate and numerically efficient r2SCAN meta-generalized gradient approximation. J. Phys. Chem. Lett. 2020, 11, 8208–8215. 10.1021/acs.jpclett.0c02405. 32876454

[ref56] Perdew J. P.; Burke K.; Ernzerhof M. Generalized gradient approximation made simple. Phys. Rev. Lett. 1996, 77, 3865–3868. 10.1103/PhysRevLett.77.3865. 10062328

[ref57] Adamo C.; Barone V. Toward reliable density functional methods without adjustable parameters: The PBE0 model. J. Chem. Phys. 1999, 110, 6158–6170. 10.1063/1.478522.

[ref58] Rohrdanz M. A.; Herbert J. M. Simultaneous benchmarking of ground- and excited-state properties with long-range-corrected density functional theory. J. Chem. Phys. 2008, 129, 034107 10.1063/1.2954017. 18647016

[ref59] Tao J.; Perdew J. P.; Staroverov V. N.; Scuseria G. E. Climbing the density functional ladder: nonempirical meta–generalized gradient approximation designed for molecules and solids. Phys. Rev. Lett. 2003, 91, 146401 10.1103/PhysRevLett.91.146401. 14611541

[ref60] Perdew J. P.; Ruzsinszky A.; Csonka G. I.; Constantin L. A.; Sun J. Workhorse semilocal density functional for condensed matter physics and quantum chemistry. Phys. Rev. Lett. 2009, 103, 026403 10.1103/PhysRevLett.103.026403. 19659225

[ref61] Staroverov V. N.; Scuseria G. E.; Tao J.; Perdew J. P. Comparative assessment of a new nonempirical density functional: molecules and hydrogen-bonded complexes. J. Chem. Phys. 2003, 119, 12129–12137. 10.1063/1.1626543.

[ref62] Becke A. D. Density-functional thermochemistry. V. Systematic optimization of exchange-correlation functionals. J. Chem. Phys. 1997, 107, 8554–8560. 10.1063/1.475007.

[ref63] Grimme S. Semiempirical GGA-type density functional constructed with a long-range dispersion correction. J. Comput. Chem. 2006, 27, 1787–1799. 10.1002/jcc.20495. 16955487

[ref64] Chai J.-D.; Head-Gordon M. Long-range corrected hybrid density functionals with damped atom–atom dispersion corrections. Phys. Chem. Chem. Phys. 2008, 10, 6615–6620. 10.1039/b810189b. 18989472

[ref65] Lin Y.-S.; Li G.-D.; Mao S.-P.; Chai J.-D. Long-range corrected hybrid density functionals with improved dispersion corrections. J. Chem. Theory Comput. 2013, 9, 263–272. 10.1021/ct300715s. 26589028

[ref66] Mardirossian N.; Head-Gordon M. ωB97X-V: A 10-parameter, range-separated hybrid, generalized gradient approximation density functional with nonlocal correlation, designed by a survival-of-the-fittest strategy. Phys. Chem. Chem. Phys. 2014, 16, 9904–9924. 10.1039/c3cp54374a. 24430168

[ref67] Mardirossian N.; Head-Gordon M. Mapping the genome of meta-generalized gradient approximation density functionals: The search for B97M-V. J. Chem. Phys. 2015, 142, 074111 10.1063/1.4907719. 25702006

[ref68] Mardirossian N.; Head-Gordon M. ωB97M-V: A combinatorially optimized, range-separated hybrid, meta-GGA density functional with VV10 nonlocal correlation. J. Chem. Phys. 2016, 144, 214110 10.1063/1.4952647. 27276948

[ref69] Peverati R.; Zhao Y.; Truhlar D. G. Generalized gradient approximation that recovers the second-order density-gradient expansion with optimized across-the-board performance. J. Phys. Chem. Lett. 2011, 2, 1991–1997. 10.1021/jz200616w.

[ref70] Peverati R.; Truhlar D. G. Communication: A global hybrid generalized gradient approximation to the exchange-correlation functional that satisfies the second-order density-gradient constraint and has broad applicability in chemistry. J. Chem. Phys. 2011, 135, 191102 10.1063/1.3663871. 22112059 PMC3248024

[ref71] Peverati R.; Truhlar D. G. Exchange–Correlation Functional with Good Accuracy for Both Structural and Energetic Properties while Depending Only on the Density and Its Gradient. J. Chem. Theory Comput. 2012, 8, 2310–2319. 10.1021/ct3002656. 26588964

[ref72] Peverati R.; Truhlar D. G. Screened-exchange density functionals with broad accuracy for chemistry and solid-state physics. Phys. Chem. Chem. Phys. 2012, 14, 16187–16191. 10.1039/c2cp42576a. 23132141

[ref73] Yu H. S.; He X.; Li S. L.; Truhlar D. G. MN15: A Kohn–Sham global-hybrid exchange–correlation density functional with broad accuracy for multi-reference and single-reference systems and noncovalent interactions. Chem. Sci. 2016, 7, 5032–5051. 10.1039/C6SC00705H. 30155154 PMC6018516

[ref74] Yu H. S.; He X.; Truhlar D. G. MN15-L: a new local exchange-correlation functional for Kohn–Sham density functional theory with broad accuracy for atoms, molecules, and solids. J. Chem. Theory Comput. 2016, 12, 1280–1293. 10.1021/acs.jctc.5b01082. 26722866

[ref75] Zhao Y.; Truhlar D. G. A new local density functional for main-group thermochemistry, transition metal bonding, thermochemical kinetics, and noncovalent interactions. J. Chem. Phys. 2006, 125, 194101 10.1063/1.2370993. 17129083

[ref76] Zhao Y.; Truhlar D. G. Density functional for spectroscopy: no long-range self-interaction error, good performance for Rydberg and charge-transfer states, and better performance on average than B3LYP for ground states. J. Phys. Chem. A 2006, 110, 13126–13130. 10.1021/jp066479k. 17149824

[ref77] Peverati R.; Truhlar D. G. Improving the accuracy of hybrid meta-GGA density functionals by range separation. J. Phys. Chem. Lett. 2011, 2, 2810–2817. 10.1021/jz201170d.

[ref78] Peverati R.; Truhlar D. G. M11-L: a local density functional that provides improved accuracy for electronic structure calculations in chemistry and physics. J. Phys. Chem. Lett. 2012, 3, 117–124. 10.1021/jz201525m. 22910998

[ref79] Peverati R.; Truhlar D. G. An improved and broadly accurate local approximation to the exchange–correlation density functional: The MN12-L functional for electronic structure calculations in chemistry and physics. Phys. Chem. Chem. Phys. 2012, 14, 13171–13174. 10.1039/c2cp42025b. 22910998

[ref80] Van Voorhis T.; Scuseria G. E. A novel form for the exchange-correlation energy functional. J. Chem. Phys. 1998, 109, 400–410. 10.1063/1.476577.

[ref81] Bryenton K. R.; Adeleke A. A.; Dale S. G.; Johnson E. R. Delocalization error: The greatest outstanding challenge in density-functional theory. WIREs, Comput. Mol. Sci. 2023, 13, e1631 10.1002/wcms.1631.

[ref82] Autschbach J.; Srebro M. Delocalization error and “functional tuning” in Kohn–Sham calculations of molecular properties. Acc. Chem. Res. 2014, 47, 2592–2602. 10.1021/ar500171t. 24968277

[ref83] Casademont-Reig I.; Woller T.; Contreras-García J.; Alonso M.; Torrent-Sucarrat M.; Matito E. New electron delocalization tools to describe the aromaticity in porphyrinoids. Phys. Chem. Chem. Phys. 2018, 20, 2787–2796. 10.1039/C7CP07581B. 29323373

[ref84] Lescos L.; Sitkiewicz S. P.; Beaujean P.; Blanchard-Desce M.; Champagne B.; Matito E.; Castet F. Performance of DFT functionals for calculating the second-order nonlinear optical properties of dipolar merocyanines. Phys. Chem. Chem. Phys. 2020, 22, 16579–16594. 10.1039/D0CP02992K. 32677655

[ref85] Casademont-Reig I.; Guerrero-Avilés R.; Ramos-Cordoba E.; Torrent-Sucarrat M.; Matito E. How aromatic ara molecular nanorings? The case of a six-porphyrin nanoring. Angew. Chem., Int. Ed. 2021, 60, 24080–24088. 10.1002/anie.202108997. PMC8596448 34260804

