# Supplementary material for: Spurious Oscillations Caused by Density Functional Approximations: Who is to Blame? Exchange or Correlation?
Source: J Chem Theory Comput. 2024 Apr 3;20(8):3144–53. doi: 10.1021/acs.jctc.3c01339 (PMC11044272; doi:10.1021/acs.jctc.3c01339)
Supplement: Supplementary file 1 — ct3c01339_si_001.pdf [file ct3c01339_si_001.pdf]

SUPPORTING INFORMATION

Spurious Oscillations Caused by Density Functional Approximations: Who is to Blame, Exchange or Correlation?

Sebastian P. Sitkiewicz,[*,†,‡] Rubén R. Ferradás,[†] Eloy Ramos-Cordoba,[†,¶,§,||] Robert Zaleśny,[⊥] Eduard Matito,[*,†,§] and Josep M. Luis[*,#]

†Donostia International Physics Center (DIPC), 20018 Donostia, Euskadi, Spain

‡Wrocław Centre for Networking and Supercomputing, Wrocław University of Science and Technology, Wyb. Wyspiańskiego 27, PL–50370 Wrocław, Poland

¶Polimero eta Material Aurreratuak: Fisika, Kimika eta Teknologia, Kimika Fakultatea, Euskal Herriko Unibertsitatea UPV/EHU, P.K. 1072, 20080 Donostia, Euskadi, Spain.

§Ikerbasque Foundation for Science, Plaza Euskadi 5, 48009 Bilbao, Euskadi, Spain

||Institute for Advanced Chemistry of Catalonia (IQAC), CSIC, Jordi Girona 18-26, 08034 Barcelona, Spain

⊥Faculty of Chemistry, Wrocław University of Science and Technology, Wyb. Wyspiańskiego 27, PL–50370 Wrocław, Poland

#Institut de Química Computacional i Catàlisi (IQCC) and Departament de Química, Universitat de Girona, 17003 Girona, Catalonia, Spain

E-mail: sebastian.p.sitkiewicz@gmail.com; ematito@gmail.com; josepm.luis@udg.edu

Contents

S1 Supplementary Equations 3

S2 Supplementary Figures 3

S3 Supplementary Tables 29

S4 The role of integration grids 59

S5 SCAN density functional approximations 64

References 67

S1 Supplementary Equations

$$\chi_{1,z} = -\sum_{\nu=1}^{3M-6} Q_\nu q_1^{\nu,z} \quad \text{and} \quad q_1^{\nu,z} = \frac{1}{2k_\nu}\left(\frac{\mathrm{d}\mu_z}{\mathrm{d}Q_\nu}\right)_{\boldsymbol{Q}=0}. \tag{S1}$$

In above equation, Q_ν is the ν–th normal mode with a corresponding force constant k_ν, and μ_z is z–th component of the static dipole moment (obtained for the optimized geometry). FIC1 is a linear combination of harmonic normal modes and represents an overall response of vibrations to an external electric field. It has proven itself very useful for computation of the nuclear relaxation contributions to nonlinear optical properties.

S2 Supplementary Figures

The energy obtained using a DFA X and a density Y will be indicated as X@Y.

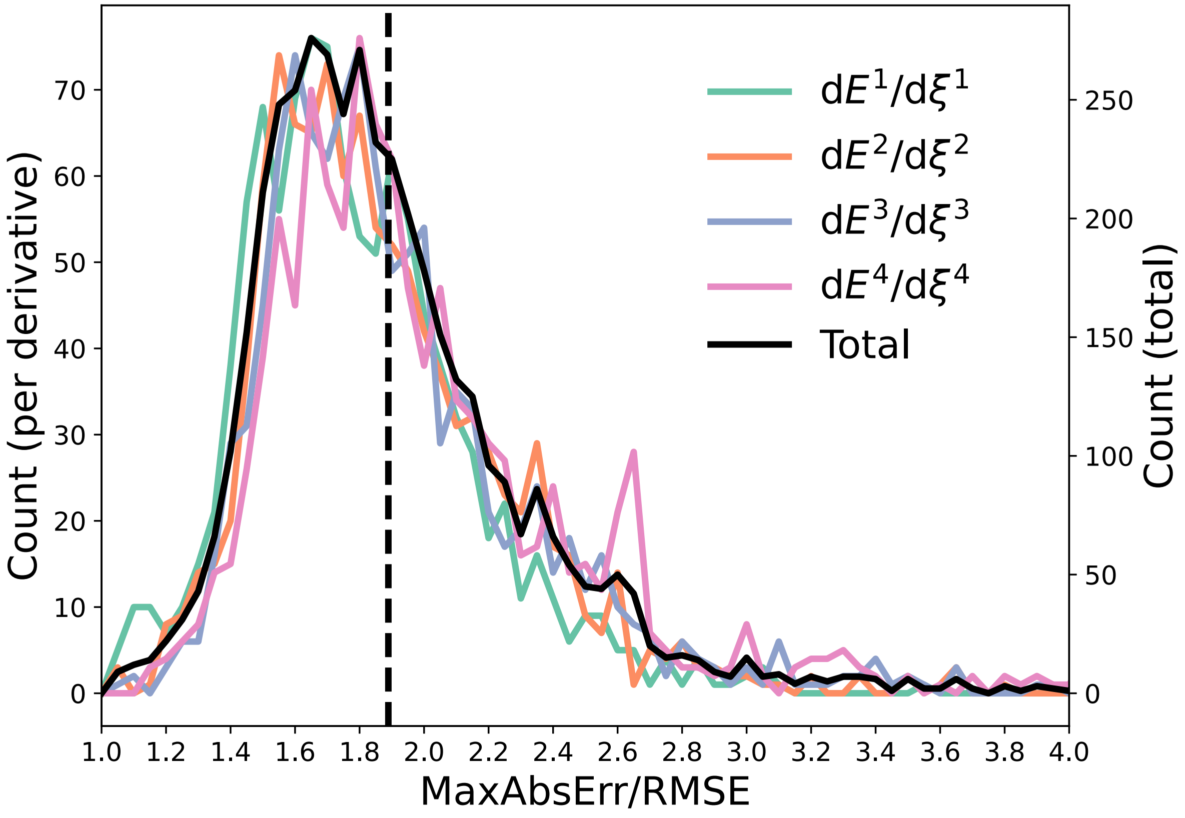

Figure S1: Distribution of values of the ratio between the maximum absolute error (MaxAbsErr) and the Root Mean Square Error (RMSE) measured over the same range of interatomic displacements for each property, with the data bin interval of 0.05, for different order of the $\mathrm{d}^m E/\mathrm{d}\xi^m$ derivatives (m = 1 - 4), as well as their overall sum (labeled as Total). The values included all tested DFAs, three integration grids, and all molecular complexes. The vertical dash line marks the average value of MaxAbsErr/RMSE (1.87).

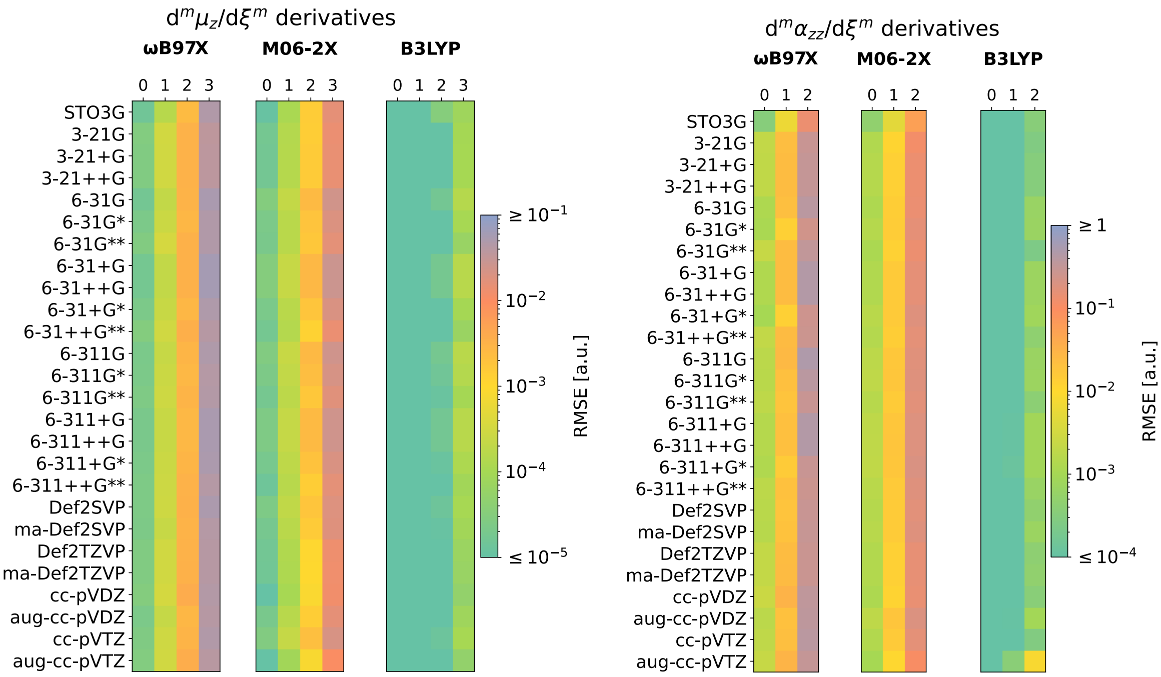

Figure S2: Study of the basis set dependence of the RMSE values in the the $d^m\mu_z/d\xi^m$ derivatives (*left panel*, m = 0 - 3, stored column-wise), and the $d^m\alpha_{zz}/d\xi^m$ derivatives (*right panel*, m = 0 - 2, stored column-wise) of HCN$\cdot$HF using ωB97X, M06-2X, and B3LYP combined with the (99, 590) integration grid. Colors reflect the values of RMSE in the property (note the logarithmic scale). Raw data is compiled in Tables S3 to S8.

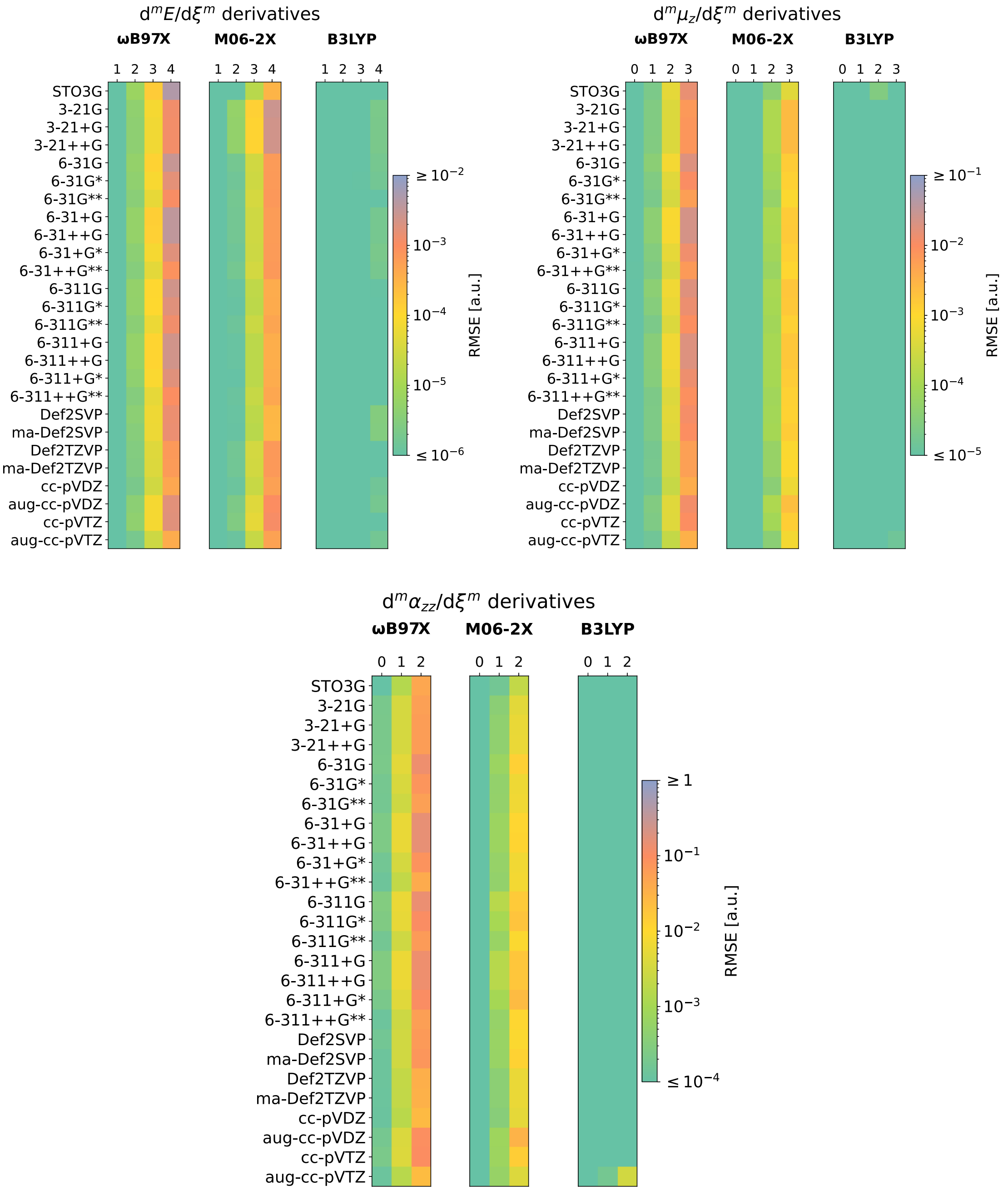

Figure S3: Study of the basis set dependence of the RMSE values in the $\mathrm{d}^m E/\mathrm{d}\chi_{1,z}{}^m$ derivatives (*left panel*, m= 1 - 4, stored column-wise), the $\mathrm{d}^m\mu_z/\mathrm{d}\xi^m$ derivatives (*middle panel*, m = 0 - 3, stored column-wise), and the $\mathrm{d}^m\alpha_{zz}/\mathrm{d}\xi^m$ derivatives (*right panel*, m = 0 - 2, stored column-wise) of HCN $\cdot$ HF using ωB97X, M06-2X, and B3LYP combined with the (250, 974) integration grid. Colors reflect the values of RMSE in the property (note the logarithmic scale). Raw data compiled in Tables S3 to S8.

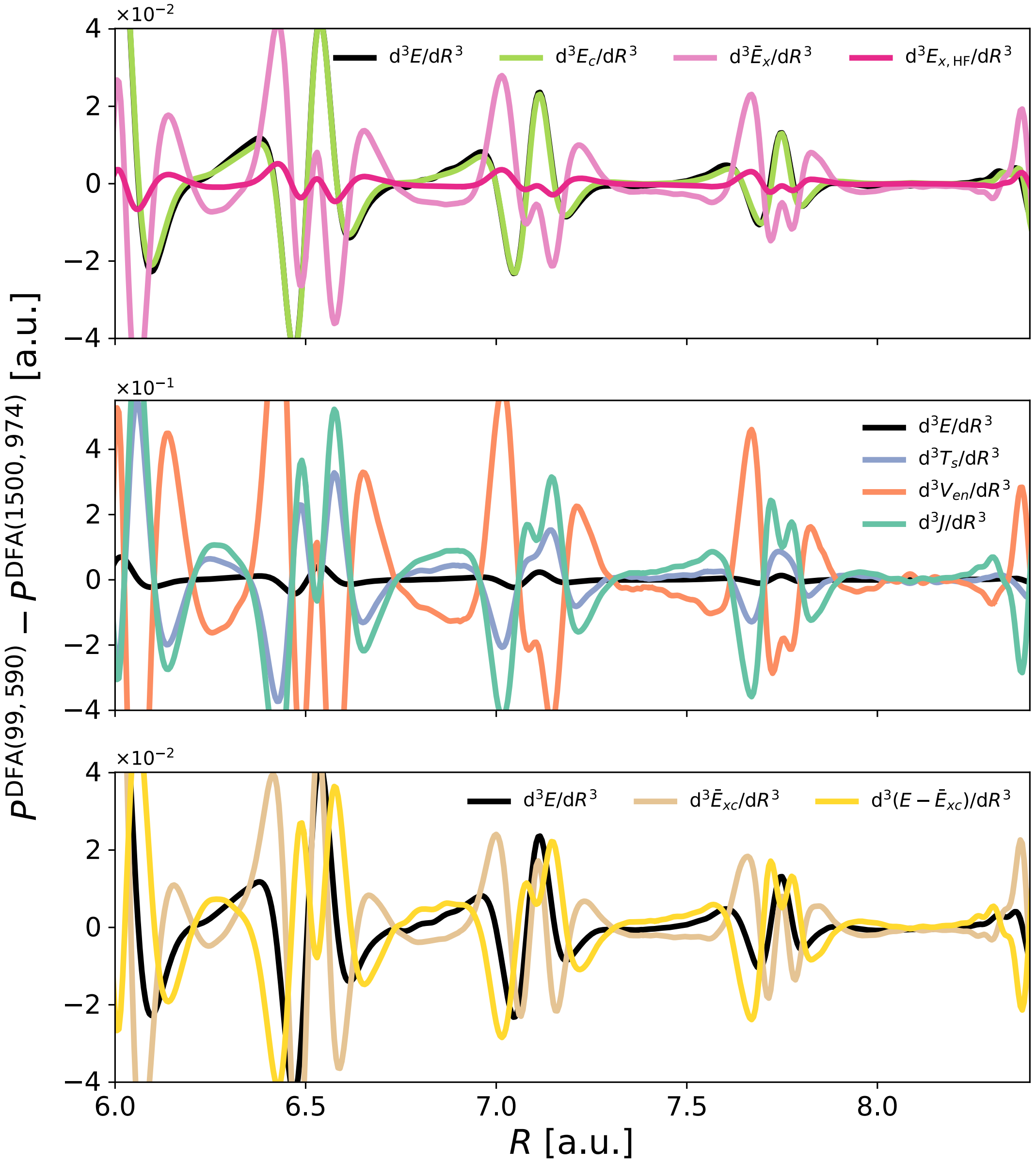

Figure S4: RMSE values capture spurious oscillations in the third derivatives of energy components of Ar_2: total electronic (E), kinetic (T), nuclei attraction (V_{en}), Coulomb repulsion (J), exact HF-like exchange $E_{x,\mathrm{HF}}$, exchange (E_x), and correlation (E_c), as well as their sums. Obtained with B97/6-31+G* as the difference between (99, 590) and (1500, 974) integration grids.

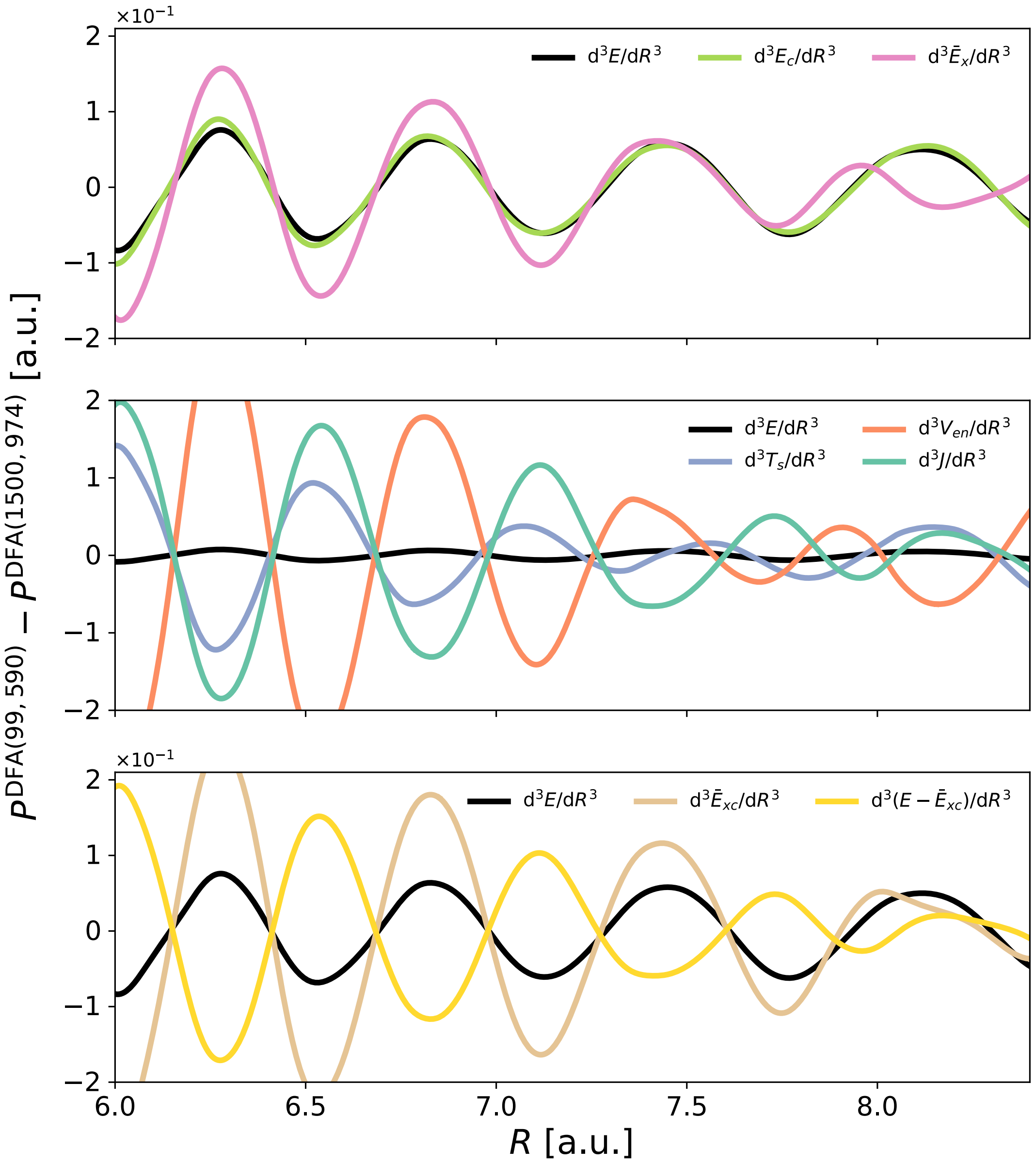

Figure S5: RMSE values capture spurious oscillations in the third derivatives of energy components of Ar_2: total electronic (E), kinetic (T), nuclei attraction (V_{en}), Coulomb repulsion (J), exact HF-like exchange $E_{x,\mathrm{HF}}$, exchange (E_x), and correlation (E_c), as well as their sums. Obtained with VSXC/6-31+G* as the difference between (99, 590) and (1500, 974) integration grids.

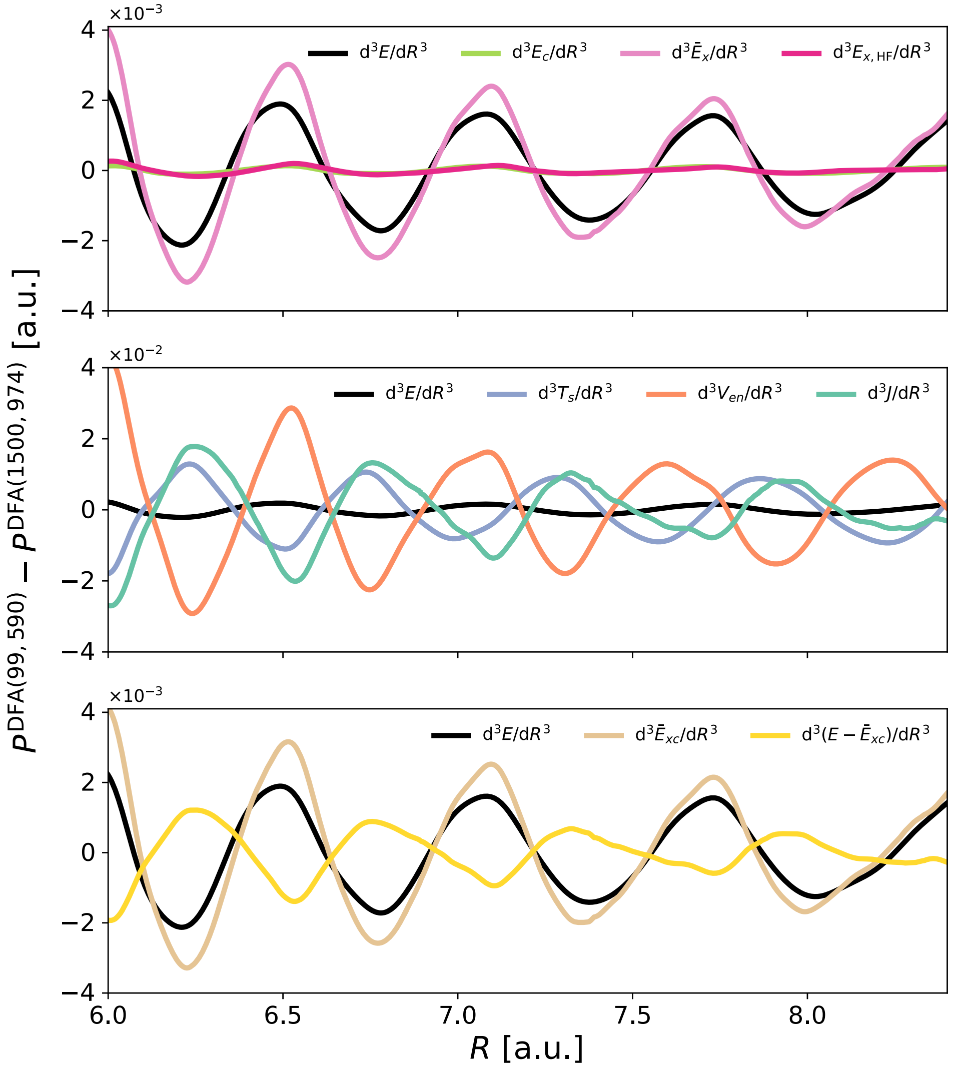

Figure S6: RMSE values capture spurious oscillations in the third derivatives of energy components of Ar_2: total electronic (E), kinetic (T), nuclei attraction (V_{en}), Coulomb repulsion (J), exact HF-like exchange $E_{x,\mathrm{HF}}$, exchange (E_x), and correlation (E_c), as well as their sums. Obtained with B3LYP/6-31+G* as the difference between (99, 590) and (1500, 974) integration grids.

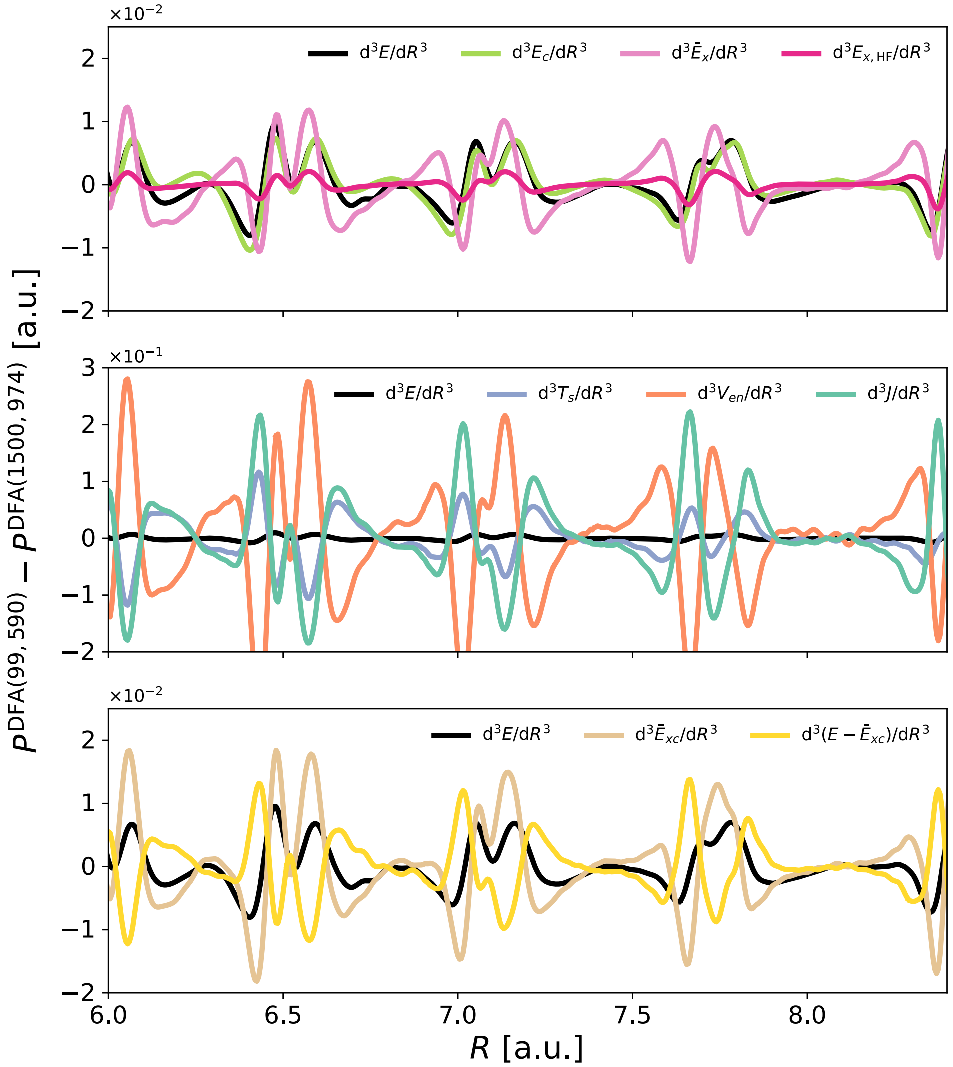

Figure S7: RMSE values capture spurious oscillations in the third derivatives of energy components of Ar_2: total electronic (E), kinetic (T), nuclei attraction (V_{en}), Coulomb repulsion (J), exact HF-like exchange $E_{x,\text{HF}}$, exchange (E_x), and correlation (E_c), as well as their sums. Obtained with ωB97M-V/6-31+G* as the difference between (99, 590) and (1500, 974) integration grids.

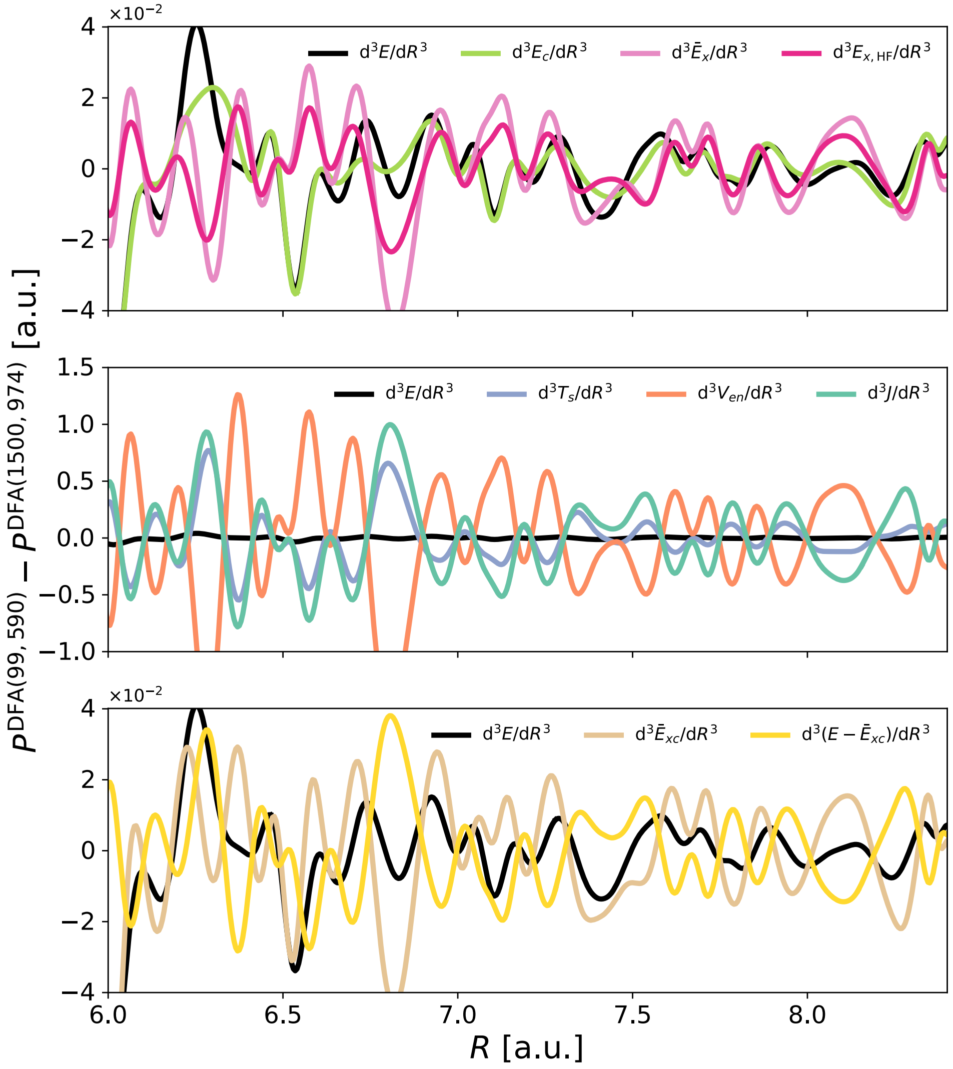

Figure S8: RMSE values capture spurious oscillations in the third derivatives of energy components of Ar_2: total electronic (E), kinetic (T), nuclei attraction (V_{en}), Coulomb repulsion (J), exact HF-like exchange $E_{x,\mathrm{HF}}$, exchange (E_x), and correlation (E_c), as well as their sums. Obtained with M06-2X/6-31+G* as the difference between (99, 590) and (1500, 974) integration grids.

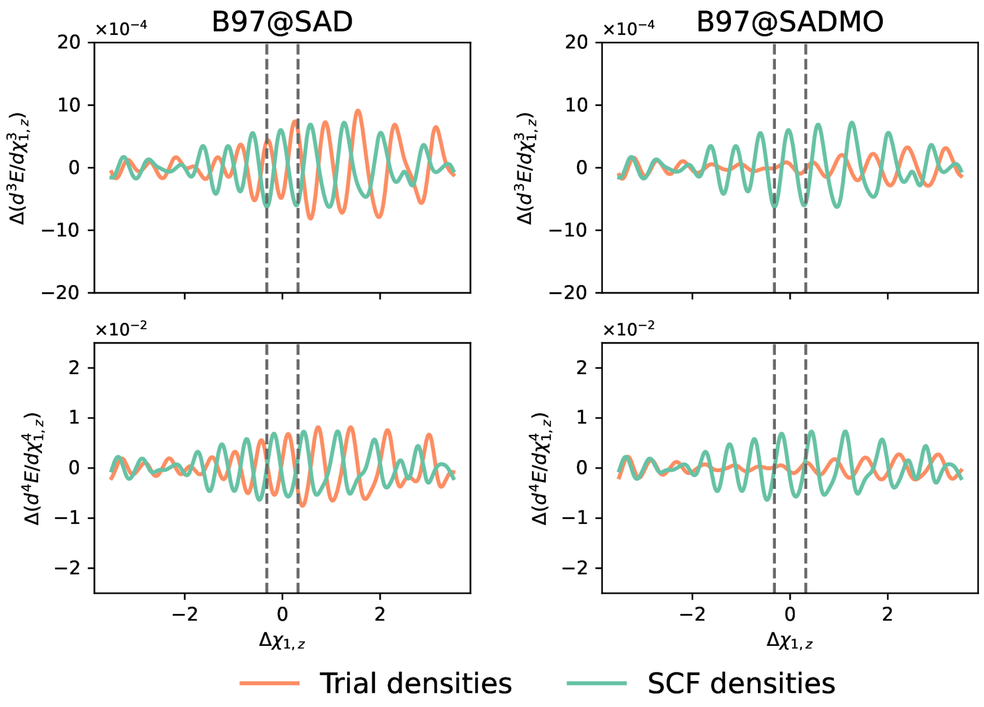

Figure S9: Spurious oscillations in $\mathrm{d}^3E/\mathrm{d}\chi_{1,z}{}^3$ and $\mathrm{d}^4E/\mathrm{d}\chi_{1,z}{}^4$ of the HCN·HF system, defined as $\Delta P = P^{\mathrm{DFA}(99,\ 590)} - P^{\mathrm{DFA}(1500,\ 974)}$, obtained with B97/6-31+G* and the trial densities (orange curves) and the SCF density (green curves, shown on each plot). Vertical dashed lines mark the displacement range of the property curve for which RMSE, and RRMSE are calculated.

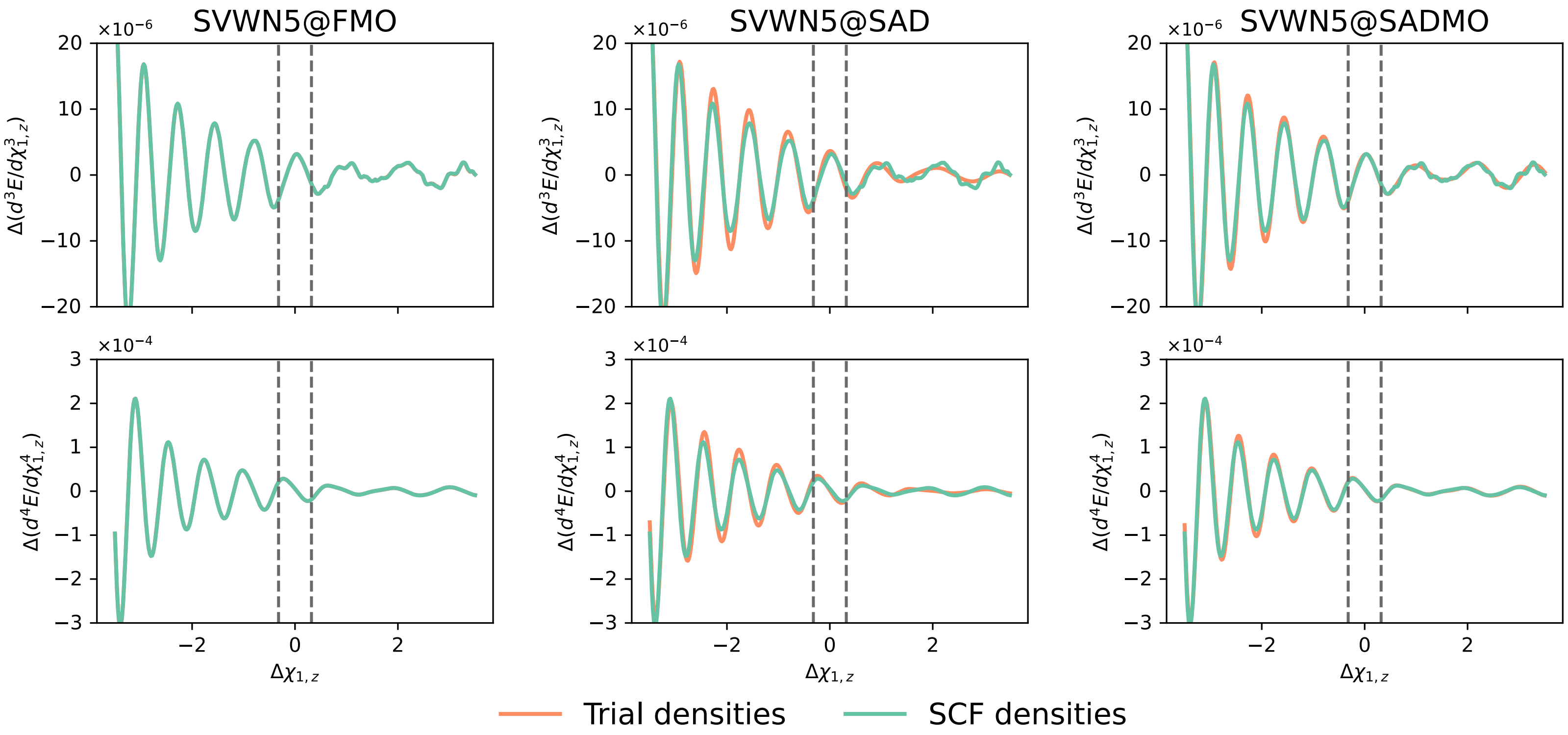

Figure S10: Spurious oscillations in $\mathrm{d}^3E/\mathrm{d}\chi_{1,z}{}^3$ and $\mathrm{d}^4E/\mathrm{d}\chi_{1,z}{}^4$ of the HCN · HF system, defined as $\Delta P = P^{\mathrm{DFA}(99,\ 590)} - P^{\mathrm{DFA}(1500,\ 974)}$, obtained with SVWN5/6-31+G* and the trial densities (orange curves) and the SCF density (green curves, shown on each plot). Vertical dashed lines mark the displacement range of the property curve for which RMSE, and RRMSE are calculated.

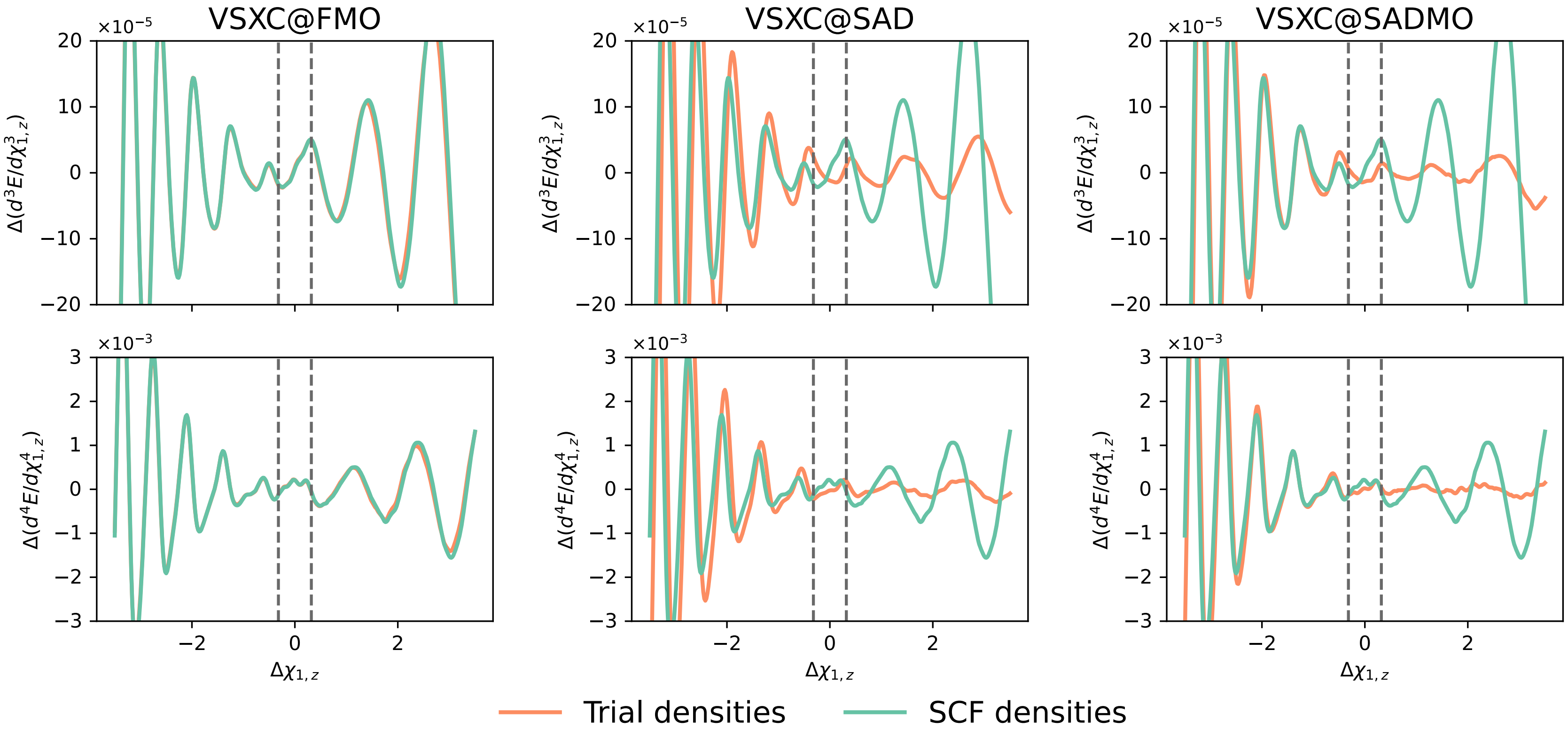

Figure S11: Spurious oscillations in $\mathrm{d}^3E/\mathrm{d}\chi_{1,z}{}^3$ and $\mathrm{d}^4E/\mathrm{d}\chi_{1,z}{}^4$ of the HCN $\cdot$ HF system, defined as $\Delta P = P^{\mathrm{DFA}(99,\ 590)} - P^{\mathrm{DFA}(1500,\ 974)}$, obtained with VSXC/6-31+G* and the trial densities (orange curves) and the SCF density (green curves, shown on each plot). Vertical dashed lines mark the displacement range of the property curve for which RMSE, and RRMSE are calculated.

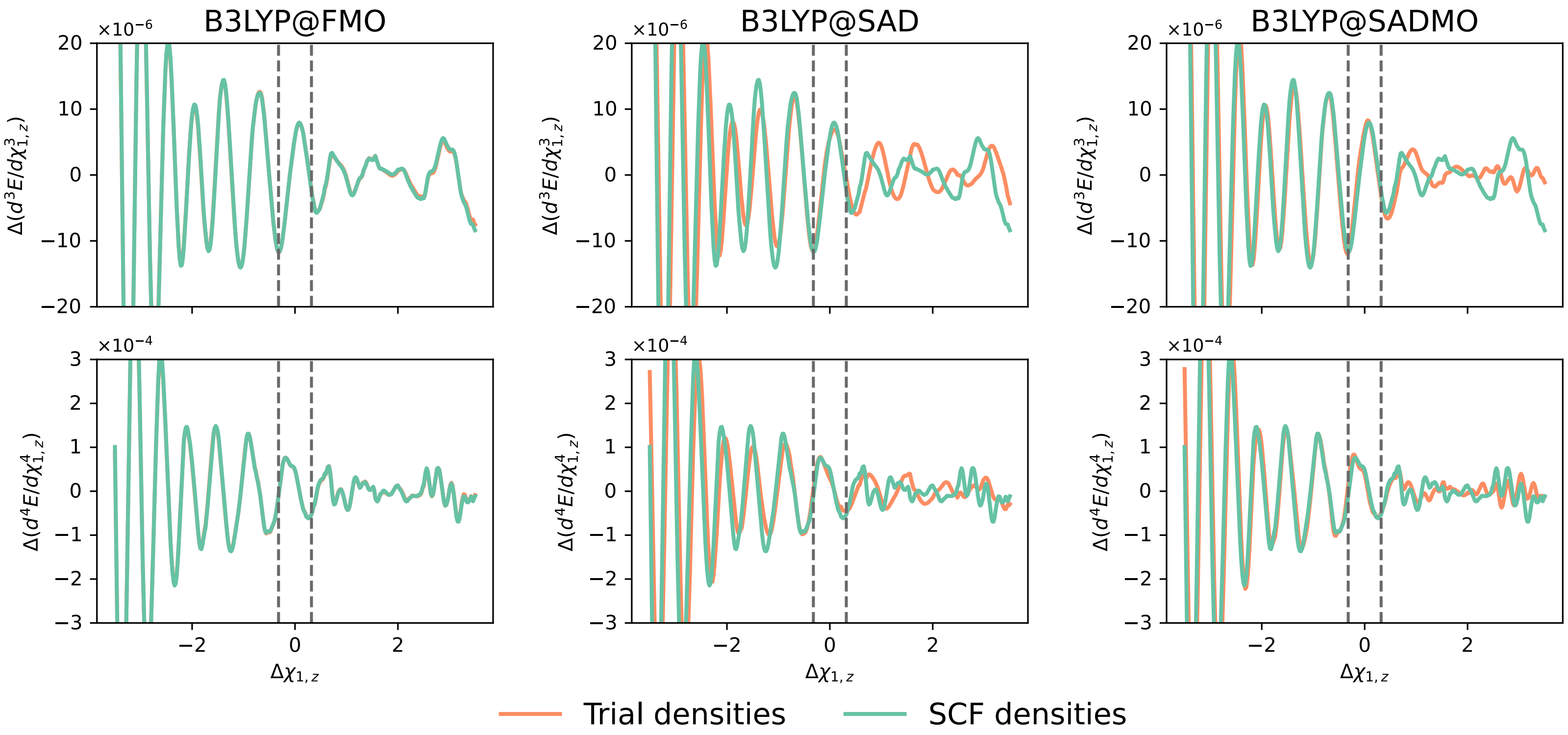

Figure S12: Spurious oscillations in $\mathrm{d}^3E/\mathrm{d}\chi_{1,z}{}^3$ and $\mathrm{d}^4E/\mathrm{d}\chi_{1,z}{}^4$ of the HCN · HF system, defined as $\Delta P = P^{\mathrm{DFA}(99,\ 590)} - P^{\mathrm{DFA}(1500,\ 974)}$, obtained with B3LYP/6-31+G* and the trial densities (orange curves) and the SCF density (green curves, shown on each plot). Vertical dashed lines mark the displacement range of the property curve for which RMSE, and RRMSE are calculated.

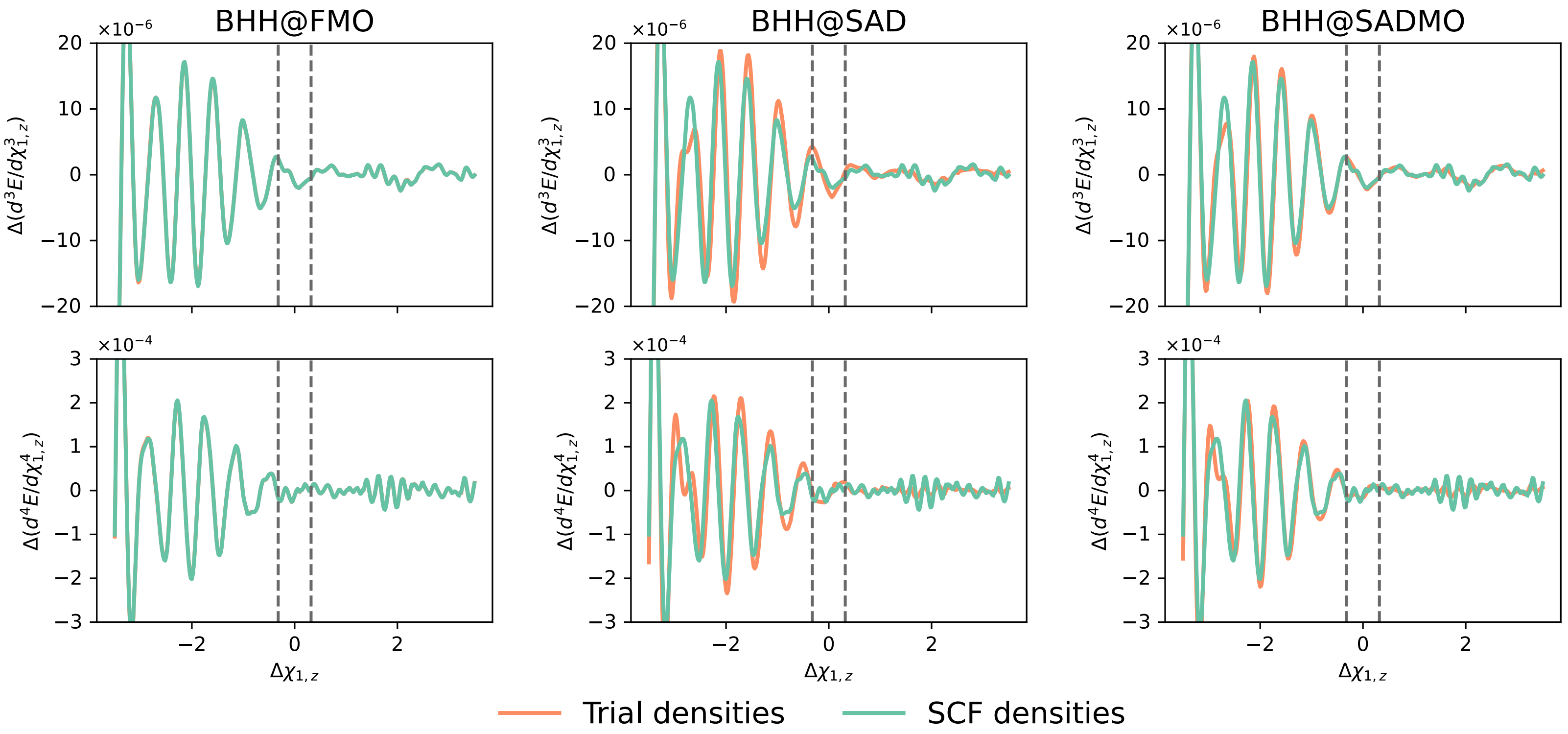

Figure S13: Spurious oscillations in $\mathrm{d}^3E/\mathrm{d}\chi_{1,z}{}^3$ and $\mathrm{d}^4E/\mathrm{d}\chi_{1,z}{}^4$ of the HCN · HF system, defined as $\Delta P = P^{\mathrm{DFA}(99,\ 590)} - P^{\mathrm{DFA}(1500,\ 974)}$, obtained with BH&H/6-31+G* and the trial densities (orange curves) and the SCF density (green curves, shown on each plot). Vertical dashed lines mark the displacement range of the property curve for which RMSE, and RRMSE are calculated.

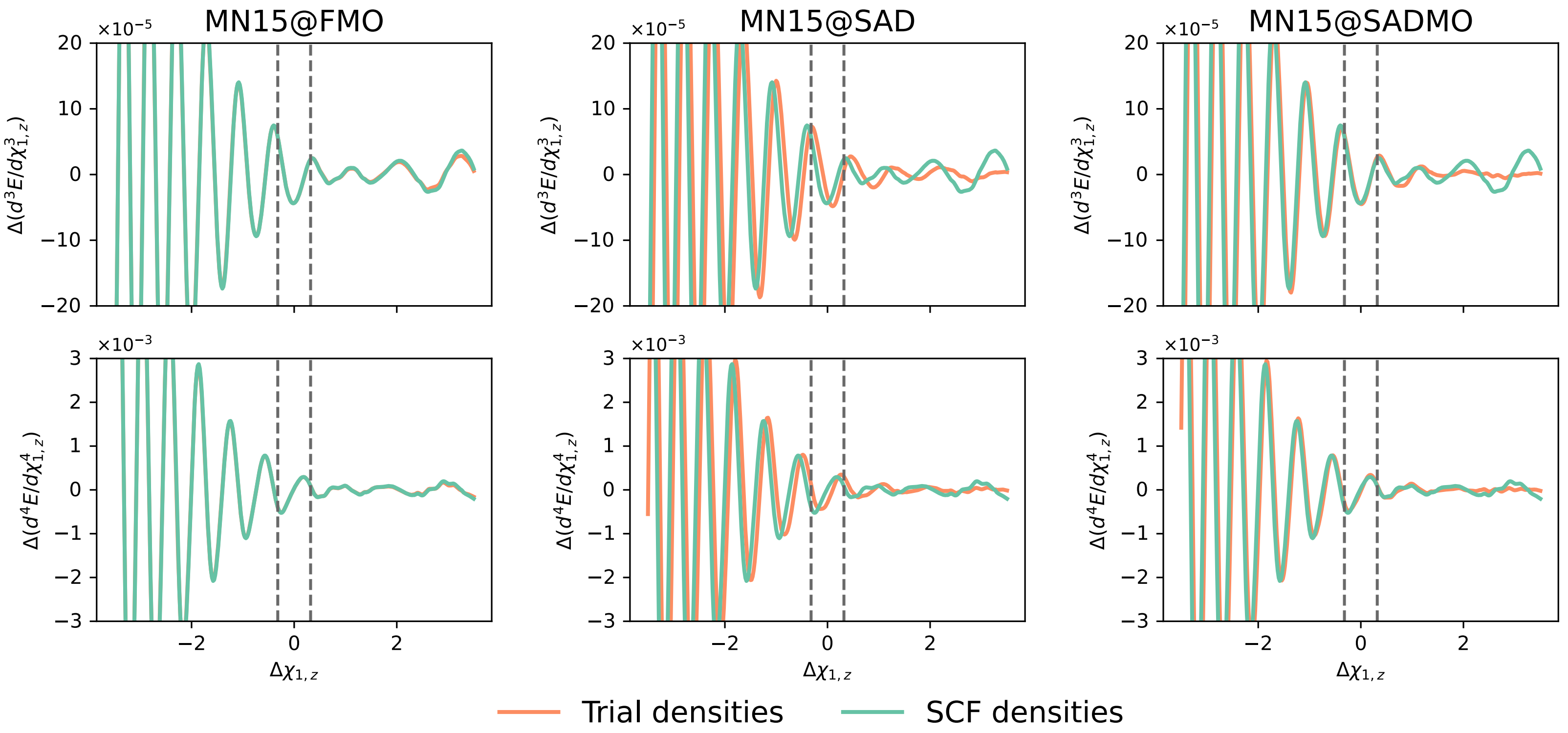

Figure S14: Spurious oscillations in $\mathrm{d}^3E/\mathrm{d}\chi_{1,z}{}^3$ and $\mathrm{d}^4E/\mathrm{d}\chi_{1,z}{}^4$ of the HCN · HF system, defined as $\Delta P = P^{\mathrm{DFA}(99,\ 590)} - P^{\mathrm{DFA}(1500,\ 974)}$, obtained with MN15/6-31+G* and the trial densities (orange curves) and the SCF density (green curves, shown on each plot). Vertical dashed lines mark the displacement range of the property curve for which RMSE, and RRMSE are calculated.

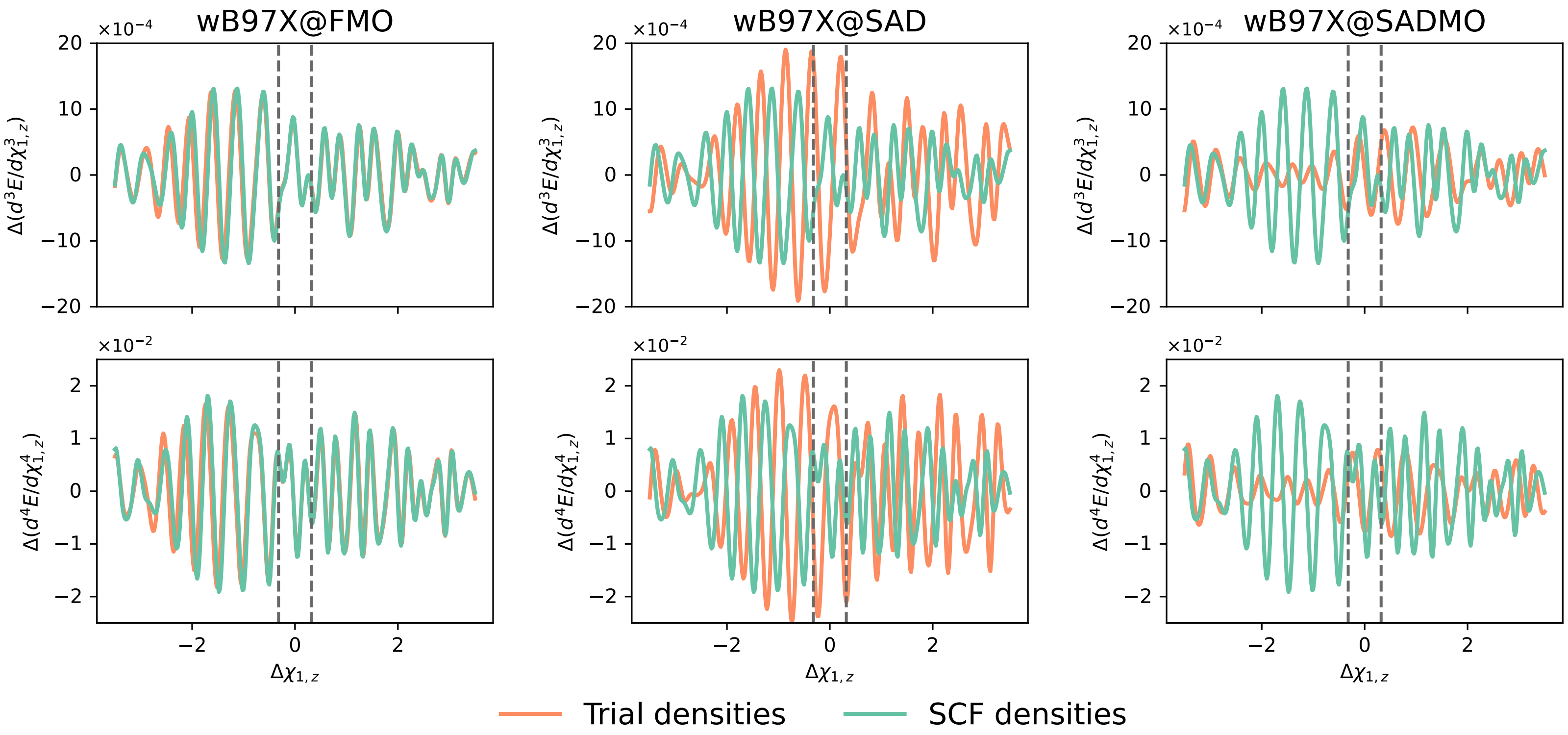

Figure S15: Spurious oscillations in $\mathrm{d}^3E/\mathrm{d}\chi_{1,z}{}^3$ and $\mathrm{d}^4E/\mathrm{d}\chi_{1,z}{}^4$ of the HCN · HF system, defined as $\Delta P = P^{\mathrm{DFA}(99,\ 590)} - P^{\mathrm{DFA}(1500,\ 974)}$, obtained with ωB97X/6-31+G* and the trial densities (orange curves) and the SCF density (green curves, shown on each plot). Vertical dashed lines mark the displacement range of the property curve for which RMSE, and RRMSE are calculated.

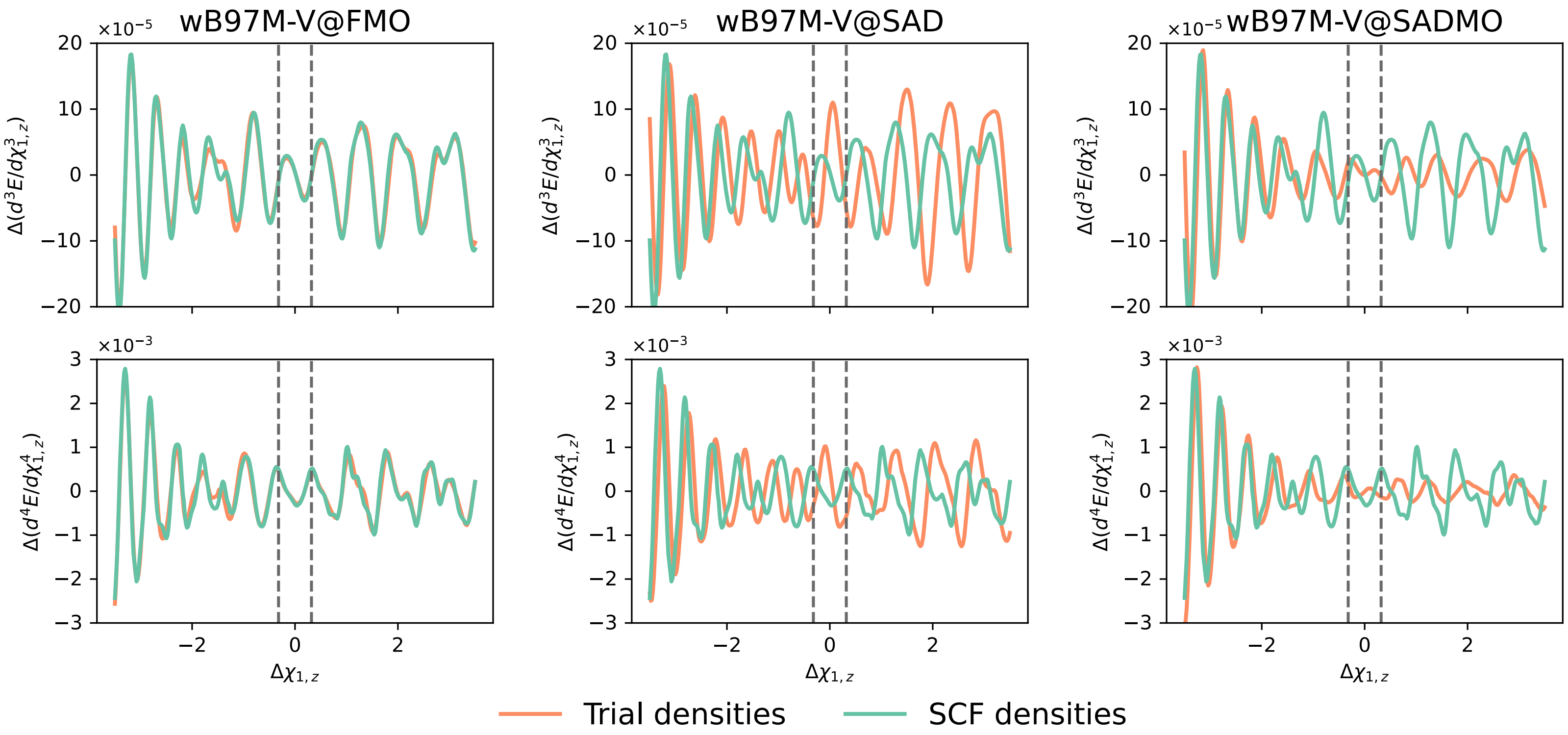

Figure S16: Spurious oscillations in $\mathrm{d}^3E/\mathrm{d}{\chi_{1,z}}^3$ and $\mathrm{d}^4E/\mathrm{d}{\chi_{1,z}}^4$ of the HCN · HF system, defined as $\Delta P = P^{\mathrm{DFA}(99,\,590)} - P^{\mathrm{DFA}(1500,\,974)}$, obtained with ωB97M-V/6-31+G* and the trial densities (orange curves) and the SCF density (green curves, shown on each plot). Vertical dashed lines mark the displacement range of the property curve for which RMSE, and RRMSE are calculated.

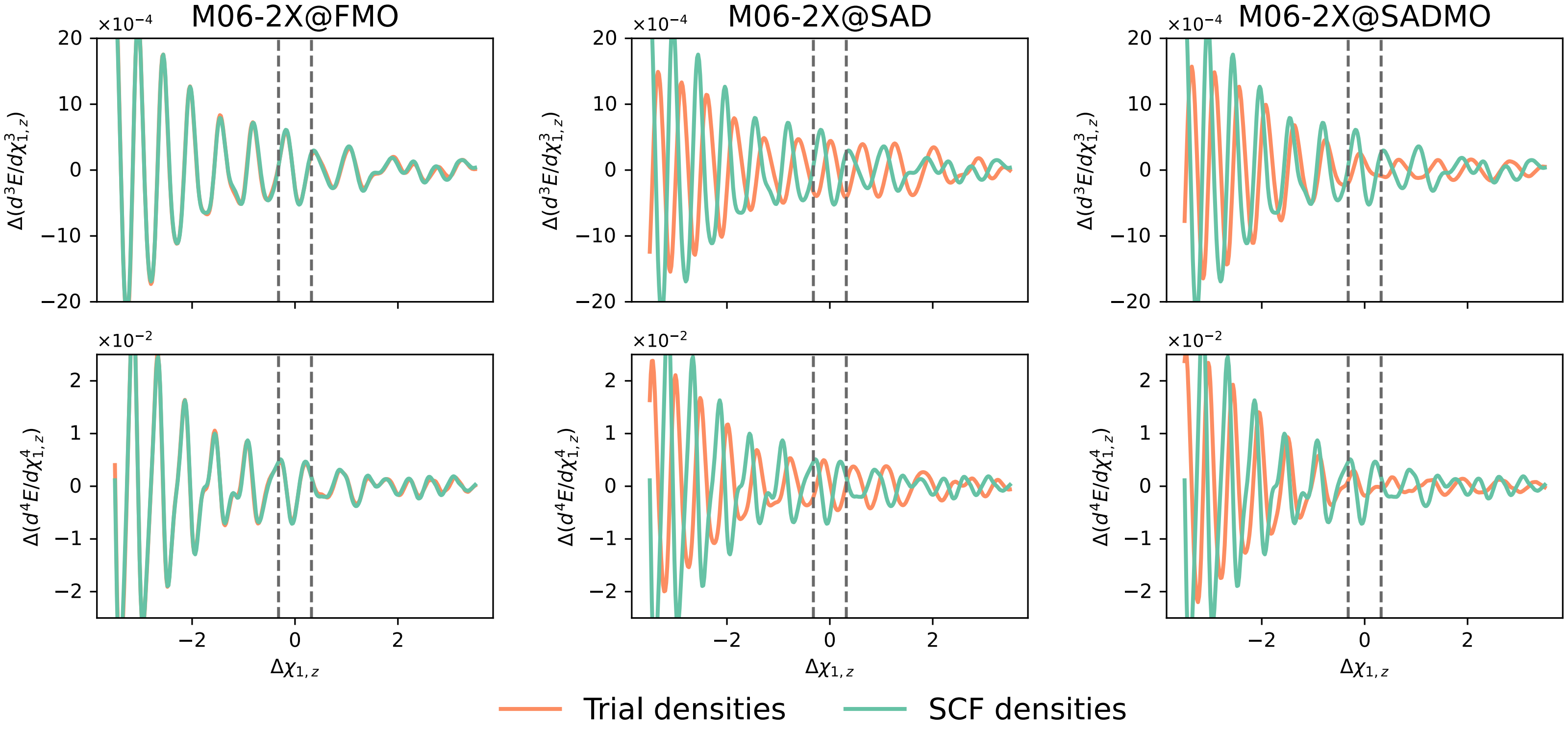

Figure S17: Spurious oscillations in $\mathrm{d}^3E/\mathrm{d}\chi_{1,z}{}^3$ and $\mathrm{d}^4E/\mathrm{d}\chi_{1,z}{}^4$ of the HCN · HF system, defined as $\Delta P = P^{\mathrm{DFA}(99,\ 590)} - P^{\mathrm{DFA}(1500,\ 974)}$, obtained with M06-2X/6-31+G* and the trial densities (orange curves) and the SCF density (green curves, shown on each plot). Vertical dashed lines mark the displacement range of the property curve for which RMSE, and RRMSE are calculated.

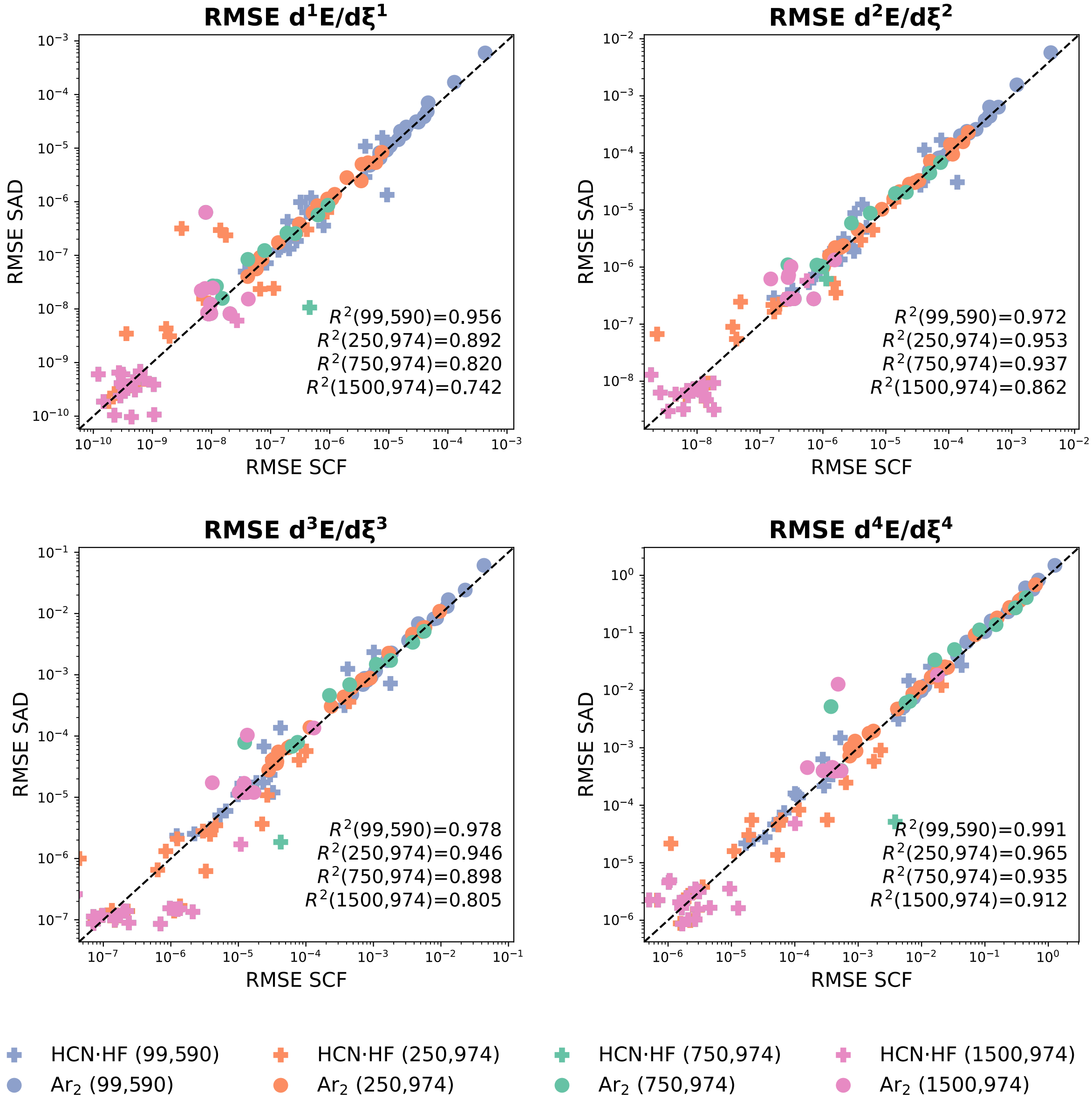

Figure S18: Correlation between RMSE in $d^mE/d\xi^m$ obtained using the SCF and SAD densities for 20 DFAs combined with the 6-31+G* basis set. Colors represent the integration grid used to calculate RMSE: (99, 590) — blue, (250, 974) — orange, (500, 974) — green, and (1500, 974) — pink. Point shapes represent different chemical systems: HCN · HF — plus, Ar_2 — circle. The line representing ideal correlation, i.e. $y = x$, is shown with a dashed line.

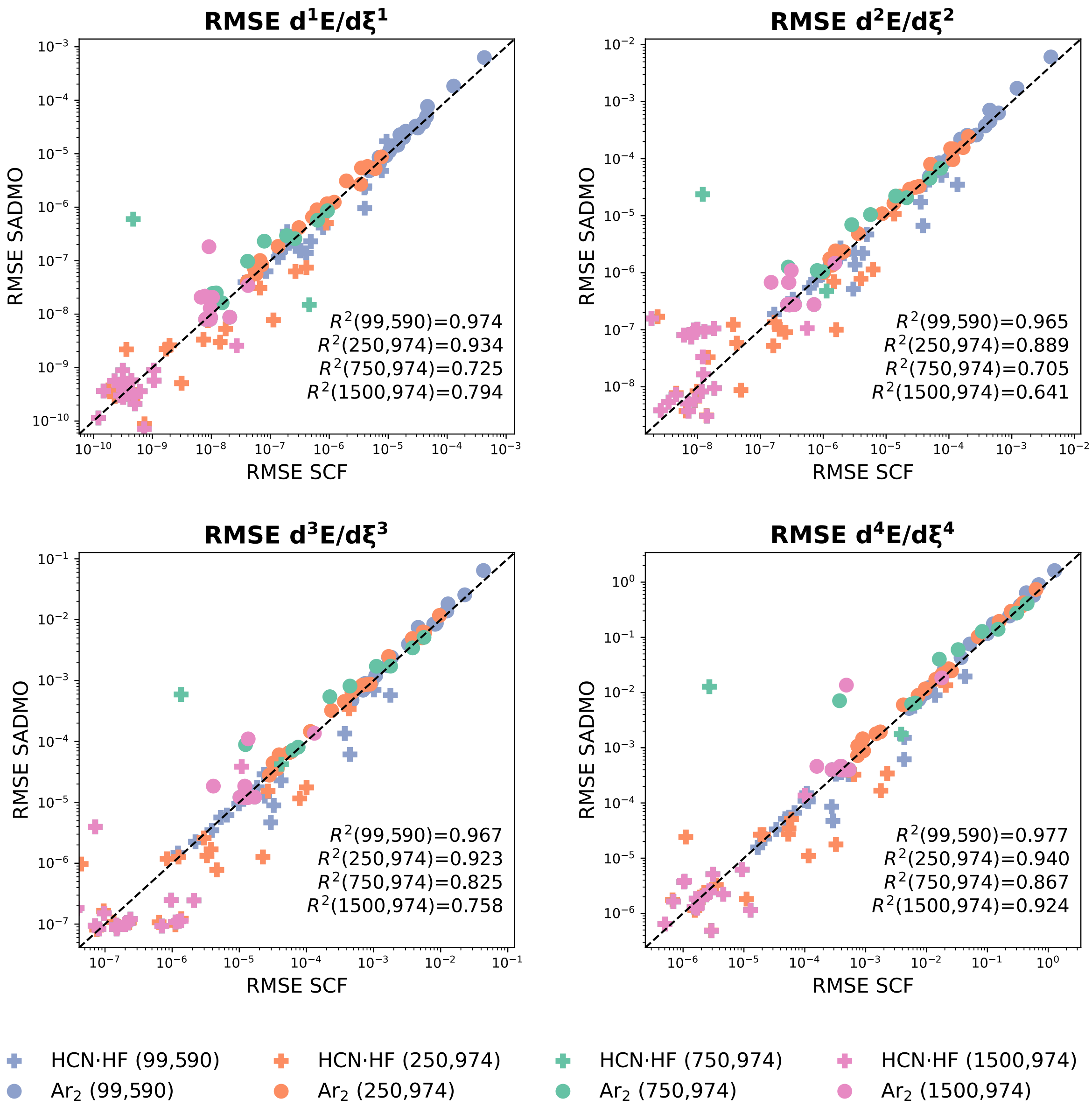

Figure S19: Correlation between RMSE in $\mathrm{d}^m E/\mathrm{d}\xi^m$ obtained using the SCF and SADMO densities for 20 DFAs combined with the 6-31+G* basis set. Colors represent the integration grid used to calculate RMSE: (99, 590) — blue, (250, 974) — orange, (500, 974) — green, and (1500, 974) — pink. Point shapes represent different chemical systems: HCN · HF — plus, Ar_2 — circle. The line representing ideal correlation, i.e. $y = x$, is shown with a dashed line.

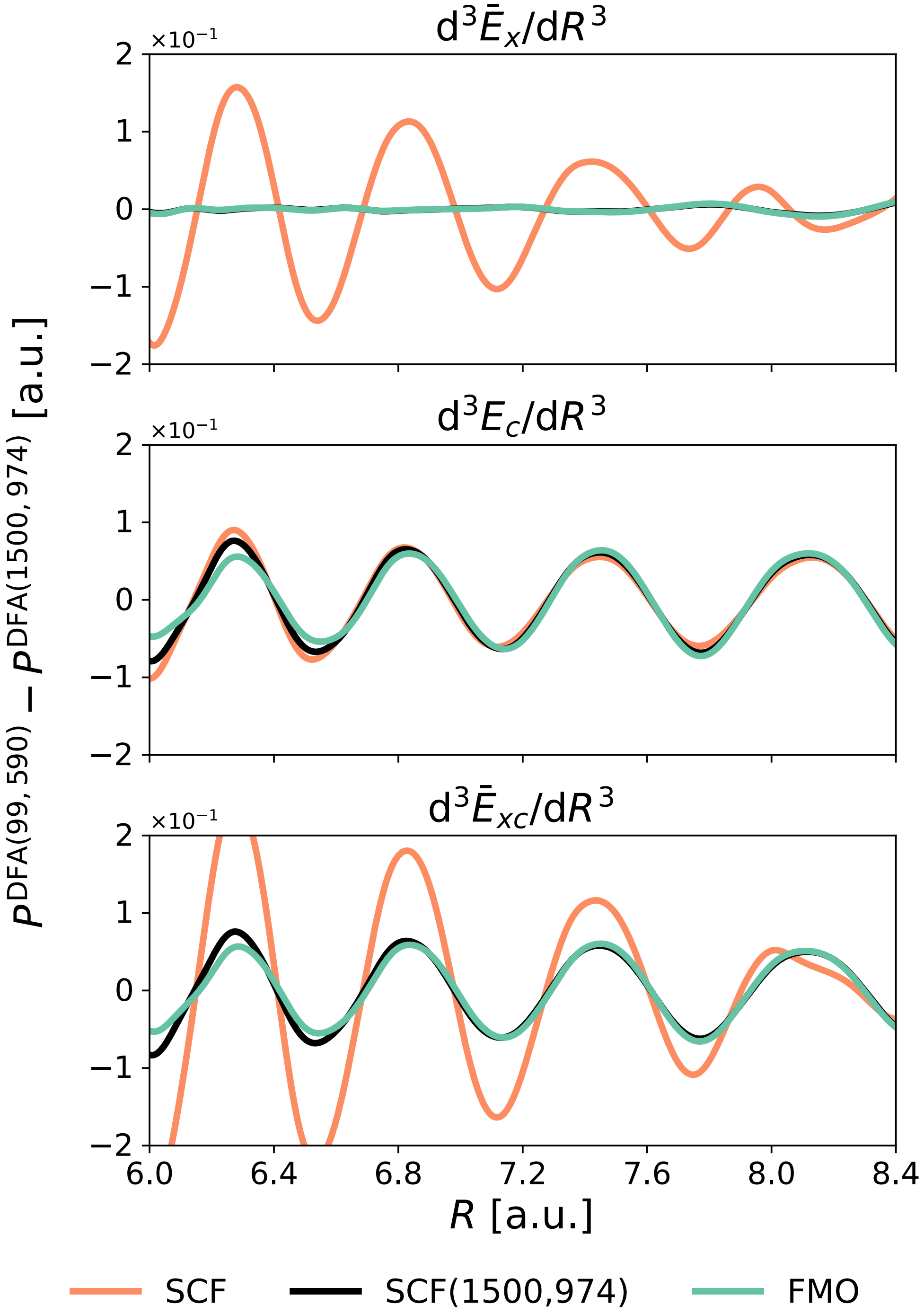

Figure S20: Spurious oscillations in the third derivatives of exchange (E_x), correlation (E_c) energy components of Ar_2, as well as their sum, obtained using VSXC/6-31+G* and the (99, 590) and (1500, 974) integration grids. Three different types of the input densities are employed: SCF — density obtained in SCF using corresponding grids, SCF(1500, 974) — density obtained in SCF using the reference (1500, 974) grid, FMO — trial density constructed using FMOs obtained for one geometry (R=7.2 Bohr) and the (250, 974) integration grid.

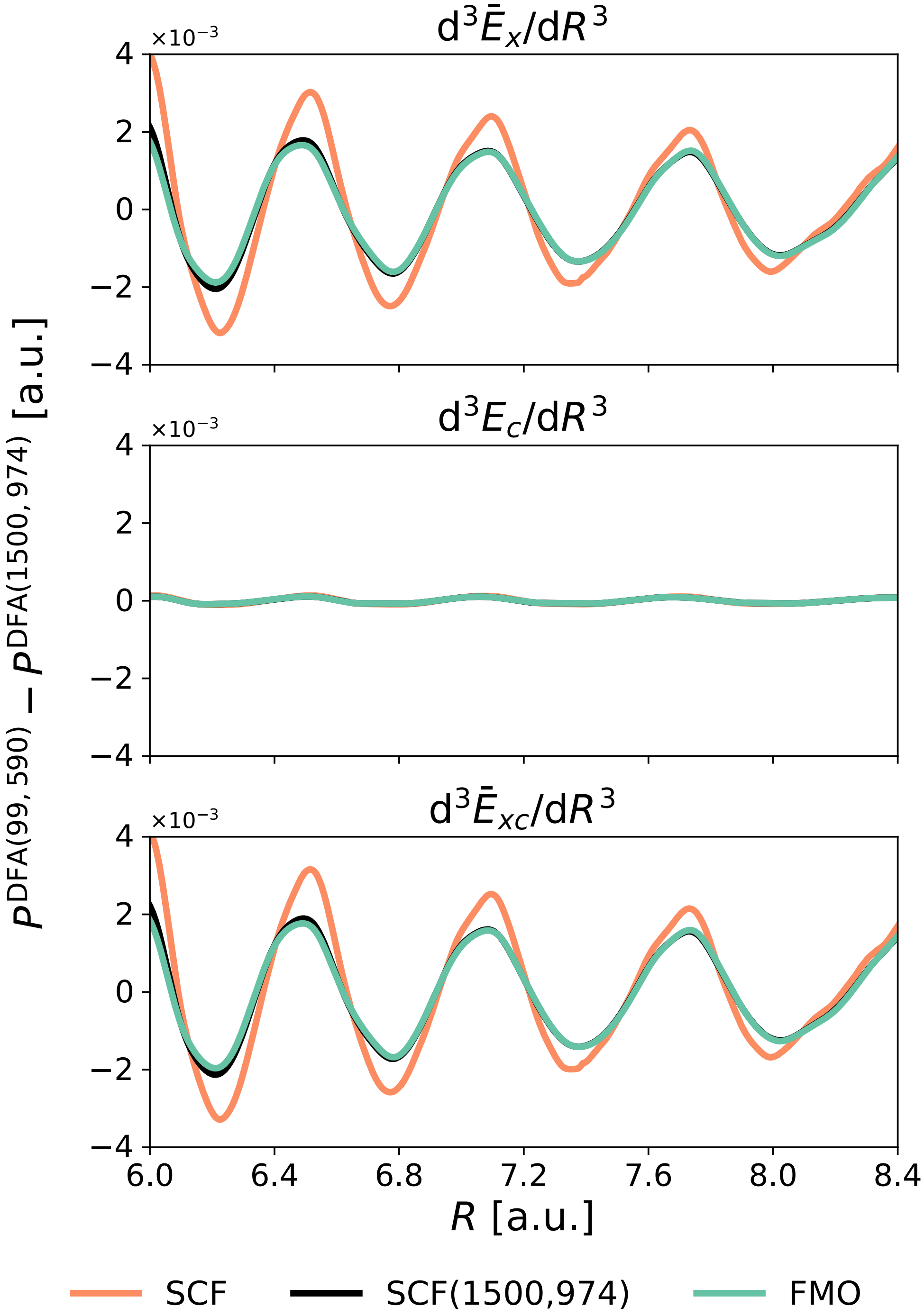

Figure S21: Spurious oscillations in the third derivatives of exchange (E_x), correlation (E_c) energy components of Ar_2, as well as their sum, obtained using B3LYP/6-31+G* and the (99, 590) and (1500, 974) integration grids. Three different types of the input densities are employed: SCF — density obtained in SCF using corresponding grids, SCF(1500, 974) — density obtained in SCF using the reference (1500, 974) grid, FMO — trial density constructed using FMOs obtained for one geometry (R=7.2 Bohr) and the (250, 974) integration grid.

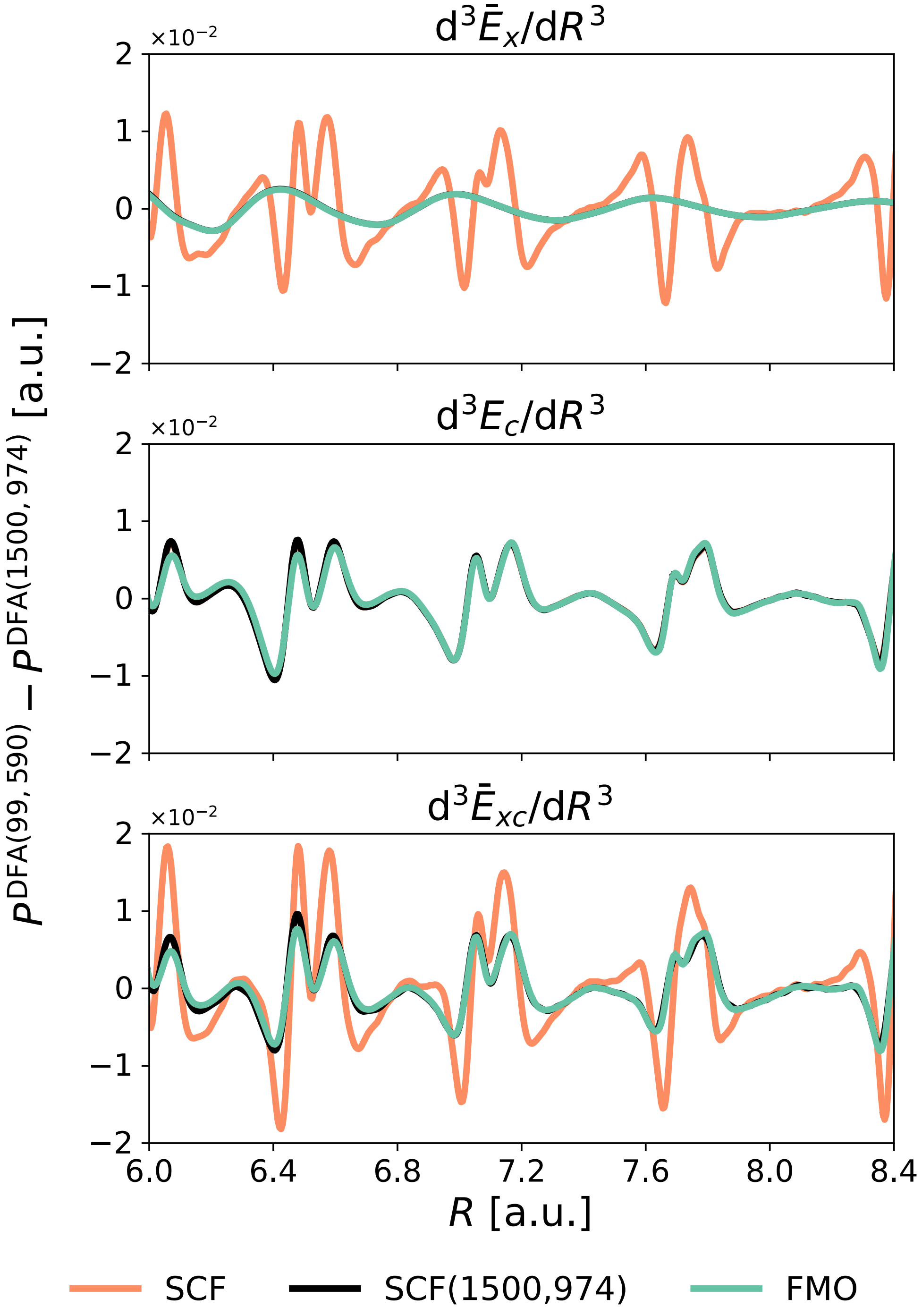

Figure S22: Spurious oscillations in the third derivatives of exchange (E_x), correlation (E_c) energy components of Ar_2, as well as their sum, obtained using ωB97M-V/6-31+G* and the (99, 590) and (1500, 974) integration grids. Three different types of the input densities are employed: SCF — density obtained in SCF using corresponding grids, SCF(1500, 974) — density obtained in SCF using the reference (1500, 974) grid, FMO — trial density constructed using FMOs obtained for one geometry (R=7.2 Bohr) and the (250, 974) integration grid.

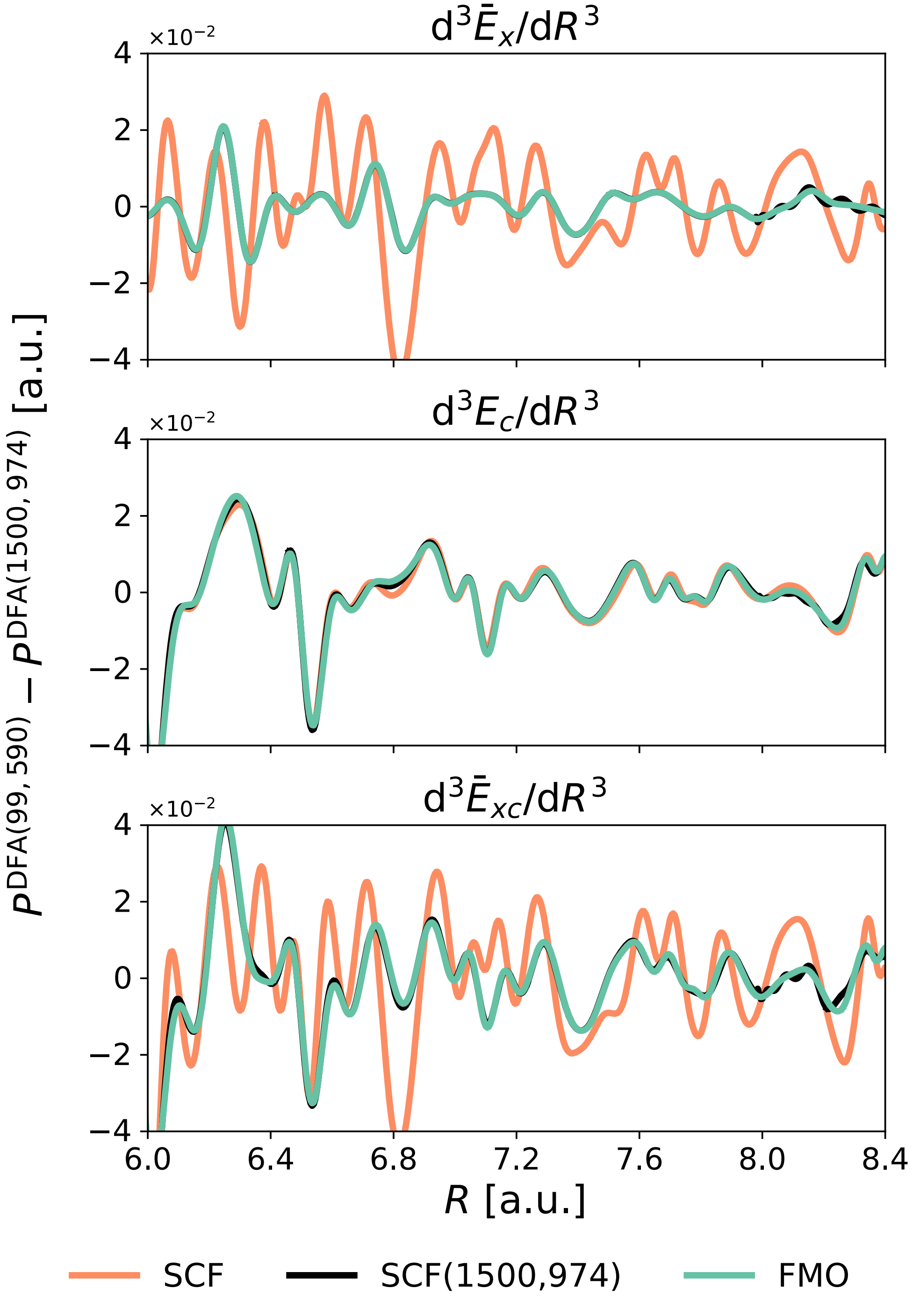

Figure S23: Spurious oscillations in the third derivatives of exchange (E_x), correlation (E_c) energy components of Ar_2, as well as their sum, obtained using M06-2X/6-31+G* and the (99, 590) and (1500, 974) integration grids. Three different types of the input densities are employed: SCF — density obtained in SCF using corresponding grids, SCF(1500, 974) — density obtained in SCF using the reference (1500, 974) grid, FMO — trial density constructed using FMOs obtained for one geometry (R=7.2 Bohr) and the (250, 974) integration grid.

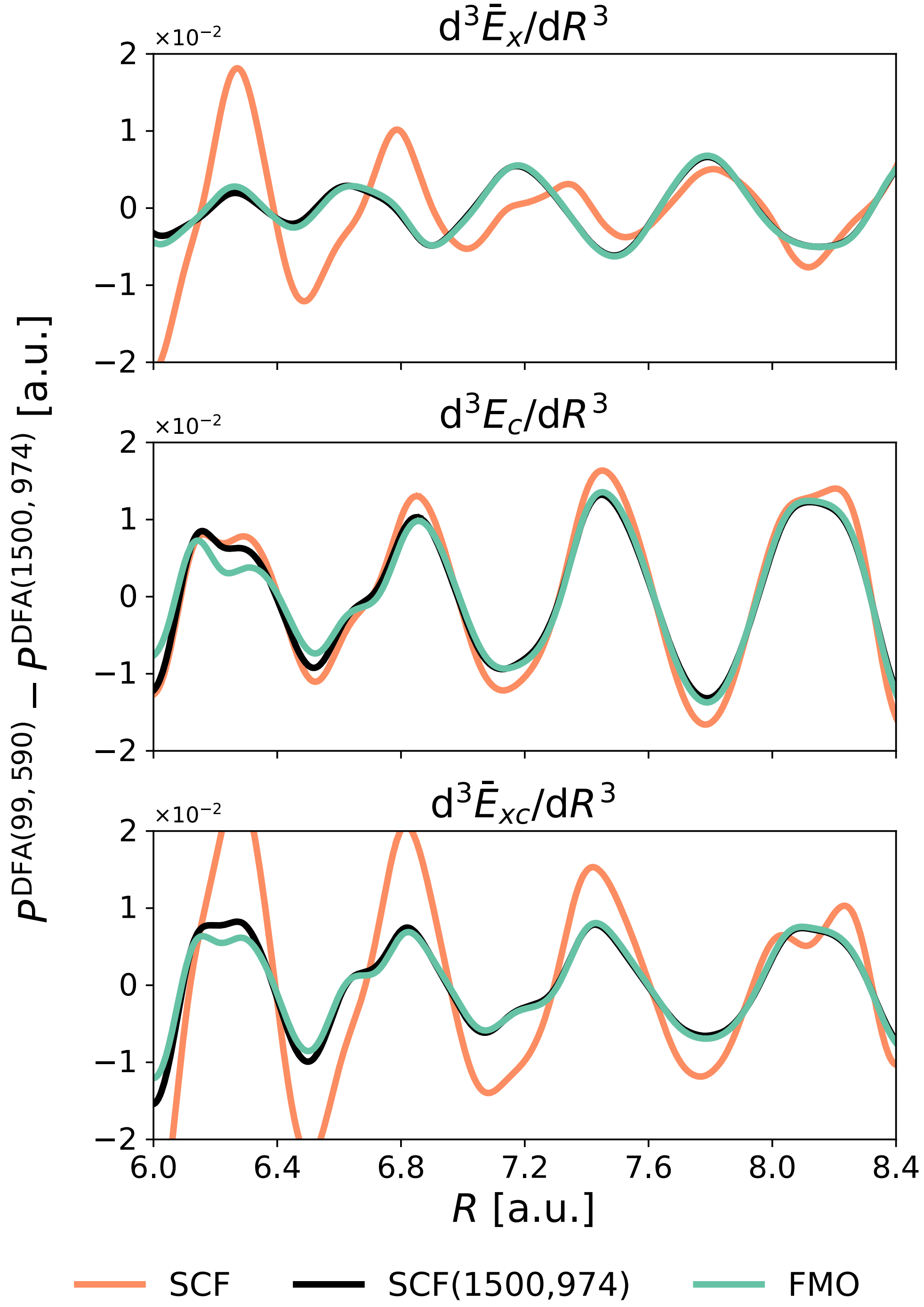

Figure S24: Spurious oscillations in the third derivatives of exchange (E_x), correlation (E_c) energy components of Ar_2, as well as their sum, obtained using MN15/6-31+G* and the (99, 590) and (1500, 974) integration grids. Three different types of input densities are employed: SCF — density obtained in SCF using corresponding grids, SCF(1500, 974) — density obtained in SCF using the reference (1500, 974) grid, FMO — trial density constructed using FMOs obtained for one geometry (R=7.2 Bohr) and the (250, 974) integration grid.

S3 Supplementary Tables

Table S1: List of DFAs used in this work; GGA — Generalized Gradient Approximation, GH — global hybrids, RSH — range-separated hybrids.

DFA	Type	Ref.	DFA	Type	Ref.
BLYP	GGA	1,2	PBE	GGA	3
B1LYP	GH GGA	4	PBE0	GH GGA	5
B3LYP	GH GGA	6,7	PBE50	GH GGA	8
BH&H	GH GGA[a]	9	LC-ωPBE	RSH GGA	10
BH&HLYP	GH GGA	9	VSXC	meta-GGA	11
LC-BLYP	RSH GGA	12	B97	GH GGA	13
CAM-B3LYP	RSH GGA	14	B97-D	GGA	15
M06	GH meta-GGA	16	ωB97	RSH GGA	17
M06-L	meta-GGA	18	ωB97X	RSH GGA	17
M06-HF	GH meta-GGA	19	ωB97X-D	RSH GGA	20
M06-2X	GH meta-GGA	16	ωB97X-D3	RSH GGA	21
SOGGA11	GGA	22	B97M-V	meta-GGA	23
SOGGA11-X	GH GGA	24	ωB97M-V	RSH meta-GGA	25
M11	RSH meta-GGA	26	ωB97X-V	RSH GGA	27
M11-L	meta-GGA	28	TPSS	meta-GGA	29
MN12-L	meta-GGA	30	RevTPSS	meta-GGA	31
N12-SX	RSH GGA	32	TPSSh	GH meta-GGA	33
N12	GGA	34	SPW92	LDA	35,36
MN12-SX	RSH meta-GGA	32	SVWN5	LDA	35,37
MN15	GH meta-GGA	38	PW91	GGA	39
MN15-L	meta-GGA	40	mPW91	GGA	41
SCAN	meta-GGA	42	B1PW91	GH GGA	4
SCAN0	GH meta-GGA	43			

[a] No GGA exchange is used, *i.e.*, $E_{xc}^{BHH} = 0.5\, E_x^{Slater} + 0.5\, E_x^{HF} + E_c^{LYP}$

Table S2: DFAs included in this work grouped by families. For the sake of simplicity, in the manuscript, we also refer to the B97 family, which comprises families B97st, B97X, and B97M.

Family	DFAs
BH&H	BH&H
LSDA	SVWN5, SPW92
BLYP	BLYP, B1LYP, B3LYP, BH&HLYP, LC-BLYP, CAM-B3LYP
PBE	PBE, PBE0, PBE50, LC-ωPBE
TPSS	TPSS, RevTPSS, TPSSh
PW91	PW91, mPW91, B1PW91
M11	M11, M11-L
MN12	MN12-L, MN12-SX
B97st	B97, B97-D, ωB97
B97X	ωB97X, ωB97X-D, ωB97X-D3, ωB97X-V
B97M	B97M-V, ωB97M-V
SCAN	SCAN, SCAN0
M06	M06, M06-L, M06-2X, M06-HF
VSXC	VSXC
MN15	MN15, MN15-L
SOGGA11	SOGGA11, SOGGA11-X
N12	N12, N12-SX

Table S3: RMSE values for various property derivatives obtained with different basis sets and ωB97X combined with the (99, 590) integration grid for the HCN $\cdot$ HF system. For each column, the values in cells have to be multiplied with the exponent given in top.

	$\mathrm{d}^m E/\mathrm{d}\chi_{1,z}{}^m$				$\mathrm{d}^m \mu_z/\mathrm{d}\chi_{1,z}{}^m$				$\mathrm{d}^m \alpha_{zz}/\mathrm{d}\chi_{1,z}{}^m$		
	1	2	3	4	0	1	2	3	0	1	2
Basis set	[$\times 10^{-6}$]	[$\times 10^{-5}$]	[$\times 10^{-4}$]	[$\times 10^{-3}$]	[$\times 10^{-5}$]	[$\times 10^{-4}$]	[$\times 10^{-3}$]	[$\times 10^{-2}$]	[$\times 10^{-3}$]	[$\times 10^{-2}$]	[$\times 10^{-1}$]
STO-3G	5.7	5.9	8.8	15.8	1.5	1.6	2.4	4.6	0.3	0.6	1.3
3-21G	4.5	4.0	4.5	5.2	2.9	3.1	3.2	3.6	2.1	2.3	2.9
3-21+G	4.3	4.1	4.6	5.6	2.7	3.0	3.2	3.7	2.0	2.3	3.1
3-21++G	4.4	4.1	4.7	5.6	2.7	3.0	3.2	3.7	2.0	2.3	3.1
6-31G	4.4	4.6	5.9	9.5	1.7	2.1	3.1	5.4	1.3	2.2	3.9
6-31G*	4.0	4.3	4.3	6.3	2.2	2.6	2.8	4.6	1.1	1.3	2.4
6-31G**	4.6	4.8	4.7	6.5	2.8	3.4	3.3	4.6	2.3	2.3	3.4
6-31+G	4.4	4.7	6.3	10.5	1.7	2.1	3.4	6.0	1.3	2.3	4.4
6-31++G	4.4	4.7	6.3	10.5	1.7	2.1	3.3	5.9	1.3	2.3	4.5
6-31+G*	4.0	4.1	4.2	6.3	2.2	2.4	2.8	4.9	1.0	1.3	2.6
6-31++G**	4.4	4.4	4.8	5.4	3.0	3.0	3.5	4.2	2.1	2.4	2.7
6-311G	4.2	3.9	4.9	7.9	2.2	2.3	2.9	4.8	1.8	2.6	4.7
6-311G*	3.7	3.8	4.1	6.7	2.3	2.6	2.7	4.4	1.7	2.1	3.9
6-311G**	3.9	4.1	4.0	6.0	2.5	2.8	3.0	4.5	1.9	1.9	2.9
6-311+G	3.9	4.1	4.7	8.5	2.1	2.4	2.7	5.2	1.5	2.1	4.5
6-311++G	3.9	4.1	4.7	8.5	2.1	2.4	2.7	5.2	1.5	2.1	4.5
6-311+G*	3.5	3.8	3.5	5.6	2.3	2.6	2.9	5.1	1.3	1.5	2.9
6-311++G**	3.9	4.0	4.2	5.2	2.4	2.7	3.2	4.4	1.8	2.0	2.6
Def2SVP	4.3	4.5	4.7	6.8	2.4	2.8	3.3	5.1	1.7	1.9	2.8
Def2TZVP	4.0	4.2	4.3	5.3	2.6	3.0	3.3	4.2	2.2	2.3	2.7
ma-Def2SVP	4.2	4.1	5.0	6.2	2.3	2.4	3.2	4.3	1.6	2.0	3.0
ma-Def2TZVP	4.0	4.1	4.3	5.1	2.7	3.0	3.3	4.1	2.2	2.4	2.8
cc-pVTZ	4.4	4.6	4.9	7.9	2.6	2.9	3.0	4.6	1.9	2.1	3.9
cc-pVDZ	4.2	4.4	5.2	6.0	3.0	3.3	3.9	4.5	2.6	3.1	3.5
aug-cc-pVDZ	3.8	3.8	4.2	6.1	2.2	2.2	2.8	4.1	1.7	2.0	3.6
aug-cc-pVTZ	4.0	4.1	5.2	5.6	2.9	3.0	3.8	3.9	2.3	3.0	3.1

Table S4: RMSE values for various property derivatives obtained with different basis sets and M06-2X combined with the (99, 590) integration grid for the HCN·HF system. For each column, the values in cells have to be multiplied with the exponent given in top.

	$\mathrm{d}^m E/\mathrm{d}\chi_{1,z}{}^m$				$\mathrm{d}^m \mu_z/\mathrm{d}\chi_{1,z}{}^m$				$\mathrm{d}^m \alpha_{zz}/\mathrm{d}\chi_{1,z}{}^m$		
	1	2	3	4	0	1	2	3	0	1	2
Basis set	$[\times 10^{-6}]$	$[\times 10^{-5}]$	$[\times 10^{-4}]$	$[\times 10^{-3}]$	$[\times 10^{-5}]$	$[\times 10^{-4}]$	$[\times 10^{-3}]$	$[\times 10^{-2}]$	$[\times 10^{-3}]$	$[\times 10^{-2}]$	$[\times 10^{-1}]$
STO-3G	4.7	4.0	4.3	4.3	1.1	1.2	1.5	1.6	0.5	0.5	0.6
3-21G	1.9	2.0	2.8	4.4	1.7	1.5	1.4	1.4	1.4	1.1	1.2
3-21+G	2.2	1.9	2.6	4.1	1.7	1.5	1.5	1.5	1.5	1.3	1.3
3-21++G	2.1	1.9	2.6	4.1	1.7	1.5	1.5	1.5	1.5	1.3	1.3
6-31G	2.4	1.6	2.2	2.3	3.0	2.2	2.5	2.4	1.3	1.4	1.4
6-31G*	3.3	2.8	3.1	3.6	2.2	1.7	1.9	2.0	1.5	1.5	1.6
6-31G**	2.8	2.2	2.1	2.2	2.1	1.7	1.7	1.6	1.2	1.3	1.4
6-31+G	2.7	1.8	2.5	2.6	3.1	2.3	2.7	2.6	1.4	1.6	1.5
6-31++G	2.7	1.8	2.5	2.6	3.1	2.3	2.7	2.6	1.4	1.6	1.5
6-31+G*	3.9	3.6	3.7	4.3	2.2	1.7	1.8	2.0	1.7	1.6	1.8
6-31++G**	2.0	1.6	1.3	1.6	1.7	1.4	1.2	1.4	1.5	1.4	1.6
6-311G	1.4	1.3	1.8	1.8	2.8	2.4	2.6	2.5	1.8	1.7	1.7
6-311G*	2.3	2.1	2.3	2.4	2.4	2.1	2.2	2.2	1.9	1.7	1.8
6-311G**	2.2	2.0	1.9	2.0	1.8	1.8	1.7	1.8	1.5	1.5	1.7
6-311+G	2.3	2.2	2.5	2.6	2.5	2.3	2.5	2.5	1.9	1.8	1.9
6-311++G	2.3	2.2	2.5	2.6	2.5	2.3	2.5	2.5	1.9	1.8	1.9
6-311+G*	3.2	3.0	3.1	3.5	1.9	1.9	1.9	2.0	2.0	1.8	2.0
6-311++G**	1.7	1.7	1.5	1.7	1.4	1.6	1.4	1.6	1.7	1.5	1.8
Def2SVP	3.3	2.6	2.4	2.3	2.4	2.0	1.8	1.9	1.5	1.5	1.7
Def2TZVP	1.6	1.4	1.1	1.4	1.6	1.3	1.1	1.3	1.4	1.3	1.5
ma-Def2SVP	2.8	2.2	1.9	2.1	1.9	1.7	1.5	1.8	1.6	1.5	1.8
ma-Def2TZVP	1.5	1.3	0.9	1.4	1.5	1.3	1.0	1.3	1.4	1.3	1.5
cc-pVTZ	3.6	2.8	2.8	3.0	2.8	2.2	2.2	2.1	1.4	1.5	1.6
cc-pVDZ	1.2	1.0	0.7	1.2	0.9	1.1	0.9	1.2	1.2	1.2	1.4
aug-cc-pVDZ	1.7	1.4	1.3	1.8	2.1	1.6	1.4	1.6	1.9	1.8	1.9
aug-cc-pVTZ	0.9	0.8	0.7	1.3	0.7	0.9	0.8	1.0	1.0	1.1	1.1

Table S5: RMSE values for various property derivatives obtained with different basis sets and B3LYP combined with the (99, 590) integration grid for the HCN $\cdot$ HF system. For each column, the values in cells have to be multiplied with the exponent given in top.

	$\mathrm{d}^m E/\mathrm{d}\chi_{1,z}{}^m$				$\mathrm{d}^m \mu_z/\mathrm{d}\chi_{1,z}{}^m$				$\mathrm{d}^m \alpha_{zz}/\mathrm{d}\chi_{1,z}{}^m$		
	1	2	3	4	0	1	2	3	0	1	2
Basis set	$[\times 10^{-7}]$	$[\times 10^{-7}]$	$[\times 10^{-6}]$	$[\times 10^{-5}]$	$[\times 10^{-7}]$	$[\times 10^{-6}]$	$[\times 10^{-5}]$	$[\times 10^{-4}]$	$[\times 10^{-6}]$	$[\times 10^{-5}]$	$[\times 10^{-4}]$
STO-3G	1.4	10.5	7.2	7.4	1.5	1.1	3.2	0.8	5.9	3.8	3.4
3-21G	1.6	9.4	8.5	6.0	3.2	1.6	1.0	1.0	6.5	5.6	2.7
3-21+G	1.6	9.4	8.5	6.1	3.0	1.6	1.0	1.0	8.1	6.1	3.2
3-21++G	1.6	9.4	8.5	6.1	3.0	1.6	1.0	1.0	7.5	6.1	3.3
6-31G	1.3	6.2	6.0	5.6	3.0	2.6	1.7	1.5	7.5	6.2	6.3
6-31G*	1.3	7.2	6.6	5.5	2.2	1.6	1.0	1.0	8.8	8.5	6.6
6-31G**	1.2	8.1	6.1	5.2	1.6	1.1	0.9	0.6	6.6	4.4	2.5
6-31+G	1.4	6.3	6.3	5.7	3.3	2.6	1.8	1.6	9.3	7.6	7.0
6-31++G	1.4	6.3	6.3	5.6	3.3	2.6	1.8	1.6	9.3	7.8	7.1
6-31+G*	1.4	7.6	6.5	5.5	2.2	1.4	0.9	1.0	10.9	10.3	8.1
6-31++G**	1.2	8.3	6.2	5.5	1.5	1.2	0.9	0.7	9.4	5.0	4.5
6-311G	0.8	5.7	3.3	3.4	3.7	2.8	1.8	1.7	8.6	9.7	6.5
6-311G*	3.5	5.8	2.9	3.0	3.4	2.4	1.5	1.5	10.8	10.1	7.2
6-311G**	0.8	6.6	3.5	3.5	2.3	1.7	1.2	1.0	10.0	5.1	4.0
6-311+G	0.8	5.9	3.2	3.2	3.5	2.6	1.7	1.6	11.2	11.0	8.8
6-311++G	0.8	5.9	3.2	3.2	3.5	2.6	1.7	1.6	11.2	11.0	8.8
6-311+G*	3.4	6.2	2.9	2.9	2.9	1.9	1.2	1.2	12.8	12.4	8.9
6-311++G**	0.8	6.9	3.8	3.7	1.9	1.6	1.0	0.9	10.6	6.7	6.3
Def2SVP	1.0	9.7	5.0	4.6	2.5	1.8	1.4	1.1	10.2	5.6	4.8
Def2TZVP	2.1	3.7	3.0	2.3	1.3	1.2	0.9	0.7	8.6	4.5	4.2
ma-Def2SVP	1.0	9.0	5.4	5.2	2.0	1.7	1.1	0.9	12.1	7.7	6.8
ma-Def2TZVP	2.1	3.8	3.0	2.4	1.2	1.2	0.8	0.6	8.8	4.9	4.7
cc-pVTZ	1.5	8.0	6.0	4.1	2.4	1.8	1.3	1.0	9.1	5.2	2.8
cc-pVDZ	1.2	8.2	5.8	5.2	1.2	1.0	0.8	0.5	9.6	3.9	3.6
aug-cc-pVDZ	1.4	7.9	5.7	4.5	2.4	1.8	1.0	0.8	15.7	10.7	9.2
aug-cc-pVTZ	3.1	9.1	6.0	5.3	0.6	0.7	0.7	0.5	43.2	37.1	84.8

Table S6: RMSE values for various property derivatives obtained with different basis sets and ωB97X combined with the (250, 974) integration grid for the HCN$\cdot$HF system. For each column, the values in cells have to be multiplied with the exponent given in top.

	$\mathrm{d}^m E/\mathrm{d}\chi_{1,z}{}^m$				$\mathrm{d}^m \mu_z/\mathrm{d}\chi_{1,z}{}^m$				$\mathrm{d}^m \alpha_{zz}/\mathrm{d}\chi_{1,z}{}^m$		
	1	2	3	4	0	1	2	3	0	1	2
Basis set	[$\times 10^{-7}$]	[$\times 10^{-6}$]	[$\times 10^{-5}$]	[$\times 10^{-3}$]	[$\times 10^{-6}$]	[$\times 10^{-5}$]	[$\times 10^{-4}$]	[$\times 10^{-3}$]	[$\times 10^{-4}$]	[$\times 10^{-3}$]	[$\times 10^{-2}$]
STO-3G	3.9	6.8	15.3	4.6	1.3	2.2	5.2	14.7	0.8	1.5	4.5
3-21G	3.9	4.6	7.8	1.2	1.8	2.6	3.9	6.7	2.0	3.5	5.5
3-21+G	2.8	4.3	7.2	1.2	1.8	2.7	3.9	7.3	2.1	3.5	5.9
3-21++G	2.8	4.3	7.1	1.2	1.8	2.7	3.9	7.3	2.1	3.5	5.9
6-31G	3.2	5.3	12.0	3.0	2.2	3.9	7.6	19.1	2.2	5.0	12.9
6-31G*	3.0	4.6	8.6	1.6	1.7	2.7	4.6	10.3	1.8	3.7	7.6
6-31G**	2.3	3.7	5.6	1.1	1.5	2.2	3.4	5.7	1.8	2.7	5.6
6-31+G	3.2	5.6	13.3	3.5	2.2	4.1	8.4	22.6	2.4	5.7	15.2
6-31++G	3.2	5.6	13.2	3.5	2.1	4.1	8.3	22.6	2.4	5.6	15.1
6-31+G*	2.7	4.0	8.0	1.8	1.8	3.0	5.4	12.7	1.6	3.4	8.2
6-31++G**	2.1	3.2	4.9	0.8	10.7	2.5	3.6	6.6	1.3	2.1	4.1
6-311G	3.3	5.4	10.7	2.4	2.2	4.0	6.9	18.0	2.8	5.8	13.9
6-311G*	4.7	5.1	9.7	1.7	1.9	3.2	5.8	13.5	2.6	5.5	10.3
6-311G**	2.3	3.9	6.2	1.2	1.4	2.2	4.0	9.1	1.7	2.9	6.3
6-311+G	3.3	5.5	11.3	2.4	1.8	3.5	7.1	18.7	2.8	6.1	13.3
6-311++G	3.3	5.5	11.3	2.4	1.9	3.5	7.1	18.8	2.8	6.1	13.3
6-311+G*	4.2	4.5	8.9	1.8	1.9	3.3	5.9	13.2	2.1	4.6	10.2
6-311++G**	2.1	3.2	5.3	1.0	1.4	2.4	4.2	8.7	1.3	2.6	5.7
Def2SVP	2.4	4.0	6.6	1.4	1.5	2.5	4.8	11.2	1.6	3.0	6.6
Def2TZVP	1.9	2.8	4.3	0.7	1.2	1.9	3.0	5.6	1.3	2.2	3.5
ma-Def2SVP	2.2	3.3	6.5	1.4	1.4	2.4	4.3	9.5	1.3	3.0	7.3
ma-Def2TZVP	1.8	2.7	4.1	0.6	1.3	1.9	3.0	5.6	1.3	2.1	3.3
cc-pVTZ	2.7	4.6	8.0	1.8	1.7	2.5	4.4	9.4	2.2	4.1	9.8
cc-pVDZ	1.5	2.1	3.0	0.4	1.1	1.6	2.3	3.7	1.2	1.7	2.5
aug-cc-pVDZ	2.4	4.0	7.8	1.7	1.4	2.8	4.7	11.6	1.8	3.9	9.2
aug-cc-pVTZ	3.5	2.0	2.7	0.4	1.0	1.5	2.2	3.4	1.3	1.6	2.3

Table S7: RMSE values for various property derivatives obtained with different basis sets and M06-2X combined with the (250, 974) integration grid for the HCN · HF system. For each column, the values in cells have to be multiplied with the exponent given in top.

	$\mathrm{d}^m E/\mathrm{d}\chi_{1,z}{}^m$				$\mathrm{d}^m \mu_z/\mathrm{d}\chi_{1,z}{}^m$				$\mathrm{d}^m \alpha_{zz}/\mathrm{d}\chi_{1,z}{}^m$		
	1	2	3	4	0	1	2	3	0	1	2
Basis set	[$\times10^{-8}$]	[$\times10^{-6}$]	[$\times10^{-5}$]	[$\times10^{-4}$]	[$\times10^{-7}$]	[$\times10^{-6}$]	[$\times10^{-4}$]	[$\times10^{-3}$]	[$\times10^{-5}$]	[$\times10^{-4}$]	[$\times10^{-3}$]
STO-3G	7.3	1.0	1.6	3.0	2.6	3.1	0.4	0.5	1.4	1.7	2.1
3-21G	27.3	5.6	13.0	26.5	3.6	6.3	1.3	2.4	2.3	3.9	4.9
3-21+G	24.9	5.1	11.8	23.9	3.6	6.3	1.3	2.4	2.9	4.6	5.8
3-21++G	24.9	5.1	11.8	23.9	3.6	6.3	1.3	2.4	2.9	4.6	5.9
6-31G	9.5	1.7	3.0	6.4	5.2	7.2	1.0	1.5	5.5	6.7	13.0
6-31G*	6.8	1.5	2.8	6.6	3.8	5.6	0.8	1.3	3.4	5.1	6.6
6-31G**	9.1	1.8	3.4	7.2	3.0	4.0	0.6	0.9	3.3	5.3	6.8
6-31+G	9.4	1.7	2.9	6.1	5.8	7.8	1.1	1.5	5.8	7.0	11.0
6-31++G	9.3	1.7	2.9	6.1	6.0	7.8	1.1	1.5	5.8	7.1	11.1
6-31+G*	6.7	1.5	2.7	6.4	4.3	5.8	0.9	1.3	3.7	5.8	7.0
6-31++G**	8.5	1.7	3.2	7.0	3.3	4.7	0.7	1.1	3.8	5.1	7.4
6-311G	6.9	1.2	1.9	3.8	5.0	8.2	1.1	1.8	5.9	16.4	15.9
6-311G*	5.5	1.1	1.8	4.0	4.6	7.0	1.0	1.6	4.9	10.7	18.8
6-311G**	8.2	1.3	2.6	4.8	13.3	5.7	0.9	1.2	4.4	6.4	9.3
6-311+G	6.5	1.1	1.8	3.6	6.4	8.3	1.1	1.7	6.1	15.8	17.5
6-311++G	6.5	1.1	1.8	3.6	6.4	8.3	1.1	1.7	6.1	16.0	17.6
6-311+G*	5.5	1.0	1.7	3.9	6.2	6.5	1.0	1.5	4.5	9.7	24.5
6-311++G**	7.2	1.2	2.3	4.5	4.0	5.8	0.9	1.3	4.4	5.8	11.1
Def2SVP	16.9	1.1	1.8	2.8	4.5	6.0	0.9	1.2	4.3	6.5	9.3
Def2TZVP	8.1	1.6	3.2	6.8	2.6	3.9	0.6	0.9	3.1	4.1	6.0
ma-Def2SVP	18.7	1.1	1.6	2.7	4.3	6.3	1.0	1.5	4.7	6.2	12.1
ma-Def2TZVP	7.8	1.6	3.2	6.8	2.6	4.0	0.6	0.9	3.1	4.0	6.0
cc-pVTZ	14.2	2.7	5.3	11.0	4.4	6.1	0.9	1.4	4.7	7.4	14.0
cc-pVDZ	6.1	1.3	2.4	5.3	2.2	2.8	0.4	0.6	2.6	3.4	5.1
aug-cc-pVDZ	10.7	2.3	4.4	9.8	5.2	8.6	1.3	2.1	5.5	7.7	30.8
aug-cc-pVTZ	5.7	1.2	2.3	5.2	1.8	2.7	0.4	0.7	10.2	5.8	4.1

Table S8: RMSE values for various property derivatives obtained with different basis sets and B3LYP combined with the (250, 974) integration grid for the HCN · HF system. For each column, the values in cells have to be multiplied with the exponent given in top.

	$\mathrm{d}^m E/\mathrm{d}\chi_{1,z}{}^m$				$\mathrm{d}^m \mu_z/\mathrm{d}\chi_{1,z}{}^m$				$\mathrm{d}^m \alpha_{zz}/\mathrm{d}\chi_{1,z}{}^m$		
	1	2	3	4	0	1	2	3	0	1	2
Basis set	[$\times 10^{-10}$]	[$\times 10^{-9}$]	[$\times 10^{-8}$]	[$\times 10^{-7}$]	[$\times 10^{-10}$]	[$\times 10^{-8}$]	[$\times 10^{-7}$]	[$\times 10^{-6}$]	[$\times 10^{-7}$]	[$\times 10^{-6}$]	[$\times 10^{-5}$]
STO-3G	2.1	6.9	7.0	7.9	8.7	1.0	275	.3 2.2	0.4	0.7	5.4
3-21G	6.0	11.9	23.2	22.0	9.1	1.1	2.4	2.8	0.7	1.1	1.7
3-21+G	5.2	15.9	21.8	21.1	10.5	1.2	2.1	2.9	0.7	1.2	1.7
3-21++G	5.3	15.7	21.7	21.1	8.5	1.2	2.1	2.9	0.7	1.2	1.7
6-31G	2.5	6.9	18.8	17.9	14.9	2.6	18.	5 5.9	1.3	1.8	2.9
6-31G*	2.7	6.8	104.0	16.2	9.6	1.4	17.	7 2.8	1.0	1.4	2.3
6-31G**	1.3	2.5	1.3	4.8	8.5	1.2	1.9	2.7	0.8	1.2	1.7
6-31+G	2.5	8.2	19.0	18.1	15.8	32.4	6.6	6.2	1.8	2.0	3.3
6-31++G	2.5	8.2	19.0	18.1	15.8	32.0	6.7	6.8	1.8	2.0	3.3
6-31+G*	4.4	9.3	13.4	20.9	22.5	1.5	2.2	3.0	1.0	1.5	2.2
6-31++G**	2.0	7.4	19.5	18.6	7.4	1.1	1.5	2.3	0.8	1.1	1.7
6-311G	5.7	1.6	3.4	10.6	14.5	28.9	14.	3 5.4	1.4	72.3	8.7
6-311G*	1.4	2.4	2.2	6.6	12.1	1.9	2.8	4.6	1.3	2.2	3.5
6-311G**	0.9	66.8	1.1	5.3	8.4	1.4	1.9	3.1	1.2	1.4	2.7
6-311+G	1.0	1.6	4.0	8.2	14.2	1.8	6.7	4.5	1.5	2.6	3.9
6-311++G	1.0	1.6	4.1	8.2	14.6	1.8	6.6	4.5	1.5	2.5	3.9
6-311+G*	1.3	2.3	2.6	6.7	10.9	1.7	2.6	4.0	1.2	19.3	4.9
6-311++G**	0.9	51.4	1.5	5.9	8.5	1.5	1.8	3.5	1.1	1.3	5.1
Def2SVP	12.7	237.6	28.6	29.5	9.7	1.6	15.	1 3.3	1.0	1.5	2.7
Def2TZVP	0.9	1.9	3.4	9.6	6.1	1.2	4.9	2.1	1.0	0.9	1.8
ma-Def2SVP	12.0	8.5	27.4	28.7	10.4	1.6	2.4	4.1	0.9	1.3	2.2
ma-Def2TZVP	0.9	2.1	3.2	9.2	6.0	1.0	1.3	1.9	1.0	0.9	1.6
cc-pVTZ	3.2	3.3	5.6	8.2	11.2	1.8	3.7	3.7	1.1	1.7	2.9
cc-pVDZ	1.1	2.0	3.1	14.9	4.2	23.6	7.6	1.8	0.5	0.8	1.3
aug-cc-pVDZ	4.4	6.7	10.2	18.3	29.1	1.8	3.0	4.8	6.9	2.7	3.1
aug-cc-pVTZ	0.7	0.6	1.8	16.9	185.4	7.5	7.9	14.6	173.0	176.7	314.5

Table S9: RMSE values for various energy derivatives obtained with the different functionals used in this work combined with the (99, 590) integration grid for the HCN · HF system.

	$\mathrm{d}^n E_c/\mathrm{d}\xi^n$ [$\times 10^{-8}$]					$\mathrm{d}^n E_x/\mathrm{d}\xi^n$ [$\times 10^{-8}$]				
**DFA**	0	1	2	3	4	0	1	2	3	4
SVWN5	$7.29 \cdot 10^{-2}$	$5.27 \cdot 10^{-1}$	$3.44 \cdot 10^{0}$	$2.68 \cdot 10^{1}$	$2.12 \cdot 10^{2}$	$2.28 \cdot 10^{0}$	$7.35 \cdot 10^{0}$	$2.95 \cdot 10^{1}$	$1.91 \cdot 10^{2}$	$1.91 \cdot 10^{3}$
SPW92	$7.34 \cdot 10^{-2}$	$5.24 \cdot 10^{-1}$	$3.42 \cdot 10^{0}$	$2.62 \cdot 10^{1}$	$2.04 \cdot 10^{2}$	$2.28 \cdot 10^{0}$	$6.32 \cdot 10^{0}$	$2.94 \cdot 10^{1}$	$1.90 \cdot 10^{2}$	$1.91 \cdot 10^{3}$
BHH	$3.55 \cdot 10^{-1}$	$1.41 \cdot 10^{0}$	$1.28 \cdot 10^{1}$	$1.19 \cdot 10^{2}$	$1.27 \cdot 10^{3}$	$8.90 \cdot 10^{-1}$	$5.28 \cdot 10^{0}$	$2.13 \cdot 10^{1}$	$1.97 \cdot 10^{2}$	$1.56 \cdot 10^{3}$
BLYP	$2.63 \cdot 10^{-1}$	$9.71 \cdot 10^{-1}$	$7.88 \cdot 10^{0}$	$6.03 \cdot 10^{1}$	$5.83 \cdot 10^{2}$	$6.36 \cdot 10^{0}$	$1.12 \cdot 10^{1}$	$9.72 \cdot 10^{1}$	$8.74 \cdot 10^{2}$	$7.83 \cdot 10^{3}$
B1LYP	$5.10 \cdot 10^{-2}$	$7.85 \cdot 10^{-1}$	$6.88 \cdot 10^{0}$	$5.40 \cdot 10^{1}$	$5.11 \cdot 10^{2}$	$9.27 \cdot 10^{-1}$	$1.23 \cdot 10^{1}$	$8.27 \cdot 10^{1}$	$6.01 \cdot 10^{2}$	$5.63 \cdot 10^{3}$
B3LYP	$4.48 \cdot 10^{-2}$	$5.52 \cdot 10^{-1}$	$4.92 \cdot 10^{0}$	$4.21 \cdot 10^{1}$	$4.25 \cdot 10^{2}$	$1.94 \cdot 10^{0}$	$1.39 \cdot 10^{1}$	$7.95 \cdot 10^{1}$	$6.71 \cdot 10^{2}$	$5.70 \cdot 10^{3}$
BHHLYP	$3.64 \cdot 10^{-1}$	$7.58 \cdot 10^{-1}$	$7.01 \cdot 10^{0}$	$5.30 \cdot 10^{1}$	$5.14 \cdot 10^{2}$	$2.22 \cdot 10^{0}$	$9.03 \cdot 10^{0}$	$6.56 \cdot 10^{1}$	$4.26 \cdot 10^{2}$	$3.75 \cdot 10^{3}$
LC-BLYP	$1.70 \cdot 10^{-1}$	$1.50 \cdot 10^{0}$	$1.14 \cdot 10^{1}$	$9.33 \cdot 10^{1}$	$8.65 \cdot 10^{2}$	$5.18 \cdot 10^{0}$	$1.77 \cdot 10^{1}$	$9.59 \cdot 10^{1}$	$6.25 \cdot 10^{2}$	$5.69 \cdot 10^{3}$
CAMB3LYP	$9.61 \cdot 10^{-2}$	$7.43 \cdot 10^{-1}$	$5.52 \cdot 10^{0}$	$5.62 \cdot 10^{1}$	$4.13 \cdot 10^{2}$	$3.09 \cdot 10^{0}$	$1.70 \cdot 10^{1}$	$8.10 \cdot 10^{1}$	$7.14 \cdot 10^{2}$	$4.78 \cdot 10^{3}$
PBE	$1.23 \cdot 10^{0}$	$7.02 \cdot 10^{0}$	$6.26 \cdot 10^{1}$	$5.60 \cdot 10^{2}$	$4.59 \cdot 10^{3}$	$8.14 \cdot 10^{0}$	$1.89 \cdot 10^{1}$	$1.59 \cdot 10^{2}$	$1.14 \cdot 10^{3}$	$1.06 \cdot 10^{4}$
PBE0	$5.75 \cdot 10^{-1}$	$6.74 \cdot 10^{0}$	$6.13 \cdot 10^{1}$	$4.34 \cdot 10^{2}$	$4.61 \cdot 10^{3}$	$2.62 \cdot 10^{0}$	$2.01 \cdot 10^{1}$	$9.26 \cdot 10^{1}$	$8.94 \cdot 10^{2}$	$5.66 \cdot 10^{3}$
PBE50	$2.15 \cdot 10^{0}$	$6.20 \cdot 10^{0}$	$6.04 \cdot 10^{1}$	$4.46 \cdot 10^{2}$	$4.18 \cdot 10^{3}$	$2.81 \cdot 10^{0}$	$1.12 \cdot 10^{1}$	$6.59 \cdot 10^{1}$	$5.33 \cdot 10^{2}$	$3.88 \cdot 10^{3}$
LC-wPBE	$1.12 \cdot 10^{0}$	$6.95 \cdot 10^{0}$	$6.18 \cdot 10^{1}$	$4.19 \cdot 10^{2}$	$4.42 \cdot 10^{3}$	$5.09 \cdot 10^{0}$	$2.02 \cdot 10^{1}$	$9.94 \cdot 10^{1}$	$1.05 \cdot 10^{3}$	$7.29 \cdot 10^{3}$
TPSS	$1.18 \cdot 10^{0}$	$7.06 \cdot 10^{0}$	$7.01 \cdot 10^{1}$	$5.90 \cdot 10^{2}$	$5.40 \cdot 10^{3}$	$2.04 \cdot 10^{1}$	$2.88 \cdot 10^{1}$	$1.94 \cdot 10^{2}$	$1.52 \cdot 10^{3}$	$1.56 \cdot 10^{4}$
RevTPSS	$1.47 \cdot 10^{0}$	$8.20 \cdot 10^{0}$	$6.26 \cdot 10^{1}$	$4.82 \cdot 10^{2}$	$4.67 \cdot 10^{3}$	$1.37 \cdot 10^{1}$	$2.47 \cdot 10^{1}$	$1.90 \cdot 10^{2}$	$1.86 \cdot 10^{3}$	$1.75 \cdot 10^{4}$
TPSSh	$7.87 \cdot 10^{-1}$	$7.42 \cdot 10^{0}$	$6.81 \cdot 10^{1}$	$5.30 \cdot 10^{2}$	$5.33 \cdot 10^{3}$	$1.78 \cdot 10^{1}$	$2.77 \cdot 10^{1}$	$1.80 \cdot 10^{2}$	$1.46 \cdot 10^{3}$	$1.37 \cdot 10^{4}$
B97	$3.44 \cdot 10^{1}$	$3.78 \cdot 10^{2}$	$3.66 \cdot 10^{3}$	$4.10 \cdot 10^{4}$	$3.94 \cdot 10^{5}$	$2.28 \cdot 10^{0}$	$1.49 \cdot 10^{1}$	$8.13 \cdot 10^{1}$	$6.31 \cdot 10^{2}$	$4.99 \cdot 10^{3}$
B97-D	$2.15 \cdot 10^{1}$	$2.29 \cdot 10^{2}$	$2.50 \cdot 10^{3}$	$2.65 \cdot 10^{4}$	$2.77 \cdot 10^{5}$	$1.14 \cdot 10^{1}$	$2.84 \cdot 10^{1}$	$1.48 \cdot 10^{2}$	$1.02 \cdot 10^{3}$	$7.91 \cdot 10^{3}$
wB97	$4.37 \cdot 10^{1}$	$3.12 \cdot 10^{2}$	$3.48 \cdot 10^{3}$	$2.93 \cdot 10^{4}$	$4.36 \cdot 10^{5}$	$4.71 \cdot 10^{0}$	$1.00 \cdot 10^{1}$	$7.46 \cdot 10^{1}$	$5.63 \cdot 10^{2}$	$4.72 \cdot 10^{3}$
wB97X	$4.16 \cdot 10^{1}$	$3.82 \cdot 10^{2}$	$3.92 \cdot 10^{3}$	$3.88 \cdot 10^{4}$	$5.74 \cdot 10^{5}$	$1.30 \cdot 10^{0}$	$1.45 \cdot 10^{1}$	$8.62 \cdot 10^{1}$	$6.58 \cdot 10^{2}$	$6.03 \cdot 10^{3}$
wB97X-D	$6.83 \cdot 10^{1}$	$5.83 \cdot 10^{2}$	$6.46 \cdot 10^{3}$	$6.86 \cdot 10^{4}$	$9.51 \cdot 10^{5}$	$2.16 \cdot 10^{0}$	$1.08 \cdot 10^{1}$	$8.45 \cdot 10^{1}$	$5.26 \cdot 10^{2}$	$5.48 \cdot 10^{3}$
wB97X-D3	$4.56 \cdot 10^{1}$	$4.10 \cdot 10^{2}$	$4.39 \cdot 10^{3}$	$4.43 \cdot 10^{4}$	$6.46 \cdot 10^{5}$	$1.06 \cdot 10^{0}$	$1.11 \cdot 10^{1}$	$8.11 \cdot 10^{1}$	$5.33 \cdot 10^{2}$	$5.42 \cdot 10^{3}$
wB97M-V	$9.49 \cdot 10^{0}$	$2.52 \cdot 10^{1}$	$3.04 \cdot 10^{2}$	$2.32 \cdot 10^{3}$	$2.44 \cdot 10^{4}$	$1.25 \cdot 10^{0}$	$1.36 \cdot 10^{1}$	$7.71 \cdot 10^{1}$	$5.91 \cdot 10^{2}$	$4.90 \cdot 10^{3}$
wB97X-V	$4.52 \cdot 10^{0}$	$3.78 \cdot 10^{1}$	$2.69 \cdot 10^{2}$	$2.68 \cdot 10^{3}$	$2.55 \cdot 10^{4}$	$2.73 \cdot 10^{0}$	$1.38 \cdot 10^{1}$	$8.76 \cdot 10^{1}$	$6.49 \cdot 10^{2}$	$5.60 \cdot 10^{3}$
B97M-V	$9.00 \cdot 10^{0}$	$5.85 \cdot 10^{1}$	$4.78 \cdot 10^{2}$	$4.41 \cdot 10^{3}$	$4.79 \cdot 10^{4}$	$7.77 \cdot 10^{0}$	$1.70 \cdot 10^{1}$	$1.14 \cdot 10^{2}$	$1.15 \cdot 10^{3}$	$8.97 \cdot 10^{3}$
B1PW91	$6.69 \cdot 10^{-1}$	$6.75 \cdot 10^{0}$	$4.95 \cdot 10^{1}$	$3.81 \cdot 10^{2}$	$4.77 \cdot 10^{3}$	$1.77 \cdot 10^{0}$	$1.52 \cdot 10^{1}$	$8.75 \cdot 10^{1}$	$6.97 \cdot 10^{2}$	$5.60 \cdot 10^{3}$
PW91	$7.34 \cdot 10^{-1}$	$6.79 \cdot 10^{0}$	$6.06 \cdot 10^{1}$	$6.19 \cdot 10^{2}$	$7.82 \cdot 10^{3}$	$8.51 \cdot 10^{0}$	$2.72 \cdot 10^{1}$	$2.37 \cdot 10^{2}$	$2.10 \cdot 10^{3}$	$3.74 \cdot 10^{4}$
mPW91	$1.01 \cdot 10^{0}$	$6.70 \cdot 10^{0}$	$5.83 \cdot 10^{1}$	$5.33 \cdot 10^{2}$	$6.59 \cdot 10^{3}$	$7.30 \cdot 10^{0}$	$2.33 \cdot 10^{1}$	$1.93 \cdot 10^{2}$	$2.03 \cdot 10^{3}$	$3.40 \cdot 10^{4}$
VSXC	$4.76 \cdot 10^{1}$	$1.24 \cdot 10^{2}$	$4.11 \cdot 10^{2}$	$4.10 \cdot 10^{3}$	$1.85 \cdot 10^{4}$	$6.14 \cdot 10^{0}$	$4.98 \cdot 10^{1}$	$1.65 \cdot 10^{2}$	$1.53 \cdot 10^{3}$	$8.50 \cdot 10^{3}$
SCAN	$8.85 \cdot 10^{1}$	$8.25 \cdot 10^{2}$	$9.82 \cdot 10^{3}$	$1.42 \cdot 10^{5}$	$3.45 \cdot 10^{6}$	$3.22 \cdot 10^{2}$	$1.69 \cdot 10^{3}$	$2.52 \cdot 10^{4}$	$3.60 \cdot 10^{5}$	$9.47 \cdot 10^{6}$
SCAN0	$8.55 \cdot 10^{1}$	$9.13 \cdot 10^{2}$	$8.91 \cdot 10^{3}$	$1.26 \cdot 10^{5}$	$2.24 \cdot 10^{6}$	$1.37 \cdot 10^{2}$	$1.69 \cdot 10^{3}$	$1.87 \cdot 10^{4}$	$2.51 \cdot 10^{5}$	$4.49 \cdot 10^{6}$
M06	$3.02 \cdot 10^{1}$	$2.30 \cdot 10^{2}$	$3.05 \cdot 10^{3}$	$2.61 \cdot 10^{4}$	$3.26 \cdot 10^{5}$	$4.75 \cdot 10^{1}$	$1.89 \cdot 10^{2}$	$1.68 \cdot 10^{3}$	$1.41 \cdot 10^{4}$	$1.42 \cdot 10^{5}$
M06-L	$4.69 \cdot 10^{1}$	$3.78 \cdot 10^{2}$	$4.77 \cdot 10^{3}$	$4.14 \cdot 10^{4}$	$4.94 \cdot 10^{5}$	$7.67 \cdot 10^{1}$	$1.39 \cdot 10^{2}$	$1.23 \cdot 10^{3}$	$1.35 \cdot 10^{4}$	$1.72 \cdot 10^{5}$
M06-HF	$5.89 \cdot 10^{1}$	$4.39 \cdot 10^{2}$	$4.40 \cdot 10^{3}$	$4.32 \cdot 10^{4}$	$4.75 \cdot 10^{5}$	$1.41 \cdot 10^{2}$	$5.51 \cdot 10^{2}$	$4.93 \cdot 10^{3}$	$4.97 \cdot 10^{4}$	$5.23 \cdot 10^{5}$
M06-2X	$3.17 \cdot 10^{1}$	$2.71 \cdot 10^{2}$	$2.65 \cdot 10^{3}$	$2.61 \cdot 10^{4}$	$2.67 \cdot 10^{5}$	$6.24 \cdot 10^{1}$	$2.28 \cdot 10^{2}$	$1.56 \cdot 10^{3}$	$1.42 \cdot 10^{4}$	$1.93 \cdot 10^{5}$
M11	$1.30 \cdot 10^{1}$	$6.72 \cdot 10^{1}$	$5.88 \cdot 10^{2}$	$4.71 \cdot 10^{3}$	$4.81 \cdot 10^{4}$	$7.45 \cdot 10^{1}$	$2.06 \cdot 10^{2}$	$1.90 \cdot 10^{3}$	$1.56 \cdot 10^{4}$	$1.78 \cdot 10^{5}$
M11-L	$3.48 \cdot 10^{1}$	$6.93 \cdot 10^{1}$	$7.17 \cdot 10^{2}$	$5.59 \cdot 10^{3}$	$5.31 \cdot 10^{4}$	$7.76 \cdot 10^{1}$	$2.90 \cdot 10^{2}$	$2.54 \cdot 10^{3}$	$2.16 \cdot 10^{4}$	$1.80 \cdot 10^{5}$
SOGGA11	$1.66 \cdot 10^{2}$	$1.62 \cdot 10^{2}$	$1.19 \cdot 10^{3}$	$1.00 \cdot 10^{4}$	$9.61 \cdot 10^{4}$	$2.50 \cdot 10^{1}$	$5.09 \cdot 10^{1}$	$1.74 \cdot 10^{2}$	$1.15 \cdot 10^{3}$	$7.05 \cdot 10^{3}$
SOGGA11-X	$1.95 \cdot 10^{1}$	$7.08 \cdot 10^{1}$	$1.70 \cdot 10^{2}$	$1.81 \cdot 10^{3}$	$4.12 \cdot 10^{4}$	$2.63 \cdot 10^{0}$	$9.87 \cdot 10^{0}$	$4.08 \cdot 10^{1}$	$2.95 \cdot 10^{2}$	$2.38 \cdot 10^{3}$
N12	$5.44 \cdot 10^{1}$	$7.52 \cdot 10^{2}$	$7.64 \cdot 10^{3}$	$9.99 \cdot 10^{4}$	$1.35 \cdot 10^{6}$	$1.52 \cdot 10^{1}$	$6.72 \cdot 10^{1}$	$1.83 \cdot 10^{2}$	$1.51 \cdot 10^{3}$	$1.63 \cdot 10^{4}$
N12-SX	$1.78 \cdot 10^{1}$	$2.67 \cdot 10^{2}$	$2.70 \cdot 10^{3}$	$3.22 \cdot 10^{4}$	$3.46 \cdot 10^{5}$	$7.54 \cdot 10^{0}$	$4.02 \cdot 10^{1}$	$2.11 \cdot 10^{2}$	$1.91 \cdot 10^{3}$	$1.98 \cdot 10^{4}$
MN12-L	$1.65 \cdot 10^{1}$	$1.10 \cdot 10^{2}$	$7.15 \cdot 10^{2}$	$5.77 \cdot 10^{3}$	$4.39 \cdot 10^{4}$	$9.76 \cdot 10^{1}$	$2.01 \cdot 10^{2}$	$1.31 \cdot 10^{3}$	$1.08 \cdot 10^{4}$	$8.73 \cdot 10^{4}$
MN12-SX	$2.04 \cdot 10^{1}$	$1.14 \cdot 10^{2}$	$5.47 \cdot 10^{2}$	$4.67 \cdot 10^{3}$	$4.00 \cdot 10^{4}$	$7.71 \cdot 10^{1}$	$1.29 \cdot 10^{2}$	$7.74 \cdot 10^{2}$	$6.25 \cdot 10^{3}$	$6.38 \cdot 10^{4}$
MN15	$2.12 \cdot 10^{1}$	$1.17 \cdot 10^{2}$	$9.88 \cdot 10^{2}$	$6.86 \cdot 10^{3}$	$6.73 \cdot 10^{4}$	$1.79 \cdot 10^{1}$	$5.84 \cdot 10^{1}$	$5.42 \cdot 10^{2}$	$3.86 \cdot 10^{3}$	$3.69 \cdot 10^{4}$
MN15-L	$1.16 \cdot 10^{1}$	$6.48 \cdot 10^{1}$	$5.14 \cdot 10^{2}$	$3.86 \cdot 10^{3}$	$3.42 \cdot 10^{4}$	$3.08 \cdot 10^{1}$	$7.29 \cdot 10^{1}$	$6.37 \cdot 10^{2}$	$3.37 \cdot 10^{3}$	$3.38 \cdot 10^{4}$

Table S10: RMSE values for various energy derivatives obtained with the different functionals used in this work combined with the (250, 974) integration grid for the HCN · HF system.

DFA	$\mathrm{d}^nE_c/\mathrm{d}\xi^n$ [$\times 10^{-8}$]					$\mathrm{d}^nE_x/\mathrm{d}\xi^n$ [$\times 10^{-8}$]				
	0	1	2	3	4	0	1	2	3	4
SVWN5	$2.7 \cdot 10^{-3}$	$1.6 \cdot 10^{-3}$	$3.1 \cdot 10^{-2}$	$9.3 \cdot 10^{-1}$	$1.8 \cdot 10^{1}$	$2.4 \cdot 10^{-3}$	$2.8 \cdot 10^{-3}$	$2.9 \cdot 10^{-2}$	$5.0 \cdot 10^{-1}$	$7.5 \cdot 10^{0}$
SPW92	$2.6 \cdot 10^{-3}$	$1.8 \cdot 10^{-3}$	$1.6 \cdot 10^{-2}$	$3.2 \cdot 10^{-1}$	$6.7 \cdot 10^{0}$	$3.0 \cdot 10^{-3}$	$1.1 \cdot 10^{0}$	$2.2 \cdot 10^{-2}$	$5.9 \cdot 10^{-1}$	$1.6 \cdot 10^{1}$
BHH	$3.2 \cdot 10^{-3}$	$3.7 \cdot 10^{-3}$	$1.7 \cdot 10^{-1}$	$9.5 \cdot 10^{-1}$	$7.3 \cdot 10^{0}$	$3.0 \cdot 10^{-3}$	$4.6 \cdot 10^{-1}$	$5.3 \cdot 10^{-2}$	$6.4 \cdot 10^{-1}$	$1.2 \cdot 10^{1}$
BLYP	$2.6 \cdot 10^{-3}$	$1.1 \cdot 10^{-3}$	$2.2 \cdot 10^{-2}$	$4.0 \cdot 10^{-1}$	$8.8 \cdot 10^{0}$	$4.0 \cdot 10^{-3}$	$1.8 \cdot 10^{0}$	$8.1 \cdot 10^{-2}$	$2.0 \cdot 10^{0}$	$4.0 \cdot 10^{1}$
B1LYP	$2.8 \cdot 10^{-3}$	$1.5 \cdot 10^{-3}$	$2.4 \cdot 10^{-2}$	$5.5 \cdot 10^{-1}$	$7.9 \cdot 10^{0}$	$3.7 \cdot 10^{-3}$	$3.6 \cdot 10^{-3}$	$4.7 \cdot 10^{-2}$	$1.1 \cdot 10^{0}$	$2.3 \cdot 10^{1}$
B3LYP	$2.9 \cdot 10^{-3}$	$1.8 \cdot 10^{-3}$	$4.4 \cdot 10^{-2}$	$6.4 \cdot 10^{-1}$	$1.3 \cdot 10^{1}$	$3.5 \cdot 10^{-3}$	$1.2 \cdot 10^{0}$	$4.8 \cdot 10^{-2}$	$9.4 \cdot 10^{-1}$	$1.8 \cdot 10^{1}$
BHHLYP	$7.7 \cdot 10^{-3}$	$2.5 \cdot 10^{-3}$	$5.6 \cdot 10^{-2}$	$3.4 \cdot 10^{-1}$	$6.5 \cdot 10^{0}$	$3.4 \cdot 10^{-3}$	$6.3 \cdot 10^{-1}$	$9.1 \cdot 10^{-2}$	$1.5 \cdot 10^{0}$	$1.8 \cdot 10^{1}$
LC-BLYP	$3.0 \cdot 10^{-3}$	$1.5 \cdot 10^{-1}$	$3.4 \cdot 10^{-2}$	$5.7 \cdot 10^{-1}$	$8.2 \cdot 10^{0}$	$2.9 \cdot 10^{-3}$	$5.4 \cdot 10^{-3}$	$4.5 \cdot 10^{-2}$	$7.9 \cdot 10^{-1}$	$1.5 \cdot 10^{1}$
CAMB3LYP	$7.0 \cdot 10^{-3}$	$2.3 \cdot 10^{-3}$	$3.0 \cdot 10^{-2}$	$6.1 \cdot 10^{-1}$	$1.8 \cdot 10^{1}$	$3.0 \cdot 10^{-3}$	$6.4 \cdot 10^{-3}$	$4.1 \cdot 10^{-2}$	$6.7 \cdot 10^{-1}$	$1.2 \cdot 10^{1}$
PBE	$2.6 \cdot 10^{-3}$	$6.0 \cdot 10^{-3}$	$5.9 \cdot 10^{-2}$	$8.5 \cdot 10^{-1}$	$1.3 \cdot 10^{1}$	$3.9 \cdot 10^{-3}$	$1.2 \cdot 10^{-2}$	$7.8 \cdot 10^{-2}$	$7.4 \cdot 10^{-1}$	$1.7 \cdot 10^{1}$
PBE0	$3.5 \cdot 10^{-3}$	$8.3 \cdot 10^{-3}$	$4.2 \cdot 10^{-2}$	$6.9 \cdot 10^{-1}$	$9.3 \cdot 10^{0}$	$2.9 \cdot 10^{-3}$	$5.8 \cdot 10^{-3}$	$4.4 \cdot 10^{-2}$	$9.3 \cdot 10^{-1}$	$1.5 \cdot 10^{1}$
PBE50	$3.6 \cdot 10^{-3}$	$1.3 \cdot 10^{-1}$	$6.3 \cdot 10^{-2}$	$8.9 \cdot 10^{-1}$	$8.9 \cdot 10^{0}$	$3.4 \cdot 10^{-3}$	$8.4 \cdot 10^{-1}$	$4.7 \cdot 10^{-2}$	$5.8 \cdot 10^{-1}$	$8.6 \cdot 10^{0}$
LC-wPBE	$3.5 \cdot 10^{-3}$	$6.7 \cdot 10^{-3}$	$8.2 \cdot 10^{-2}$	$1.2 \cdot 10^{0}$	$1.2 \cdot 10^{1}$	$7.1 \cdot 10^{-3}$	$2.0 \cdot 10^{-2}$	$9.6 \cdot 10^{-1}$	$9.9 \cdot 10^{-1}$	$1.1 \cdot 10^{1}$
TPSS	$6.7 \cdot 10^{-2}$	$3.4 \cdot 10^{-3}$	$2.5 \cdot 10^{-2}$	$7.0 \cdot 10^{-1}$	$1.2 \cdot 10^{1}$	$8.3 \cdot 10^{-3}$	$1.4 \cdot 10^{-1}$	$3.6 \cdot 10^{0}$	$6.5 \cdot 10^{1}$	$1.4 \cdot 10^{3}$
RevTPSS	$3.5 \cdot 10^{-3}$	$6.4 \cdot 10^{-3}$	$4.1 \cdot 10^{-2}$	$6.6 \cdot 10^{-1}$	$1.2 \cdot 10^{1}$	$7.5 \cdot 10^{-2}$	$7.7 \cdot 10^{-3}$	$1.3 \cdot 10^{-1}$	$2.3 \cdot 10^{0}$	$2.4 \cdot 10^{1}$
TPSSh	$3.7 \cdot 10^{-3}$	$3.0 \cdot 10^{-3}$	$3.1 \cdot 10^{-2}$	$7.1 \cdot 10^{-1}$	$1.3 \cdot 10^{1}$	$7.1 \cdot 10^{-3}$	$1.2 \cdot 10^{-1}$	$3.2 \cdot 10^{0}$	$4.9 \cdot 10^{1}$	$1.1 \cdot 10^{3}$
B97	$8.3 \cdot 10^{-1}$	$1.2 \cdot 10^{1}$	$1.6 \cdot 10^{2}$	$2.4 \cdot 10^{3}$	$3.4 \cdot 10^{4}$	$4.0 \cdot 10^{-3}$	$1.2 \cdot 10^{-2}$	$7.8 \cdot 10^{-2}$	$8.3 \cdot 10^{-1}$	$8.5 \cdot 10^{0}$
B97-D	$5.7 \cdot 10^{-1}$	$7.7 \cdot 10^{0}$	$1.1 \cdot 10^{2}$	$1.5 \cdot 10^{3}$	$2.3 \cdot 10^{4}$	$7.4 \cdot 10^{-3}$	$9.6 \cdot 10^{-3}$	$1.6 \cdot 10^{-1}$	$2.4 \cdot 10^{0}$	$1.8 \cdot 10^{1}$
wB97	$7.7 \cdot 10^{-1}$	$1.2 \cdot 10^{1}$	$2.1 \cdot 10^{2}$	$4.9 \cdot 10^{3}$	$1.3 \cdot 10^{5}$	$1.1 \cdot 10^{-1}$	$1.2 \cdot 10^{-2}$	$1.4 \cdot 10^{-1}$	$3.4 \cdot 10^{0}$	$3.2 \cdot 10^{1}$
wB97X	$1.8 \cdot 10^{0}$	$2.7 \cdot 10^{1}$	$4.1 \cdot 10^{2}$	$7.7 \cdot 10^{3}$	$1.7 \cdot 10^{5}$	$5.9 \cdot 10^{-3}$	$1.1 \cdot 10^{-2}$	$8.4 \cdot 10^{-2}$	$1.7 \cdot 10^{0}$	$1.2 \cdot 10^{1}$
wB97X-D	$3.4 \cdot 10^{0}$	$5.0 \cdot 10^{1}$	$8.2 \cdot 10^{2}$	$1.4 \cdot 10^{4}$	$3.3 \cdot 10^{5}$	$6.5 \cdot 10^{-3}$	$6.4 \cdot 10^{-3}$	$1.1 \cdot 10^{-1}$	$1.9 \cdot 10^{0}$	$1.1 \cdot 10^{1}$
wB97X-D3	$2.2 \cdot 10^{0}$	$3.2 \cdot 10^{1}$	$5.1 \cdot 10^{2}$	$9.6 \cdot 10^{3}$	$2.0 \cdot 10^{5}$	$4.3 \cdot 10^{-3}$	$8.0 \cdot 10^{-3}$	$9.4 \cdot 10^{-2}$	$1.3 \cdot 10^{0}$	$1.1 \cdot 10^{1}$
wB97M-V	$9.8 \cdot 10^{-2}$	$1.4 \cdot 10^{0}$	$2.1 \cdot 10^{1}$	$3.3 \cdot 10^{2}$	$5.4 \cdot 10^{3}$	$3.8 \cdot 10^{-3}$	$1.2 \cdot 10^{0}$	$5.1 \cdot 10^{-2}$	$9.8 \cdot 10^{-1}$	$1.6 \cdot 10^{1}$
wB97X-V	$3.4 \cdot 10^{-2}$	$4.4 \cdot 10^{-1}$	$7.3 \cdot 10^{0}$	$9.2 \cdot 10^{1}$	$1.7 \cdot 10^{3}$	$4.3 \cdot 10^{-3}$	$7.3 \cdot 10^{-3}$	$6.9 \cdot 10^{-2}$	$1.0 \cdot 10^{0}$	$1.5 \cdot 10^{1}$
B97M-V	$1.4 \cdot 10^{-1}$	$2.1 \cdot 10^{0}$	$2.3 \cdot 10^{1}$	$3.1 \cdot 10^{2}$	$4.5 \cdot 10^{3}$	$6.9 \cdot 10^{-3}$	$7.2 \cdot 10^{-3}$	$1.4 \cdot 10^{-1}$	$1.9 \cdot 10^{0}$	$2.4 \cdot 10^{1}$
B1PW91	$7.6 \cdot 10^{-3}$	$8.5 \cdot 10^{-3}$	$1.7 \cdot 10^{-1}$	$2.5 \cdot 10^{0}$	$4.3 \cdot 10^{1}$	$3.4 \cdot 10^{-3}$	$9.4 \cdot 10^{-3}$	$4.2 \cdot 10^{-2}$	$1.1 \cdot 10^{0}$	$2.3 \cdot 10^{1}$
PW91	$7.0 \cdot 10^{-3}$	$3.2 \cdot 10^{-1}$	$5.7 \cdot 10^{-1}$	$3.0 \cdot 10^{0}$	$6.4 \cdot 10^{1}$	$2.4 \cdot 10^{-2}$	$4.7 \cdot 10^{-1}$	$1.1 \cdot 10^{1}$	$3.0 \cdot 10^{2}$	$8.4 \cdot 10^{3}$
mPW91	$6.1 \cdot 10^{-3}$	$2.1 \cdot 10^{-1}$	$1.2 \cdot 10^{-1}$	$2.5 \cdot 10^{0}$	$5.5 \cdot 10^{1}$	$2.7 \cdot 10^{-2}$	$2.5 \cdot 10^{0}$	$1.3 \cdot 10^{1}$	$3.5 \cdot 10^{2}$	$9.1 \cdot 10^{3}$
VSXC	$3.8 \cdot 10^{-2}$	$1.3 \cdot 10^{0}$	$1.2 \cdot 10^{1}$	$2.3 \cdot 10^{2}$	$3.8 \cdot 10^{3}$	$1.8 \cdot 10^{-2}$	$7.8 \cdot 10^{-1}$	$5.6 \cdot 10^{0}$	$9.4 \cdot 10^{1}$	$1.8 \cdot 10^{3}$
SCAN	$5.5 \cdot 10^{0}$	$3.2 \cdot 10^{1}$	$7.2 \cdot 10^{2}$	$2.2 \cdot 10^{4}$	$1.0 \cdot 10^{6}$	$2.0 \cdot 10^{1}$	$1.0 \cdot 10^{2}$	$2.5 \cdot 10^{3}$	$7.5 \cdot 10^{4}$	$3.6 \cdot 10^{6}$
SCAN0	$6.1 \cdot 10^{0}$	$3.4 \cdot 10^{1}$	$5.2 \cdot 10^{2}$	$1.9 \cdot 10^{4}$	$9.3 \cdot 10^{5}$	$1.8 \cdot 10^{1}$	$1.0 \cdot 10^{2}$	$1.7 \cdot 10^{3}$	$5.8 \cdot 10^{4}$	$2.7 \cdot 10^{6}$
M06	$4.0 \cdot 10^{-1}$	$5.7 \cdot 10^{0}$	$8.8 \cdot 10^{1}$	$1.3 \cdot 10^{3}$	$2.1 \cdot 10^{4}$	$7.3 \cdot 10^{-2}$	$1.5 \cdot 10^{0}$	$3.5 \cdot 10^{1}$	$7.4 \cdot 10^{2}$	$1.7 \cdot 10^{4}$
M06-L	$5.1 \cdot 10^{-1}$	$7.5 \cdot 10^{0}$	$1.1 \cdot 10^{2}$	$1.6 \cdot 10^{3}$	$2.4 \cdot 10^{4}$	$3.4 \cdot 10^{-1}$	$7.6 \cdot 10^{0}$	$1.6 \cdot 10^{2}$	$3.6 \cdot 10^{3}$	$7.8 \cdot 10^{4}$
M06-HF	$4.0 \cdot 10^{-1}$	$6.4 \cdot 10^{0}$	$8.6 \cdot 10^{1}$	$1.4 \cdot 10^{3}$	$2.0 \cdot 10^{4}$	$7.5 \cdot 10^{-1}$	$1.6 \cdot 10^{1}$	$3.6 \cdot 10^{2}$	$7.6 \cdot 10^{3}$	$1.7 \cdot 10^{5}$
M06-2X	$3.9 \cdot 10^{-1}$	$5.9 \cdot 10^{0}$	$8.3 \cdot 10^{1}$	$1.3 \cdot 10^{3}$	$1.9 \cdot 10^{4}$	$3.6 \cdot 10^{-1}$	$7.1 \cdot 10^{0}$	$1.6 \cdot 10^{2}$	$3.2 \cdot 10^{3}$	$7.1 \cdot 10^{4}$
M11	$4.4 \cdot 10^{-2}$	$8.7 \cdot 10^{-1}$	$2.0 \cdot 10^{1}$	$3.9 \cdot 10^{2}$	$9.1 \cdot 10^{3}$	$7.7 \cdot 10^{-1}$	$6.8 \cdot 10^{0}$	$7.2 \cdot 10^{1}$	$1.4 \cdot 10^{3}$	$3.2 \cdot 10^{4}$
M11-L	$9.2 \cdot 10^{-2}$	$1.8 \cdot 10^{0}$	$3.5 \cdot 10^{1}$	$6.9 \cdot 10^{2}$	$1.3 \cdot 10^{4}$	$2.2 \cdot 10^{0}$	$9.7 \cdot 10^{0}$	$1.6 \cdot 10^{2}$	$3.2 \cdot 10^{3}$	$6.2 \cdot 10^{4}$
SOGGA11	$4.5 \cdot 10^{-1}$	$5.8 \cdot 10^{0}$	$9.9 \cdot 10^{1}$	$1.7 \cdot 10^{3}$	$3.0 \cdot 10^{4}$	$6.5 \cdot 10^{-2}$	$6.3 \cdot 10^{-2}$	$5.8 \cdot 10^{-1}$	$1.0 \cdot 10^{1}$	$4.5 \cdot 10^{2}$
SOGGA11-X	$3.5 \cdot 10^{-1}$	$4.4 \cdot 10^{0}$	$7.8 \cdot 10^{1}$	$1.6 \cdot 10^{3}$	$3.3 \cdot 10^{4}$	$5.8 \cdot 10^{-3}$	$7.4 \cdot 10^{-3}$	$1.0 \cdot 10^{-1}$	$1.8 \cdot 10^{0}$	$1.9 \cdot 10^{1}$
N12	$2.8 \cdot 10^{0}$	$4.1 \cdot 10^{1}$	$6.3 \cdot 10^{2}$	$1.0 \cdot 10^{4}$	$2.2 \cdot 10^{5}$	$2.0 \cdot 10^{-2}$	$1.3 \cdot 10^{0}$	$9.5 \cdot 10^{0}$	$2.2 \cdot 10^{2}$	$4.7 \cdot 10^{3}$
N12-SX	$5.4 \cdot 10^{-1}$	$8.0 \cdot 10^{0}$	$1.2 \cdot 10^{2}$	$2.0 \cdot 10^{3}$	$3.9 \cdot 10^{4}$	$1.0 \cdot 10^{-1}$	$1.3 \cdot 10^{0}$	$8.5 \cdot 10^{0}$	$1.7 \cdot 10^{2}$	$3.8 \cdot 10^{3}$
MN12-L	$3.1 \cdot 10^{-2}$	$5.8 \cdot 10^{-1}$	$1.1 \cdot 10^{1}$	$2.1 \cdot 10^{2}$	$4.1 \cdot 10^{3}$	$7.6 \cdot 10^{-2}$	$1.4 \cdot 10^{0}$	$2.8 \cdot 10^{1}$	$5.2 \cdot 10^{2}$	$1.0 \cdot 10^{4}$
MN12-SX	$9.5 \cdot 10^{-2}$	$1.9 \cdot 10^{0}$	$3.6 \cdot 10^{1}$	$7.3 \cdot 10^{2}$	$1.4 \cdot 10^{4}$	$4.8 \cdot 10^{-1}$	$4.0 \cdot 10^{0}$	$4.0 \cdot 10^{1}$	$7.6 \cdot 10^{2}$	$1.5 \cdot 10^{4}$
MN15	$1.2 \cdot 10^{-2}$	$2.2 \cdot 10^{-1}$	$4.7 \cdot 10^{0}$	$9.3 \cdot 10^{1}$	$2.0 \cdot 10^{3}$	$7.7 \cdot 10^{-1}$	$1.6 \cdot 10^{-2}$	$9.6 \cdot 10^{-2}$	$9.2 \cdot 10^{-1}$	$9.7 \cdot 10^{0}$
MN15-L	$1.6 \cdot 10^{-2}$	$2.7 \cdot 10^{-1}$	$4.7 \cdot 10^{0}$	$8.5 \cdot 10^{1}$	$1.5 \cdot 10^{3}$	$1.0 \cdot 10^{-2}$	$2.0 \cdot 10^{0}$	$3.2 \cdot 10^{0}$	$4.9 \cdot 10^{1}$	$9.3 \cdot 10^{2}$

Table S11: RMSE values for various energy derivatives obtained with the different functionals used in this work combined with the (99, 590) integration grid for the HCN · HCl system.

**DFA**	$\mathrm{d}^n E_c/\mathrm{d}\xi^n$ [$\times 10^{-8}$]					$\mathrm{d}^n E_x/\mathrm{d}\xi^n$ [$\times 10^{-8}$]				
	0	1	2	3	4	0	1	2	3	4
SVWN5	$6.2 \cdot 10^{-1}$	$3.1 \cdot 10^{-1}$	$1.3 \cdot 10^{0}$	$6.2 \cdot 10^{0}$	$3.0 \cdot 10^{1}$	$1.0 \cdot 10^{1}$	$8.5 \cdot 10^{0}$	$1.4 \cdot 10^{1}$	$4.5 \cdot 10^{1}$	$2.0 \cdot 10^{2}$
SPW92	$6.1 \cdot 10^{-1}$	$3.2 \cdot 10^{-1}$	$1.3 \cdot 10^{0}$	$6.2 \cdot 10^{0}$	$5.0 \cdot 10^{1}$	$1.0 \cdot 10^{1}$	$8.5 \cdot 10^{0}$	$1.4 \cdot 10^{1}$	$4.5 \cdot 10^{1}$	$2.0 \cdot 10^{2}$
BHH	$1.6 \cdot 10^{0}$	$6.8 \cdot 10^{-1}$	$4.0 \cdot 10^{0}$	$3.0 \cdot 10^{1}$	$2.2 \cdot 10^{2}$	$6.0 \cdot 10^{0}$	$1.4 \cdot 10^{1}$	$1.9 \cdot 10^{1}$	$6.5 \cdot 10^{1}$	$4.0 \cdot 10^{2}$
BLYP	$4.2 \cdot 10^{-1}$	$4.3 \cdot 10^{-1}$	$2.0 \cdot 10^{0}$	$1.3 \cdot 10^{1}$	$6.9 \cdot 10^{1}$	$1.6 \cdot 10^{0}$	$6.3 \cdot 10^{0}$	$6.7 \cdot 10^{1}$	$3.3 \cdot 10^{2}$	$2.1 \cdot 10^{3}$
B1LYP	$3.9 \cdot 10^{-1}$	$3.6 \cdot 10^{-1}$	$2.1 \cdot 10^{0}$	$1.1 \cdot 10^{1}$	$8.2 \cdot 10^{1}$	$7.9 \cdot 10^{0}$	$6.9 \cdot 10^{0}$	$5.1 \cdot 10^{1}$	$2.7 \cdot 10^{2}$	$1.6 \cdot 10^{3}$
B3LYP	$2.6 \cdot 10^{-1}$	$3.8 \cdot 10^{-1}$	$1.8 \cdot 10^{0}$	$1.2 \cdot 10^{1}$	$7.7 \cdot 10^{1}$	$8.8 \cdot 10^{0}$	$5.2 \cdot 10^{0}$	$5.3 \cdot 10^{1}$	$2.5 \cdot 10^{2}$	$1.7 \cdot 10^{3}$
BHHLYP	$4.5 \cdot 10^{-1}$	$3.2 \cdot 10^{-1}$	$2.1 \cdot 10^{0}$	$1.1 \cdot 10^{1}$	$7.7 \cdot 10^{1}$	$7.9 \cdot 10^{0}$	$5.5 \cdot 10^{0}$	$3.6 \cdot 10^{1}$	$1.9 \cdot 10^{2}$	$1.2 \cdot 10^{3}$
LC-BLYP	$7.9 \cdot 10^{-1}$	$4.8 \cdot 10^{-1}$	$1.8 \cdot 10^{0}$	$1.6 \cdot 10^{1}$	$6.1 \cdot 10^{1}$	$1.5 \cdot 10^{1}$	$1.3 \cdot 10^{1}$	$2.5 \cdot 10^{1}$	$1.2 \cdot 10^{2}$	$5.6 \cdot 10^{2}$
CAMB3LYP	$3.3 \cdot 10^{-1}$	$2.7 \cdot 10^{-1}$	$1.8 \cdot 10^{0}$	$8.7 \cdot 10^{0}$	$7.5 \cdot 10^{1}$	$1.2 \cdot 10^{1}$	$8.3 \cdot 10^{0}$	$3.6 \cdot 10^{1}$	$2.0 \cdot 10^{2}$	$1.1 \cdot 10^{3}$
PBE	$8.1 \cdot 10^{0}$	$1.7 \cdot 10^{0}$	$1.1 \cdot 10^{1}$	$9.2 \cdot 10^{1}$	$4.4 \cdot 10^{2}$	$7.7 \cdot 10^{0}$	$2.1 \cdot 10^{1}$	$5.5 \cdot 10^{1}$	$2.4 \cdot 10^{2}$	$1.8 \cdot 10^{3}$
PBE0	$6.7 \cdot 10^{0}$	$2.7 \cdot 10^{0}$	$1.2 \cdot 10^{1}$	$7.4 \cdot 10^{1}$	$5.1 \cdot 10^{2}$	$1.0 \cdot 10^{1}$	$1.3 \cdot 10^{1}$	$2.8 \cdot 10^{1}$	$1.8 \cdot 10^{2}$	$7.8 \cdot 10^{2}$
PBE50	$6.1 \cdot 10^{0}$	$2.9 \cdot 10^{0}$	$1.2 \cdot 10^{1}$	$9.4 \cdot 10^{1}$	$4.0 \cdot 10^{2}$	$1.0 \cdot 10^{1}$	$5.3 \cdot 10^{0}$	$1.7 \cdot 10^{1}$	$1.0 \cdot 10^{2}$	$5.6 \cdot 10^{2}$
LC-wPBE	$4.7 \cdot 10^{0}$	$2.4 \cdot 10^{0}$	$1.7 \cdot 10^{1}$	$7.6 \cdot 10^{1}$	$5.9 \cdot 10^{2}$	$7.6 \cdot 10^{0}$	$6.5 \cdot 10^{0}$	$2.1 \cdot 10^{1}$	$1.1 \cdot 10^{2}$	$7.0 \cdot 10^{2}$
PBE-GXLY	$5.5 \cdot 10^{0}$	$3.3 \cdot 10^{0}$	$4.4 \cdot 10^{0}$	$2.5 \cdot 10^{1}$	$1.8 \cdot 10^{2}$	$2.4 \cdot 10^{3}$	$4.0 \cdot 10^{3}$	$1.0 \cdot 10^{5}$	$3.2 \cdot 10^{6}$	$1.3 \cdot 10^{8}$
TPSS	$7.6 \cdot 10^{0}$	$2.6 \cdot 10^{0}$	$2.1 \cdot 10^{1}$	$9.5 \cdot 10^{1}$	$9.0 \cdot 10^{2}$	$1.3 \cdot 10^{1}$	$4.0 \cdot 10^{1}$	$1.9 \cdot 10^{2}$	$1.0 \cdot 10^{3}$	$6.9 \cdot 10^{3}$
RevTPSS	$6.5 \cdot 10^{0}$	$2.7 \cdot 10^{0}$	$1.1 \cdot 10^{1}$	$1.1 \cdot 10^{2}$	$4.9 \cdot 10^{2}$	$7.7 \cdot 10^{0}$	$2.8 \cdot 10^{1}$	$1.4 \cdot 10^{2}$	$9.1 \cdot 10^{2}$	$5.1 \cdot 10^{3}$
TPSSh	$7.1 \cdot 10^{0}$	$3.2 \cdot 10^{0}$	$1.8 \cdot 10^{1}$	$1.1 \cdot 10^{2}$	$7.8 \cdot 10^{2}$	$7.8 \cdot 10^{0}$	$3.2 \cdot 10^{1}$	$1.7 \cdot 10^{2}$	$9.5 \cdot 10^{2}$	$5.8 \cdot 10^{3}$
B97	$1.2 \cdot 10^{2}$	$6.3 \cdot 10^{2}$	$4.8 \cdot 10^{3}$	$3.0 \cdot 10^{4}$	$2.4 \cdot 10^{5}$	$5.3 \cdot 10^{0}$	$3.3 \cdot 10^{0}$	$1.2 \cdot 10^{1}$	$7.0 \cdot 10^{1}$	$4.7 \cdot 10^{2}$
B97-D	$6.6 \cdot 10^{1}$	$4.4 \cdot 10^{2}$	$2.5 \cdot 10^{3}$	$2.1 \cdot 10^{4}$	$1.3 \cdot 10^{5}$	$7.5 \cdot 10^{0}$	$5.5 \cdot 10^{0}$	$1.8 \cdot 10^{1}$	$1.1 \cdot 10^{2}$	$6.0 \cdot 10^{2}$
wB97	$1.3 \cdot 10^{2}$	$7.1 \cdot 10^{2}$	$4.8 \cdot 10^{3}$	$3.9 \cdot 10^{4}$	$3.5 \cdot 10^{5}$	$2.0 \cdot 10^{1}$	$7.0 \cdot 10^{0}$	$3.6 \cdot 10^{1}$	$1.5 \cdot 10^{2}$	$8.5 \cdot 10^{2}$
wB97X	$5.2 \cdot 10^{1}$	$4.1 \cdot 10^{2}$	$2.7 \cdot 10^{3}$	$3.5 \cdot 10^{4}$	$4.4 \cdot 10^{5}$	$1.9 \cdot 10^{1}$	$1.4 \cdot 10^{1}$	$3.0 \cdot 10^{1}$	$1.1 \cdot 10^{2}$	$7.5 \cdot 10^{2}$
wB97X-D	$1.4 \cdot 10^{2}$	$6.9 \cdot 10^{2}$	$6.2 \cdot 10^{3}$	$5.6 \cdot 10^{4}$	$8.0 \cdot 10^{5}$	$1.5 \cdot 10^{1}$	$1.3 \cdot 10^{1}$	$2.4 \cdot 10^{1}$	$8.1 \cdot 10^{1}$	$7.0 \cdot 10^{2}$
wB97X-D3	$6.7 \cdot 10^{1}$	$4.5 \cdot 10^{2}$	$3.9 \cdot 10^{3}$	$3.8 \cdot 10^{4}$	$5.5 \cdot 10^{5}$	$1.6 \cdot 10^{1}$	$1.1 \cdot 10^{1}$	$2.6 \cdot 10^{1}$	$9.1 \cdot 10^{1}$	$7.1 \cdot 10^{2}$
wB97M-V	$3.3 \cdot 10^{1}$	$1.3 \cdot 10^{2}$	$7.2 \cdot 10^{2}$	$4.6 \cdot 10^{3}$	$3.2 \cdot 10^{4}$	$1.4 \cdot 10^{1}$	$1.1 \cdot 10^{1}$	$3.4 \cdot 10^{1}$	$1.5 \cdot 10^{2}$	$8.7 \cdot 10^{2}$
wB97X-V	$1.5 \cdot 10^{1}$	$6.5 \cdot 10^{1}$	$4.7 \cdot 10^{2}$	$2.4 \cdot 10^{3}$	$2.1 \cdot 10^{4}$	$8.5 \cdot 10^{0}$	$2.8 \cdot 10^{0}$	$1.3 \cdot 10^{1}$	$8.0 \cdot 10^{1}$	$5.0 \cdot 10^{2}$
B97M-V	$3.6 \cdot 10^{1}$	$4.5 \cdot 10^{1}$	$2.7 \cdot 10^{2}$	$1.6 \cdot 10^{3}$	$1.4 \cdot 10^{4}$	$1.9 \cdot 10^{1}$	$1.3 \cdot 10^{1}$	$6.5 \cdot 10^{1}$	$3.4 \cdot 10^{2}$	$1.6 \cdot 10^{3}$
B1PW91	$4.0 \cdot 10^{0}$	$3.8 \cdot 10^{0}$	$2.2 \cdot 10^{1}$	$1.6 \cdot 10^{2}$	$2.2 \cdot 10^{3}$	$1.0 \cdot 10^{1}$	$7.4 \cdot 10^{0}$	$5.1 \cdot 10^{1}$	$2.7 \cdot 10^{2}$	$1.6 \cdot 10^{3}$
PW91	$7.5 \cdot 10^{0}$	$2.2 \cdot 10^{0}$	$1.8 \cdot 10^{1}$	$2.3 \cdot 10^{2}$	$3.3 \cdot 10^{3}$	$6.9 \cdot 10^{0}$	$2.9 \cdot 10^{1}$	$1.0 \cdot 10^{2}$	$1.2 \cdot 10^{3}$	$1.9 \cdot 10^{4}$
mPW91	$6.5 \cdot 10^{0}$	$3.5 \cdot 10^{0}$	$1.7 \cdot 10^{1}$	$1.9 \cdot 10^{2}$	$2.7 \cdot 10^{3}$	$8.2 \cdot 10^{0}$	$2.9 \cdot 10^{1}$	$1.3 \cdot 10^{2}$	$1.2 \cdot 10^{3}$	$1.8 \cdot 10^{4}$
VSXC	$8.8 \cdot 10^{1}$	$1.9 \cdot 10^{2}$	$6.1 \cdot 10^{2}$	$2.8 \cdot 10^{3}$	$1.2 \cdot 10^{4}$	$1.7 \cdot 10^{1}$	$7.1 \cdot 10^{0}$	$2.8 \cdot 10^{1}$	$1.5 \cdot 10^{2}$	$7.3 \cdot 10^{2}$
SCAN	$1.3 \cdot 10^{2}$	$4.2 \cdot 10^{2}$	$2.9 \cdot 10^{3}$	$4.2 \cdot 10^{4}$	$1.1 \cdot 10^{6}$	$8.2 \cdot 10^{2}$	$2.3 \cdot 10^{3}$	$1.8 \cdot 10^{4}$	$2.1 \cdot 10^{5}$	$3.9 \cdot 10^{6}$
SCAN0	$1.7 \cdot 10^{2}$	$5.8 \cdot 10^{2}$	$3.3 \cdot 10^{3}$	$4.5 \cdot 10^{4}$	$1.0 \cdot 10^{6}$	$5.0 \cdot 10^{2}$	$2.5 \cdot 10^{3}$	$1.4 \cdot 10^{4}$	$1.6 \cdot 10^{5}$	$3.3 \cdot 10^{6}$
M06	$1.3 \cdot 10^{2}$	$3.9 \cdot 10^{2}$	$3.2 \cdot 10^{3}$	$2.0 \cdot 10^{4}$	$1.7 \cdot 10^{5}$	$1.7 \cdot 10^{2}$	$1.6 \cdot 10^{2}$	$5.7 \cdot 10^{2}$	$2.9 \cdot 10^{3}$	$2.4 \cdot 10^{4}$
M06-L	$2.1 \cdot 10^{2}$	$7.8 \cdot 10^{2}$	$5.5 \cdot 10^{3}$	$4.1 \cdot 10^{4}$	$3.2 \cdot 10^{5}$	$1.4 \cdot 10^{2}$	$4.0 \cdot 10^{2}$	$5.8 \cdot 10^{2}$	$5.2 \cdot 10^{3}$	$4.5 \cdot 10^{4}$
M06-HF	$1.5 \cdot 10^{2}$	$7.5 \cdot 10^{2}$	$6.3 \cdot 10^{3}$	$3.7 \cdot 10^{4}$	$3.2 \cdot 10^{5}$	$5.0 \cdot 10^{2}$	$1.6 \cdot 10^{2}$	$1.2 \cdot 10^{3}$	$5.5 \cdot 10^{3}$	$6.0 \cdot 10^{4}$
M06-2X	$6.6 \cdot 10^{1}$	$3.0 \cdot 10^{2}$	$2.7 \cdot 10^{3}$	$1.5 \cdot 10^{4}$	$1.4 \cdot 10^{5}$	$1.3 \cdot 10^{2}$	$8.2 \cdot 10^{1}$	$4.1 \cdot 10^{2}$	$2.3 \cdot 10^{3}$	$2.6 \cdot 10^{4}$
M11	$9.9 \cdot 10^{1}$	$5.4 \cdot 10^{1}$	$2.0 \cdot 10^{2}$	$1.3 \cdot 10^{3}$	$6.8 \cdot 10^{3}$	$1.1 \cdot 10^{2}$	$1.3 \cdot 10^{2}$	$3.1 \cdot 10^{2}$	$2.8 \cdot 10^{3}$	$1.5 \cdot 10^{4}$
M11-L	$2.5 \cdot 10^{2}$	$3.0 \cdot 10^{1}$	$2.2 \cdot 10^{2}$	$1.7 \cdot 10^{3}$	$8.2 \cdot 10^{3}$	$2.5 \cdot 10^{2}$	$1.3 \cdot 10^{2}$	$6.5 \cdot 10^{2}$	$4.8 \cdot 10^{3}$	$3.4 \cdot 10^{4}$
SOGGA11	$1.2 \cdot 10^{2}$	$4.2 \cdot 10^{2}$	$8.4 \cdot 10^{2}$	$8.3 \cdot 10^{3}$	$4.6 \cdot 10^{4}$	$2.7 \cdot 10^{1}$	$9.2 \cdot 10^{1}$	$2.4 \cdot 10^{2}$	$1.1 \cdot 10^{3}$	$4.6 \cdot 10^{3}$
SOGGA11-	$1.4 \cdot 10^{2}$	$1.3 \cdot 10^{2}$	$2.3 \cdot 10^{2}$	$6.8 \cdot 10^{2}$	$1.0 \cdot 10^{4}$	$4.9 \cdot 10^{0}$	$6.1 \cdot 10^{0}$	$1.9 \cdot 10^{1}$	$1.1 \cdot 10^{2}$	$5.7 \cdot 10^{2}$
N12	$2.2 \cdot 10^{2}$	$8.4 \cdot 10^{2}$	$5.7 \cdot 10^{3}$	$5.8 \cdot 10^{4}$	$7.9 \cdot 10^{5}$	$3.3 \cdot 10^{1}$	$1.1 \cdot 10^{1}$	$5.0 \cdot 10^{1}$	$2.6 \cdot 10^{2}$	$1.4 \cdot 10^{3}$
N12-SX	$4.1 \cdot 10^{1}$	$3.6 \cdot 10^{2}$	$2.3 \cdot 10^{3}$	$1.7 \cdot 10^{4}$	$1.2 \cdot 10^{5}$	$3.4 \cdot 10^{1}$	$7.9 \cdot 10^{0}$	$5.8 \cdot 10^{1}$	$3.1 \cdot 10^{2}$	$2.4 \cdot 10^{3}$
MN12-L	$3.5 \cdot 10^{1}$	$7.2 \cdot 10^{1}$	$3.5 \cdot 10^{2}$	$2.0 \cdot 10^{3}$	$1.1 \cdot 10^{4}$	$5.9 \cdot 10^{1}$	$8.9 \cdot 10^{1}$	$4.0 \cdot 10^{2}$	$2.3 \cdot 10^{3}$	$1.7 \cdot 10^{4}$
MN12-SX	$7.6 \cdot 10^{1}$	$9.4 \cdot 10^{1}$	$3.3 \cdot 10^{2}$	$2.0 \cdot 10^{3}$	$9.1 \cdot 10^{3}$	$7.6 \cdot 10^{1}$	$4.5 \cdot 10^{1}$	$2.0 \cdot 10^{2}$	$1.3 \cdot 10^{3}$	$1.1 \cdot 10^{4}$
MN15	$4.6 \cdot 10^{1}$	$6.6 \cdot 10^{1}$	$3.2 \cdot 10^{2}$	$2.0 \cdot 10^{3}$	$1.0 \cdot 10^{4}$	$1.8 \cdot 10^{1}$	$7.5 \cdot 10^{1}$	$2.1 \cdot 10^{2}$	$1.4 \cdot 10^{3}$	$7.3 \cdot 10^{3}$
MN15-L	$5.0 \cdot 10^{1}$	$6.1 \cdot 10^{1}$	$1.6 \cdot 10^{2}$	$9.5 \cdot 10^{2}$	$2.6 \cdot 10^{3}$	$9.3 \cdot 10^{1}$	$3.4 \cdot 10^{1}$	$1.4 \cdot 10^{2}$	$5.8 \cdot 10^{2}$	$4.2 \cdot 10^{3}$

Table S12: RMSE values for various energy derivatives obtained with the different functionals used in this work combined with the (250, 974) integration grid for the HCN · HCl system.

DFA	$\mathrm{d}^nE_c/\mathrm{d}\xi^n$ [$\times 10^{-8}$] 0	1	2	3	4	$\mathrm{d}^nE_x/\mathrm{d}\xi^n$ [$\times 10^{-8}$] 0	1	2	3	4
SVWN5	$2.8 \cdot 10^{-3}$	$1.8 \cdot 10^{-3}$	$4.1 \cdot 10^{-2}$	$3.4 \cdot 10^{-1}$	$9.7 \cdot 10^{0}$	$5.1 \cdot 10^{-3}$	$1.6 \cdot 10^{-2}$	$2.9 \cdot 10^{-2}$	$7.0 \cdot 10^{-1}$	$1.2 \cdot 10^{1}$
SPW92	$2.5 \cdot 10^{-3}$	$2.5 \cdot 10^{-3}$	$2.9 \cdot 10^{-2}$	$4.2 \cdot 10^{-1}$	$1.1 \cdot 10^{1}$	$4.8 \cdot 10^{-3}$	$1.4 \cdot 10^{-2}$	$3.9 \cdot 10^{-2}$	$6.5 \cdot 10^{-1}$	$1.0 \cdot 10^{1}$
BHH	$3.0 \cdot 10^{-3}$	$3.0 \cdot 10^{-3}$	$3.6 \cdot 10^{-2}$	$3.8 \cdot 10^{-1}$	$9.1 \cdot 10^{0}$	$4.7 \cdot 10^{-3}$	$1.4 \cdot 10^{-2}$	$2.7 \cdot 10^{-2}$	$3.4 \cdot 10^{-1}$	$8.1 \cdot 10^{0}$
BLYP	$3.2 \cdot 10^{-3}$	$1.8 \cdot 10^{-3}$	$3.8 \cdot 10^{-2}$	$4.9 \cdot 10^{-1}$	$8.0 \cdot 10^{0}$	$8.6 \cdot 10^{-3}$	$1.9 \cdot 10^{-2}$	$2.1 \cdot 10^{-1}$	$2.6 \cdot 10^{0}$	$2.5 \cdot 10^{1}$
B1LYP	$2.7 \cdot 10^{-3}$	$1.2 \cdot 10^{-3}$	$3.3 \cdot 10^{-2}$	$7.2 \cdot 10^{-1}$	$1.2 \cdot 10^{1}$	$5.5 \cdot 10^{-3}$	$1.5 \cdot 10^{-2}$	$1.6 \cdot 10^{-1}$	$2.1 \cdot 10^{0}$	$2.4 \cdot 10^{1}$
B3LYP	$3.6 \cdot 10^{-3}$	$4.6 \cdot 10^{-3}$	$2.5 \cdot 10^{-2}$	$4.5 \cdot 10^{-1}$	$6.8 \cdot 10^{0}$	$5.1 \cdot 10^{-3}$	$2.2 \cdot 10^{-2}$	$2.1 \cdot 10^{-1}$	$2.2 \cdot 10^{0}$	$2.3 \cdot 10^{1}$
BHHLYP	$3.2 \cdot 10^{-3}$	$4.5 \cdot 10^{-3}$	$2.4 \cdot 10^{-2}$	$5.7 \cdot 10^{-1}$	$1.0 \cdot 10^{1}$	$6.1 \cdot 10^{-3}$	$1.1 \cdot 10^{-2}$	$1.4 \cdot 10^{-1}$	$1.7 \cdot 10^{0}$	$1.8 \cdot 10^{1}$
LC-BLYP	$3.3 \cdot 10^{-3}$	$1.9 \cdot 10^{-3}$	$3.7 \cdot 10^{-2}$	$6.9 \cdot 10^{-1}$	$1.2 \cdot 10^{1}$	$6.4 \cdot 10^{-3}$	$1.8 \cdot 10^{-2}$	$4.9 \cdot 10^{-2}$	$5.4 \cdot 10^{-1}$	$1.2 \cdot 10^{1}$
CAMB3LYP	$3.2 \cdot 10^{-3}$	$1.8 \cdot 10^{-3}$	$1.7 \cdot 10^{-2}$	$3.8 \cdot 10^{-1}$	$7.1 \cdot 10^{0}$	$3.6 \cdot 10^{-3}$	$1.6 \cdot 10^{-2}$	$6.7 \cdot 10^{-2}$	$1.0 \cdot 10^{0}$	$1.2 \cdot 10^{1}$
PBE	$4.5 \cdot 10^{-3}$	$5.0 \cdot 10^{-3}$	$4.6 \cdot 10^{-2}$	$1.3 \cdot 10^{0}$	$3.1 \cdot 10^{1}$	$4.2 \cdot 10^{-3}$	$2.8 \cdot 10^{-1}$	$6.7 \cdot 10^{-2}$	$7.1 \cdot 10^{-1}$	$2.3 \cdot 10^{1}$
PBE0	$3.9 \cdot 10^{-3}$	$5.0 \cdot 10^{-3}$	$6.0 \cdot 10^{-2}$	$1.4 \cdot 10^{0}$	$2.9 \cdot 10^{1}$	$4.3 \cdot 10^{-3}$	$1.2 \cdot 10^{-2}$	$4.8 \cdot 10^{-2}$	$1.4 \cdot 10^{0}$	$2.1 \cdot 10^{1}$
PBE50	$3.9 \cdot 10^{-3}$	$4.9 \cdot 10^{-3}$	$3.3 \cdot 10^{-2}$	$1.4 \cdot 10^{0}$	$3.6 \cdot 10^{1}$	$8.1 \cdot 10^{-3}$	$6.0 \cdot 10^{-3}$	$5.2 \cdot 10^{-2}$	$8.2 \cdot 10^{-1}$	$1.9 \cdot 10^{1}$
LC-wPBE	$3.7 \cdot 10^{-3}$	$5.3 \cdot 10^{-3}$	$5.1 \cdot 10^{-2}$	$1.3 \cdot 10^{0}$	$3.0 \cdot 10^{1}$	$5.9 \cdot 10^{-3}$	$2.1 \cdot 10^{-2}$	$8.9 \cdot 10^{-2}$	$2.3 \cdot 10^{0}$	$4.7 \cdot 10^{1}$
PBE-GXLY	$5.6 \cdot 10^{-3}$	$1.1 \cdot 10^{-3}$	$1.2 \cdot 10^{-2}$	$2.4 \cdot 10^{-1}$	$1.0 \cdot 10^{1}$	$2.3 \cdot 10^{3}$	$2.5 \cdot 10^{3}$	$4.1 \cdot 10^{4}$	$1.0 \cdot 10^{6}$	$2.4 \cdot 10^{7}$
TPSS	$3.4 \cdot 10^{-3}$	$6.9 \cdot 10^{-3}$	$5.1 \cdot 10^{-2}$	$1.4 \cdot 10^{0}$	$3.1 \cdot 10^{1}$	$1.2 \cdot 10^{-2}$	$1.5 \cdot 10^{-1}$	$2.1 \cdot 10^{0}$	$3.3 \cdot 10^{1}$	$5.2 \cdot 10^{2}$
RevTPSS	$4.2 \cdot 10^{-3}$	$6.8 \cdot 10^{-3}$	$7.0 \cdot 10^{-2}$	$1.6 \cdot 10^{0}$	$3.4 \cdot 10^{1}$	$9.2 \cdot 10^{-2}$	$3.0 \cdot 10^{-2}$	$9.8 \cdot 10^{-2}$	$4.9 \cdot 10^{0}$	$1.2 \cdot 10^{2}$
TPSSh	$3.6 \cdot 10^{-3}$	$6.0 \cdot 10^{-3}$	$3.1 \cdot 10^{-2}$	$2.8 \cdot 10^{0}$	$2.8 \cdot 10^{1}$	$1.3 \cdot 10^{-2}$	$1.2 \cdot 10^{-1}$	$1.9 \cdot 10^{0}$	$2.8 \cdot 10^{1}$	$4.8 \cdot 10^{2}$
B97	$1.9 \cdot 10^{0}$	$2.0 \cdot 10^{1}$	$2.1 \cdot 10^{2}$	$2.4 \cdot 10^{3}$	$2.8 \cdot 10^{4}$	$4.1 \cdot 10^{-3}$	$1.1 \cdot 10^{-2}$	$6.4 \cdot 10^{-2}$	$8.8 \cdot 10^{-1}$	$1.9 \cdot 10^{1}$
B97-D	$1.3 \cdot 10^{0}$	$1.4 \cdot 10^{1}$	$1.4 \cdot 10^{2}$	$1.5 \cdot 10^{3}$	$1.7 \cdot 10^{4}$	$1.2 \cdot 10^{-2}$	$2.2 \cdot 10^{-1}$	$1.4 \cdot 10^{0}$	$2.2 \cdot 10^{1}$	$3.5 \cdot 10^{2}$
wB97	$1.4 \cdot 10^{0}$	$2.4 \cdot 10^{1}$	$4.3 \cdot 10^{2}$	$8.7 \cdot 10^{3}$	$1.6 \cdot 10^{5}$	$1.0 \cdot 10^{-2}$	$1.6 \cdot 10^{-2}$	$8.5 \cdot 10^{-2}$	$4.1 \cdot 10^{0}$	$9.3 \cdot 10^{1}$
wB97X	$3.0 \cdot 10^{0}$	$3.9 \cdot 10^{1}$	$6.3 \cdot 10^{2}$	$1.2 \cdot 10^{4}$	$2.2 \cdot 10^{5}$	$6.3 \cdot 10^{-3}$	$1.2 \cdot 10^{-2}$	$4.9 \cdot 10^{-2}$	$1.9 \cdot 10^{0}$	$4.7 \cdot 10^{1}$
wB97X-D	$5.4 \cdot 10^{0}$	$7.2 \cdot 10^{1}$	$1.1 \cdot 10^{3}$	$1.9 \cdot 10^{4}$	$3.7 \cdot 10^{5}$	$4.8 \cdot 10^{-3}$	$1.2 \cdot 10^{-2}$	$2.9 \cdot 10^{-2}$	$1.9 \cdot 10^{0}$	$4.7 \cdot 10^{1}$
wB97X-D3	$3.5 \cdot 10^{0}$	$4.7 \cdot 10^{1}$	$7.4 \cdot 10^{2}$	$1.3 \cdot 10^{4}$	$2.6 \cdot 10^{5}$	$6.3 \cdot 10^{-3}$	$1.1 \cdot 10^{-2}$	$4.6 \cdot 10^{-2}$	$1.7 \cdot 10^{0}$	$4.2 \cdot 10^{1}$
wB97M-V	$2.0 \cdot 10^{-1}$	$2.1 \cdot 10^{0}$	$2.4 \cdot 10^{1}$	$2.8 \cdot 10^{2}$	$3.6 \cdot 10^{3}$	$4.7 \cdot 10^{-3}$	$9.2 \cdot 10^{-3}$	$4.9 \cdot 10^{-2}$	$5.9 \cdot 10^{-1}$	$2.0 \cdot 10^{1}$
wB97X-V	$9.0 \cdot 10^{-2}$	$8.5 \cdot 10^{-1}$	$9.4 \cdot 10^{0}$	$9.2 \cdot 10^{1}$	$1.0 \cdot 10^{3}$	$8.4 \cdot 10^{-3}$	$7.8 \cdot 10^{-3}$	$5.4 \cdot 10^{-2}$	$1.3 \cdot 10^{0}$	$2.8 \cdot 10^{1}$
B97M-V	$1.3 \cdot 10^{-1}$	$1.3 \cdot 10^{0}$	$1.6 \cdot 10^{1}$	$1.8 \cdot 10^{2}$	$2.3 \cdot 10^{3}$	$1.6 \cdot 10^{-2}$	$1.2 \cdot 10^{-1}$	$1.5 \cdot 10^{0}$	$2.1 \cdot 10^{1}$	$3.3 \cdot 10^{2}$
B1PW91	$1.7 \cdot 10^{-2}$	$3.2 \cdot 10^{-1}$	$5.7 \cdot 10^{0}$	$1.1 \cdot 10^{2}$	$2.1 \cdot 10^{3}$	$4.6 \cdot 10^{-3}$	$1.6 \cdot 10^{-2}$	$1.1 \cdot 10^{-1}$	$1.7 \cdot 10^{0}$	$2.0 \cdot 10^{1}$
PW91	$1.0 \cdot 10^{-2}$	$2.3 \cdot 10^{-1}$	$4.1 \cdot 10^{0}$	$8.5 \cdot 10^{1}$	$1.9 \cdot 10^{3}$	$5.8 \cdot 10^{-2}$	$1.1 \cdot 10^{0}$	$2.4 \cdot 10^{1}$	$5.0 \cdot 10^{2}$	$1.1 \cdot 10^{4}$
mPW91	$1.3 \cdot 10^{-2}$	$2.5 \cdot 10^{-1}$	$4.4 \cdot 10^{0}$	$8.8 \cdot 10^{1}$	$1.9 \cdot 10^{3}$	$7.2 \cdot 10^{-2}$	$1.4 \cdot 10^{0}$	$2.8 \cdot 10^{1}$	$5.6 \cdot 10^{2}$	$1.1 \cdot 10^{4}$
VSXC	$3.3 \cdot 10^{-2}$	$4.7 \cdot 10^{-1}$	$5.3 \cdot 10^{0}$	$7.6 \cdot 10^{1}$	$9.0 \cdot 10^{2}$	$1.2 \cdot 10^{-1}$	$1.7 \cdot 10^{-1}$	$2.5 \cdot 10^{0}$	$3.0 \cdot 10^{1}$	$4.6 \cdot 10^{2}$
SCAN	$8.6 \cdot 10^{0}$	$5.4 \cdot 10^{1}$	$9.2 \cdot 10^{2}$	$1.9 \cdot 10^{4}$	$5.2 \cdot 10^{5}$	$4.5 \cdot 10^{1}$	$2.5 \cdot 10^{2}$	$4.2 \cdot 10^{3}$	$8.7 \cdot 10^{4}$	$2.2 \cdot 10^{6}$
SCAN0	$8.1 \cdot 10^{0}$	$4.9 \cdot 10^{1}$	$7.7 \cdot 10^{2}$	$1.8 \cdot 10^{4}$	$5.1 \cdot 10^{5}$	$1.2 \cdot 10^{1}$	$1.7 \cdot 10^{2}$	$2.9 \cdot 10^{3}$	$5.9 \cdot 10^{4}$	$1.4 \cdot 10^{6}$
M06	$8.6 \cdot 10^{-1}$	$8.5 \cdot 10^{0}$	$9.8 \cdot 10^{1}$	$9.6 \cdot 10^{2}$	$1.1 \cdot 10^{4}$	$2.0 \cdot 10^{-1}$	$1.8 \cdot 10^{0}$	$2.9 \cdot 10^{1}$	$4.2 \cdot 10^{2}$	$7.3 \cdot 10^{3}$
M06-L	$1.0 \cdot 10^{0}$	$1.2 \cdot 10^{1}$	$1.2 \cdot 10^{2}$	$1.5 \cdot 10^{3}$	$1.5 \cdot 10^{4}$	$4.2 \cdot 10^{-1}$	$5.6 \cdot 10^{0}$	$9.4 \cdot 10^{1}$	$1.4 \cdot 10^{3}$	$2.4 \cdot 10^{4}$
M06-HF	$1.6 \cdot 10^{0}$	$1.7 \cdot 10^{1}$	$1.6 \cdot 10^{2}$	$1.8 \cdot 10^{3}$	$1.6 \cdot 10^{4}$	$8.6 \cdot 10^{-1}$	$1.2 \cdot 10^{1}$	$1.8 \cdot 10^{2}$	$2.6 \cdot 10^{3}$	$3.9 \cdot 10^{4}$
M06-2X	$9.8 \cdot 10^{-1}$	$1.1 \cdot 10^{1}$	$1.1 \cdot 10^{2}$	$1.2 \cdot 10^{3}$	$1.2 \cdot 10^{4}$	$4.2 \cdot 10^{-1}$	$5.1 \cdot 10^{0}$	$7.8 \cdot 10^{1}$	$1.2 \cdot 10^{3}$	$1.7 \cdot 10^{4}$
M11	$3.8 \cdot 10^{-2}$	$5.9 \cdot 10^{-1}$	$8.6 \cdot 10^{0}$	$1.4 \cdot 10^{2}$	$2.1 \cdot 10^{3}$	$5.1 \cdot 10^{-1}$	$1.8 \cdot 10^{1}$	$4.4 \cdot 10^{1}$	$6.9 \cdot 10^{2}$	$1.0 \cdot 10^{4}$
M11-L	$1.1 \cdot 10^{-1}$	$1.6 \cdot 10^{0}$	$2.3 \cdot 10^{1}$	$3.5 \cdot 10^{2}$	$5.0 \cdot 10^{3}$	$4.7 \cdot 10^{-1}$	$6.9 \cdot 10^{0}$	$9.8 \cdot 10^{1}$	$1.5 \cdot 10^{3}$	$2.2 \cdot 10^{4}$
SOGGA11	$4.6 \cdot 10^{-1}$	$4.5 \cdot 10^{0}$	$4.0 \cdot 10^{1}$	$3.8 \cdot 10^{2}$	$3.3 \cdot 10^{3}$	$1.2 \cdot 10^{-2}$	$1.3 \cdot 10^{-1}$	$1.4 \cdot 10^{0}$	$1.7 \cdot 10^{1}$	$2.2 \cdot 10^{2}$
SOGGA11-	$1.2 \cdot 10^{-1}$	$1.4 \cdot 10^{0}$	$2.0 \cdot 10^{1}$	$3.0 \cdot 10^{2}$	$4.3 \cdot 10^{3}$	$5.7 \cdot 10^{-3}$	$1.2 \cdot 10^{-2}$	$5.1 \cdot 10^{-2}$	$1.8 \cdot 10^{0}$	$4.0 \cdot 10^{1}$
N12	$4.7 \cdot 10^{0}$	$5.8 \cdot 10^{1}$	$8.0 \cdot 10^{2}$	$1.5 \cdot 10^{4}$	$2.8 \cdot 10^{5}$	$2.4 \cdot 10^{-2}$	$4.6 \cdot 10^{-1}$	$4.5 \cdot 10^{0}$	$7.6 \cdot 10^{1}$	$1.1 \cdot 10^{3}$
N12-SX	$1.2 \cdot 10^{0}$	$1.4 \cdot 10^{1}$	$1.4 \cdot 10^{2}$	$2.0 \cdot 10^{3}$	$2.9 \cdot 10^{4}$	$3.9 \cdot 10^{-2}$	$2.5 \cdot 10^{-1}$	$3.7 \cdot 10^{0}$	$5.8 \cdot 10^{1}$	$9.2 \cdot 10^{2}$
MN12-L	$2.4 \cdot 10^{-2}$	$3.4 \cdot 10^{-1}$	$4.5 \cdot 10^{0}$	$6.5 \cdot 10^{1}$	$8.8 \cdot 10^{2}$	$1.3 \cdot 10^{-1}$	$1.4 \cdot 10^{0}$	$2.0 \cdot 10^{1}$	$2.9 \cdot 10^{2}$	$4.2 \cdot 10^{3}$
MN12-SX	$8.6 \cdot 10^{-2}$	$1.5 \cdot 10^{0}$	$1.8 \cdot 10^{1}$	$2.4 \cdot 10^{2}$	$3.6 \cdot 10^{3}$	$1.4 \cdot 10^{-1}$	$1.9 \cdot 10^{0}$	$2.4 \cdot 10^{1}$	$3.4 \cdot 10^{2}$	$4.8 \cdot 10^{3}$
MN15	$2.0 \cdot 10^{-2}$	$2.5 \cdot 10^{-1}$	$3.4 \cdot 10^{0}$	$4.9 \cdot 10^{1}$	$7.3 \cdot 10^{2}$	$3.5 \cdot 10^{-2}$	$4.1 \cdot 10^{-2}$	$1.5 \cdot 10^{-1}$	$3.4 \cdot 10^{0}$	$6.6 \cdot 10^{1}$
MN15-L	$1.6 \cdot 10^{-2}$	$1.8 \cdot 10^{-1}$	$2.4 \cdot 10^{0}$	$2.9 \cdot 10^{1}$	$3.8 \cdot 10^{2}$	$1.6 \cdot 10^{-2}$	$1.9 \cdot 10^{-1}$	$2.4 \cdot 10^{0}$	$2.8 \cdot 10^{1}$	$4.3 \cdot 10^{2}$

Table S13: RMSE values for various energy derivatives obtained with the different functionals used in this work combined with the (99, 590) integration grid for the HCN · BrF system.

DFA	$\mathrm{d}^nE_c/\mathrm{d}\xi^n$ [$\times 10^{-8}$]					$\mathrm{d}^nE_x/\mathrm{d}\xi^n$ [$\times 10^{-8}$]				
	0	1	2	3	4	0	1	2	3	4
SVWN5	$6.9 \cdot 10^{0}$	$2.4 \cdot 10^{1}$	$1.0 \cdot 10^{2}$	$3.0 \cdot 10^{2}$	$1.3 \cdot 10^{3}$	$1.2 \cdot 10^{2}$	$5.7 \cdot 10^{2}$	$1.7 \cdot 10^{3}$	$6.9 \cdot 10^{3}$	$2.1 \cdot 10^{4}$
SPW92	$6.9 \cdot 10^{0}$	$2.4 \cdot 10^{1}$	$1.0 \cdot 10^{2}$	$3.0 \cdot 10^{2}$	$1.3 \cdot 10^{3}$	$1.2 \cdot 10^{2}$	$5.7 \cdot 10^{2}$	$1.7 \cdot 10^{3}$	$6.9 \cdot 10^{3}$	$2.1 \cdot 10^{4}$
BHH	$6.0 \cdot 10^{0}$	$2.6 \cdot 10^{1}$	$1.3 \cdot 10^{2}$	$4.5 \cdot 10^{2}$	$2.0 \cdot 10^{3}$	$6.3 \cdot 10^{1}$	$2.8 \cdot 10^{2}$	$1.0 \cdot 10^{3}$	$3.6 \cdot 10^{3}$	$1.5 \cdot 10^{4}$
BLYP	$7.1 \cdot 10^{0}$	$3.5 \cdot 10^{1}$	$1.2 \cdot 10^{2}$	$5.6 \cdot 10^{2}$	$1.9 \cdot 10^{3}$	$1.8 \cdot 10^{2}$	$4.2 \cdot 10^{2}$	$1.7 \cdot 10^{3}$	$6.7 \cdot 10^{3}$	$2.2 \cdot 10^{4}$
B1LYP	$7.9 \cdot 10^{0}$	$4.0 \cdot 10^{1}$	$1.1 \cdot 10^{2}$	$5.2 \cdot 10^{2}$	$1.6 \cdot 10^{3}$	$1.5 \cdot 10^{2}$	$4.1 \cdot 10^{2}$	$1.0 \cdot 10^{3}$	$4.6 \cdot 10^{3}$	$1.2 \cdot 10^{4}$
B3LYP	$8.9 \cdot 10^{0}$	$3.0 \cdot 10^{1}$	$1.4 \cdot 10^{2}$	$4.2 \cdot 10^{2}$	$1.9 \cdot 10^{3}$	$8.7 \cdot 10^{1}$	$4.2 \cdot 10^{2}$	$1.2 \cdot 10^{3}$	$4.7 \cdot 10^{3}$	$1.5 \cdot 10^{4}$
BHHLYP	$1.2 \cdot 10^{1}$	$2.9 \cdot 10^{1}$	$1.2 \cdot 10^{2}$	$3.7 \cdot 10^{2}$	$1.7 \cdot 10^{3}$	$9.1 \cdot 10^{1}$	$1.8 \cdot 10^{2}$	$8.5 \cdot 10^{2}$	$2.0 \cdot 10^{3}$	$1.0 \cdot 10^{4}$
LC-BLYP	$6.6 \cdot 10^{0}$	$3.3 \cdot 10^{1}$	$1.2 \cdot 10^{2}$	$4.5 \cdot 10^{2}$	$1.8 \cdot 10^{3}$	$1.1 \cdot 10^{2}$	$4.1 \cdot 10^{2}$	$1.2 \cdot 10^{3}$	$5.7 \cdot 10^{3}$	$1.4 \cdot 10^{4}$
CAMB3LYP	$7.0 \cdot 10^{0}$	$3.6 \cdot 10^{1}$	$1.1 \cdot 10^{2}$	$4.6 \cdot 10^{2}$	$1.6 \cdot 10^{3}$	$1.4 \cdot 10^{2}$	$4.0 \cdot 10^{2}$	$9.9 \cdot 10^{2}$	$4.9 \cdot 10^{3}$	$1.2 \cdot 10^{4}$
PBE	$6.2 \cdot 10^{0}$	$3.7 \cdot 10^{1}$	$1.8 \cdot 10^{2}$	$7.2 \cdot 10^{2}$	$3.2 \cdot 10^{3}$	$1.3 \cdot 10^{2}$	$6.7 \cdot 10^{2}$	$1.5 \cdot 10^{3}$	$7.5 \cdot 10^{3}$	$2.3 \cdot 10^{4}$
PBE0	$2.4 \cdot 10^{1}$	$3.2 \cdot 10^{1}$	$1.3 \cdot 10^{2}$	$4.4 \cdot 10^{2}$	$2.1 \cdot 10^{3}$	$9.6 \cdot 10^{1}$	$3.7 \cdot 10^{2}$	$1.0 \cdot 10^{3}$	$4.9 \cdot 10^{3}$	$1.5 \cdot 10^{4}$
PBE50	$1.3 \cdot 10^{1}$	$2.7 \cdot 10^{1}$	$6.0 \cdot 10^{1}$	$3.0 \cdot 10^{2}$	$1.0 \cdot 10^{3}$	$5.4 \cdot 10^{1}$	$1.6 \cdot 10^{2}$	$7.5 \cdot 10^{2}$	$2.4 \cdot 10^{3}$	$9.4 \cdot 10^{3}$
LC-wPBE	$1.4 \cdot 10^{1}$	$2.0 \cdot 10^{1}$	$6.3 \cdot 10^{1}$	$3.2 \cdot 10^{2}$	$1.2 \cdot 10^{3}$	$9.2 \cdot 10^{1}$	$3.8 \cdot 10^{2}$	$1.2 \cdot 10^{3}$	$4.9 \cdot 10^{3}$	$1.6 \cdot 10^{4}$
TPSS	$1.2 \cdot 10^{1}$	$2.9 \cdot 10^{1}$	$1.1 \cdot 10^{2}$	$4.6 \cdot 10^{2}$	$2.2 \cdot 10^{3}$	$2.9 \cdot 10^{2}$	$5.9 \cdot 10^{2}$	$2.8 \cdot 10^{3}$	$8.9 \cdot 10^{3}$	$4.1 \cdot 10^{4}$
RevTPSS	$1.4 \cdot 10^{1}$	$3.2 \cdot 10^{1}$	$1.1 \cdot 10^{2}$	$4.8 \cdot 10^{2}$	$2.1 \cdot 10^{3}$	$3.0 \cdot 10^{2}$	$5.4 \cdot 10^{2}$	$2.4 \cdot 10^{3}$	$7.7 \cdot 10^{3}$	$3.7 \cdot 10^{4}$
TPSSh	$2.0 \cdot 10^{1}$	$3.2 \cdot 10^{1}$	$1.0 \cdot 10^{2}$	$4.8 \cdot 10^{2}$	$1.9 \cdot 10^{3}$	$2.2 \cdot 10^{2}$	$6.2 \cdot 10^{2}$	$2.1 \cdot 10^{3}$	$8.3 \cdot 10^{3}$	$3.1 \cdot 10^{4}$
B97	$4.3 \cdot 10^{2}$	$1.1 \cdot 10^{3}$	$4.7 \cdot 10^{3}$	$1.2 \cdot 10^{4}$	$8.1 \cdot 10^{4}$	$9.0 \cdot 10^{1}$	$3.5 \cdot 10^{2}$	$9.0 \cdot 10^{2}$	$4.1 \cdot 10^{3}$	$1.0 \cdot 10^{4}$
B97-D	$2.8 \cdot 10^{2}$	$1.4 \cdot 10^{3}$	$3.6 \cdot 10^{3}$	$1.7 \cdot 10^{4}$	$6.9 \cdot 10^{4}$	$8.6 \cdot 10^{1}$	$4.3 \cdot 10^{2}$	$1.8 \cdot 10^{3}$	$6.3 \cdot 10^{3}$	$2.7 \cdot 10^{4}$
wB97	$7.5 \cdot 10^{2}$	$8.9 \cdot 10^{2}$	$3.8 \cdot 10^{3}$	$2.6 \cdot 10^{4}$	$2.0 \cdot 10^{5}$	$1.5 \cdot 10^{2}$	$3.1 \cdot 10^{2}$	$1.2 \cdot 10^{3}$	$3.4 \cdot 10^{3}$	$1.3 \cdot 10^{4}$
wB97X	$8.1 \cdot 10^{2}$	$1.4 \cdot 10^{3}$	$6.7 \cdot 10^{3}$	$5.1 \cdot 10^{4}$	$3.6 \cdot 10^{5}$	$1.8 \cdot 10^{2}$	$2.6 \cdot 10^{2}$	$1.3 \cdot 10^{3}$	$3.0 \cdot 10^{3}$	$1.5 \cdot 10^{4}$
wB97X-D	$1.6 \cdot 10^{3}$	$2.7 \cdot 10^{3}$	$1.5 \cdot 10^{4}$	$9.7 \cdot 10^{4}$	$6.9 \cdot 10^{5}$	$1.9 \cdot 10^{2}$	$2.7 \cdot 10^{2}$	$1.3 \cdot 10^{3}$	$3.3 \cdot 10^{3}$	$1.6 \cdot 10^{4}$
wB97X-D3	$1.0 \cdot 10^{3}$	$1.6 \cdot 10^{3}$	$9.5 \cdot 10^{3}$	$6.0 \cdot 10^{4}$	$4.4 \cdot 10^{5}$	$1.8 \cdot 10^{2}$	$2.6 \cdot 10^{2}$	$1.3 \cdot 10^{3}$	$3.0 \cdot 10^{3}$	$1.5 \cdot 10^{4}$
wB97M-V	$1.9 \cdot 10^{2}$	$3.1 \cdot 10^{2}$	$1.4 \cdot 10^{3}$	$4.5 \cdot 10^{3}$	$2.3 \cdot 10^{4}$	$2.0 \cdot 10^{2}$	$3.0 \cdot 10^{2}$	$1.4 \cdot 10^{3}$	$3.2 \cdot 10^{3}$	$1.6 \cdot 10^{4}$
wB97X-V	$4.4 \cdot 10^{1}$	$5.6 \cdot 10^{1}$	$3.6 \cdot 10^{2}$	$2.8 \cdot 10^{2}$	$3.2 \cdot 10^{3}$	$1.4 \cdot 10^{2}$	$2.0 \cdot 10^{2}$	$1.0 \cdot 10^{3}$	$2.3 \cdot 10^{3}$	$1.2 \cdot 10^{4}$
B97M-V	$9.1 \cdot 10^{1}$	$2.8 \cdot 10^{2}$	$6.6 \cdot 10^{2}$	$3.3 \cdot 10^{3}$	$1.4 \cdot 10^{4}$	$2.5 \cdot 10^{2}$	$8.6 \cdot 10^{2}$	$2.3 \cdot 10^{3}$	$1.1 \cdot 10^{4}$	$2.9 \cdot 10^{4}$
B1PW91	$1.4 \cdot 10^{1}$	$1.9 \cdot 10^{1}$	$1.1 \cdot 10^{2}$	$4.6 \cdot 10^{2}$	$3.4 \cdot 10^{3}$	$3.7 \cdot 10^{1}$	$2.9 \cdot 10^{2}$	$1.2 \cdot 10^{3}$	$4.0 \cdot 10^{3}$	$1.7 \cdot 10^{4}$
PW91	$5.9 \cdot 10^{0}$	$3.3 \cdot 10^{1}$	$9.6 \cdot 10^{1}$	$4.7 \cdot 10^{2}$	$1.4 \cdot 10^{3}$	$1.4 \cdot 10^{2}$	$7.4 \cdot 10^{2}$	$1.5 \cdot 10^{3}$	$7.7 \cdot 10^{3}$	$1.6 \cdot 10^{4}$
mPW91	$4.8 \cdot 10^{0}$	$2.9 \cdot 10^{1}$	$1.2 \cdot 10^{2}$	$3.4 \cdot 10^{2}$	$3.0 \cdot 10^{3}$	$1.4 \cdot 10^{2}$	$7.1 \cdot 10^{2}$	$1.7 \cdot 10^{3}$	$7.7 \cdot 10^{3}$	$3.0 \cdot 10^{4}$
VSXC	$2.0 \cdot 10^{2}$	$3.7 \cdot 10^{2}$	$1.2 \cdot 10^{3}$	$3.5 \cdot 10^{3}$	$8.6 \cdot 10^{3}$	$4.7 \cdot 10^{1}$	$2.3 \cdot 10^{2}$	$7.7 \cdot 10^{2}$	$2.2 \cdot 10^{3}$	$8.8 \cdot 10^{3}$
SCAN	$1.8 \cdot 10^{2}$	$6.6 \cdot 10^{2}$	$2.7 \cdot 10^{3}$	$2.5 \cdot 10^{4}$	$3.8 \cdot 10^{5}$	$8.1 \cdot 10^{2}$	$2.0 \cdot 10^{3}$	$1.1 \cdot 10^{4}$	$6.3 \cdot 10^{4}$	$9.3 \cdot 10^{5}$
SCAN0	$1.3 \cdot 10^{2}$	$6.0 \cdot 10^{2}$	$2.3 \cdot 10^{3}$	$2.0 \cdot 10^{4}$	$3.0 \cdot 10^{5}$	$8.3 \cdot 10^{2}$	$2.6 \cdot 10^{3}$	$1.2 \cdot 10^{4}$	$7.5 \cdot 10^{4}$	$9.8 \cdot 10^{5}$
M06	$1.6 \cdot 10^{2}$	$1.9 \cdot 10^{2}$	$8.8 \cdot 10^{2}$	$6.5 \cdot 10^{3}$	$3.3 \cdot 10^{4}$	$2.1 \cdot 10^{2}$	$1.1 \cdot 10^{3}$	$9.4 \cdot 10^{2}$	$4.8 \cdot 10^{3}$	$1.4 \cdot 10^{4}$
M06-L	$2.4 \cdot 10^{2}$	$1.1 \cdot 10^{2}$	$1.1 \cdot 10^{3}$	$6.7 \cdot 10^{3}$	$2.6 \cdot 10^{4}$	$7.8 \cdot 10^{2}$	$4.0 \cdot 10^{2}$	$1.3 \cdot 10^{3}$	$7.5 \cdot 10^{3}$	$4.7 \cdot 10^{4}$
M06-HF	$8.5 \cdot 10^{2}$	$1.7 \cdot 10^{3}$	$8.4 \cdot 10^{3}$	$1.8 \cdot 10^{4}$	$9.6 \cdot 10^{4}$	$1.7 \cdot 10^{3}$	$1.0 \cdot 10^{3}$	$1.2 \cdot 10^{3}$	$1.1 \cdot 10^{4}$	$4.6 \cdot 10^{4}$
M06-2X	$1.7 \cdot 10^{2}$	$3.9 \cdot 10^{2}$	$1.8 \cdot 10^{3}$	$1.0 \cdot 10^{4}$	$6.4 \cdot 10^{4}$	$4.7 \cdot 10^{2}$	$4.3 \cdot 10^{2}$	$1.1 \cdot 10^{3}$	$6.8 \cdot 10^{3}$	$2.2 \cdot 10^{4}$
M11	$3.5 \cdot 10^{2}$	$1.3 \cdot 10^{2}$	$5.8 \cdot 10^{2}$	$3.6 \cdot 10^{3}$	$9.5 \cdot 10^{3}$	$2.0 \cdot 10^{2}$	$5.5 \cdot 10^{2}$	$1.8 \cdot 10^{3}$	$6.6 \cdot 10^{3}$	$2.8 \cdot 10^{4}$
M11-L	$2.3 \cdot 10^{2}$	$3.4 \cdot 10^{2}$	$1.4 \cdot 10^{3}$	$4.0 \cdot 10^{3}$	$1.7 \cdot 10^{4}$	$1.0 \cdot 10^{3}$	$1.4 \cdot 10^{3}$	$1.7 \cdot 10^{3}$	$5.1 \cdot 10^{3}$	$3.4 \cdot 10^{4}$
SOGGA11	$3.7 \cdot 10^{2}$	$1.2 \cdot 10^{3}$	$3.2 \cdot 10^{3}$	$1.8 \cdot 10^{4}$	$6.9 \cdot 10^{4}$	$4.0 \cdot 10^{1}$	$2.2 \cdot 10^{2}$	$1.2 \cdot 10^{3}$	$6.5 \cdot 10^{3}$	$2.8 \cdot 10^{4}$
SOGGA11-X	$5.6 \cdot 10^{1}$	$2.6 \cdot 10^{2}$	$4.0 \cdot 10^{2}$	$2.1 \cdot 10^{3}$	$6.9 \cdot 10^{3}$	$1.3 \cdot 10^{2}$	$2.4 \cdot 10^{2}$	$1.2 \cdot 10^{3}$	$3.0 \cdot 10^{3}$	$1.5 \cdot 10^{4}$
N12	$1.4 \cdot 10^{3}$	$3.2 \cdot 10^{3}$	$7.7 \cdot 10^{3}$	$5.6 \cdot 10^{4}$	$5.7 \cdot 10^{5}$	$1.6 \cdot 10^{2}$	$7.6 \cdot 10^{2}$	$2.6 \cdot 10^{3}$	$1.2 \cdot 10^{4}$	$4.1 \cdot 10^{4}$
N12-SX	$1.2 \cdot 10^{2}$	$2.3 \cdot 10^{2}$	$1.4 \cdot 10^{3}$	$7.5 \cdot 10^{3}$	$3.9 \cdot 10^{4}$	$1.2 \cdot 10^{2}$	$5.3 \cdot 10^{2}$	$2.3 \cdot 10^{3}$	$1.0 \cdot 10^{4}$	$3.8 \cdot 10^{4}$
MN12-L	$2.1 \cdot 10^{2}$	$1.4 \cdot 10^{2}$	$6.1 \cdot 10^{2}$	$1.9 \cdot 10^{3}$	$7.7 \cdot 10^{3}$	$1.7 \cdot 10^{2}$	$7.8 \cdot 10^{2}$	$3.5 \cdot 10^{3}$	$1.2 \cdot 10^{4}$	$4.9 \cdot 10^{4}$
MN12-SX	$1.2 \cdot 10^{2}$	$6.3 \cdot 10^{1}$	$1.4 \cdot 10^{2}$	$1.0 \cdot 10^{3}$	$2.3 \cdot 10^{3}$	$4.2 \cdot 10^{2}$	$5.9 \cdot 10^{2}$	$9.3 \cdot 10^{2}$	$4.8 \cdot 10^{3}$	$1.2 \cdot 10^{4}$
MN15	$5.1 \cdot 10^{1}$	$1.8 \cdot 10^{1}$	$9.1 \cdot 10^{1}$	$3.9 \cdot 10^{2}$	$2.5 \cdot 10^{3}$	$5.4 \cdot 10^{2}$	$3.4 \cdot 10^{2}$	$2.6 \cdot 10^{2}$	$2.3 \cdot 10^{3}$	$9.5 \cdot 10^{3}$
MN15-L	$1.7 \cdot 10^{2}$	$6.3 \cdot 10^{1}$	$2.9 \cdot 10^{2}$	$1.2 \cdot 10^{3}$	$4.8 \cdot 10^{3}$	$5.2 \cdot 10^{2}$	$5.3 \cdot 10^{2}$	$2.6 \cdot 10^{3}$	$5.6 \cdot 10^{3}$	$3.0 \cdot 10^{4}$

Table S14: RMSE values for various energy derivatives obtained with the different functionals used in this work combined with the (250, 974) integration grid for the HCN · BrF system.

DFA	$\mathrm{d}^n E_c/\mathrm{d}\xi^n$ [$\times 10^{-8}$]					$\mathrm{d}^n E_x/\mathrm{d}\xi^n$ [$\times 10^{-8}$]				
	0	1	2	3	4	0	1	2	3	4
SVWN5	$6.0 \cdot 10^{-3}$	$1.6 \cdot 10^{-2}$	$1.6 \cdot 10^{-1}$	$1.4 \cdot 10^{0}$	$8.4 \cdot 10^{0}$	$1.5 \cdot 10^{-1}$	$5.1 \cdot 10^{-1}$	$5.1 \cdot 10^{0}$	$3.8 \cdot 10^{1}$	$3.6 \cdot 10^{2}$
SPW92	$5.9 \cdot 10^{-3}$	$1.4 \cdot 10^{-2}$	$1.5 \cdot 10^{-1}$	$1.4 \cdot 10^{0}$	$8.3 \cdot 10^{0}$	$1.5 \cdot 10^{-1}$	$5.1 \cdot 10^{-1}$	$5.1 \cdot 10^{0}$	$3.9 \cdot 10^{1}$	$3.7 \cdot 10^{2}$
BHH	$4.0 \cdot 10^{-3}$	$1.1 \cdot 10^{-2}$	$8.8 \cdot 10^{-2}$	$9.5 \cdot 10^{-1}$	$4.3 \cdot 10^{0}$	$7.2 \cdot 10^{-2}$	$4.1 \cdot 10^{-1}$	$2.7 \cdot 10^{0}$	$2.5 \cdot 10^{1}$	$2.1 \cdot 10^{2}$
BLYP	$8.2 \cdot 10^{-3}$	$1.1 \cdot 10^{-2}$	$5.2 \cdot 10^{-2}$	$5.0 \cdot 10^{-1}$	$5.8 \cdot 10^{0}$	$1.1 \cdot 10^{-1}$	$9.2 \cdot 10^{-1}$	$6.1 \cdot 10^{0}$	$6.0 \cdot 10^{1}$	$4.8 \cdot 10^{2}$
B1LYP	$3.1 \cdot 10^{-3}$	$9.9 \cdot 10^{-3}$	$8.9 \cdot 10^{-2}$	$6.8 \cdot 10^{-1}$	$3.5 \cdot 10^{0}$	$1.3 \cdot 10^{-1}$	$3.8 \cdot 10^{-1}$	$3.6 \cdot 10^{0}$	$3.0 \cdot 10^{1}$	$2.6 \cdot 10^{2}$
B3LYP	$4.8 \cdot 10^{-3}$	$7.7 \cdot 10^{-3}$	$7.5 \cdot 10^{-2}$	$7.1 \cdot 10^{-1}$	$4.1 \cdot 10^{0}$	$1.4 \cdot 10^{-1}$	$4.4 \cdot 10^{-1}$	$4.4 \cdot 10^{0}$	$3.2 \cdot 10^{1}$	$3.2 \cdot 10^{2}$
BHHLYP	$3.0 \cdot 10^{-3}$	$5.6 \cdot 10^{-3}$	$4.5 \cdot 10^{-2}$	$4.4 \cdot 10^{-1}$	$2.1 \cdot 10^{0}$	$5.9 \cdot 10^{-2}$	$2.9 \cdot 10^{-1}$	$2.7 \cdot 10^{0}$	$2.0 \cdot 10^{1}$	$1.9 \cdot 10^{2}$
LC-BLYP	$3.3 \cdot 10^{-3}$	$5.4 \cdot 10^{-3}$	$3.9 \cdot 10^{-2}$	$2.9 \cdot 10^{-1}$	$1.4 \cdot 10^{0}$	$1.8 \cdot 10^{-1}$	$8.5 \cdot 10^{-1}$	$5.3 \cdot 10^{0}$	$4.9 \cdot 10^{1}$	$3.5 \cdot 10^{2}$
CAMB3LYP	$3.1 \cdot 10^{-3}$	$7.4 \cdot 10^{-3}$	$7.0 \cdot 10^{-2}$	$8.6 \cdot 10^{-1}$	$3.6 \cdot 10^{0}$	$8.4 \cdot 10^{-2}$	$6.2 \cdot 10^{-1}$	$4.1 \cdot 10^{0}$	$3.9 \cdot 10^{1}$	$2.9 \cdot 10^{2}$
PBE	$2.0 \cdot 10^{-2}$	$9.7 \cdot 10^{-2}$	$7.1 \cdot 10^{-1}$	$5.8 \cdot 10^{0}$	$5.6 \cdot 10^{1}$	$1.6 \cdot 10^{-1}$	$7.6 \cdot 10^{-1}$	$7.8 \cdot 10^{0}$	$6.6 \cdot 10^{1}$	$6.2 \cdot 10^{2}$
PBE0	$2.4 \cdot 10^{-2}$	$5.9 \cdot 10^{-2}$	$5.1 \cdot 10^{-1}$	$4.5 \cdot 10^{0}$	$4.6 \cdot 10^{1}$	$1.0 \cdot 10^{-1}$	$6.6 \cdot 10^{-1}$	$4.9 \cdot 10^{0}$	$4.5 \cdot 10^{1}$	$3.6 \cdot 10^{2}$
PBE50	$2.5 \cdot 10^{-2}$	$6.0 \cdot 10^{-2}$	$4.5 \cdot 10^{-1}$	$3.8 \cdot 10^{0}$	$5.8 \cdot 10^{1}$	$5.0 \cdot 10^{-2}$	$3.6 \cdot 10^{-1}$	$3.1 \cdot 10^{0}$	$2.5 \cdot 10^{1}$	$2.3 \cdot 10^{2}$
LC-wPBE	$2.0 \cdot 10^{-2}$	$3.8 \cdot 10^{-2}$	$3.5 \cdot 10^{-1}$	$3.3 \cdot 10^{0}$	$2.8 \cdot 10^{1}$	$1.6 \cdot 10^{-1}$	$8.5 \cdot 10^{-1}$	$6.8 \cdot 10^{0}$	$5.0 \cdot 10^{1}$	$4.8 \cdot 10^{2}$
TPSS	$1.0 \cdot 10^{-2}$	$6.8 \cdot 10^{-2}$	$4.0 \cdot 10^{-1}$	$4.8 \cdot 10^{0}$	$6.2 \cdot 10^{1}$	$1.3 \cdot 10^{-1}$	$8.7 \cdot 10^{-1}$	$7.9 \cdot 10^{0}$	$6.5 \cdot 10^{1}$	$6.3 \cdot 10^{2}$
RevTPSS	$1.2 \cdot 10^{-2}$	$7.3 \cdot 10^{-2}$	$4.6 \cdot 10^{-1}$	$5.1 \cdot 10^{0}$	$4.9 \cdot 10^{1}$	$1.4 \cdot 10^{-1}$	$9.7 \cdot 10^{-1}$	$7.9 \cdot 10^{0}$	$6.8 \cdot 10^{1}$	$6.3 \cdot 10^{2}$
TPSSh	$1.8 \cdot 10^{-2}$	$6.4 \cdot 10^{-2}$	$6.2 \cdot 10^{-1}$	$5.5 \cdot 10^{0}$	$6.0 \cdot 10^{1}$	$1.0 \cdot 10^{-1}$	$6.7 \cdot 10^{-1}$	$5.8 \cdot 10^{0}$	$5.0 \cdot 10^{1}$	$4.5 \cdot 10^{2}$
B97	$6.2 \cdot 10^{0}$	$5.9 \cdot 10^{1}$	$4.9 \cdot 10^{2}$	$4.6 \cdot 10^{3}$	$4.0 \cdot 10^{4}$	$1.2 \cdot 10^{-1}$	$5.6 \cdot 10^{-1}$	$5.0 \cdot 10^{0}$	$4.0 \cdot 10^{1}$	$3.6 \cdot 10^{2}$
B97-D	$2.4 \cdot 10^{0}$	$2.3 \cdot 10^{1}$	$2.0 \cdot 10^{2}$	$1.9 \cdot 10^{3}$	$1.7 \cdot 10^{4}$	$1.2 \cdot 10^{-1}$	$8.1 \cdot 10^{-1}$	$6.3 \cdot 10^{0}$	$5.5 \cdot 10^{1}$	$4.6 \cdot 10^{2}$
wB97	$1.6 \cdot 10^{1}$	$1.4 \cdot 10^{2}$	$9.6 \cdot 10^{2}$	$8.6 \cdot 10^{3}$	$6.4 \cdot 10^{4}$	$1.8 \cdot 10^{-1}$	$7.5 \cdot 10^{-1}$	$5.2 \cdot 10^{0}$	$3.6 \cdot 10^{1}$	$3.2 \cdot 10^{2}$
wB97X	$4.0 \cdot 10^{1}$	$3.0 \cdot 10^{2}$	$2.7 \cdot 10^{3}$	$2.1 \cdot 10^{4}$	$1.9 \cdot 10^{5}$	$1.5 \cdot 10^{-1}$	$6.9 \cdot 10^{-1}$	$5.8 \cdot 10^{0}$	$4.3 \cdot 10^{1}$	$4.0 \cdot 10^{2}$
wB97X-D	$8.3 \cdot 10^{1}$	$7.0 \cdot 10^{2}$	$5.8 \cdot 10^{3}$	$5.1 \cdot 10^{4}$	$4.3 \cdot 10^{5}$	$1.1 \cdot 10^{-1}$	$6.3 \cdot 10^{-1}$	$4.8 \cdot 10^{0}$	$4.5 \cdot 10^{1}$	$3.4 \cdot 10^{2}$
wB97X-D3	$5.0 \cdot 10^{1}$	$4.1 \cdot 10^{2}$	$3.4 \cdot 10^{3}$	$3.0 \cdot 10^{4}$	$2.5 \cdot 10^{5}$	$1.2 \cdot 10^{-1}$	$5.9 \cdot 10^{-1}$	$4.7 \cdot 10^{0}$	$4.4 \cdot 10^{1}$	$3.3 \cdot 10^{2}$
wB97M-V	$1.5 \cdot 10^{0}$	$1.1 \cdot 10^{1}$	$1.1 \cdot 10^{2}$	$8.2 \cdot 10^{2}$	$7.7 \cdot 10^{3}$	$9.7 \cdot 10^{-2}$	$7.0 \cdot 10^{-1}$	$4.3 \cdot 10^{0}$	$4.0 \cdot 10^{1}$	$2.7 \cdot 10^{2}$
wB97X-V	$2.7 \cdot 10^{-1}$	$1.9 \cdot 10^{0}$	$1.9 \cdot 10^{1}$	$1.4 \cdot 10^{2}$	$1.4 \cdot 10^{3}$	$9.5 \cdot 10^{-2}$	$5.7 \cdot 10^{-1}$	$4.1 \cdot 10^{0}$	$3.9 \cdot 10^{1}$	$2.7 \cdot 10^{2}$
B97M-V	$1.1 \cdot 10^{0}$	$1.0 \cdot 10^{1}$	$7.7 \cdot 10^{1}$	$7.3 \cdot 10^{2}$	$6.0 \cdot 10^{3}$	$7.2 \cdot 10^{-2}$	$5.0 \cdot 10^{-1}$	$2.9 \cdot 10^{0}$	$2.8 \cdot 10^{1}$	$1.9 \cdot 10^{2}$
B1PW91	$1.8 \cdot 10^{-1}$	$1.1 \cdot 10^{0}$	$1.2 \cdot 10^{1}$	$8.5 \cdot 10^{1}$	$9.9 \cdot 10^{2}$	$3.3 \cdot 10^{-2}$	$5.0 \cdot 10^{-1}$	$4.5 \cdot 10^{0}$	$3.7 \cdot 10^{1}$	$3.5 \cdot 10^{2}$
PW91	$1.2 \cdot 10^{-1}$	$1.2 \cdot 10^{0}$	$7.6 \cdot 10^{0}$	$9.5 \cdot 10^{1}$	$8.5 \cdot 10^{2}$	$6.0 \cdot 10^{-1}$	$6.3 \cdot 10^{0}$	$4.5 \cdot 10^{1}$	$5.7 \cdot 10^{2}$	$5.0 \cdot 10^{3}$
mPW91	$1.9 \cdot 10^{-1}$	$1.3 \cdot 10^{0}$	$1.5 \cdot 10^{1}$	$1.2 \cdot 10^{2}$	$1.4 \cdot 10^{3}$	$1.1 \cdot 10^{0}$	$7.5 \cdot 10^{0}$	$8.4 \cdot 10^{1}$	$6.7 \cdot 10^{2}$	$8.0 \cdot 10^{3}$
VSXC	$2.1 \cdot 10^{-1}$	$5.7 \cdot 10^{-1}$	$2.9 \cdot 10^{0}$	$2.5 \cdot 10^{1}$	$2.5 \cdot 10^{2}$	$2.2 \cdot 10^{-1}$	$1.1 \cdot 10^{0}$	$7.0 \cdot 10^{0}$	$5.7 \cdot 10^{1}$	$3.9 \cdot 10^{2}$
SCAN	$1.9 \cdot 10^{1}$	$7.2 \cdot 10^{1}$	$8.5 \cdot 10^{2}$	$1.0 \cdot 10^{4}$	$1.7 \cdot 10^{5}$	$6.6 \cdot 10^{1}$	$4.9 \cdot 10^{2}$	$6.2 \cdot 10^{3}$	$7.2 \cdot 10^{4}$	$9.9 \cdot 10^{5}$
SCAN0	$2.4 \cdot 10^{0}$	$1.8 \cdot 10^{1}$	$3.4 \cdot 10^{2}$	$5.3 \cdot 10^{3}$	$1.3 \cdot 10^{5}$	$2.0 \cdot 10^{1}$	$1.5 \cdot 10^{2}$	$2.0 \cdot 10^{3}$	$2.2 \cdot 10^{4}$	$3.9 \cdot 10^{5}$
M06	$7.7 \cdot 10^{-1}$	$6.7 \cdot 10^{0}$	$3.0 \cdot 10^{1}$	$3.3 \cdot 10^{2}$	$1.6 \cdot 10^{3}$	$1.6 \cdot 10^{0}$	$7.9 \cdot 10^{0}$	$5.0 \cdot 10^{1}$	$4.7 \cdot 10^{2}$	$4.5 \cdot 10^{3}$
M06-L	$7.4 \cdot 10^{-1}$	$8.3 \cdot 10^{0}$	$5.0 \cdot 10^{1}$	$6.3 \cdot 10^{2}$	$4.8 \cdot 10^{3}$	$2.7 \cdot 10^{0}$	$1.4 \cdot 10^{1}$	$1.4 \cdot 10^{2}$	$1.3 \cdot 10^{3}$	$1.3 \cdot 10^{4}$
M06-HF	$5.9 \cdot 10^{0}$	$5.4 \cdot 10^{1}$	$4.2 \cdot 10^{2}$	$4.0 \cdot 10^{3}$	$3.0 \cdot 10^{4}$	$4.6 \cdot 10^{0}$	$3.8 \cdot 10^{1}$	$3.3 \cdot 10^{2}$	$3.1 \cdot 10^{3}$	$2.9 \cdot 10^{4}$
M06-2X	$5.0 \cdot 10^{0}$	$3.5 \cdot 10^{1}$	$3.5 \cdot 10^{2}$	$2.6 \cdot 10^{3}$	$2.6 \cdot 10^{4}$	$2.8 \cdot 10^{0}$	$1.4 \cdot 10^{1}$	$1.4 \cdot 10^{2}$	$1.3 \cdot 10^{3}$	$1.2 \cdot 10^{4}$
M11	$9.7 \cdot 10^{-1}$	$7.0 \cdot 10^{0}$	$6.7 \cdot 10^{1}$	$5.8 \cdot 10^{2}$	$5.5 \cdot 10^{3}$	$2.7 \cdot 10^{0}$	$9.8 \cdot 10^{0}$	$8.0 \cdot 10^{1}$	$7.1 \cdot 10^{2}$	$6.7 \cdot 10^{3}$
M11-L	$3.6 \cdot 10^{-1}$	$2.2 \cdot 10^{0}$	$3.9 \cdot 10^{0}$	$5.0 \cdot 10^{1}$	$4.0 \cdot 10^{2}$	$3.2 \cdot 10^{0}$	$1.9 \cdot 10^{1}$	$4.8 \cdot 10^{1}$	$5.5 \cdot 10^{2}$	$5.5 \cdot 10^{3}$
SOGGA11	$7.6 \cdot 10^{-1}$	$3.1 \cdot 10^{0}$	$1.9 \cdot 10^{1}$	$1.5 \cdot 10^{2}$	$2.7 \cdot 10^{3}$	$1.5 \cdot 10^{-1}$	$7.7 \cdot 10^{-1}$	$7.6 \cdot 10^{0}$	$5.7 \cdot 10^{1}$	$6.6 \cdot 10^{2}$
SOGGA11-X	$1.5 \cdot 10^{-1}$	$1.1 \cdot 10^{0}$	$1.0 \cdot 10^{1}$	$1.0 \cdot 10^{2}$	$9.7 \cdot 10^{2}$	$9.8 \cdot 10^{-2}$	$2.1 \cdot 10^{-1}$	$1.9 \cdot 10^{0}$	$1.6 \cdot 10^{1}$	$1.1 \cdot 10^{2}$
N12	$1.2 \cdot 10^{1}$	$1.4 \cdot 10^{2}$	$9.5 \cdot 10^{2}$	$1.1 \cdot 10^{4}$	$1.4 \cdot 10^{5}$	$3.5 \cdot 10^{-1}$	$1.5 \cdot 10^{0}$	$7.0 \cdot 10^{0}$	$5.2 \cdot 10^{1}$	$4.7 \cdot 10^{2}$
N12-SX	$1.0 \cdot 10^{0}$	$8.2 \cdot 10^{0}$	$6.5 \cdot 10^{1}$	$7.3 \cdot 10^{2}$	$6.4 \cdot 10^{3}$	$4.0 \cdot 10^{-1}$	$1.7 \cdot 10^{0}$	$9.7 \cdot 10^{0}$	$7.8 \cdot 10^{1}$	$9.8 \cdot 10^{2}$
MN12-L	$2.7 \cdot 10^{-1}$	$6.4 \cdot 10^{-1}$	$4.8 \cdot 10^{0}$	$5.0 \cdot 10^{1}$	$4.6 \cdot 10^{2}$	$2.6 \cdot 10^{0}$	$3.3 \cdot 10^{0}$	$3.6 \cdot 10^{1}$	$3.3 \cdot 10^{2}$	$3.1 \cdot 10^{3}$
MN12-SX	$2.3 \cdot 10^{-1}$	$1.4 \cdot 10^{0}$	$1.3 \cdot 10^{1}$	$1.2 \cdot 10^{2}$	$1.1 \cdot 10^{3}$	$1.6 \cdot 10^{0}$	$3.4 \cdot 10^{0}$	$2.9 \cdot 10^{1}$	$2.6 \cdot 10^{2}$	$2.5 \cdot 10^{3}$
MN15	$1.7 \cdot 10^{-1}$	$8.9 \cdot 10^{-1}$	$4.3 \cdot 10^{0}$	$4.0 \cdot 10^{1}$	$3.8 \cdot 10^{2}$	$5.6 \cdot 10^{-1}$	$2.5 \cdot 10^{0}$	$1.2 \cdot 10^{1}$	$1.1 \cdot 10^{2}$	$9.7 \cdot 10^{2}$
MN15-L	$2.3 \cdot 10^{-1}$	$1.0 \cdot 10^{0}$	$5.7 \cdot 10^{0}$	$4.6 \cdot 10^{1}$	$3.9 \cdot 10^{2}$	$4.9 \cdot 10^{-1}$	$3.0 \cdot 10^{0}$	$1.9 \cdot 10^{1}$	$1.7 \cdot 10^{2}$	$1.4 \cdot 10^{3}$

Table S15: RMSE values for various energy derivatives obtained with the different functionals used in this work combined with the (99, 590) integration grid for the OC · HF system.

**DFA**	$\mathrm{d}^n E_c/\mathrm{d}\xi^n$ [$\times 10^{-8}$] 0	1	2	3	4	$\mathrm{d}^n E_x/\mathrm{d}\xi^n$ [$\times 10^{-8}$] 0	1	2	3	4
SVWN5	$1.1 \cdot 10^{-1}$	$3.7 \cdot 10^{-1}$	$2.5 \cdot 10^{0}$	$1.7 \cdot 10^{1}$	$1.0 \cdot 10^{2}$	$3.8 \cdot 10^{0}$	$7.5 \cdot 10^{0}$	$1.9 \cdot 10^{1}$	$1.5 \cdot 10^{2}$	$8.2 \cdot 10^{2}$
SPW92	$1.1 \cdot 10^{-1}$	$3.7 \cdot 10^{-1}$	$2.6 \cdot 10^{0}$	$1.8 \cdot 10^{1}$	$1.2 \cdot 10^{2}$	$3.8 \cdot 10^{0}$	$7.5 \cdot 10^{0}$	$2.0 \cdot 10^{1}$	$1.5 \cdot 10^{2}$	$8.3 \cdot 10^{2}$
BHH	$1.2 \cdot 10^{0}$	$1.6 \cdot 10^{0}$	$1.1 \cdot 10^{1}$	$1.1 \cdot 10^{2}$	$7.0 \cdot 10^{2}$	$1.4 \cdot 10^{0}$	$5.0 \cdot 10^{0}$	$1.7 \cdot 10^{1}$	$8.9 \cdot 10^{1}$	$7.0 \cdot 10^{2}$
BLYP	$6.2 \cdot 10^{-1}$	$1.2 \cdot 10^{0}$	$8.9 \cdot 10^{0}$	$5.4 \cdot 10^{1}$	$4.2 \cdot 10^{2}$	$4.1 \cdot 10^{-1}$	$8.3 \cdot 10^{0}$	$5.8 \cdot 10^{1}$	$3.6 \cdot 10^{2}$	$3.1 \cdot 10^{3}$
B1LYP	$1.7 \cdot 10^{-1}$	$1.2 \cdot 10^{0}$	$6.0 \cdot 10^{0}$	$5.2 \cdot 10^{1}$	$2.9 \cdot 10^{2}$	$6.5 \cdot 10^{0}$	$5.7 \cdot 10^{0}$	$3.2 \cdot 10^{1}$	$3.0 \cdot 10^{2}$	$1.9 \cdot 10^{3}$
B3LYP	$1.2 \cdot 10^{-1}$	$7.1 \cdot 10^{-1}$	$5.4 \cdot 10^{0}$	$3.7 \cdot 10^{1}$	$2.6 \cdot 10^{2}$	$6.1 \cdot 10^{0}$	$5.8 \cdot 10^{0}$	$4.2 \cdot 10^{1}$	$3.0 \cdot 10^{2}$	$2.1 \cdot 10^{3}$
BHHLYP	$8.0 \cdot 10^{-1}$	$9.1 \cdot 10^{-1}$	$5.4 \cdot 10^{0}$	$4.7 \cdot 10^{1}$	$2.7 \cdot 10^{2}$	$3.3 \cdot 10^{0}$	$4.8 \cdot 10^{0}$	$2.4 \cdot 10^{1}$	$2.2 \cdot 10^{2}$	$1.4 \cdot 10^{3}$
LC-BLYP	$5.7 \cdot 10^{-1}$	$1.6 \cdot 10^{0}$	$8.6 \cdot 10^{0}$	$7.7 \cdot 10^{1}$	$4.3 \cdot 10^{2}$	$3.5 \cdot 10^{0}$	$1.3 \cdot 10^{1}$	$3.3 \cdot 10^{1}$	$2.6 \cdot 10^{2}$	$1.3 \cdot 10^{3}$
CAMB3LYP	$2.8 \cdot 10^{-1}$	$7.0 \cdot 10^{-1}$	$6.6 \cdot 10^{0}$	$3.9 \cdot 10^{1}$	$3.4 \cdot 10^{2}$	$5.8 \cdot 10^{0}$	$7.9 \cdot 10^{0}$	$4.8 \cdot 10^{1}$	$2.6 \cdot 10^{2}$	$2.3 \cdot 10^{3}$
PBE	$2.8 \cdot 10^{0}$	$7.4 \cdot 10^{0}$	$5.1 \cdot 10^{1}$	$4.6 \cdot 10^{2}$	$3.0 \cdot 10^{3}$	$4.5 \cdot 10^{0}$	$2.0 \cdot 10^{1}$	$1.1 \cdot 10^{2}$	$6.9 \cdot 10^{2}$	$5.4 \cdot 10^{3}$
PBE0	$2.6 \cdot 10^{0}$	$6.9 \cdot 10^{0}$	$5.1 \cdot 10^{1}$	$3.8 \cdot 10^{2}$	$2.4 \cdot 10^{3}$	$1.1 \cdot 10^{1}$	$1.1 \cdot 10^{1}$	$7.5 \cdot 10^{1}$	$4.2 \cdot 10^{2}$	$3.4 \cdot 10^{3}$
PBE50	$5.7 \cdot 10^{0}$	$7.7 \cdot 10^{0}$	$4.9 \cdot 10^{1}$	$3.6 \cdot 10^{2}$	$2.3 \cdot 10^{3}$	$4.7 \cdot 10^{0}$	$7.7 \cdot 10^{0}$	$4.4 \cdot 10^{1}$	$2.7 \cdot 10^{2}$	$1.9 \cdot 10^{3}$
LC-wPBE	$1.1 \cdot 10^{0}$	$8.3 \cdot 10^{0}$	$4.9 \cdot 10^{1}$	$3.8 \cdot 10^{2}$	$2.1 \cdot 10^{3}$	$7.3 \cdot 10^{0}$	$1.3 \cdot 10^{1}$	$8.5 \cdot 10^{1}$	$5.0 \cdot 10^{2}$	$4.2 \cdot 10^{3}$
TPSS	$2.6 \cdot 10^{0}$	$8.3 \cdot 10^{0}$	$7.3 \cdot 10^{1}$	$5.0 \cdot 10^{2}$	$4.3 \cdot 10^{3}$	$2.2 \cdot 10^{1}$	$2.3 \cdot 10^{1}$	$1.5 \cdot 10^{2}$	$1.1 \cdot 10^{3}$	$9.5 \cdot 10^{3}$
RevTPSS	$3.4 \cdot 10^{0}$	$9.9 \cdot 10^{0}$	$6.3 \cdot 10^{1}$	$4.7 \cdot 10^{2}$	$3.1 \cdot 10^{3}$	$1.9 \cdot 10^{1}$	$2.2 \cdot 10^{1}$	$2.0 \cdot 10^{2}$	$1.4 \cdot 10^{3}$	$1.4 \cdot 10^{4}$
TPSSh	$1.4 \cdot 10^{0}$	$8.5 \cdot 10^{0}$	$7.4 \cdot 10^{1}$	$4.7 \cdot 10^{2}$	$4.0 \cdot 10^{3}$	$1.1 \cdot 10^{1}$	$2.4 \cdot 10^{1}$	$1.4 \cdot 10^{2}$	$1.0 \cdot 10^{3}$	$9.2 \cdot 10^{3}$
B97	$5.5 \cdot 10^{1}$	$3.1 \cdot 10^{2}$	$2.9 \cdot 10^{3}$	$1.9 \cdot 10^{4}$	$1.9 \cdot 10^{5}$	$5.8 \cdot 10^{0}$	$1.7 \cdot 10^{1}$	$6.8 \cdot 10^{1}$	$4.8 \cdot 10^{2}$	$3.0 \cdot 10^{3}$
B97-D	$3.7 \cdot 10^{1}$	$2.9 \cdot 10^{2}$	$2.1 \cdot 10^{3}$	$1.6 \cdot 10^{4}$	$1.3 \cdot 10^{5}$	$1.9 \cdot 10^{1}$	$3.2 \cdot 10^{1}$	$1.5 \cdot 10^{2}$	$7.7 \cdot 10^{2}$	$6.1 \cdot 10^{3}$
wB97	$6.2 \cdot 10^{1}$	$4.8 \cdot 10^{2}$	$2.9 \cdot 10^{3}$	$2.9 \cdot 10^{4}$	$2.8 \cdot 10^{5}$	$1.1 \cdot 10^{1}$	$1.3 \cdot 10^{1}$	$6.5 \cdot 10^{1}$	$4.0 \cdot 10^{2}$	$3.0 \cdot 10^{3}$
wB97X	$6.0 \cdot 10^{1}$	$2.5 \cdot 10^{2}$	$1.9 \cdot 10^{3}$	$2.4 \cdot 10^{4}$	$3.6 \cdot 10^{5}$	$8.0 \cdot 10^{0}$	$6.4 \cdot 10^{0}$	$5.4 \cdot 10^{1}$	$3.4 \cdot 10^{2}$	$2.4 \cdot 10^{3}$
wB97X-D	$6.0 \cdot 10^{1}$	$3.7 \cdot 10^{2}$	$3.2 \cdot 10^{3}$	$4.1 \cdot 10^{4}$	$5.7 \cdot 10^{5}$	$8.5 \cdot 10^{0}$	$6.4 \cdot 10^{0}$	$3.9 \cdot 10^{1}$	$3.4 \cdot 10^{2}$	$1.9 \cdot 10^{3}$
wB97X-D3	$5.7 \cdot 10^{1}$	$2.2 \cdot 10^{2}$	$2.4 \cdot 10^{3}$	$2.6 \cdot 10^{4}$	$4.3 \cdot 10^{5}$	$6.7 \cdot 10^{0}$	$6.8 \cdot 10^{0}$	$4.1 \cdot 10^{1}$	$3.3 \cdot 10^{2}$	$1.8 \cdot 10^{3}$
wB97M-V	$1.6 \cdot 10^{1}$	$5.7 \cdot 10^{1}$	$2.9 \cdot 10^{2}$	$2.3 \cdot 10^{3}$	$1.5 \cdot 10^{4}$	$1.1 \cdot 10^{1}$	$7.8 \cdot 10^{0}$	$3.1 \cdot 10^{1}$	$2.4 \cdot 10^{2}$	$1.5 \cdot 10^{3}$
wB97X-V	$3.5 \cdot 10^{0}$	$1.4 \cdot 10^{1}$	$1.2 \cdot 10^{2}$	$8.9 \cdot 10^{2}$	$9.5 \cdot 10^{3}$	$5.1 \cdot 10^{0}$	$1.7 \cdot 10^{1}$	$6.6 \cdot 10^{1}$	$4.8 \cdot 10^{2}$	$2.9 \cdot 10^{3}$
B97M-V	$3.5 \cdot 10^{0}$	$2.3 \cdot 10^{1}$	$2.3 \cdot 10^{2}$	$2.1 \cdot 10^{3}$	$1.8 \cdot 10^{4}$	$6.4 \cdot 10^{0}$	$1.5 \cdot 10^{1}$	$1.2 \cdot 10^{2}$	$7.3 \cdot 10^{2}$	$6.5 \cdot 10^{3}$
B1PW91	$2.0 \cdot 10^{0}$	$9.6 \cdot 10^{0}$	$5.4 \cdot 10^{1}$	$3.7 \cdot 10^{2}$	$3.3 \cdot 10^{3}$	$7.5 \cdot 10^{0}$	$3.8 \cdot 10^{0}$	$4.4 \cdot 10^{1}$	$2.8 \cdot 10^{2}$	$2.1 \cdot 10^{3}$
PW91	$1.2 \cdot 10^{0}$	$8.1 \cdot 10^{0}$	$5.3 \cdot 10^{1}$	$4.7 \cdot 10^{2}$	$3.6 \cdot 10^{3}$	$4.0 \cdot 10^{0}$	$1.9 \cdot 10^{1}$	$1.2 \cdot 10^{2}$	$9.8 \cdot 10^{2}$	$1.5 \cdot 10^{4}$
mPW91	$1.9 \cdot 10^{0}$	$7.6 \cdot 10^{0}$	$5.8 \cdot 10^{1}$	$4.2 \cdot 10^{2}$	$3.7 \cdot 10^{3}$	$2.9 \cdot 10^{0}$	$1.9 \cdot 10^{1}$	$8.0 \cdot 10^{1}$	$1.1 \cdot 10^{3}$	$1.3 \cdot 10^{4}$
VSXC	$1.0 \cdot 10^{2}$	$1.2 \cdot 10^{2}$	$6.6 \cdot 10^{2}$	$4.2 \cdot 10^{3}$	$2.0 \cdot 10^{4}$	$1.0 \cdot 10^{1}$	$4.4 \cdot 10^{1}$	$1.6 \cdot 10^{2}$	$1.4 \cdot 10^{3}$	$6.5 \cdot 10^{3}$
SCAN	$1.8 \cdot 10^{2}$	$7.8 \cdot 10^{2}$	$6.3 \cdot 10^{3}$	$1.2 \cdot 10^{5}$	$2.8 \cdot 10^{6}$	$4.7 \cdot 10^{2}$	$2.8 \cdot 10^{3}$	$2.1 \cdot 10^{4}$	$3.7 \cdot 10^{5}$	$8.4 \cdot 10^{6}$
SCAN0	$1.4 \cdot 10^{2}$	$5.8 \cdot 10^{2}$	$6.6 \cdot 10^{3}$	$9.2 \cdot 10^{4}$	$3.0 \cdot 10^{6}$	$2.5 \cdot 10^{2}$	$1.8 \cdot 10^{3}$	$1.8 \cdot 10^{4}$	$2.3 \cdot 10^{5}$	$5.6 \cdot 10^{6}$
M06	$3.9 \cdot 10^{1}$	$3.8 \cdot 10^{2}$	$2.4 \cdot 10^{3}$	$1.9 \cdot 10^{4}$	$1.4 \cdot 10^{5}$	$8.7 \cdot 10^{1}$	$2.1 \cdot 10^{2}$	$1.3 \cdot 10^{3}$	$1.2 \cdot 10^{4}$	$1.1 \cdot 10^{5}$
M06-L	$8.1 \cdot 10^{1}$	$5.3 \cdot 10^{2}$	$4.2 \cdot 10^{3}$	$2.8 \cdot 10^{4}$	$2.5 \cdot 10^{5}$	$1.5 \cdot 10^{2}$	$1.9 \cdot 10^{2}$	$1.2 \cdot 10^{3}$	$1.4 \cdot 10^{4}$	$1.7 \cdot 10^{5}$
M06-HF	$7.9 \cdot 10^{1}$	$3.6 \cdot 10^{2}$	$3.5 \cdot 10^{3}$	$2.6 \cdot 10^{4}$	$2.3 \cdot 10^{5}$	$2.9 \cdot 10^{2}$	$3.0 \cdot 10^{2}$	$3.3 \cdot 10^{3}$	$3.2 \cdot 10^{4}$	$4.0 \cdot 10^{5}$
M06-2X	$2.1 \cdot 10^{1}$	$1.2 \cdot 10^{2}$	$1.1 \cdot 10^{3}$	$8.7 \cdot 10^{3}$	$8.3 \cdot 10^{4}$	$1.3 \cdot 10^{2}$	$8.3 \cdot 10^{1}$	$7.5 \cdot 10^{2}$	$8.1 \cdot 10^{3}$	$1.3 \cdot 10^{5}$
M11	$1.5 \cdot 10^{1}$	$4.8 \cdot 10^{1}$	$3.7 \cdot 10^{2}$	$2.3 \cdot 10^{3}$	$2.5 \cdot 10^{4}$	$1.4 \cdot 10^{2}$	$1.5 \cdot 10^{2}$	$1.2 \cdot 10^{3}$	$8.6 \cdot 10^{3}$	$1.1 \cdot 10^{5}$
M11-L	$1.1 \cdot 10^{2}$	$1.6 \cdot 10^{2}$	$7.3 \cdot 10^{2}$	$6.0 \cdot 10^{3}$	$5.2 \cdot 10^{4}$	$2.1 \cdot 10^{2}$	$3.0 \cdot 10^{2}$	$1.5 \cdot 10^{3}$	$1.5 \cdot 10^{4}$	$1.7 \cdot 10^{5}$
SOGGA11	$7.8 \cdot 10^{1}$	$1.5 \cdot 10^{2}$	$9.3 \cdot 10^{2}$	$4.3 \cdot 10^{3}$	$4.1 \cdot 10^{4}$	$1.5 \cdot 10^{1}$	$4.0 \cdot 10^{1}$	$1.6 \cdot 10^{2}$	$9.3 \cdot 10^{2}$	$3.7 \cdot 10^{3}$
SOGGA11-X	$5.2 \cdot 10^{1}$	$5.1 \cdot 10^{1}$	$1.7 \cdot 10^{2}$	$1.8 \cdot 10^{3}$	$2.7 \cdot 10^{4}$	$7.7 \cdot 10^{0}$	$4.4 \cdot 10^{0}$	$3.0 \cdot 10^{1}$	$1.7 \cdot 10^{2}$	$1.5 \cdot 10^{3}$
N12	$1.1 \cdot 10^{2}$	$3.9 \cdot 10^{2}$	$2.7 \cdot 10^{3}$	$2.3 \cdot 10^{4}$	$3.6 \cdot 10^{5}$	$2.7 \cdot 10^{1}$	$2.6 \cdot 10^{1}$	$3.8 \cdot 10^{1}$	$4.6 \cdot 10^{2}$	$4.7 \cdot 10^{3}$
N12-SX	$3.1 \cdot 10^{1}$	$2.3 \cdot 10^{2}$	$1.8 \cdot 10^{3}$	$1.4 \cdot 10^{4}$	$1.1 \cdot 10^{5}$	$6.7 \cdot 10^{0}$	$2.8 \cdot 10^{1}$	$9.8 \cdot 10^{1}$	$9.1 \cdot 10^{2}$	$5.6 \cdot 10^{3}$
MN12-L	$1.2 \cdot 10^{1}$	$8.8 \cdot 10^{1}$	$3.7 \cdot 10^{2}$	$3.4 \cdot 10^{3}$	$1.5 \cdot 10^{4}$	$1.7 \cdot 10^{2}$	$1.1 \cdot 10^{2}$	$9.9 \cdot 10^{2}$	$7.0 \cdot 10^{3}$	$5.6 \cdot 10^{4}$
MN12-SX	$2.0 \cdot 10^{1}$	$8.3 \cdot 10^{1}$	$4.4 \cdot 10^{2}$	$3.6 \cdot 10^{3}$	$2.1 \cdot 10^{4}$	$1.7 \cdot 10^{2}$	$7.2 \cdot 10^{1}$	$6.9 \cdot 10^{2}$	$4.3 \cdot 10^{3}$	$3.8 \cdot 10^{4}$
MN15	$2.0 \cdot 10^{1}$	$1.4 \cdot 10^{2}$	$7.6 \cdot 10^{2}$	$6.3 \cdot 10^{3}$	$3.6 \cdot 10^{4}$	$2.2 \cdot 10^{1}$	$8.0 \cdot 10^{1}$	$4.1 \cdot 10^{2}$	$3.4 \cdot 10^{3}$	$1.8 \cdot 10^{4}$
MN15-L	$9.4 \cdot 10^{0}$	$9.0 \cdot 10^{1}$	$5.5 \cdot 10^{2}$	$4.4 \cdot 10^{3}$	$2.8 \cdot 10^{4}$	$3.2 \cdot 10^{1}$	$7.4 \cdot 10^{1}$	$4.5 \cdot 10^{2}$	$2.6 \cdot 10^{3}$	$1.8 \cdot 10^{4}$

Table S16: RMSE values for various energy derivatives obtained with the different functionals used in this work combined with the (250, 974) integration grid for the OC · HF system.

**DFA**	$\mathrm{d}^nE_c/\mathrm{d}\xi^n$ [$\times 10^{-8}$] 0	1	2	3	4	$\mathrm{d}^nE_x/\mathrm{d}\xi^n$ [$\times 10^{-8}$] 0	1	2	3	4
SVWN5	$3.3 \cdot 10^{-3}$	$2.1 \cdot 10^{-3}$	$1.9 \cdot 10^{-2}$	$4.2 \cdot 10^{-1}$	$1.0 \cdot 10^{1}$	$6.8 \cdot 10^{-3}$	$9.8 \cdot 10^{-3}$	$5.1 \cdot 10^{-2}$	$6.1 \cdot 10^{-1}$	$1.3 \cdot 10^{1}$
SPW92	$2.9 \cdot 10^{-3}$	$1.8 \cdot 10^{-3}$	$3.2 \cdot 10^{-2}$	$6.8 \cdot 10^{-1}$	$1.2 \cdot 10^{1}$	$6.8 \cdot 10^{-3}$	$1.1 \cdot 10^{-2}$	$8.5 \cdot 10^{-2}$	$1.1 \cdot 10^{0}$	$1.7 \cdot 10^{1}$
BHH	$2.7 \cdot 10^{-3}$	$2.1 \cdot 10^{-3}$	$3.2 \cdot 10^{-2}$	$3.5 \cdot 10^{-1}$	$1.0 \cdot 10^{1}$	$3.0 \cdot 10^{-3}$	$3.4 \cdot 10^{-3}$	$3.3 \cdot 10^{-2}$	$4.3 \cdot 10^{-1}$	$9.6 \cdot 10^{0}$
BLYP	$3.2 \cdot 10^{-3}$	$3.2 \cdot 10^{-3}$	$5.5 \cdot 10^{-2}$	$9.3 \cdot 10^{-1}$	$1.6 \cdot 10^{1}$	$6.4 \cdot 10^{-3}$	$9.8 \cdot 10^{-3}$	$8.5 \cdot 10^{-2}$	$2.1 \cdot 10^{0}$	$2.9 \cdot 10^{1}$
B1LYP	$3.2 \cdot 10^{-3}$	$1.6 \cdot 10^{-3}$	$2.7 \cdot 10^{-2}$	$2.9 \cdot 10^{-1}$	$6.7 \cdot 10^{0}$	$5.7 \cdot 10^{-3}$	$1.3 \cdot 10^{-2}$	$8.9 \cdot 10^{-2}$	$1.5 \cdot 10^{0}$	$2.9 \cdot 10^{1}$
B3LYP	$2.7 \cdot 10^{-3}$	$1.5 \cdot 10^{-3}$	$1.7 \cdot 10^{-2}$	$4.0 \cdot 10^{-1}$	$9.0 \cdot 10^{0}$	$3.5 \cdot 10^{-3}$	$6.0 \cdot 10^{-3}$	$6.8 \cdot 10^{-2}$	$1.4 \cdot 10^{0}$	$2.7 \cdot 10^{1}$
BHHLYP	$2.4 \cdot 10^{-3}$	$1.3 \cdot 10^{-3}$	$1.6 \cdot 10^{-2}$	$3.4 \cdot 10^{-1}$	$7.8 \cdot 10^{0}$	$3.4 \cdot 10^{-3}$	$3.5 \cdot 10^{-3}$	$2.4 \cdot 10^{-2}$	$7.0 \cdot 10^{-1}$	$1.7 \cdot 10^{1}$
LC-BLYP	$3.0 \cdot 10^{-3}$	$4.4 \cdot 10^{-2}$	$3.6 \cdot 10^{-2}$	$7.9 \cdot 10^{-1}$	$1.5 \cdot 10^{1}$	$3.5 \cdot 10^{-2}$	$6.8 \cdot 10^{-3}$	$4.1 \cdot 10^{-2}$	$4.8 \cdot 10^{-1}$	$1.1 \cdot 10^{1}$
CAMB3LYP	$3.3 \cdot 10^{-3}$	$1.1 \cdot 10^{-3}$	$3.5 \cdot 10^{-2}$	$5.8 \cdot 10^{-1}$	$1.0 \cdot 10^{1}$	$5.2 \cdot 10^{-3}$	$4.9 \cdot 10^{-3}$	$5.6 \cdot 10^{-2}$	$7.6 \cdot 10^{-1}$	$1.1 \cdot 10^{1}$
PBE	$3.0 \cdot 10^{-3}$	$2.5 \cdot 10^{-3}$	$3.1 \cdot 10^{-2}$	$4.0 \cdot 10^{-1}$	$1.1 \cdot 10^{1}$	$3.2 \cdot 10^{-3}$	$6.6 \cdot 10^{-3}$	$6.0 \cdot 10^{-2}$	$6.4 \cdot 10^{-1}$	$1.4 \cdot 10^{1}$
PBE0	$3.3 \cdot 10^{-3}$	$4.2 \cdot 10^{-3}$	$4.6 \cdot 10^{-2}$	$4.5 \cdot 10^{-1}$	$1.4 \cdot 10^{1}$	$5.7 \cdot 10^{-3}$	$4.5 \cdot 10^{-3}$	$4.0 \cdot 10^{-2}$	$1.0 \cdot 10^{0}$	$2.5 \cdot 10^{1}$
PBE50	$3.5 \cdot 10^{-3}$	$3.6 \cdot 10^{-3}$	$5.2 \cdot 10^{-2}$	$8.2 \cdot 10^{-1}$	$2.5 \cdot 10^{1}$	$4.1 \cdot 10^{-3}$	$5.0 \cdot 10^{-3}$	$2.9 \cdot 10^{-2}$	$4.4 \cdot 10^{-1}$	$2.0 \cdot 10^{1}$
LC-wPBE	$3.7 \cdot 10^{-3}$	$4.1 \cdot 10^{-3}$	$4.1 \cdot 10^{-2}$	$6.1 \cdot 10^{-1}$	$2.3 \cdot 10^{1}$	$9.9 \cdot 10^{-3}$	$2.4 \cdot 10^{-2}$	$3.1 \cdot 10^{-1}$	$8.4 \cdot 10^{0}$	$4.7 \cdot 10^{1}$
TPSS	$2.8 \cdot 10^{-2}$	$1.5 \cdot 10^{-2}$	$6.2 \cdot 10^{-2}$	$7.6 \cdot 10^{-1}$	$1.8 \cdot 10^{1}$	$1.0 \cdot 10^{-2}$	$3.1 \cdot 10^{-1}$	$1.5 \cdot 10^{0}$	$2.7 \cdot 10^{1}$	$4.7 \cdot 10^{2}$
RevTPSS	$3.5 \cdot 10^{-3}$	$6.2 \cdot 10^{-3}$	$4.1 \cdot 10^{-2}$	$5.5 \cdot 10^{-1}$	$1.9 \cdot 10^{1}$	$8.0 \cdot 10^{-3}$	$1.0 \cdot 10^{-2}$	$5.1 \cdot 10^{-2}$	$1.5 \cdot 10^{0}$	$5.1 \cdot 10^{1}$
TPSSh	$3.8 \cdot 10^{-3}$	$5.1 \cdot 10^{-3}$	$4.2 \cdot 10^{-2}$	$4.0 \cdot 10^{-1}$	$2.2 \cdot 10^{1}$	$5.5 \cdot 10^{-3}$	$1.4 \cdot 10^{-2}$	$1.3 \cdot 10^{-1}$	$1.5 \cdot 10^{0}$	$6.4 \cdot 10^{1}$
B97	$9.7 \cdot 10^{-1}$	$1.2 \cdot 10^{1}$	$1.5 \cdot 10^{2}$	$2.0 \cdot 10^{3}$	$2.4 \cdot 10^{4}$	$3.5 \cdot 10^{-3}$	$8.9 \cdot 10^{-3}$	$4.3 \cdot 10^{-2}$	$8.3 \cdot 10^{-1}$	$2.5 \cdot 10^{1}$
B97-D	$7.1 \cdot 10^{-1}$	$8.9 \cdot 10^{0}$	$1.2 \cdot 10^{2}$	$1.5 \cdot 10^{3}$	$2.0 \cdot 10^{4}$	$8.7 \cdot 10^{-3}$	$2.6 \cdot 10^{-1}$	$2.2 \cdot 10^{0}$	$3.3 \cdot 10^{1}$	$5.9 \cdot 10^{2}$
wB97	$1.2 \cdot 10^{0}$	$1.1 \cdot 10^{1}$	$2.1 \cdot 10^{2}$	$4.7 \cdot 10^{3}$	$9.4 \cdot 10^{4}$	$4.3 \cdot 10^{-2}$	$2.5 \cdot 10^{-1}$	$2.5 \cdot 10^{0}$	$4.3 \cdot 10^{1}$	$7.3 \cdot 10^{2}$
wB97X	$1.8 \cdot 10^{0}$	$2.4 \cdot 10^{1}$	$3.8 \cdot 10^{2}$	$7.1 \cdot 10^{3}$	$1.3 \cdot 10^{5}$	$1.0 \cdot 10^{-2}$	$1.6 \cdot 10^{-2}$	$1.4 \cdot 10^{-1}$	$1.4 \cdot 10^{0}$	$7.4 \cdot 10^{1}$
wB97X-D	$3.1 \cdot 10^{0}$	$4.0 \cdot 10^{1}$	$6.9 \cdot 10^{2}$	$1.3 \cdot 10^{4}$	$2.4 \cdot 10^{5}$	$8.9 \cdot 10^{-3}$	$1.6 \cdot 10^{-2}$	$1.2 \cdot 10^{-1}$	$1.8 \cdot 10^{0}$	$9.1 \cdot 10^{1}$
wB97X-D3	$2.1 \cdot 10^{0}$	$2.7 \cdot 10^{1}$	$4.5 \cdot 10^{2}$	$8.1 \cdot 10^{3}$	$1.6 \cdot 10^{5}$	$8.4 \cdot 10^{-3}$	$1.4 \cdot 10^{-2}$	$1.1 \cdot 10^{-1}$	$1.3 \cdot 10^{0}$	$7.0 \cdot 10^{1}$
wB97M-V	$1.0 \cdot 10^{-1}$	$1.2 \cdot 10^{0}$	$1.7 \cdot 10^{1}$	$2.1 \cdot 10^{2}$	$3.0 \cdot 10^{3}$	$4.1 \cdot 10^{-3}$	$6.6 \cdot 10^{-3}$	$3.7 \cdot 10^{-2}$	$8.8 \cdot 10^{-1}$	$3.0 \cdot 10^{1}$
wB97X-V	$4.9 \cdot 10^{-2}$	$5.6 \cdot 10^{-1}$	$7.7 \cdot 10^{0}$	$1.2 \cdot 10^{2}$	$2.2 \cdot 10^{3}$	$3.3 \cdot 10^{-2}$	$9.7 \cdot 10^{-3}$	$4.9 \cdot 10^{-2}$	$1.0 \cdot 10^{0}$	$3.3 \cdot 10^{1}$
B97M-V	$9.0 \cdot 10^{-2}$	$8.1 \cdot 10^{-1}$	$9.0 \cdot 10^{0}$	$1.8 \cdot 10^{2}$	$3.9 \cdot 10^{3}$	$6.7 \cdot 10^{-3}$	$2.5 \cdot 10^{-1}$	$1.5 \cdot 10^{0}$	$2.8 \cdot 10^{1}$	$4.5 \cdot 10^{2}$
B1PW91	$1.3 \cdot 10^{-2}$	$1.5 \cdot 10^{-1}$	$3.3 \cdot 10^{0}$	$5.9 \cdot 10^{1}$	$1.3 \cdot 10^{3}$	$3.5 \cdot 10^{-3}$	$3.5 \cdot 10^{-3}$	$8.4 \cdot 10^{-2}$	$1.4 \cdot 10^{0}$	$2.1 \cdot 10^{1}$
PW91	$2.8 \cdot 10^{-2}$	$3.3 \cdot 10^{-2}$	$1.3 \cdot 10^{-1}$	$1.7 \cdot 10^{0}$	$5.0 \cdot 10^{1}$	$1.2 \cdot 10^{-1}$	$9.0 \cdot 10^{-1}$	$1.0 \cdot 10^{1}$	$2.2 \cdot 10^{2}$	$5.3 \cdot 10^{3}$
mPW91	$3.8 \cdot 10^{-2}$	$3.3 \cdot 10^{-2}$	$1.5 \cdot 10^{-1}$	$2.1 \cdot 10^{0}$	$3.9 \cdot 10^{1}$	$1.2 \cdot 10^{-1}$	$8.3 \cdot 10^{-1}$	$1.2 \cdot 10^{1}$	$2.5 \cdot 10^{2}$	$5.5 \cdot 10^{3}$
VSXC	$4.8 \cdot 10^{-2}$	$7.2 \cdot 10^{-1}$	$1.1 \cdot 10^{1}$	$1.7 \cdot 10^{2}$	$2.7 \cdot 10^{3}$	$2.1 \cdot 10^{-2}$	$3.3 \cdot 10^{-1}$	$4.7 \cdot 10^{0}$	$7.2 \cdot 10^{1}$	$1.1 \cdot 10^{3}$
SCAN	$4.4 \cdot 10^{0}$	$7.7 \cdot 10^{1}$	$9.8 \cdot 10^{2}$	$2.2 \cdot 10^{4}$	$6.1 \cdot 10^{5}$	$2.4 \cdot 10^{1}$	$2.9 \cdot 10^{2}$	$4.6 \cdot 10^{3}$	$1.0 \cdot 10^{5}$	$2.5 \cdot 10^{6}$
SCAN0	$4.5 \cdot 10^{0}$	$7.1 \cdot 10^{1}$	$1.2 \cdot 10^{3}$	$2.8 \cdot 10^{4}$	$9.1 \cdot 10^{5}$	$1.2 \cdot 10^{1}$	$2.2 \cdot 10^{2}$	$3.8 \cdot 10^{3}$	$8.5 \cdot 10^{4}$	$1.9 \cdot 10^{6}$
M06	$5.5 \cdot 10^{-1}$	$6.8 \cdot 10^{0}$	$9.6 \cdot 10^{1}$	$1.3 \cdot 10^{3}$	$1.8 \cdot 10^{4}$	$1.9 \cdot 10^{-1}$	$3.4 \cdot 10^{0}$	$6.5 \cdot 10^{1}$	$1.2 \cdot 10^{3}$	$2.2 \cdot 10^{4}$
M06-L	$6.4 \cdot 10^{-1}$	$8.6 \cdot 10^{0}$	$1.1 \cdot 10^{2}$	$1.5 \cdot 10^{3}$	$1.9 \cdot 10^{4}$	$6.0 \cdot 10^{-1}$	$1.1 \cdot 10^{1}$	$2.1 \cdot 10^{2}$	$3.9 \cdot 10^{3}$	$7.5 \cdot 10^{4}$
M06-HF	$9.9 \cdot 10^{-1}$	$1.1 \cdot 10^{1}$	$1.5 \cdot 10^{2}$	$1.6 \cdot 10^{3}$	$2.3 \cdot 10^{4}$	$1.4 \cdot 10^{0}$	$2.6 \cdot 10^{1}$	$4.3 \cdot 10^{2}$	$8.0 \cdot 10^{3}$	$1.3 \cdot 10^{5}$
M06-2X	$5.8 \cdot 10^{-1}$	$6.8 \cdot 10^{0}$	$9.6 \cdot 10^{1}$	$1.1 \cdot 10^{3}$	$1.6 \cdot 10^{4}$	$6.2 \cdot 10^{-1}$	$1.0 \cdot 10^{1}$	$1.9 \cdot 10^{2}$	$3.2 \cdot 10^{3}$	$5.8 \cdot 10^{4}$
M11	$9.0 \cdot 10^{-2}$	$1.5 \cdot 10^{0}$	$2.8 \cdot 10^{1}$	$4.7 \cdot 10^{2}$	$8.6 \cdot 10^{3}$	$2.8 \cdot 10^{-1}$	$5.3 \cdot 10^{0}$	$8.5 \cdot 10^{1}$	$1.5 \cdot 10^{3}$	$2.6 \cdot 10^{4}$
M11-L	$1.9 \cdot 10^{-1}$	$3.2 \cdot 10^{0}$	$4.9 \cdot 10^{1}$	$8.1 \cdot 10^{2}$	$1.3 \cdot 10^{4}$	$9.4 \cdot 10^{-1}$	$1.6 \cdot 10^{1}$	$2.5 \cdot 10^{2}$	$4.3 \cdot 10^{3}$	$6.4 \cdot 10^{4}$
SOGGA11	$5.8 \cdot 10^{-1}$	$7.0 \cdot 10^{0}$	$1.0 \cdot 10^{2}$	$1.6 \cdot 10^{3}$	$2.4 \cdot 10^{4}$	$4.7 \cdot 10^{-2}$	$6.8 \cdot 10^{-1}$	$1.1 \cdot 10^{1}$	$1.5 \cdot 10^{2}$	$2.4 \cdot 10^{3}$
SOGGA11-X	$1.3 \cdot 10^{0}$	$4.0 \cdot 10^{0}$	$6.9 \cdot 10^{1}$	$1.2 \cdot 10^{3}$	$2.1 \cdot 10^{4}$	$8.0 \cdot 10^{-3}$	$1.5 \cdot 10^{-2}$	$9.2 \cdot 10^{-2}$	$2.3 \cdot 10^{0}$	$9.7 \cdot 10^{1}$
N12	$2.7 \cdot 10^{0}$	$3.6 \cdot 10^{1}$	$5.2 \cdot 10^{2}$	$8.9 \cdot 10^{3}$	$1.6 \cdot 10^{5}$	$1.5 \cdot 10^{-2}$	$2.8 \cdot 10^{-1}$	$4.7 \cdot 10^{0}$	$8.2 \cdot 10^{1}$	$1.6 \cdot 10^{3}$
N12-SX	$7.3 \cdot 10^{-1}$	$7.7 \cdot 10^{0}$	$1.1 \cdot 10^{2}$	$1.5 \cdot 10^{3}$	$2.2 \cdot 10^{4}$	$1.7 \cdot 10^{-2}$	$2.8 \cdot 10^{-1}$	$4.3 \cdot 10^{0}$	$7.7 \cdot 10^{1}$	$1.4 \cdot 10^{3}$
MN12-L	$4.0 \cdot 10^{-2}$	$6.7 \cdot 10^{-1}$	$9.7 \cdot 10^{0}$	$1.7 \cdot 10^{2}$	$2.4 \cdot 10^{3}$	$9.1 \cdot 10^{-2}$	$1.6 \cdot 10^{0}$	$2.2 \cdot 10^{1}$	$3.8 \cdot 10^{2}$	$5.6 \cdot 10^{3}$
MN12-SX	$1.8 \cdot 10^{-1}$	$2.8 \cdot 10^{0}$	$4.7 \cdot 10^{1}$	$7.2 \cdot 10^{2}$	$1.2 \cdot 10^{4}$	$1.4 \cdot 10^{-1}$	$2.4 \cdot 10^{0}$	$3.5 \cdot 10^{1}$	$6.1 \cdot 10^{2}$	$8.8 \cdot 10^{3}$
MN15	$9.6 \cdot 10^{-3}$	$6.6 \cdot 10^{-1}$	$2.5 \cdot 10^{0}$	$3.8 \cdot 10^{1}$	$6.9 \cdot 10^{2}$	$2.0 \cdot 10^{-1}$	$1.6 \cdot 10^{-2}$	$5.9 \cdot 10^{-2}$	$7.2 \cdot 10^{-1}$	$1.0 \cdot 10^{1}$
MN15-L	$1.9 \cdot 10^{-2}$	$2.8 \cdot 10^{-1}$	$4.4 \cdot 10^{0}$	$6.5 \cdot 10^{1}$	$1.0 \cdot 10^{3}$	$1.2 \cdot 10^{-2}$	$4.7 \cdot 10^{-1}$	$1.5 \cdot 10^{0}$	$1.9 \cdot 10^{1}$	$2.6 \cdot 10^{2}$

Table S17: RMSE values for various energy derivatives obtained with the different functionals used in this work combined with the (99, 590) integration grid for the $N_2 \cdot HF$ system.

DFA	$d^nE_c/d\xi^n$ [$\times 10^{-8}$]					$d^nE_x/d\xi^n$ [$\times 10^{-8}$]				
	0	1	2	3	4	0	1	2	3	4
SVWN5	$8.5 \cdot 10^{-2}$	$3.3 \cdot 10^{-1}$	$1.8 \cdot 10^{0}$	$1.0 \cdot 10^{1}$	$6.2 \cdot 10^{1}$	$2.6 \cdot 10^{0}$	$4.3 \cdot 10^{0}$	$1.8 \cdot 10^{1}$	$9.6 \cdot 10^{1}$	$5.9 \cdot 10^{2}$
SPW92	$8.4 \cdot 10^{-2}$	$3.3 \cdot 10^{-1}$	$1.8 \cdot 10^{0}$	$9.8 \cdot 10^{0}$	$6.0 \cdot 10^{1}$	$2.6 \cdot 10^{0}$	$4.2 \cdot 10^{0}$	$1.7 \cdot 10^{1}$	$9.3 \cdot 10^{1}$	$5.5 \cdot 10^{2}$
BHH	$2.1 \cdot 10^{-1}$	$6.9 \cdot 10^{-1}$	$5.4 \cdot 10^{0}$	$4.1 \cdot 10^{1}$	$3.3 \cdot 10^{2}$	$2.4 \cdot 10^{0}$	$1.9 \cdot 10^{0}$	$1.1 \cdot 10^{1}$	$1.0 \cdot 10^{2}$	$6.2 \cdot 10^{2}$
BLYP	$3.1 \cdot 10^{-1}$	$5.8 \cdot 10^{-1}$	$2.7 \cdot 10^{0}$	$1.8 \cdot 10^{1}$	$9.7 \cdot 10^{1}$	$2.6 \cdot 10^{0}$	$1.3 \cdot 10^{1}$	$6.5 \cdot 10^{1}$	$3.0 \cdot 10^{2}$	$2.1 \cdot 10^{3}$
B1LYP	$2.4 \cdot 10^{-1}$	$6.0 \cdot 10^{-1}$	$2.6 \cdot 10^{0}$	$1.9 \cdot 10^{1}$	$8.8 \cdot 10^{1}$	$5.2 \cdot 10^{0}$	$6.6 \cdot 10^{0}$	$3.6 \cdot 10^{1}$	$1.5 \cdot 10^{2}$	$7.6 \cdot 10^{2}$
B3LYP	$1.4 \cdot 10^{-1}$	$5.3 \cdot 10^{-1}$	$2.7 \cdot 10^{0}$	$1.4 \cdot 10^{1}$	$1.1 \cdot 10^{2}$	$5.0 \cdot 10^{0}$	$6.6 \cdot 10^{0}$	$3.5 \cdot 10^{1}$	$1.4 \cdot 10^{2}$	$8.6 \cdot 10^{2}$
BHHLYP	$3.6 \cdot 10^{-1}$	$4.5 \cdot 10^{-1}$	$3.1 \cdot 10^{0}$	$1.6 \cdot 10^{1}$	$1.4 \cdot 10^{2}$	$3.1 \cdot 10^{0}$	$4.4 \cdot 10^{0}$	$2.4 \cdot 10^{1}$	$1.3 \cdot 10^{2}$	$7.5 \cdot 10^{2}$
LC-BLYP	$3.2 \cdot 10^{-2}$	$6.0 \cdot 10^{-1}$	$4.3 \cdot 10^{0}$	$2.7 \cdot 10^{1}$	$2.3 \cdot 10^{2}$	$4.9 \cdot 10^{0}$	$6.5 \cdot 10^{0}$	$3.5 \cdot 10^{1}$	$1.9 \cdot 10^{2}$	$1.7 \cdot 10^{3}$
CAMB3LYP	$1.1 \cdot 10^{-1}$	$6.3 \cdot 10^{-1}$	$2.5 \cdot 10^{0}$	$2.1 \cdot 10^{1}$	$1.3 \cdot 10^{2}$	$4.8 \cdot 10^{0}$	$4.7 \cdot 10^{0}$	$1.8 \cdot 10^{1}$	$1.5 \cdot 10^{2}$	$8.9 \cdot 10^{2}$
PBE	$1.3 \cdot 10^{0}$	$1.2 \cdot 10^{0}$	$1.0 \cdot 10^{1}$	$9.0 \cdot 10^{1}$	$7.6 \cdot 10^{2}$	$1.7 \cdot 10^{0}$	$9.4 \cdot 10^{0}$	$5.0 \cdot 10^{1}$	$3.8 \cdot 10^{2}$	$2.2 \cdot 10^{3}$
PBE0	$1.3 \cdot 10^{0}$	$3.8 \cdot 10^{0}$	$2.5 \cdot 10^{1}$	$1.3 \cdot 10^{2}$	$7.9 \cdot 10^{2}$	$5.7 \cdot 10^{0}$	$4.8 \cdot 10^{0}$	$3.0 \cdot 10^{1}$	$2.1 \cdot 10^{2}$	$1.3 \cdot 10^{3}$
PBE50	$1.1 \cdot 10^{0}$	$5.5 \cdot 10^{0}$	$3.5 \cdot 10^{1}$	$1.4 \cdot 10^{2}$	$1.0 \cdot 10^{3}$	$1.6 \cdot 10^{0}$	$2.3 \cdot 10^{0}$	$1.9 \cdot 10^{1}$	$1.0 \cdot 10^{2}$	$8.8 \cdot 10^{2}$
LC-wPBE	$1.1 \cdot 10^{0}$	$6.3 \cdot 10^{0}$	$3.0 \cdot 10^{1}$	$1.6 \cdot 10^{2}$	$1.0 \cdot 10^{3}$	$4.9 \cdot 10^{0}$	$9.1 \cdot 10^{0}$	$3.5 \cdot 10^{1}$	$2.1 \cdot 10^{2}$	$9.9 \cdot 10^{2}$
TPSS	$1.1 \cdot 10^{0}$	$2.4 \cdot 10^{0}$	$2.1 \cdot 10^{1}$	$1.8 \cdot 10^{2}$	$1.3 \cdot 10^{3}$	$1.2 \cdot 10^{1}$	$3.2 \cdot 10^{1}$	$1.5 \cdot 10^{2}$	$1.1 \cdot 10^{3}$	$6.6 \cdot 10^{3}$
RevTPSS	$1.1 \cdot 10^{0}$	$1.6 \cdot 10^{0}$	$1.7 \cdot 10^{1}$	$1.5 \cdot 10^{2}$	$1.2 \cdot 10^{3}$	$1.2 \cdot 10^{1}$	$2.6 \cdot 10^{1}$	$1.2 \cdot 10^{2}$	$1.1 \cdot 10^{3}$	$5.8 \cdot 10^{3}$
TPSSh	$3.0 \cdot 10^{-1}$	$3.3 \cdot 10^{0}$	$2.9 \cdot 10^{1}$	$1.9 \cdot 10^{2}$	$1.4 \cdot 10^{3}$	$6.5 \cdot 10^{0}$	$2.6 \cdot 10^{1}$	$1.5 \cdot 10^{2}$	$7.6 \cdot 10^{2}$	$6.7 \cdot 10^{3}$
B97	$6.3 \cdot 10^{1}$	$3.8 \cdot 10^{2}$	$3.5 \cdot 10^{3}$	$2.1 \cdot 10^{4}$	$2.2 \cdot 10^{5}$	$5.7 \cdot 10^{0}$	$6.6 \cdot 10^{0}$	$2.3 \cdot 10^{1}$	$1.3 \cdot 10^{2}$	$7.0 \cdot 10^{2}$
B97-D	$4.7 \cdot 10^{1}$	$2.8 \cdot 10^{2}$	$1.6 \cdot 10^{3}$	$1.2 \cdot 10^{4}$	$9.2 \cdot 10^{4}$	$2.8 \cdot 10^{0}$	$1.7 \cdot 10^{1}$	$9.4 \cdot 10^{1}$	$4.2 \cdot 10^{2}$	$2.6 \cdot 10^{3}$
wB97	$3.1 \cdot 10^{1}$	$3.6 \cdot 10^{2}$	$3.2 \cdot 10^{3}$	$3.3 \cdot 10^{4}$	$5.0 \cdot 10^{5}$	$6.1 \cdot 10^{0}$	$1.1 \cdot 10^{1}$	$3.1 \cdot 10^{1}$	$2.5 \cdot 10^{2}$	$1.1 \cdot 10^{3}$
wB97X	$3.8 \cdot 10^{1}$	$3.7 \cdot 10^{2}$	$3.1 \cdot 10^{3}$	$4.0 \cdot 10^{4}$	$6.7 \cdot 10^{5}$	$4.9 \cdot 10^{0}$	$6.6 \cdot 10^{0}$	$3.7 \cdot 10^{1}$	$2.7 \cdot 10^{2}$	$1.5 \cdot 10^{3}$
wB97X-D	$1.0 \cdot 10^{2}$	$6.3 \cdot 10^{2}$	$6.2 \cdot 10^{3}$	$6.0 \cdot 10^{4}$	$1.0 \cdot 10^{6}$	$3.1 \cdot 10^{0}$	$4.7 \cdot 10^{0}$	$3.1 \cdot 10^{1}$	$1.6 \cdot 10^{2}$	$1.2 \cdot 10^{3}$
wB97X-D3	$7.3 \cdot 10^{1}$	$4.0 \cdot 10^{2}$	$4.2 \cdot 10^{3}$	$4.1 \cdot 10^{4}$	$7.2 \cdot 10^{5}$	$3.2 \cdot 10^{0}$	$5.0 \cdot 10^{0}$	$2.9 \cdot 10^{1}$	$1.8 \cdot 10^{2}$	$1.2 \cdot 10^{3}$
wB97M-V	$7.7 \cdot 10^{0}$	$6.7 \cdot 10^{1}$	$4.8 \cdot 10^{2}$	$4.4 \cdot 10^{3}$	$3.0 \cdot 10^{4}$	$5.8 \cdot 10^{0}$	$8.3 \cdot 10^{0}$	$4.8 \cdot 10^{1}$	$2.8 \cdot 10^{2}$	$1.8 \cdot 10^{3}$
wB97X-V	$6.2 \cdot 10^{0}$	$3.3 \cdot 10^{1}$	$3.0 \cdot 10^{2}$	$2.2 \cdot 10^{3}$	$1.7 \cdot 10^{4}$	$5.6 \cdot 10^{0}$	$8.1 \cdot 10^{0}$	$2.5 \cdot 10^{1}$	$1.4 \cdot 10^{2}$	$6.7 \cdot 10^{2}$
B97M-V	$7.1 \cdot 10^{0}$	$2.9 \cdot 10^{1}$	$1.8 \cdot 10^{2}$	$1.7 \cdot 10^{3}$	$1.1 \cdot 10^{4}$	$3.1 \cdot 10^{0}$	$2.3 \cdot 10^{1}$	$1.2 \cdot 10^{2}$	$7.3 \cdot 10^{2}$	$4.2 \cdot 10^{3}$
B1PW91	$2.6 \cdot 10^{0}$	$1.2 \cdot 10^{1}$	$5.4 \cdot 10^{1}$	$3.3 \cdot 10^{2}$	$2.9 \cdot 10^{3}$	$3.7 \cdot 10^{0}$	$7.5 \cdot 10^{0}$	$3.3 \cdot 10^{1}$	$1.5 \cdot 10^{2}$	$7.0 \cdot 10^{2}$
PW91	$6.0 \cdot 10^{-1}$	$4.3 \cdot 10^{0}$	$2.1 \cdot 10^{1}$	$2.0 \cdot 10^{2}$	$2.7 \cdot 10^{3}$	$2.1 \cdot 10^{0}$	$1.2 \cdot 10^{1}$	$7.6 \cdot 10^{1}$	$1.2 \cdot 10^{3}$	$1.9 \cdot 10^{4}$
mPW91	$1.4 \cdot 10^{0}$	$5.9 \cdot 10^{0}$	$4.0 \cdot 10^{1}$	$2.4 \cdot 10^{2}$	$2.6 \cdot 10^{3}$	$2.9 \cdot 10^{0}$	$1.0 \cdot 10^{1}$	$4.2 \cdot 10^{1}$	$8.2 \cdot 10^{2}$	$1.6 \cdot 10^{4}$
VSXC	$1.0 \cdot 10^{2}$	$4.1 \cdot 10^{2}$	$2.0 \cdot 10^{3}$	$1.1 \cdot 10^{4}$	$4.8 \cdot 10^{4}$	$2.6 \cdot 10^{1}$	$2.7 \cdot 10^{1}$	$4.7 \cdot 10^{1}$	$4.9 \cdot 10^{2}$	$1.8 \cdot 10^{3}$
SCAN	$8.9 \cdot 10^{1}$	$2.2 \cdot 10^{2}$	$2.6 \cdot 10^{3}$	$4.5 \cdot 10^{4}$	$1.1 \cdot 10^{6}$	$2.4 \cdot 10^{2}$	$1.6 \cdot 10^{3}$	$1.6 \cdot 10^{4}$	$1.5 \cdot 10^{5}$	$3.5 \cdot 10^{6}$
SCAN0	$6.8 \cdot 10^{1}$	$3.2 \cdot 10^{2}$	$2.7 \cdot 10^{3}$	$4.3 \cdot 10^{4}$	$1.2 \cdot 10^{6}$	$2.1 \cdot 10^{2}$	$1.3 \cdot 10^{3}$	$1.2 \cdot 10^{4}$	$1.2 \cdot 10^{5}$	$2.7 \cdot 10^{6}$
M06	$2.2 \cdot 10^{1}$	$2.1 \cdot 10^{2}$	$1.0 \cdot 10^{3}$	$1.0 \cdot 10^{4}$	$1.0 \cdot 10^{5}$	$3.3 \cdot 10^{1}$	$9.8 \cdot 10^{1}$	$9.1 \cdot 10^{2}$	$5.7 \cdot 10^{3}$	$6.5 \cdot 10^{4}$
M06-L	$3.0 \cdot 10^{1}$	$4.3 \cdot 10^{2}$	$3.6 \cdot 10^{3}$	$2.9 \cdot 10^{4}$	$2.9 \cdot 10^{5}$	$4.3 \cdot 10^{1}$	$8.7 \cdot 10^{1}$	$8.1 \cdot 10^{2}$	$8.3 \cdot 10^{3}$	$8.7 \cdot 10^{4}$
M06-HF	$9.5 \cdot 10^{1}$	$6.0 \cdot 10^{2}$	$5.2 \cdot 10^{3}$	$3.2 \cdot 10^{4}$	$2.9 \cdot 10^{5}$	$4.7 \cdot 10^{1}$	$1.7 \cdot 10^{2}$	$1.1 \cdot 10^{3}$	$1.1 \cdot 10^{4}$	$1.1 \cdot 10^{5}$
M06-2X	$5.0 \cdot 10^{1}$	$3.3 \cdot 10^{2}$	$2.0 \cdot 10^{3}$	$1.2 \cdot 10^{4}$	$8.3 \cdot 10^{4}$	$1.7 \cdot 10^{1}$	$1.1 \cdot 10^{2}$	$4.5 \cdot 10^{2}$	$4.0 \cdot 10^{3}$	$5.0 \cdot 10^{4}$
M11	$1.7 \cdot 10^{1}$	$9.0 \cdot 10^{1}$	$3.0 \cdot 10^{2}$	$2.1 \cdot 10^{3}$	$1.4 \cdot 10^{4}$	$2.7 \cdot 10^{1}$	$8.8 \cdot 10^{1}$	$5.7 \cdot 10^{2}$	$4.9 \cdot 10^{3}$	$4.2 \cdot 10^{4}$
M11-L	$3.0 \cdot 10^{1}$	$1.2 \cdot 10^{2}$	$7.4 \cdot 10^{2}$	$4.6 \cdot 10^{3}$	$2.8 \cdot 10^{4}$	$3.5 \cdot 10^{1}$	$1.2 \cdot 10^{2}$	$9.5 \cdot 10^{2}$	$7.9 \cdot 10^{3}$	$7.7 \cdot 10^{4}$
SOGGA11	$1.8 \cdot 10^{2}$	$8.6 \cdot 10^{2}$	$4.1 \cdot 10^{3}$	$2.0 \cdot 10^{4}$	$1.3 \cdot 10^{5}$	$3.5 \cdot 10^{1}$	$5.0 \cdot 10^{1}$	$1.5 \cdot 10^{2}$	$6.6 \cdot 10^{2}$	$6.9 \cdot 10^{3}$
SOGGA11-X	$6.7 \cdot 10^{1}$	$3.4 \cdot 10^{1}$	$2.6 \cdot 10^{2}$	$1.9 \cdot 10^{3}$	$2.0 \cdot 10^{4}$	$2.4 \cdot 10^{0}$	$2.6 \cdot 10^{0}$	$2.8 \cdot 10^{1}$	$1.1 \cdot 10^{2}$	$9.3 \cdot 10^{2}$
N12	$1.2 \cdot 10^{2}$	$1.0 \cdot 10^{3}$	$9.7 \cdot 10^{3}$	$9.5 \cdot 10^{4}$	$1.3 \cdot 10^{6}$	$2.8 \cdot 10^{1}$	$2.3 \cdot 10^{1}$	$1.4 \cdot 10^{2}$	$8.5 \cdot 10^{2}$	$5.6 \cdot 10^{3}$
N12-SX	$2.4 \cdot 10^{1}$	$1.3 \cdot 10^{2}$	$1.1 \cdot 10^{3}$	$7.6 \cdot 10^{3}$	$8.3 \cdot 10^{4}$	$1.8 \cdot 10^{1}$	$2.6 \cdot 10^{1}$	$1.0 \cdot 10^{2}$	$6.3 \cdot 10^{2}$	$4.9 \cdot 10^{3}$
MN12-L	$3.7 \cdot 10^{1}$	$1.1 \cdot 10^{2}$	$6.7 \cdot 10^{2}$	$3.7 \cdot 10^{3}$	$2.4 \cdot 10^{4}$	$5.6 \cdot 10^{1}$	$6.0 \cdot 10^{1}$	$4.9 \cdot 10^{2}$	$3.3 \cdot 10^{3}$	$2.7 \cdot 10^{4}$
MN12-SX	$4.9 \cdot 10^{1}$	$1.3 \cdot 10^{2}$	$5.0 \cdot 10^{2}$	$3.5 \cdot 10^{3}$	$1.7 \cdot 10^{4}$	$4.8 \cdot 10^{1}$	$1.0 \cdot 10^{2}$	$1.3 \cdot 10^{2}$	$1.6 \cdot 10^{3}$	$1.7 \cdot 10^{4}$
MN15	$1.2 \cdot 10^{1}$	$9.7 \cdot 10^{1}$	$5.1 \cdot 10^{2}$	$3.9 \cdot 10^{3}$	$2.1 \cdot 10^{4}$	$6.1 \cdot 10^{0}$	$5.9 \cdot 10^{1}$	$3.2 \cdot 10^{2}$	$2.6 \cdot 10^{3}$	$1.4 \cdot 10^{4}$
MN15-L	$2.5 \cdot 10^{1}$	$1.4 \cdot 10^{2}$	$7.5 \cdot 10^{2}$	$3.7 \cdot 10^{3}$	$2.1 \cdot 10^{4}$	$1.4 \cdot 10^{1}$	$1.6 \cdot 10^{1}$	$1.7 \cdot 10^{2}$	$8.9 \cdot 10^{2}$	$7.1 \cdot 10^{3}$

Table S18: RMSE values for various energy derivatives obtained with the different functionals used in this work combined with the (250, 974) integration grid for the $N_2 \cdot HF$ system.

**DFA**	$d^nE_c/d\xi^n$ [$\times 10^{-8}$] 0	1	2	3	4	$d^nE_x/d\xi^n$ [$\times 10^{-8}$] 0	1	2	3	4
SVWN5	$2.9 \cdot 10^{-3}$	$1.7 \cdot 10^{-3}$	$2.5 \cdot 10^{-2}$	$2.8 \cdot 10^{-1}$	$9.2 \cdot 10^{0}$	$3.2 \cdot 10^{-3}$	$6.0 \cdot 10^{-3}$	$2.0 \cdot 10^{-2}$	$6.2 \cdot 10^{-1}$	$1.6 \cdot 10^{1}$
SPW92	$2.7 \cdot 10^{-3}$	$1.1 \cdot 10^{-3}$	$2.6 \cdot 10^{-2}$	$5.6 \cdot 10^{-1}$	$1.3 \cdot 10^{1}$	$2.7 \cdot 10^{-3}$	$4.7 \cdot 10^{-3}$	$2.8 \cdot 10^{-2}$	$3.5 \cdot 10^{-1}$	$1.2 \cdot 10^{1}$
BHH	$2.9 \cdot 10^{-3}$	$2.0 \cdot 10^{-3}$	$4.2 \cdot 10^{-2}$	$5.1 \cdot 10^{-1}$	$6.4 \cdot 10^{0}$	$2.8 \cdot 10^{-3}$	$3.7 \cdot 10^{-3}$	$2.7 \cdot 10^{-2}$	$5.2 \cdot 10^{-1}$	$7.2 \cdot 10^{0}$
BLYP	$2.7 \cdot 10^{-3}$	$1.3 \cdot 10^{-3}$	$1.6 \cdot 10^{-2}$	$3.4 \cdot 10^{-1}$	$9.1 \cdot 10^{0}$	$6.2 \cdot 10^{-3}$	$4.0 \cdot 10^{-3}$	$1.7 \cdot 10^{-1}$	$3.8 \cdot 10^{0}$	$7.1 \cdot 10^{1}$
B1LYP	$2.6 \cdot 10^{-3}$	$1.7 \cdot 10^{-3}$	$2.0 \cdot 10^{-2}$	$4.2 \cdot 10^{-1}$	$8.9 \cdot 10^{0}$	$6.0 \cdot 10^{-3}$	$5.5 \cdot 10^{-3}$	$2.2 \cdot 10^{-1}$	$4.4 \cdot 10^{0}$	$6.6 \cdot 10^{1}$
B3LYP	$2.8 \cdot 10^{-3}$	$1.1 \cdot 10^{-3}$	$3.7 \cdot 10^{-2}$	$6.8 \cdot 10^{-1}$	$1.0 \cdot 10^{1}$	$4.5 \cdot 10^{-3}$	$4.2 \cdot 10^{-3}$	$1.5 \cdot 10^{-1}$	$2.6 \cdot 10^{0}$	$4.8 \cdot 10^{1}$
BHHLYP	$3.1 \cdot 10^{-3}$	$2.2 \cdot 10^{-3}$	$3.6 \cdot 10^{-2}$	$6.0 \cdot 10^{-1}$	$1.0 \cdot 10^{1}$	$4.2 \cdot 10^{-3}$	$6.4 \cdot 10^{-3}$	$1.8 \cdot 10^{-1}$	$3.2 \cdot 10^{0}$	$4.7 \cdot 10^{1}$
LC-BLYP	$2.9 \cdot 10^{-3}$	$2.1 \cdot 10^{-3}$	$2.4 \cdot 10^{-2}$	$5.0 \cdot 10^{-1}$	$1.0 \cdot 10^{1}$	$3.4 \cdot 10^{-3}$	$7.9 \cdot 10^{-3}$	$4.5 \cdot 10^{-2}$	$4.8 \cdot 10^{-1}$	$7.2 \cdot 10^{0}$
CAMB3LYP	$2.8 \cdot 10^{-3}$	$2.7 \cdot 10^{-3}$	$3.5 \cdot 10^{-2}$	$5.0 \cdot 10^{-1}$	$4.7 \cdot 10^{0}$	$3.7 \cdot 10^{-3}$	$2.7 \cdot 10^{-3}$	$8.6 \cdot 10^{-2}$	$1.4 \cdot 10^{0}$	$2.3 \cdot 10^{1}$
PBE	$7.6 \cdot 10^{-3}$	$7.5 \cdot 10^{-3}$	$5.7 \cdot 10^{-2}$	$6.4 \cdot 10^{-1}$	$2.8 \cdot 10^{1}$	$5.4 \cdot 10^{-3}$	$9.4 \cdot 10^{-3}$	$6.7 \cdot 10^{-2}$	$4.7 \cdot 10^{-1}$	$2.5 \cdot 10^{1}$
PBE0	$4.2 \cdot 10^{-3}$	$7.8 \cdot 10^{-3}$	$4.7 \cdot 10^{-2}$	$9.8 \cdot 10^{-1}$	$3.8 \cdot 10^{1}$	$3.0 \cdot 10^{-3}$	$7.6 \cdot 10^{-3}$	$6.1 \cdot 10^{-2}$	$5.0 \cdot 10^{-1}$	$1.6 \cdot 10^{1}$
PBE50	$5.7 \cdot 10^{-3}$	$4.1 \cdot 10^{-3}$	$3.5 \cdot 10^{-2}$	$5.8 \cdot 10^{-1}$	$2.3 \cdot 10^{1}$	$3.7 \cdot 10^{-3}$	$3.1 \cdot 10^{-3}$	$2.1 \cdot 10^{-2}$	$3.9 \cdot 10^{-1}$	$1.4 \cdot 10^{1}$
LC-wPBE	$6.3 \cdot 10^{-3}$	$5.6 \cdot 10^{-3}$	$4.8 \cdot 10^{-2}$	$1.3 \cdot 10^{0}$	$3.8 \cdot 10^{1}$	$4.8 \cdot 10^{-3}$	$2.8 \cdot 10^{-1}$	$4.1 \cdot 10^{-1}$	$1.8 \cdot 10^{0}$	$6.0 \cdot 10^{1}$
TPSS	$6.3 \cdot 10^{-3}$	$4.9 \cdot 10^{-3}$	$4.1 \cdot 10^{-2}$	$5.4 \cdot 10^{-1}$	$2.6 \cdot 10^{1}$	$1.3 \cdot 10^{-2}$	$7.3 \cdot 10^{-1}$	$3.3 \cdot 10^{0}$	$6.0 \cdot 10^{1}$	$9.1 \cdot 10^{2}$
RevTPSS	$4.2 \cdot 10^{-3}$	$4.3 \cdot 10^{-3}$	$3.7 \cdot 10^{-2}$	$5.3 \cdot 10^{-1}$	$2.1 \cdot 10^{1}$	$1.1 \cdot 10^{-2}$	$8.5 \cdot 10^{-1}$	$2.9 \cdot 10^{0}$	$4.5 \cdot 10^{1}$	$8.1 \cdot 10^{2}$
TPSSh	$7.0 \cdot 10^{-3}$	$4.9 \cdot 10^{-3}$	$4.0 \cdot 10^{-2}$	$1.0 \cdot 10^{0}$	$3.5 \cdot 10^{1}$	$1.2 \cdot 10^{-2}$	$6.2 \cdot 10^{-1}$	$3.1 \cdot 10^{0}$	$4.8 \cdot 10^{1}$	$8.7 \cdot 10^{2}$
B97	$2.2 \cdot 10^{0}$	$2.5 \cdot 10^{1}$	$3.5 \cdot 10^{2}$	$4.3 \cdot 10^{3}$	$6.8 \cdot 10^{4}$	$3.8 \cdot 10^{-3}$	$7.8 \cdot 10^{-3}$	$4.4 \cdot 10^{-2}$	$1.3 \cdot 10^{0}$	$3.4 \cdot 10^{1}$
B97-D	$1.3 \cdot 10^{0}$	$1.5 \cdot 10^{1}$	$1.7 \cdot 10^{2}$	$2.4 \cdot 10^{3}$	$3.2 \cdot 10^{4}$	$1.0 \cdot 10^{-2}$	$1.7 \cdot 10^{-1}$	$1.9 \cdot 10^{0}$	$2.8 \cdot 10^{1}$	$4.6 \cdot 10^{2}$
wB97	$1.7 \cdot 10^{0}$	$3.0 \cdot 10^{1}$	$6.2 \cdot 10^{2}$	$1.2 \cdot 10^{4}$	$2.7 \cdot 10^{5}$	$8.0 \cdot 10^{-3}$	$2.3 \cdot 10^{-1}$	$2.2 \cdot 10^{0}$	$3.9 \cdot 10^{1}$	$6.7 \cdot 10^{2}$
wB97X	$2.2 \cdot 10^{0}$	$4.2 \cdot 10^{1}$	$8.8 \cdot 10^{2}$	$1.8 \cdot 10^{4}$	$3.7 \cdot 10^{5}$	$4.4 \cdot 10^{-3}$	$1.0 \cdot 10^{-2}$	$8.1 \cdot 10^{-2}$	$1.0 \cdot 10^{0}$	$4.9 \cdot 10^{1}$
wB97X-D	$5.0 \cdot 10^{0}$	$9.2 \cdot 10^{1}$	$1.9 \cdot 10^{3}$	$3.5 \cdot 10^{4}$	$7.2 \cdot 10^{5}$	$5.4 \cdot 10^{-3}$	$1.2 \cdot 10^{-2}$	$8.5 \cdot 10^{-2}$	$1.5 \cdot 10^{0}$	$6.1 \cdot 10^{1}$
wB97X-D3	$3.0 \cdot 10^{0}$	$6.1 \cdot 10^{1}$	$1.2 \cdot 10^{3}$	$2.4 \cdot 10^{4}$	$4.6 \cdot 10^{5}$	$3.5 \cdot 10^{-3}$	$6.6 \cdot 10^{-3}$	$3.7 \cdot 10^{-2}$	$9.7 \cdot 10^{-1}$	$2.9 \cdot 10^{1}$
wB97M-V	$7.7 \cdot 10^{-2}$	$7.8 \cdot 10^{-1}$	$1.2 \cdot 10^{1}$	$1.7 \cdot 10^{2}$	$3.2 \cdot 10^{3}$	$3.7 \cdot 10^{-3}$	$4.6 \cdot 10^{-3}$	$3.1 \cdot 10^{-2}$	$9.3 \cdot 10^{-1}$	$3.1 \cdot 10^{1}$
wB97X-V	$1.1 \cdot 10^{-1}$	$1.5 \cdot 10^{0}$	$1.7 \cdot 10^{1}$	$2.3 \cdot 10^{2}$	$2.8 \cdot 10^{3}$	$4.6 \cdot 10^{-3}$	$8.4 \cdot 10^{-3}$	$7.3 \cdot 10^{-2}$	$1.5 \cdot 10^{0}$	$3.2 \cdot 10^{1}$
B97M-V	$3.5 \cdot 10^{-2}$	$5.0 \cdot 10^{-1}$	$7.0 \cdot 10^{0}$	$1.2 \cdot 10^{2}$	$2.2 \cdot 10^{3}$	$9.0 \cdot 10^{-3}$	$7.9 \cdot 10^{-1}$	$2.3 \cdot 10^{0}$	$3.9 \cdot 10^{1}$	$6.2 \cdot 10^{2}$
B1PW91	$1.5 \cdot 10^{-2}$	$2.8 \cdot 10^{-1}$	$4.8 \cdot 10^{0}$	$8.5 \cdot 10^{1}$	$1.7 \cdot 10^{3}$	$6.0 \cdot 10^{-3}$	$7.9 \cdot 10^{-3}$	$2.7 \cdot 10^{-1}$	$4.1 \cdot 10^{0}$	$6.6 \cdot 10^{1}$
PW91	$1.7 \cdot 10^{-2}$	$3.2 \cdot 10^{-1}$	$6.5 \cdot 10^{0}$	$1.4 \cdot 10^{2}$	$3.2 \cdot 10^{3}$	$9.4 \cdot 10^{-2}$	$1.8 \cdot 10^{0}$	$4.1 \cdot 10^{1}$	$8.4 \cdot 10^{2}$	$1.9 \cdot 10^{4}$
mPW91	$1.5 \cdot 10^{-2}$	$3.3 \cdot 10^{-1}$	$5.7 \cdot 10^{0}$	$1.2 \cdot 10^{2}$	$2.5 \cdot 10^{3}$	$1.0 \cdot 10^{-1}$	$2.1 \cdot 10^{0}$	$3.8 \cdot 10^{1}$	$7.7 \cdot 10^{2}$	$1.5 \cdot 10^{4}$
VSXC	$4.3 \cdot 10^{-2}$	$7.0 \cdot 10^{-1}$	$8.9 \cdot 10^{0}$	$1.3 \cdot 10^{2}$	$1.9 \cdot 10^{3}$	$2.0 \cdot 10^{-2}$	$3.3 \cdot 10^{-1}$	$4.0 \cdot 10^{0}$	$6.0 \cdot 10^{1}$	$8.8 \cdot 10^{2}$
SCAN	$2.3 \cdot 10^{0}$	$3.3 \cdot 10^{1}$	$4.8 \cdot 10^{2}$	$1.5 \cdot 10^{4}$	$6.2 \cdot 10^{5}$	$5.8 \cdot 10^{0}$	$9.2 \cdot 10^{1}$	$1.7 \cdot 10^{3}$	$4.2 \cdot 10^{4}$	$1.6 \cdot 10^{6}$
SCAN0	$3.0 \cdot 10^{0}$	$3.4 \cdot 10^{1}$	$5.8 \cdot 10^{2}$	$1.6 \cdot 10^{4}$	$6.6 \cdot 10^{5}$	$4.1 \cdot 10^{0}$	$5.0 \cdot 10^{1}$	$9.0 \cdot 10^{2}$	$2.9 \cdot 10^{4}$	$1.4 \cdot 10^{6}$
M06	$1.1 \cdot 10^{0}$	$1.3 \cdot 10^{1}$	$1.8 \cdot 10^{2}$	$2.2 \cdot 10^{3}$	$3.1 \cdot 10^{4}$	$1.5 \cdot 10^{-1}$	$2.7 \cdot 10^{0}$	$4.6 \cdot 10^{1}$	$8.8 \cdot 10^{2}$	$1.5 \cdot 10^{4}$
M06-L	$1.6 \cdot 10^{0}$	$2.3 \cdot 10^{1}$	$2.7 \cdot 10^{2}$	$3.8 \cdot 10^{3}$	$4.4 \cdot 10^{4}$	$4.7 \cdot 10^{-1}$	$8.1 \cdot 10^{0}$	$1.5 \cdot 10^{2}$	$2.6 \cdot 10^{3}$	$4.8 \cdot 10^{4}$
M06-HF	$2.5 \cdot 10^{0}$	$2.8 \cdot 10^{1}$	$3.6 \cdot 10^{2}$	$4.0 \cdot 10^{3}$	$5.0 \cdot 10^{4}$	$1.0 \cdot 10^{0}$	$1.7 \cdot 10^{1}$	$2.5 \cdot 10^{2}$	$4.3 \cdot 10^{3}$	$6.2 \cdot 10^{4}$
M06-2X	$1.0 \cdot 10^{0}$	$1.2 \cdot 10^{1}$	$1.6 \cdot 10^{2}$	$1.9 \cdot 10^{3}$	$2.6 \cdot 10^{4}$	$4.5 \cdot 10^{-1}$	$8.3 \cdot 10^{0}$	$1.3 \cdot 10^{2}$	$2.3 \cdot 10^{3}$	$3.6 \cdot 10^{4}$
M11	$5.6 \cdot 10^{-2}$	$8.5 \cdot 10^{-1}$	$1.5 \cdot 10^{1}$	$2.4 \cdot 10^{2}$	$4.2 \cdot 10^{3}$	$2.7 \cdot 10^{-1}$	$4.4 \cdot 10^{0}$	$7.6 \cdot 10^{1}$	$1.2 \cdot 10^{3}$	$2.2 \cdot 10^{4}$
M11-L	$1.4 \cdot 10^{-1}$	$2.2 \cdot 10^{0}$	$3.8 \cdot 10^{1}$	$5.9 \cdot 10^{2}$	$1.0 \cdot 10^{4}$	$5.8 \cdot 10^{-1}$	$9.6 \cdot 10^{0}$	$1.6 \cdot 10^{2}$	$2.6 \cdot 10^{3}$	$4.3 \cdot 10^{4}$
SOGGA11	$8.4 \cdot 10^{-1}$	$8.8 \cdot 10^{0}$	$1.2 \cdot 10^{2}$	$1.2 \cdot 10^{3}$	$1.6 \cdot 10^{4}$	$1.5 \cdot 10^{-2}$	$1.9 \cdot 10^{-1}$	$2.5 \cdot 10^{0}$	$3.2 \cdot 10^{1}$	$4.2 \cdot 10^{2}$
SOGGA11-X	$3.5 \cdot 10^{-1}$	$2.4 \cdot 10^{0}$	$3.6 \cdot 10^{1}$	$5.4 \cdot 10^{2}$	$1.0 \cdot 10^{4}$	$4.0 \cdot 10^{-3}$	$2.3 \cdot 10^{-1}$	$7.0 \cdot 10^{-2}$	$1.0 \cdot 10^{0}$	$4.1 \cdot 10^{1}$
N12	$3.9 \cdot 10^{0}$	$7.4 \cdot 10^{1}$	$1.6 \cdot 10^{3}$	$3.4 \cdot 10^{4}$	$7.4 \cdot 10^{5}$	$2.0 \cdot 10^{-2}$	$3.9 \cdot 10^{-1}$	$5.5 \cdot 10^{0}$	$1.0 \cdot 10^{2}$	$1.7 \cdot 10^{3}$
N12-SX	$1.1 \cdot 10^{0}$	$1.4 \cdot 10^{1}$	$1.7 \cdot 10^{2}$	$2.4 \cdot 10^{3}$	$3.4 \cdot 10^{4}$	$1.9 \cdot 10^{-2}$	$3.4 \cdot 10^{-1}$	$4.9 \cdot 10^{0}$	$8.7 \cdot 10^{1}$	$1.4 \cdot 10^{3}$
MN12-L	$3.9 \cdot 10^{-2}$	$6.6 \cdot 10^{-1}$	$6.7 \cdot 10^{0}$	$1.1 \cdot 10^{2}$	$1.6 \cdot 10^{3}$	$1.4 \cdot 10^{-1}$	$1.9 \cdot 10^{0}$	$3.4 \cdot 10^{1}$	$5.0 \cdot 10^{2}$	$8.7 \cdot 10^{3}$
MN12-SX	$1.1 \cdot 10^{-1}$	$1.8 \cdot 10^{0}$	$2.8 \cdot 10^{1}$	$4.8 \cdot 10^{2}$	$7.5 \cdot 10^{3}$	$2.7 \cdot 10^{-1}$	$2.6 \cdot 10^{0}$	$3.9 \cdot 10^{1}$	$6.7 \cdot 10^{2}$	$1.0 \cdot 10^{4}$
MN15	$2.1 \cdot 10^{-2}$	$5.7 \cdot 10^{-1}$	$5.0 \cdot 10^{0}$	$8.5 \cdot 10^{1}$	$1.3 \cdot 10^{3}$	$7.7 \cdot 10^{-3}$	$1.1 \cdot 10^{-2}$	$3.4 \cdot 10^{-2}$	$2.6 \cdot 10^{0}$	$8.6 \cdot 10^{1}$
MN15-L	$2.0 \cdot 10^{-2}$	$2.7 \cdot 10^{-1}$	$3.9 \cdot 10^{0}$	$5.7 \cdot 10^{1}$	$8.7 \cdot 10^{2}$	$2.6 \cdot 10^{-2}$	$4.2 \cdot 10^{-1}$	$3.9 \cdot 10^{0}$	$6.2 \cdot 10^{1}$	$8.6 \cdot 10^{2}$

Table S19: RMSE values for various energy derivatives obtained with the different functionals used in this work combined with the (99, 590) integration grid for the Ar_2 system.

**DFA**	$\mathrm{d}^n E_c/\mathrm{d}\xi^n$ [$\times 10^{-8}$] 0	1	2	3	4	$\mathrm{d}^n E_x/\mathrm{d}\xi^n$ [$\times 10^{-8}$] 0	1	2	3	4
SVWN5	$7.8 \cdot 10^{0}$	$7.4 \cdot 10^{1}$	$7.5 \cdot 10^{2}$	$7.4 \cdot 10^{3}$	$7.7 \cdot 10^{4}$	$8.9 \cdot 10^{1}$	$8.4 \cdot 10^{2}$	$8.7 \cdot 10^{3}$	$8.5 \cdot 10^{4}$	$9.2 \cdot 10^{5}$
SPW92	$7.7 \cdot 10^{0}$	$7.4 \cdot 10^{1}$	$7.5 \cdot 10^{2}$	$7.3 \cdot 10^{3}$	$7.7 \cdot 10^{4}$	$8.9 \cdot 10^{1}$	$8.4 \cdot 10^{2}$	$8.7 \cdot 10^{3}$	$8.5 \cdot 10^{4}$	$9.2 \cdot 10^{5}$
BHH	$8.8 \cdot 10^{0}$	$5.6 \cdot 10^{1}$	$5.5 \cdot 10^{2}$	$5.7 \cdot 10^{3}$	$6.9 \cdot 10^{4}$	$4.4 \cdot 10^{1}$	$4.2 \cdot 10^{2}$	$4.4 \cdot 10^{3}$	$4.3 \cdot 10^{4}$	$4.6 \cdot 10^{5}$
BLYP	$8.8 \cdot 10^{0}$	$5.6 \cdot 10^{1}$	$5.6 \cdot 10^{2}$	$5.7 \cdot 10^{3}$	$7.0 \cdot 10^{4}$	$1.3 \cdot 10^{2}$	$1.3 \cdot 10^{3}$	$1.3 \cdot 10^{4}$	$1.3 \cdot 10^{5}$	$1.3 \cdot 10^{6}$
B1LYP	$8.8 \cdot 10^{0}$	$5.6 \cdot 10^{1}$	$5.6 \cdot 10^{2}$	$5.7 \cdot 10^{3}$	$7.0 \cdot 10^{4}$	$1.0 \cdot 10^{2}$	$1.0 \cdot 10^{3}$	$1.0 \cdot 10^{4}$	$9.9 \cdot 10^{4}$	$1.0 \cdot 10^{6}$
B3LYP	$8.8 \cdot 10^{0}$	$6.4 \cdot 10^{1}$	$6.3 \cdot 10^{2}$	$6.3 \cdot 10^{3}$	$7.4 \cdot 10^{4}$	$1.0 \cdot 10^{2}$	$1.0 \cdot 10^{3}$	$1.0 \cdot 10^{4}$	$1.0 \cdot 10^{5}$	$1.0 \cdot 10^{6}$
BHHLYP	$8.9 \cdot 10^{0}$	$5.7 \cdot 10^{1}$	$5.6 \cdot 10^{2}$	$5.7 \cdot 10^{3}$	$7.0 \cdot 10^{4}$	$6.9 \cdot 10^{1}$	$6.9 \cdot 10^{2}$	$6.9 \cdot 10^{3}$	$6.8 \cdot 10^{4}$	$7.0 \cdot 10^{5}$
LC-BLYP	$8.9 \cdot 10^{0}$	$5.6 \cdot 10^{1}$	$5.5 \cdot 10^{2}$	$5.7 \cdot 10^{3}$	$6.9 \cdot 10^{4}$	$6.8 \cdot 10^{1}$	$6.4 \cdot 10^{2}$	$6.6 \cdot 10^{3}$	$6.5 \cdot 10^{4}$	$7.0 \cdot 10^{5}$
CAMB3LYP	$8.4 \cdot 10^{0}$	$6.0 \cdot 10^{1}$	$5.9 \cdot 10^{2}$	$6.0 \cdot 10^{3}$	$7.0 \cdot 10^{4}$	$7.8 \cdot 10^{1}$	$7.7 \cdot 10^{2}$	$7.8 \cdot 10^{3}$	$7.6 \cdot 10^{4}$	$7.9 \cdot 10^{5}$
PBE	$9.1 \cdot 10^{1}$	$9.9 \cdot 10^{2}$	$9.3 \cdot 10^{3}$	$9.9 \cdot 10^{4}$	$9.8 \cdot 10^{5}$	$1.2 \cdot 10^{2}$	$1.2 \cdot 10^{3}$	$1.1 \cdot 10^{4}$	$1.1 \cdot 10^{5}$	$1.1 \cdot 10^{6}$
PBE0	$1.0 \cdot 10^{2}$	$1.1 \cdot 10^{3}$	$1.1 \cdot 10^{4}$	$1.1 \cdot 10^{5}$	$1.1 \cdot 10^{6}$	$9.5 \cdot 10^{1}$	$9.4 \cdot 10^{2}$	$8.9 \cdot 10^{3}$	$8.8 \cdot 10^{4}$	$8.6 \cdot 10^{5}$
PBE50	$1.2 \cdot 10^{2}$	$1.3 \cdot 10^{3}$	$1.2 \cdot 10^{4}$	$1.3 \cdot 10^{5}$	$1.2 \cdot 10^{6}$	$6.6 \cdot 10^{1}$	$6.6 \cdot 10^{2}$	$6.2 \cdot 10^{3}$	$6.2 \cdot 10^{4}$	$6.0 \cdot 10^{5}$
LC-wPBE	$1.0 \cdot 10^{2}$	$1.1 \cdot 10^{3}$	$1.1 \cdot 10^{4}$	$1.1 \cdot 10^{5}$	$1.1 \cdot 10^{6}$	$5.7 \cdot 10^{1}$	$5.6 \cdot 10^{2}$	$6.4 \cdot 10^{3}$	$6.5 \cdot 10^{4}$	$7.9 \cdot 10^{5}$
TPSS	$1.1 \cdot 10^{2}$	$1.2 \cdot 10^{3}$	$1.1 \cdot 10^{4}$	$1.2 \cdot 10^{5}$	$1.2 \cdot 10^{6}$	$1.0 \cdot 10^{2}$	$8.7 \cdot 10^{2}$	$8.6 \cdot 10^{3}$	$7.8 \cdot 10^{4}$	$1.2 \cdot 10^{6}$
RevTPSS	$6.7 \cdot 10^{1}$	$7.4 \cdot 10^{2}$	$6.9 \cdot 10^{3}$	$7.6 \cdot 10^{4}$	$7.6 \cdot 10^{5}$	$6.6 \cdot 10^{1}$	$5.8 \cdot 10^{2}$	$5.9 \cdot 10^{3}$	$5.6 \cdot 10^{4}$	$9.0 \cdot 10^{5}$
TPSSh	$1.1 \cdot 10^{2}$	$1.2 \cdot 10^{3}$	$1.1 \cdot 10^{4}$	$1.2 \cdot 10^{5}$	$1.2 \cdot 10^{6}$	$9.5 \cdot 10^{1}$	$8.2 \cdot 10^{2}$	$8.1 \cdot 10^{3}$	$7.2 \cdot 10^{4}$	$1.1 \cdot 10^{6}$
B97	$9.6 \cdot 10^{1}$	$1.2 \cdot 10^{3}$	$2.7 \cdot 10^{4}$	$8.5 \cdot 10^{5}$	$3.4 \cdot 10^{7}$	$6.8 \cdot 10^{1}$	$6.5 \cdot 10^{2}$	$6.3 \cdot 10^{3}$	$6.4 \cdot 10^{4}$	$6.4 \cdot 10^{5}$
B97-D	$5.2 \cdot 10^{2}$	$5.4 \cdot 10^{3}$	$5.6 \cdot 10^{4}$	$7.9 \cdot 10^{5}$	$2.4 \cdot 10^{7}$	$1.9 \cdot 10^{2}$	$2.0 \cdot 10^{3}$	$2.0 \cdot 10^{4}$	$2.1 \cdot 10^{5}$	$2.1 \cdot 10^{6}$
wB97	$3.2 \cdot 10^{2}$	$4.0 \cdot 10^{3}$	$4.9 \cdot 10^{4}$	$1.3 \cdot 10^{6}$	$6.0 \cdot 10^{7}$	$6.7 \cdot 10^{1}$	$6.3 \cdot 10^{2}$	$7.1 \cdot 10^{3}$	$6.9 \cdot 10^{4}$	$7.8 \cdot 10^{5}$
wB97X	$2.2 \cdot 10^{2}$	$2.8 \cdot 10^{3}$	$3.7 \cdot 10^{4}$	$1.2 \cdot 10^{6}$	$6.9 \cdot 10^{7}$	$7.7 \cdot 10^{1}$	$7.4 \cdot 10^{2}$	$7.2 \cdot 10^{3}$	$7.1 \cdot 10^{4}$	$7.5 \cdot 10^{5}$
wB97X-D	$2.1 \cdot 10^{2}$	$2.8 \cdot 10^{3}$	$5.3 \cdot 10^{4}$	$2.1 \cdot 10^{6}$	$1.2 \cdot 10^{8}$	$9.4 \cdot 10^{1}$	$9.0 \cdot 10^{2}$	$8.6 \cdot 10^{3}$	$8.4 \cdot 10^{4}$	$9.0 \cdot 10^{5}$
wB97X-D3	$1.7 \cdot 10^{2}$	$2.3 \cdot 10^{3}$	$3.7 \cdot 10^{4}$	$1.4 \cdot 10^{6}$	$8.2 \cdot 10^{7}$	$7.8 \cdot 10^{1}$	$7.6 \cdot 10^{2}$	$7.4 \cdot 10^{3}$	$7.3 \cdot 10^{4}$	$7.8 \cdot 10^{5}$
wB97M-V	$1.8 \cdot 10^{2}$	$2.0 \cdot 10^{3}$	$2.2 \cdot 10^{4}$	$3.5 \cdot 10^{5}$	$9.9 \cdot 10^{6}$	$1.1 \cdot 10^{2}$	$1.1 \cdot 10^{3}$	$1.2 \cdot 10^{4}$	$1.1 \cdot 10^{5}$	$1.2 \cdot 10^{6}$
wB97X-V	$7.5 \cdot 10^{1}$	$8.4 \cdot 10^{2}$	$9.4 \cdot 10^{3}$	$1.4 \cdot 10^{5}$	$3.6 \cdot 10^{6}$	$5.9 \cdot 10^{1}$	$2.8 \cdot 10^{3}$	$6.7 \cdot 10^{3}$	$5.6 \cdot 10^{4}$	$5.8 \cdot 10^{5}$
B97M-V	$6.6 \cdot 10^{1}$	$5.4 \cdot 10^{2}$	$6.8 \cdot 10^{3}$	$7.9 \cdot 10^{4}$	$1.5 \cdot 10^{6}$	$1.7 \cdot 10^{2}$	$1.8 \cdot 10^{3}$	$1.9 \cdot 10^{4}$	$2.0 \cdot 10^{5}$	$2.1 \cdot 10^{6}$
B1PW91	$1.1 \cdot 10^{2}$	$1.2 \cdot 10^{3}$	$1.1 \cdot 10^{4}$	$1.2 \cdot 10^{5}$	$1.2 \cdot 10^{6}$	$1.0 \cdot 10^{2}$	$1.0 \cdot 10^{3}$	$1.0 \cdot 10^{4}$	$1.0 \cdot 10^{5}$	$1.1 \cdot 10^{6}$
PW91	$9.0 \cdot 10^{1}$	$9.7 \cdot 10^{2}$	$9.3 \cdot 10^{3}$	$9.8 \cdot 10^{4}$	$9.7 \cdot 10^{5}$	$1.3 \cdot 10^{2}$	$3.3 \cdot 10^{3}$	$1.4 \cdot 10^{4}$	$1.3 \cdot 10^{5}$	$2.5 \cdot 10^{6}$
mPW91	$9.4 \cdot 10^{1}$	$1.0 \cdot 10^{3}$	$9.7 \cdot 10^{3}$	$1.0 \cdot 10^{5}$	$1.0 \cdot 10^{6}$	$1.4 \cdot 10^{2}$	$1.4 \cdot 10^{3}$	$1.4 \cdot 10^{4}$	$1.4 \cdot 10^{5}$	$2.5 \cdot 10^{6}$
VSXC	$4.2 \cdot 10^{3}$	$4.5 \cdot 10^{4}$	$4.3 \cdot 10^{5}$	$4.6 \cdot 10^{6}$	$4.5 \cdot 10^{7}$	$2.3 \cdot 10^{2}$	$2.6 \cdot 10^{3}$	$1.9 \cdot 10^{4}$	$2.4 \cdot 10^{5}$	$2.2 \cdot 10^{6}$
SCAN	$1.7 \cdot 10^{2}$	$1.6 \cdot 10^{3}$	$2.1 \cdot 10^{4}$	$5.1 \cdot 10^{5}$	$3.6 \cdot 10^{7}$	$5.7 \cdot 10^{2}$	$5.3 \cdot 10^{3}$	$7.9 \cdot 10^{4}$	$1.5 \cdot 10^{6}$	$1.0 \cdot 10^{8}$
SCAN0	$1.7 \cdot 10^{2}$	$1.6 \cdot 10^{3}$	$2.1 \cdot 10^{4}$	$5.2 \cdot 10^{5}$	$3.6 \cdot 10^{7}$	$4.3 \cdot 10^{2}$	$4.0 \cdot 10^{3}$	$6.0 \cdot 10^{4}$	$1.2 \cdot 10^{6}$	$7.8 \cdot 10^{7}$
M06	$4.6 \cdot 10^{3}$	$4.7 \cdot 10^{4}$	$4.6 \cdot 10^{5}$	$4.9 \cdot 10^{6}$	$6.2 \cdot 10^{7}$	$2.9 \cdot 10^{2}$	$1.9 \cdot 10^{3}$	$1.9 \cdot 10^{4}$	$3.1 \cdot 10^{5}$	$7.0 \cdot 10^{6}$
M06-L	$1.6 \cdot 10^{3}$	$1.6 \cdot 10^{4}$	$1.4 \cdot 10^{5}$	$1.6 \cdot 10^{6}$	$4.9 \cdot 10^{7}$	$3.3 \cdot 10^{2}$	$3.9 \cdot 10^{3}$	$3.6 \cdot 10^{4}$	$5.1 \cdot 10^{5}$	$9.5 \cdot 10^{6}$
M06-HF	$1.2 \cdot 10^{3}$	$1.4 \cdot 10^{4}$	$1.3 \cdot 10^{5}$	$1.8 \cdot 10^{6}$	$4.7 \cdot 10^{7}$	$5.6 \cdot 10^{2}$	$2.9 \cdot 10^{3}$	$5.1 \cdot 10^{4}$	$7.9 \cdot 10^{5}$	$2.3 \cdot 10^{7}$
M06-2X	$4.1 \cdot 10^{2}$	$4.1 \cdot 10^{3}$	$3.8 \cdot 10^{4}$	$6.6 \cdot 10^{5}$	$1.9 \cdot 10^{7}$	$3.4 \cdot 10^{2}$	$1.6 \cdot 10^{3}$	$2.3 \cdot 10^{4}$	$3.3 \cdot 10^{5}$	$9.1 \cdot 10^{6}$
M11	$6.9 \cdot 10^{2}$	$6.0 \cdot 10^{3}$	$4.8 \cdot 10^{4}$	$5.0 \cdot 10^{5}$	$4.6 \cdot 10^{6}$	$1.4 \cdot 10^{2}$	$1.3 \cdot 10^{3}$	$1.7 \cdot 10^{4}$	$3.3 \cdot 10^{5}$	$8.1 \cdot 10^{6}$
M11-L	$1.2 \cdot 10^{3}$	$1.3 \cdot 10^{4}$	$1.2 \cdot 10^{5}$	$1.3 \cdot 10^{6}$	$1.4 \cdot 10^{7}$	$1.2 \cdot 10^{3}$	$1.1 \cdot 10^{4}$	$1.1 \cdot 10^{5}$	$1.2 \cdot 10^{6}$	$1.8 \cdot 10^{7}$
SOGGA11	$8.8 \cdot 10^{2}$	$7.0 \cdot 10^{3}$	$6.8 \cdot 10^{4}$	$5.5 \cdot 10^{5}$	$1.1 \cdot 10^{7}$	$1.2 \cdot 10^{3}$	$5.8 \cdot 10^{3}$	$4.6 \cdot 10^{4}$	$4.9 \cdot 10^{5}$	$5.8 \cdot 10^{6}$
SOGGA11-X	$6.2 \cdot 10^{2}$	$4.1 \cdot 10^{3}$	$3.9 \cdot 10^{4}$	$2.9 \cdot 10^{5}$	$4.1 \cdot 10^{6}$	$1.6 \cdot 10^{2}$	$8.5 \cdot 10^{2}$	$5.0 \cdot 10^{3}$	$5.3 \cdot 10^{4}$	$3.0 \cdot 10^{5}$
N12	$2.8 \cdot 10^{2}$	$3.2 \cdot 10^{3}$	$5.6 \cdot 10^{4}$	$2.2 \cdot 10^{6}$	$1.2 \cdot 10^{8}$	$3.5 \cdot 10^{2}$	$3.6 \cdot 10^{3}$	$3.6 \cdot 10^{4}$	$3.6 \cdot 10^{5}$	$3.9 \cdot 10^{6}$
N12-SX	$4.7 \cdot 10^{2}$	$4.5 \cdot 10^{3}$	$4.9 \cdot 10^{4}$	$6.4 \cdot 10^{5}$	$1.9 \cdot 10^{7}$	$9.7 \cdot 10^{1}$	$9.6 \cdot 10^{2}$	$8.2 \cdot 10^{3}$	$9.6 \cdot 10^{4}$	$1.1 \cdot 10^{6}$
MN12-L	$1.4 \cdot 10^{3}$	$1.5 \cdot 10^{4}$	$1.4 \cdot 10^{5}$	$1.5 \cdot 10^{6}$	$1.5 \cdot 10^{7}$	$7.9 \cdot 10^{2}$	$8.2 \cdot 10^{3}$	$7.7 \cdot 10^{4}$	$8.3 \cdot 10^{5}$	$9.4 \cdot 10^{6}$
MN12-SX	$1.2 \cdot 10^{3}$	$1.3 \cdot 10^{4}$	$1.2 \cdot 10^{5}$	$1.3 \cdot 10^{6}$	$1.3 \cdot 10^{7}$	$2.0 \cdot 10^{3}$	$2.1 \cdot 10^{4}$	$2.0 \cdot 10^{5}$	$2.1 \cdot 10^{6}$	$2.1 \cdot 10^{7}$
MN15	$7.3 \cdot 10^{2}$	$8.1 \cdot 10^{3}$	$7.4 \cdot 10^{4}$	$8.3 \cdot 10^{5}$	$8.2 \cdot 10^{6}$	$3.5 \cdot 10^{2}$	$3.6 \cdot 10^{3}$	$3.4 \cdot 10^{4}$	$4.1 \cdot 10^{5}$	$3.9 \cdot 10^{6}$
MN15-L	$1.3 \cdot 10^{3}$	$1.5 \cdot 10^{4}$	$1.3 \cdot 10^{5}$	$1.5 \cdot 10^{6}$	$1.4 \cdot 10^{7}$	$1.9 \cdot 10^{2}$	$2.0 \cdot 10^{3}$	$1.5 \cdot 10^{4}$	$2.1 \cdot 10^{5}$	$2.7 \cdot 10^{6}$

Table S20: RMSE values for various energy derivatives obtained with the different functionals used in this work combined with the (250, 974) integration grid for the Ar_2 system.

**DFA**	$\mathrm{d}^n E_c/\mathrm{d}\xi^n$ [$\times 10^{-8}$]					$\mathrm{d}^n E_x/\mathrm{d}\xi^n$ [$\times 10^{-8}$]				
	0	1	2	3	4	0	1	2	3	4
SVWN5	$8.7 \cdot 10^{-3}$	$3.2 \cdot 10^{-1}$	$4.6 \cdot 10^{0}$	$1.1 \cdot 10^{2}$	$3.0 \cdot 10^{3}$	$2.1 \cdot 10^{-1}$	$9.3 \cdot 10^{0}$	$1.2 \cdot 10^{2}$	$3.0 \cdot 10^{3}$	$7.3 \cdot 10^{4}$
SPW92	$8.2 \cdot 10^{-3}$	$3.1 \cdot 10^{-1}$	$4.4 \cdot 10^{0}$	$1.0 \cdot 10^{2}$	$2.6 \cdot 10^{3}$	$2.1 \cdot 10^{-1}$	$9.3 \cdot 10^{0}$	$1.2 \cdot 10^{2}$	$3.0 \cdot 10^{3}$	$7.3 \cdot 10^{4}$
BHH	$5.2 \cdot 10^{-2}$	$1.3 \cdot 10^{0}$	$3.5 \cdot 10^{1}$	$9.0 \cdot 10^{2}$	$2.5 \cdot 10^{4}$	$1.1 \cdot 10^{-1}$	$4.8 \cdot 10^{0}$	$6.2 \cdot 10^{1}$	$1.5 \cdot 10^{3}$	$3.6 \cdot 10^{4}$
BLYP	$5.2 \cdot 10^{-2}$	$1.3 \cdot 10^{0}$	$3.4 \cdot 10^{1}$	$8.8 \cdot 10^{2}$	$2.4 \cdot 10^{4}$	$2.7 \cdot 10^{-1}$	$1.2 \cdot 10^{1}$	$1.5 \cdot 10^{2}$	$3.8 \cdot 10^{3}$	$9.1 \cdot 10^{4}$
B1LYP	$5.2 \cdot 10^{-2}$	$1.3 \cdot 10^{0}$	$3.5 \cdot 10^{1}$	$9.0 \cdot 10^{2}$	$2.4 \cdot 10^{4}$	$2.3 \cdot 10^{-1}$	$9.6 \cdot 10^{0}$	$1.3 \cdot 10^{2}$	$3.2 \cdot 10^{3}$	$7.5 \cdot 10^{4}$
B3LYP	$4.4 \cdot 10^{-2}$	$1.1 \cdot 10^{0}$	$2.9 \cdot 10^{1}$	$7.4 \cdot 10^{2}$	$2.0 \cdot 10^{4}$	$2.3 \cdot 10^{-1}$	$9.9 \cdot 10^{0}$	$1.3 \cdot 10^{2}$	$3.2 \cdot 10^{3}$	$7.5 \cdot 10^{4}$
BHHLYP	$5.2 \cdot 10^{-2}$	$1.3 \cdot 10^{0}$	$3.5 \cdot 10^{1}$	$9.0 \cdot 10^{2}$	$2.5 \cdot 10^{4}$	$1.7 \cdot 10^{-1}$	$6.9 \cdot 10^{0}$	$9.8 \cdot 10^{1}$	$2.4 \cdot 10^{3}$	$5.8 \cdot 10^{4}$
LC-BLYP	$5.1 \cdot 10^{-2}$	$1.3 \cdot 10^{0}$	$3.5 \cdot 10^{1}$	$8.9 \cdot 10^{2}$	$2.4 \cdot 10^{4}$	$1.6 \cdot 10^{-1}$	$8.7 \cdot 10^{0}$	$9.9 \cdot 10^{1}$	$2.6 \cdot 10^{3}$	$6.3 \cdot 10^{4}$
CAMB3LYP	$4.4 \cdot 10^{-2}$	$1.1 \cdot 10^{0}$	$2.9 \cdot 10^{1}$	$7.5 \cdot 10^{2}$	$2.0 \cdot 10^{4}$	$1.5 \cdot 10^{-1}$	$8.0 \cdot 10^{0}$	$8.2 \cdot 10^{1}$	$2.0 \cdot 10^{3}$	$4.6 \cdot 10^{4}$
PBE	$2.8 \cdot 10^{-1}$	$8.4 \cdot 10^{0}$	$2.1 \cdot 10^{2}$	$6.2 \cdot 10^{3}$	$1.6 \cdot 10^{5}$	$2.8 \cdot 10^{-1}$	$1.2 \cdot 10^{1}$	$2.1 \cdot 10^{2}$	$5.4 \cdot 10^{3}$	$1.6 \cdot 10^{5}$
PBE0	$3.0 \cdot 10^{-1}$	$8.9 \cdot 10^{0}$	$2.3 \cdot 10^{2}$	$6.4 \cdot 10^{3}$	$1.7 \cdot 10^{5}$	$2.2 \cdot 10^{-1}$	$9.1 \cdot 10^{0}$	$1.6 \cdot 10^{2}$	$4.1 \cdot 10^{3}$	$1.2 \cdot 10^{5}$
PBE50	$3.2 \cdot 10^{-1}$	$9.5 \cdot 10^{0}$	$2.4 \cdot 10^{2}$	$6.8 \cdot 10^{3}$	$1.7 \cdot 10^{5}$	$1.5 \cdot 10^{-1}$	$6.2 \cdot 10^{0}$	$1.1 \cdot 10^{2}$	$2.8 \cdot 10^{3}$	$8.2 \cdot 10^{4}$
LC-wPBE	$3.0 \cdot 10^{-1}$	$8.7 \cdot 10^{0}$	$2.2 \cdot 10^{2}$	$6.3 \cdot 10^{3}$	$1.6 \cdot 10^{5}$	$2.5 \cdot 10^{-1}$	$6.3 \cdot 10^{0}$	$1.9 \cdot 10^{2}$	$5.0 \cdot 10^{3}$	$1.6 \cdot 10^{5}$
TPSS	$3.0 \cdot 10^{-1}$	$8.7 \cdot 10^{0}$	$2.2 \cdot 10^{2}$	$6.3 \cdot 10^{3}$	$1.6 \cdot 10^{5}$	$1.8 \cdot 10^{0}$	$4.9 \cdot 10^{1}$	$1.2 \cdot 10^{3}$	$3.5 \cdot 10^{4}$	$9.1 \cdot 10^{5}$
RevTPSS	$2.7 \cdot 10^{-1}$	$7.8 \cdot 10^{0}$	$2.0 \cdot 10^{2}$	$5.7 \cdot 10^{3}$	$1.5 \cdot 10^{5}$	$1.5 \cdot 10^{0}$	$4.1 \cdot 10^{1}$	$1.0 \cdot 10^{3}$	$2.8 \cdot 10^{4}$	$7.3 \cdot 10^{5}$
TPSSh	$3.0 \cdot 10^{-1}$	$8.8 \cdot 10^{0}$	$2.3 \cdot 10^{2}$	$6.4 \cdot 10^{3}$	$1.6 \cdot 10^{5}$	$1.6 \cdot 10^{0}$	$4.5 \cdot 10^{1}$	$1.2 \cdot 10^{3}$	$3.2 \cdot 10^{4}$	$8.4 \cdot 10^{5}$
B97	$2.2 \cdot 10^{1}$	$6.0 \cdot 10^{2}$	$1.7 \cdot 10^{4}$	$5.7 \cdot 10^{5}$	$2.4 \cdot 10^{7}$	$1.5 \cdot 10^{-1}$	$8.0 \cdot 10^{0}$	$1.2 \cdot 10^{2}$	$3.2 \cdot 10^{3}$	$9.4 \cdot 10^{4}$
B97-D	$1.3 \cdot 10^{1}$	$3.7 \cdot 10^{2}$	$1.0 \cdot 10^{4}$	$3.8 \cdot 10^{5}$	$1.7 \cdot 10^{7}$	$7.0 \cdot 10^{-1}$	$2.1 \cdot 10^{1}$	$5.1 \cdot 10^{2}$	$1.5 \cdot 10^{4}$	$3.9 \cdot 10^{5}$
wB97	$3.6 \cdot 10^{1}$	$8.9 \cdot 10^{2}$	$2.2 \cdot 10^{4}$	$6.9 \cdot 10^{5}$	$3.1 \cdot 10^{7}$	$3.3 \cdot 10^{-1}$	$1.1 \cdot 10^{1}$	$2.2 \cdot 10^{2}$	$5.7 \cdot 10^{3}$	$1.8 \cdot 10^{5}$
wB97X	$1.8 \cdot 10^{1}$	$4.4 \cdot 10^{2}$	$1.2 \cdot 10^{4}$	$5.4 \cdot 10^{5}$	$3.9 \cdot 10^{7}$	$1.6 \cdot 10^{-1}$	$7.6 \cdot 10^{0}$	$1.2 \cdot 10^{2}$	$3.4 \cdot 10^{3}$	$1.1 \cdot 10^{5}$
wB97X-D	$2.8 \cdot 10^{1}$	$7.0 \cdot 10^{2}$	$2.0 \cdot 10^{4}$	$9.7 \cdot 10^{5}$	$7.0 \cdot 10^{7}$	$2.4 \cdot 10^{-1}$	$8.8 \cdot 10^{0}$	$1.8 \cdot 10^{2}$	$5.0 \cdot 10^{3}$	$1.5 \cdot 10^{5}$
wB97X-D3	$1.8 \cdot 10^{1}$	$4.5 \cdot 10^{2}$	$1.3 \cdot 10^{4}$	$6.3 \cdot 10^{5}$	$4.6 \cdot 10^{7}$	$1.6 \cdot 10^{-1}$	$7.5 \cdot 10^{0}$	$1.2 \cdot 10^{2}$	$3.3 \cdot 10^{3}$	$1.0 \cdot 10^{5}$
wB97M-V	$7.9 \cdot 10^{0}$	$2.0 \cdot 10^{2}$	$5.1 \cdot 10^{3}$	$1.7 \cdot 10^{5}$	$7.1 \cdot 10^{6}$	$1.9 \cdot 10^{0}$	$9.9 \cdot 10^{0}$	$3.4 \cdot 10^{3}$	$4.6 \cdot 10^{3}$	$6.1 \cdot 10^{4}$
wB97X-V	$4.2 \cdot 10^{0}$	$1.1 \cdot 10^{2}$	$2.9 \cdot 10^{3}$	$7.7 \cdot 10^{4}$	$2.3 \cdot 10^{6}$	$1.7 \cdot 10^{0}$	$2.7 \cdot 10^{3}$	$3.0 \cdot 10^{3}$	$4.5 \cdot 10^{3}$	$9.4 \cdot 10^{4}$
B97M-V	$9.9 \cdot 10^{-1}$	$2.6 \cdot 10^{1}$	$7.0 \cdot 10^{2}$	$2.3 \cdot 10^{4}$	$8.3 \cdot 10^{5}$	$2.3 \cdot 10^{0}$	$3.0 \cdot 10^{1}$	$4.0 \cdot 10^{3}$	$2.1 \cdot 10^{4}$	$5.6 \cdot 10^{5}$
B1PW91	$2.5 \cdot 10^{-1}$	$7.3 \cdot 10^{0}$	$1.8 \cdot 10^{2}$	$5.1 \cdot 10^{3}$	$1.3 \cdot 10^{5}$	$2.7 \cdot 10^{-1}$	$1.0 \cdot 10^{1}$	$1.5 \cdot 10^{2}$	$3.8 \cdot 10^{3}$	$9.2 \cdot 10^{4}$
PW91	$2.3 \cdot 10^{-1}$	$6.8 \cdot 10^{0}$	$1.7 \cdot 10^{2}$	$4.9 \cdot 10^{3}$	$1.3 \cdot 10^{5}$	$3.3 \cdot 10^{0}$	$2.9 \cdot 10^{3}$	$3.3 \cdot 10^{3}$	$1.1 \cdot 10^{4}$	$5.5 \cdot 10^{5}$
mPW91	$2.3 \cdot 10^{-1}$	$6.9 \cdot 10^{0}$	$1.7 \cdot 10^{2}$	$4.9 \cdot 10^{3}$	$1.3 \cdot 10^{5}$	$3.9 \cdot 10^{-1}$	$1.4 \cdot 10^{1}$	$2.9 \cdot 10^{2}$	$1.1 \cdot 10^{4}$	$5.7 \cdot 10^{5}$
VSXC	$5.2 \cdot 10^{0}$	$1.3 \cdot 10^{2}$	$3.8 \cdot 10^{3}$	$9.8 \cdot 10^{4}$	$2.9 \cdot 10^{6}$	$2.3 \cdot 10^{0}$	$6.4 \cdot 10^{1}$	$1.7 \cdot 10^{3}$	$4.7 \cdot 10^{4}$	$1.3 \cdot 10^{6}$
SCAN	$3.8 \cdot 10^{0}$	$1.0 \cdot 10^{2}$	$4.2 \cdot 10^{3}$	$2.5 \cdot 10^{5}$	$2.1 \cdot 10^{7}$	$1.7 \cdot 10^{1}$	$5.2 \cdot 10^{2}$	$1.9 \cdot 10^{4}$	$8.5 \cdot 10^{5}$	$5.5 \cdot 10^{7}$
SCAN0	$3.8 \cdot 10^{0}$	$1.0 \cdot 10^{2}$	$4.2 \cdot 10^{3}$	$2.5 \cdot 10^{5}$	$2.1 \cdot 10^{7}$	$1.2 \cdot 10^{1}$	$3.8 \cdot 10^{2}$	$1.4 \cdot 10^{4}$	$6.3 \cdot 10^{5}$	$4.1 \cdot 10^{7}$
M06	$4.2 \cdot 10^{1}$	$1.1 \cdot 10^{3}$	$2.7 \cdot 10^{4}$	$7.3 \cdot 10^{5}$	$2.3 \cdot 10^{7}$	$7.4 \cdot 10^{0}$	$2.1 \cdot 10^{2}$	$5.6 \cdot 10^{3}$	$1.6 \cdot 10^{5}$	$4.4 \cdot 10^{6}$
M06-L	$4.6 \cdot 10^{1}$	$1.1 \cdot 10^{3}$	$2.8 \cdot 10^{4}$	$8.5 \cdot 10^{5}$	$3.4 \cdot 10^{7}$	$6.7 \cdot 10^{0}$	$1.8 \cdot 10^{2}$	$5.4 \cdot 10^{3}$	$1.5 \cdot 10^{5}$	$5.3 \cdot 10^{6}$
M06-HF	$3.0 \cdot 10^{1}$	$7.9 \cdot 10^{2}$	$2.1 \cdot 10^{4}$	$6.5 \cdot 10^{5}$	$2.4 \cdot 10^{7}$	$2.2 \cdot 10^{1}$	$6.4 \cdot 10^{2}$	$1.7 \cdot 10^{4}$	$5.0 \cdot 10^{5}$	$1.4 \cdot 10^{7}$
M06-2X	$9.1 \cdot 10^{0}$	$2.0 \cdot 10^{2}$	$6.8 \cdot 10^{3}$	$2.7 \cdot 10^{5}$	$1.3 \cdot 10^{7}$	$9.3 \cdot 10^{0}$	$2.7 \cdot 10^{2}$	$7.1 \cdot 10^{3}$	$2.1 \cdot 10^{5}$	$6.1 \cdot 10^{6}$
M11	$3.8 \cdot 10^{0}$	$9.5 \cdot 10^{1}$	$2.8 \cdot 10^{3}$	$7.1 \cdot 10^{4}$	$2.2 \cdot 10^{6}$	$1.0 \cdot 10^{1}$	$2.8 \cdot 10^{2}$	$7.9 \cdot 10^{3}$	$2.1 \cdot 10^{5}$	$6.2 \cdot 10^{6}$
M11-L	$1.1 \cdot 10^{1}$	$2.9 \cdot 10^{2}$	$7.8 \cdot 10^{3}$	$2.2 \cdot 10^{5}$	$5.8 \cdot 10^{6}$	$2.0 \cdot 10^{1}$	$5.5 \cdot 10^{2}$	$1.5 \cdot 10^{4}$	$4.2 \cdot 10^{5}$	$1.2 \cdot 10^{7}$
SOGGA11	$2.1 \cdot 10^{1}$	$5.2 \cdot 10^{2}$	$1.4 \cdot 10^{4}$	$3.6 \cdot 10^{5}$	$1.1 \cdot 10^{7}$	$1.4 \cdot 10^{0}$	$3.8 \cdot 10^{1}$	$1.0 \cdot 10^{3}$	$2.9 \cdot 10^{4}$	$8.7 \cdot 10^{5}$
SOGGA11-X	$4.4 \cdot 10^{0}$	$1.1 \cdot 10^{2}$	$2.9 \cdot 10^{3}$	$8.8 \cdot 10^{4}$	$3.3 \cdot 10^{6}$	$4.9 \cdot 10^{-1}$	$1.1 \cdot 10^{1}$	$3.8 \cdot 10^{2}$	$5.4 \cdot 10^{3}$	$1.2 \cdot 10^{5}$
N12	$3.0 \cdot 10^{1}$	$7.8 \cdot 10^{2}$	$2.1 \cdot 10^{4}$	$9.6 \cdot 10^{5}$	$6.3 \cdot 10^{7}$	$7.4 \cdot 10^{-1}$	$2.2 \cdot 10^{1}$	$5.5 \cdot 10^{2}$	$1.6 \cdot 10^{4}$	$4.6 \cdot 10^{5}$
N12-SX	$1.6 \cdot 10^{1}$	$3.9 \cdot 10^{2}$	$1.1 \cdot 10^{4}$	$3.5 \cdot 10^{5}$	$1.7 \cdot 10^{7}$	$9.2 \cdot 10^{-1}$	$2.8 \cdot 10^{1}$	$6.9 \cdot 10^{2}$	$2.0 \cdot 10^{4}$	$5.5 \cdot 10^{5}$
MN12-L	$4.6 \cdot 10^{0}$	$1.3 \cdot 10^{2}$	$3.3 \cdot 10^{3}$	$9.4 \cdot 10^{4}$	$2.5 \cdot 10^{6}$	$8.3 \cdot 10^{0}$	$2.2 \cdot 10^{2}$	$6.3 \cdot 10^{3}$	$1.7 \cdot 10^{5}$	$4.8 \cdot 10^{6}$
MN12-SX	$6.1 \cdot 10^{0}$	$1.5 \cdot 10^{2}$	$5.1 \cdot 10^{3}$	$1.1 \cdot 10^{5}$	$3.1 \cdot 10^{6}$	$7.5 \cdot 10^{0}$	$2.0 \cdot 10^{2}$	$5.6 \cdot 10^{3}$	$1.5 \cdot 10^{5}$	$4.4 \cdot 10^{6}$
MN15	$3.5 \cdot 10^{0}$	$1.0 \cdot 10^{2}$	$2.6 \cdot 10^{3}$	$7.6 \cdot 10^{4}$	$2.0 \cdot 10^{6}$	$7.9 \cdot 10^{-1}$	$2.4 \cdot 10^{1}$	$5.8 \cdot 10^{2}$	$1.5 \cdot 10^{4}$	$4.4 \cdot 10^{5}$
MN15-L	$4.7 \cdot 10^{0}$	$1.4 \cdot 10^{2}$	$3.5 \cdot 10^{3}$	$9.9 \cdot 10^{4}$	$2.6 \cdot 10^{6}$	$3.3 \cdot 10^{0}$	$9.5 \cdot 10^{1}$	$2.5 \cdot 10^{3}$	$6.9 \cdot 10^{4}$	$1.8 \cdot 10^{6}$

Table S21: *Part 1)* Ratios between the RMSE values of E_x and E_c for some DFAs combined with the (99, 590) grid. The numbers correspond to the averages of ratios for the $\mathrm{d}^3E/\mathrm{d}\xi^3$ and $\mathrm{d}^4E/\mathrm{d}\xi^4$ derivatives, computed separately for each chemical system. The second and third columns show the names of the (meta-)GGAs exchange and correlation functionals included in a given DFA (information not shown for the standalone functionals, for which the exchange and correlation parts are not meant to be used separately).

DFA	E_x^{DFA}	E_c^{DFA}	HCN · HF	HCN · HCl	OC · HF	N_2 · HF	HCN · BrF	Ar_2
SVWN5	S	VWN5	8.1	7.0	8.4	9.6	19.3	11.7
SPW92	S	PW92	8.3	5.7	7.9	9.3	19.4	11.8
BH&H	S	LYP	1.4	2.0	0.9	2.2	7.8	7.1
BLYP	B88	LYP	14.0	27.5	6.9	19.2	11.8	20.9
B1LYP	B88	LYP	11.1	21.8	6.1	8.2	8.1	16.1
B3LYP	B88	LYP	14.7	21.3	8.0	8.6	9.5	15.1
BH&HLYP	B88	LYP	7.7	16.4	5.0	6.5	5.8	11.0
LC-BLYP	B88	LYP	6.6	8.3	3.2	7.2	10.4	10.8
CAM-B3LYP	B88	LYP	12.1	18.8	6.7	7.0	9.1	12.0
PBE	PBE	PBE	2.2	3.3	1.6	3.6	8.9	1.1
PBE0	PBE	PBE	1.6	2.0	1.3	1.7	9.1	0.8
PBE50	PBE	PBE	1.1	1.2	0.8	0.8	8.7	0.5
LC-ωPBE	PBE	PBE	2.1	1.3	1.7	1.1	14.3	0.6
TPSS	TPSS	TPSS	2.7	9.1	2.2	5.5	19.1	0.8
RevTPSS	RevTPSS	RevTPSS	3.8	9.5	3.8	5.9	16.7	1.0
TPSSh	TPSS	TPSS	2.7	8.0	2.2	4.4	16.9	0.8
B97			$1.4{\cdot}10^{-2}$	$2.1{\cdot}10^{-3}$	$2.1{\cdot}10^{-2}$	$4.6{\cdot}10^{-3}$	$2.3{\cdot}10^{-1}$	$4.7{\cdot}10^{-2}$
B97-D			$3.3{\cdot}10^{-2}$	$4.8{\cdot}10^{-3}$	$4.7{\cdot}10^{-2}$	$3.2{\cdot}10^{-2}$	$3.8{\cdot}10^{-1}$	$1.8{\cdot}10^{-1}$
ωB97			$1.5{\cdot}10^{-2}$	$3.2{\cdot}10^{-3}$	$1.2{\cdot}10^{-2}$	$4.7{\cdot}10^{-3}$	$9.9{\cdot}10^{-2}$	$3.3{\cdot}10^{-2}$
ωB97X			$1.4{\cdot}10^{-2}$	$2.4{\cdot}10^{-3}$	$1.1{\cdot}10^{-2}$	$4.5{\cdot}10^{-3}$	$5.0{\cdot}10^{-2}$	$3.5{\cdot}10^{-2}$
ωB97X-D			$6.7{\cdot}10^{-3}$	$1.2{\cdot}10^{-3}$	$5.8{\cdot}10^{-3}$	$1.9{\cdot}10^{-3}$	$2.8{\cdot}10^{-2}$	$2.4{\cdot}10^{-2}$
ωB97X-D3			$1.0{\cdot}10^{-2}$	$1.8{\cdot}10^{-3}$	$8.7{\cdot}10^{-3}$	$3.1{\cdot}10^{-3}$	$4.2{\cdot}10^{-2}$	$3.1{\cdot}10^{-2}$
ωB97M-V			$2.3{\cdot}10^{-1}$	$3.0{\cdot}10^{-2}$	$1.0{\cdot}10^{-1}$	$6.2{\cdot}10^{-2}$	$7.0{\cdot}10^{-1}$	$2.2{\cdot}10^{-1}$
ωB97X-V			$2.3{\cdot}10^{-1}$	$2.9{\cdot}10^{-2}$	$4.2{\cdot}10^{-1}$	$5.2{\cdot}10^{-2}$	5.9	$2.8{\cdot}10^{-1}$
B97M-V			$2.2{\cdot}10^{-1}$	$1.6{\cdot}10^{-1}$	$3.6{\cdot}10^{-1}$	$4.0{\cdot}10^{-1}$	2.7	2.0

Table S22: *Part 2)* Ratios between the RMSE values of E_x and E_c for some DFAs combined with the (99, 590) grid. The numbers correspond to the averages of ratios for the $\mathrm{d}^3E/\mathrm{d}\xi^3$ and $\mathrm{d}^4E/\mathrm{d}\xi^4$ derivatives, computed separately for each chemical system. The second and third columns show the names of the (meta-)GGAs exchange and correlation functionals included in a given DFA (information not shown for the standalone functionals, for which the exchange and correlation parts are not meant to be used separately).

DFA	E_x^{DFA}	E_c^{DFA}	$HCN \cdot HF$	$HCN \cdot HCl$	$OC \cdot HF$	$N_2 \cdot HF$	$HCN \cdot BrF$	Ar_2
B1PW91	B88	PW91	1.5	1.2	0.7	0.4	6.9	0.9
PW91	PW91	PW91	4.1	5.7	3.1	6.4	13.8	1.9
mPW91	mPW91	mPW91	4.5	6.4	3.0	4.9	16.2	1.9
VSXC			0.4	0.1	0.3	$4.2{\cdot}10^{-2}$	0.8	0.1
SCAN			2.6	4.2	3.0	3.3	2.5	2.9
SCAN0			2.0	3.4	2.2	2.5	3.5	2.2
M06			0.5	0.1	0.7	0.6	0.6	0.1
M06-L			0.3	0.1	0.6	0.3	1.5	0.3
M06-HF			1.1	0.2	1.5	0.4	0.5	0.5
M06-2X			0.6	0.2	1.2	0.5	0.5	0.5
M11			3.5	2.2	4.0	2.7	2.4	1.2
M11-L			3.6	3.5	2.8	2.2	1.6	1.1
SOGGA11			$9.4{\cdot}10^{-2}$	$1.2{\cdot}10^{-1}$	$1.5{\cdot}10^{-1}$	$4.3{\cdot}10^{-2}$	$3.9{\cdot}10^{-1}$	$7.0{\cdot}10^{-1}$
SOGGA11-X			$1.1{\cdot}10^{-1}$	$1.1{\cdot}10^{-1}$	$7.4{\cdot}10^{-2}$	$5.2{\cdot}10^{-2}$	1.8	$1.3{\cdot}10^{-1}$
N12			$1.4{\cdot}10^{-2}$	$3.1{\cdot}10^{-3}$	$1.6{\cdot}10^{-2}$	$6.6{\cdot}10^{-3}$	$1.4{\cdot}10^{-1}$	$9.8{\cdot}10^{-2}$
N12-SX			$5.8{\cdot}10^{-2}$	$1.9{\cdot}10^{-2}$	$5.8{\cdot}10^{-2}$	$7.0{\cdot}10^{-2}$	1.2	$1.0{\cdot}10^{-1}$
MN12-L			1.9	1.4	2.9	1.0	6.2	0.6
MN12-SX			1.5	0.9	1.5	0.7	5.0	1.7
MN15			0.6	0.7	0.5	0.7	4.9	0.5
MN15-L			0.9	1.1	0.6	0.3	5.5	0.2

Table S23: *Part 1)* Ratios between the RMSE values of E_x and E_c for some DFAs combined with the (250, 974) grid. The numbers correspond to the averages of ratios for the $\mathrm{d}^3E/\mathrm{d}\xi^3$ and $\mathrm{d}^4E/\mathrm{d}\xi^4$ derivatives, computed separately for each chemical system. The second and third columns show the names of the (meta-)GGAs exchange and correlation functionals included in a given DFA (information not shown for standalone functionals, for which exchange and correlation parts are not meant to be used separately).

DFA	E_x^{DFA}	E_c^{DFA}	HCN · HF	HCN · HCl	OC · HF	N_2 · HF	HCN · BrF	Ar_2
SVWN5	S	VWN5	0.5	1.7	1.4	1.9	35.0	25.5
SPW92	S	PW92	2.1	1.3	1.5	0.8	36.0	28.5
BH&H	S	LYP	1.2	0.9	1.1	1.1	37.3	1.6
BLYP	B88	LYP	4.8	4.2	2.0	9.6	101.1	4.0
B1LYP	B88	LYP	2.4	2.5	4.8	8.9	58.4	3.3
B3LYP	B88	LYP	1.4	4.1	3.3	4.3	61.8	4.1
BH&HLYP	B88	LYP	3.6	2.5	2.1	5.0	67.0	2.5
LC-BLYP	B88	LYP	1.6	0.9	0.7	0.8	208.1	2.7
CAM-B3LYP	B88	LYP	0.9	2.2	1.2	3.9	62.7	2.5
PBE	PBE	PBE	1.1	0.6	1.4	0.8	11.2	0.9
PBE0	PBE	PBE	1.5	0.9	2.0	0.5	8.9	0.7
PBE50	PBE	PBE	0.8	0.6	0.7	0.6	5.2	0.4
LC-ωPBE	PBE	PBE	0.9	1.7	7.9	1.5	16.1	0.9
TPSS	TPSS	TPSS	103.7	20.4	30.6	73.3	11.8	5.5
RevTPSS	RevTPSS	RevTPSS	2.7	3.3	2.7	61.8	13.0	4.9
TPSSh	TPSS	TPSS	74.5	13.6	3.3	36.5	8.2	5.0
B97			$3.0{\cdot}10^{-4}$	$5.3{\cdot}10^{-4}$	$7.3{\cdot}10^{-4}$	$4.1{\cdot}10^{-4}$	$8.8{\cdot}10^{-3}$	$4.7{\cdot}10^{-3}$
B97-D			$1.2{\cdot}10^{-3}$	$1.7{\cdot}10^{-2}$	$2.6{\cdot}10^{-2}$	$1.3{\cdot}10^{-2}$	$2.8{\cdot}10^{-2}$	$3.1{\cdot}10^{-2}$
ωB97			$4.8{\cdot}10^{-4}$	$5.2{\cdot}10^{-4}$	$8.5{\cdot}10^{-3}$	$2.8{\cdot}10^{-3}$	$4.5{\cdot}10^{-3}$	$7.1{\cdot}10^{-3}$
ωB97X			$1.5{\cdot}10^{-4}$	$1.9{\cdot}10^{-4}$	$3.7{\cdot}10^{-4}$	$9.3{\cdot}10^{-5}$	$2.1{\cdot}10^{-3}$	$4.5{\cdot}10^{-3}$
ωB97X-D			$8.2{\cdot}10^{-5}$	$1.1{\cdot}10^{-4}$	$2.6{\cdot}10^{-4}$	$6.5{\cdot}10^{-5}$	$8.4{\cdot}10^{-4}$	$3.6{\cdot}10^{-3}$
ωB97X-D3			$9.6{\cdot}10^{-5}$	$1.5{\cdot}10^{-4}$	$3.0{\cdot}10^{-4}$	$5.2{\cdot}10^{-5}$	$1.4{\cdot}10^{-3}$	$3.7{\cdot}10^{-3}$
ωB97M-V			$2.9{\cdot}10^{-3}$	$3.9{\cdot}10^{-3}$	$7.2{\cdot}10^{-3}$	$7.5{\cdot}10^{-3}$	$4.2{\cdot}10^{-2}$	$1.8{\cdot}10^{-2}$
ωB97X-V			$1.0{\cdot}10^{-2}$	$2.1{\cdot}10^{-2}$	$1.2{\cdot}10^{-2}$	$9.0{\cdot}10^{-3}$	$2.3{\cdot}10^{-1}$	$5.0{\cdot}10^{-2}$
B97M-V			$5.7{\cdot}10^{-3}$	$1.3{\cdot}10^{-1}$	$1.4{\cdot}10^{-1}$	$3.1{\cdot}10^{-1}$	$3.5{\cdot}10^{-2}$	$7.9{\cdot}10^{-1}$

Table S24: *Part 2)* Ratios between the RMSE values of E_x and E_c for some DFAs combined with the (250, 974) grid. The numbers correspond to the averages of ratios for the $d^3E/d\xi^3$ and $d^4E/d\xi^4$ derivatives, computed separately for each chemical system. The second and third columns show the names of the (meta-)GGAs exchange and correlation functionals included in a given DFA (information not shown for standalone functionals, for which exchange and correlation parts are not meant to be used separately).

DFA	E_x^{DFA}	E_c^{DFA}	HCN · HF	HCN · HCl	OC · HF	N_2 · HF	HCN · BrF	Ar_2
B1PW91	B88	PW91	$4.8{\cdot}10^{-1}$	$1.2{\cdot}10^{-2}$	$2.0{\cdot}10^{-2}$	$4.3{\cdot}10^{-2}$	$4.0{\cdot}10^{-1}$	$7.2{\cdot}10^{-1}$
PW91	PW91	PW91	116.2	5.7	118.5	6.0	5.9	3.3
mPW91	mPW91	mPW91	151.1	6.2	129.1	6.3	5.6	3.3
VSXC			0.4	0.5	0.4	0.5	1.9	0.5
SCAN			3.5	4.4	4.5	2.6	6.5	3.0
SCAN0			3.0	3.0	2.6	1.9	3.5	2.3
M06			0.7	0.5	1.1	0.4	2.1	0.2
M06-L			2.7	1.3	3.2	0.9	2.5	0.2
M06-HF			7.1	1.9	5.3	1.2	0.9	0.7
M06-2X			3.1	1.2	3.2	1.3	0.5	0.6
M11			3.5	4.9	3.1	5.1	1.2	2.9
M11-L			4.6	4.4	5.1	4.3	12.5	2.0
SOGGA11			$1.0{\cdot}10^{-2}$	$5.6{\cdot}10^{-2}$	$9.8{\cdot}10^{-2}$	$2.6{\cdot}10^{-2}$	$3.1{\cdot}10^{-1}$	$7.9{\cdot}10^{-2}$
SOGGA11-X			$8.5{\cdot}10^{-4}$	$7.6{\cdot}10^{-3}$	$3.3{\cdot}10^{-3}$	$3.0{\cdot}10^{-3}$	$1.3{\cdot}10^{-1}$	$4.8{\cdot}10^{-2}$
N12			$2.1{\cdot}10^{-2}$	$4.5{\cdot}10^{-3}$	$9.6{\cdot}10^{-3}$	$2.7{\cdot}10^{-3}$	$3.9{\cdot}10^{-3}$	$1.2{\cdot}10^{-2}$
N12-SX			$9.0{\cdot}10^{-2}$	$3.0{\cdot}10^{-2}$	$5.9{\cdot}10^{-2}$	$3.8{\cdot}10^{-2}$	$1.3{\cdot}10^{-1}$	$4.5{\cdot}10^{-2}$
MN12-L			2.4	4.6	2.3	5.1	6.7	1.9
MN12-SX			1.1	1.4	0.8	1.4	2.2	1.4
MN15			$7.3{\cdot}10^{-3}$	$8.0{\cdot}10^{-2}$	$1.7{\cdot}10^{-2}$	$4.9{\cdot}10^{-2}$	2.6	$2.1{\cdot}10^{-1}$
MN15-L			0.6	1.1	0.3	1.0	3.7	

Table S25: Functionals used in Figure 4.

DFA
B3LYP
BHH
BHHLYP
LC-BLYP
M06-2X
N12
MN15
MN15L
SCAN0
PBE
PBE0
B97
wB97X
B97M-V
wB97M-V
wB97X-V
TPSSh
SVWN5
mPW91
VSXC

Table S26: The first six columns display the average maximum RMSE error of each DFA family (refer to Table S25 for the family members), arranged from lowest to highest. The concluding three columns give the maximal RMSE percentage contribution to exchange and correlation components. This data was obtained from Table 1 in the manuscript and utilized to classify the families in Figure 6 of the manuscript (note that in Figure 6, we have grouped the families B97st, B97X, and B97M under the name B97). Furthermore, the font size in Figure 6 follows the rule $\log[(RMSE_{E_{xc}^{\text{func.}}}/RMSE_{E_{xc}^{\text{SCAN}}}) \times 1000] \times 150$, with constants in the formula chosen to maintain a reasonable font size range, and E_{xc} reaped from the data in this table.

Family	E_c	Family	E_x	Family	E_{xc}	Family	% E_x	% E_c
BH&H	6.92×10^{-4}	BH&H	4.59×10^{-3}	BH&H	5.29×10^{-3}	B97X	1.09	98.91
BLYP	7.03×10^{-4}	B97X	7.53×10^{-3}	LSDA	9.94×10^{-3}	B97st	2.89	97.11
LSDA	7.69×10^{-4}	PBE	8.40×10^{-3}	BLYP	1.00×10^{-2}	N12	3.37	96.63
TPSS	1.05×10^{-2}	LSDA	9.17×10^{-3}	PBE	1.95×10^{-2}	VSXC	4.72	95.28
PW91	1.05×10^{-2}	BLYP	9.32×10^{-3}	TPSS	2.13×10^{-2}	M06	21.72	78.28
PBE	1.11×10^{-2}	TPSS	1.08×10^{-2}	PW91	3.08×10^{-2}	B97M	22.46	77.54
B97M	5.73×10^{-2}	B97st	1.18×10^{-2}	B97M	7.39×10^{-2}	MN15	22.98	77.02
SOGGA11	7.76×10^{-2}	B97M	1.66×10^{-2}	SOGGA11	1.08×10^{-1}	SOGGA11	28.21	71.79
M11	9.11×10^{-2}	PW91	2.03×10^{-2}	MN15	1.44×10^{-1}	PBE	43.00	57.00
MN15	1.11×10^{-1}	VSXC	2.24×10^{-2}	M11	2.21×10^{-1}	TPSS	50.67	49.33
MN12	1.36×10^{-1}	N12	2.50×10^{-2}	MN12	2.89×10^{-1}	MN12	53.06	46.94
SCAN	3.61×10^{-1}	SOGGA11	3.05×10^{-2}	B97st	4.08×10^{-1}	M11	58.79	41.21
B97st	3.97×10^{-1}	MN15	3.30×10^{-2}	VSXC	4.75×10^{-1}	PW91	65.85	34.15
M06	4.40×10^{-1}	M06	1.22×10^{-1}	M06	5.62×10^{-1}	SCAN	71.58	28.42
VSXC	4.53×10^{-1}	M11	1.30×10^{-1}	B97X	6.93×10^{-1}	BH&H	86.91	13.09
B97X	6.86×10^{-1}	MN12	1.54×10^{-1}	N12	7.43×10^{-1}	LSDA	92.26	7.74
N12	7.18×10^{-1}	SCAN	9.09×10^{-1}	SCAN	1.27×10^{0}	BLYP	92.99	7.01

Table S27: RMSE values for various correlation energy derivatives obtained with different functionals, averaged across all systems and grids.

DFA	$\mathrm{d}^1E_c/\mathrm{d}\xi^1$	DFA	$\mathrm{d}^2E_c/\mathrm{d}\xi^2$	DFA	$\mathrm{d}^3E_c/\mathrm{d}\xi^3$	DFA	$\mathrm{d}^4E_c/\mathrm{d}\xi^4$
BH&H	$7.37 \cdot 10^{-8}$	B1LYP	$6.01 \cdot 10^{-7}$	BH&HLYP	$5.93 \cdot 10^{-6}$	SPW92	$6.75 \cdot 10^{-5}$
BH&HLYP	$7.48 \cdot 10^{-8}$	BLYP	$6.07 \cdot 10^{-7}$	LC-BLYP	$6.01 \cdot 10^{-6}$	SVWN5	$6.83 \cdot 10^{-5}$
LC-BLYP	$7.92 \cdot 10^{-8}$	LC-BLYP	$6.12 \cdot 10^{-7}$	B1LYP	$6.04 \cdot 10^{-6}$	CAMB3LYP	$7.72 \cdot 10^{-5}$
BLYP	$8.00 \cdot 10^{-8}$	BH&HLYP	$6.13 \cdot 10^{-7}$	BLYP	$6.06 \cdot 10^{-6}$	B3LYP	$8.01 \cdot 10^{-5}$
B3LYP	$8.10 \cdot 10^{-8}$	BH&H	$6.25 \cdot 10^{-7}$	CAMB3LYP	$6.08 \cdot 10^{-6}$	B1LYP	$8.06 \cdot 10^{-5}$
CAMB3LYP	$8.24 \cdot 10^{-8}$	CAMB3LYP	$6.25 \cdot 10^{-7}$	BH&H	$6.10 \cdot 10^{-6}$	BLYP	$8.06 \cdot 10^{-5}$
SPW92	$8.30 \cdot 10^{-8}$	B3LYP	$6.79 \cdot 10^{-7}$	B3LYP	$6.34 \cdot 10^{-6}$	LC-BLYP	$8.08 \cdot 10^{-5}$
SVWN5	$8.36 \cdot 10^{-8}$	SPW92	$7.23 \cdot 10^{-7}$	SPW92	$6.49 \cdot 10^{-6}$	BH&HLYP	$8.10 \cdot 10^{-5}$
B1LYP	$8.40 \cdot 10^{-8}$	SVWN5	$7.27 \cdot 10^{-7}$	SVWN5	$6.54 \cdot 10^{-6}$	BH&H	$8.21 \cdot 10^{-5}$
RevTPSS	$6.67 \cdot 10^{-7}$	RevTPSS	$6.18 \cdot 10^{-6}$	RevTPSS	$6.98 \cdot 10^{-5}$	RevTPSS	$7.64 \cdot 10^{-4}$
B97M-V	$8.46 \cdot 10^{-7}$	B97M-V	$7.87 \cdot 10^{-6}$	PW91	$8.77 \cdot 10^{-5}$	PW91	$9.38 \cdot 10^{-4}$
PW91	$8.59 \cdot 10^{-7}$	PW91	$8.11 \cdot 10^{-6}$	PBE	$8.92 \cdot 10^{-5}$	PBE	$9.58 \cdot 10^{-4}$
PBE	$8.73 \cdot 10^{-7}$	PBE	$8.19 \cdot 10^{-6}$	mPW91	$9.11 \cdot 10^{-5}$	mPW91	$9.73 \cdot 10^{-4}$
mPW91	$8.93 \cdot 10^{-7}$	mPW91	$8.49 \cdot 10^{-6}$	B97M-V	$9.74 \cdot 10^{-5}$	LC-wPBE	$1.07 \cdot 10^{-3}$
wB97X-V	$9.66 \cdot 10^{-7}$	LC-wPBE	$9.21 \cdot 10^{-6}$	LC-wPBE	$1.00 \cdot 10^{-4}$	PBE0	$1.08 \cdot 10^{-3}$
LC-wPBE	$9.76 \cdot 10^{-7}$	PBE0	$9.30 \cdot 10^{-6}$	PBE0	$1.01 \cdot 10^{-4}$	B1PW91	$1.10 \cdot 10^{-3}$
PBE0	$9.91 \cdot 10^{-7}$	TPSS	$9.64 \cdot 10^{-6}$	B1PW91	$1.04 \cdot 10^{-4}$	TPSS	$1.13 \cdot 10^{-3}$
TPSS	$1.02 \cdot 10^{-6}$	B1PW91	$9.68 \cdot 10^{-6}$	TPSS	$1.06 \cdot 10^{-4}$	TPSSh	$1.16 \cdot 10^{-3}$
B1PW91	$1.02 \cdot 10^{-6}$	TPSSh	$1.00 \cdot 10^{-5}$	TPSSh	$1.09 \cdot 10^{-4}$	PBE50	$1.19 \cdot 10^{-3}$
TPSSh	$1.06 \cdot 10^{-6}$	PBE50	$1.03 \cdot 10^{-5}$	PBE50	$1.12 \cdot 10^{-4}$	B97M-V	$2.06 \cdot 10^{-3}$
PBE50	$1.10 \cdot 10^{-6}$	wB97X-V	$1.15 \cdot 10^{-5}$	wB97X-V	$1.92 \cdot 10^{-4}$	wB97X-V	$4.95 \cdot 10^{-3}$
wB97M-V	$2.30 \cdot 10^{-6}$	wB97M-V	$2.57 \cdot 10^{-5}$	SOGGA11-X	$3.27 \cdot 10^{-4}$	M11	$5.77 \cdot 10^{-3}$
B97	$3.89 \cdot 10^{-6}$	SOGGA11-X	$3.65 \cdot 10^{-5}$	wB97M-V	$4.48 \cdot 10^{-4}$	SOGGA11-X	$6.28 \cdot 10^{-3}$
SOGGA11-X	$4.00 \cdot 10^{-6}$	SCAN0	$4.37 \cdot 10^{-5}$	M11	$4.91 \cdot 10^{-4}$	MN15	$8.56 \cdot 10^{-3}$
SCAN	$4.03 \cdot 10^{-6}$	SCAN	$4.41 \cdot 10^{-5}$	MN15	$7.71 \cdot 10^{-4}$	MN12-SX	$1.32 \cdot 10^{-2}$
SCAN0	$4.09 \cdot 10^{-6}$	M11	$4.46 \cdot 10^{-5}$	SOGGA11	$8.06 \cdot 10^{-4}$	MN15-L	$1.39 \cdot 10^{-2}$
M06-2X	$4.80 \cdot 10^{-6}$	M06-2X	$4.65 \cdot 10^{-5}$	M06-2X	$8.36 \cdot 10^{-4}$	MN12-L	$1.42 \cdot 10^{-2}$
N12-SX	$5.15 \cdot 10^{-6}$	B97	$5.37 \cdot 10^{-5}$	N12-SX	$8.99 \cdot 10^{-4}$	wB97M-V	$1.43 \cdot 10^{-2}$
wB97X	$5.35 \cdot 10^{-6}$	N12-SX	$5.84 \cdot 10^{-5}$	SCAN0	$9.86 \cdot 10^{-4}$	M11-L	$1.63 \cdot 10^{-2}$
wB97X-D3	$5.35 \cdot 10^{-6}$	wB97X	$6.00 \cdot 10^{-5}$	SCAN	$1.02 \cdot 10^{-3}$	SOGGA11	$1.93 \cdot 10^{-2}$
M11	$5.44 \cdot 10^{-6}$	B97-D	$6.62 \cdot 10^{-5}$	B97-D	$1.06 \cdot 10^{-3}$	M06-2X	$2.72 \cdot 10^{-2}$
wB97	$6.54 \cdot 10^{-6}$	MN15	$6.64 \cdot 10^{-5}$	MN12-SX	$1.16 \cdot 10^{-3}$	N12-SX	$3.05 \cdot 10^{-2}$
B97-D	$7.06 \cdot 10^{-6}$	wB97X-D3	$6.70 \cdot 10^{-5}$	M11-L	$1.24 \cdot 10^{-3}$	B97-D	$3.51 \cdot 10^{-2}$
MN15	$7.17 \cdot 10^{-6}$	wB97	$7.71 \cdot 10^{-5}$	B97	$1.31 \cdot 10^{-3}$	VSXC	$4.02 \cdot 10^{-2}$
wB97X-D	$7.83 \cdot 10^{-6}$	SOGGA11	$7.75 \cdot 10^{-5}$	MN15-L	$1.32 \cdot 10^{-3}$	B97	$5.00 \cdot 10^{-2}$
SOGGA11	$8.57 \cdot 10^{-6}$	N12	$9.54 \cdot 10^{-5}$	MN12-L	$1.33 \cdot 10^{-3}$	SCAN0	$5.68 \cdot 10^{-2}$
N12	$8.77 \cdot 10^{-6}$	wB97X-D	$1.00 \cdot 10^{-4}$	wB97X	$1.67 \cdot 10^{-3}$	SCAN	$5.71 \cdot 10^{-2}$
MN12-SX	$1.11 \cdot 10^{-5}$	MN12-SX	$1.07 \cdot 10^{-4}$	wB97	$1.81 \cdot 10^{-3}$	M06-HF	$5.98 \cdot 10^{-2}$
M11-L	$1.14 \cdot 10^{-5}$	M11-L	$1.10 \cdot 10^{-4}$	wB97X-D3	$1.94 \cdot 10^{-3}$	M06-L	$7.01 \cdot 10^{-2}$
MN15-L	$1.27 \cdot 10^{-5}$	MN15-L	$1.15 \cdot 10^{-4}$	M06-HF	$2.17 \cdot 10^{-3}$	M06	$7.21 \cdot 10^{-2}$
MN12-L	$1.28 \cdot 10^{-5}$	MN12-L	$1.20 \cdot 10^{-4}$	M06-L	$2.17 \cdot 10^{-3}$	wB97	$7.82 \cdot 10^{-2}$
M06-HF	$1.52 \cdot 10^{-5}$	M06-HF	$1.47 \cdot 10^{-4}$	wB97X-D	$2.90 \cdot 10^{-3}$	wB97X	$9.26 \cdot 10^{-2}$
M06-L	$1.59 \cdot 10^{-5}$	M06-L	$1.55 \cdot 10^{-4}$	N12	$2.98 \cdot 10^{-3}$	wB97X-D3	$1.11 \cdot 10^{-1}$
VSXC	$3.88 \cdot 10^{-5}$	VSXC	$3.68 \cdot 10^{-4}$	VSXC	$3.94 \cdot 10^{-3}$	N12	$1.61 \cdot 10^{-1}$
M06	$4.09 \cdot 10^{-5}$	M06	$4.16 \cdot 10^{-4}$	M06	$4.76 \cdot 10^{-3}$	wB97X-D	$1.63 \cdot 10^{-1}$

Table S28: RMSE values for various exchange energy derivatives obtained with different functionals, averaged across all systems and grids.

DFA	$d^1E_x/d\xi^1$	DFA	$d^2E_x/d\xi^2$	DFA	$d^3E_x/d\xi^3$	DFA	$d^4E_x/d\xi^4$
BH&H	$3.74 \cdot 10^{-5}$	BH&H	$3.18 \cdot 10^{-5}$	PBE50	$9.99 \cdot 10^{-6}$	BH&H	$4.48 \cdot 10^{-6}$
BH&HLYP	$5.97 \cdot 10^{-5}$	CAMB3LYP	$4.14 \cdot 10^{-5}$	BH&HLYP	$1.00 \cdot 10^{-5}$	SOGGA11-X	$5.94 \cdot 10^{-6}$
LC-BLYP	$5.98 \cdot 10^{-5}$	SOGGA11-X	$4.67 \cdot 10^{-5}$	B97	$1.16 \cdot 10^{-5}$	PBE50	$6.26 \cdot 10^{-6}$
CAMB3LYP	$6.83 \cdot 10^{-5}$	wB97X-V	$5.07 \cdot 10^{-5}$	B1LYP	$1.21 \cdot 10^{-5}$	B97	$6.57 \cdot 10^{-6}$
PBE50	$7.10 \cdot 10^{-5}$	BHHLYP	$5.08 \cdot 10^{-5}$	CAMB3LYP	$1.23 \cdot 10^{-5}$	BH&HLYP	$7.06 \cdot 10^{-6}$
SPW92	$7.44 \cdot 10^{-5}$	PBE50	$5.42 \cdot 10^{-5}$	wB97X-V	$1.30 \cdot 10^{-5}$	LC-BLYP	$7.11 \cdot 10^{-6}$
SVWN5	$7.44 \cdot 10^{-5}$	LC-BLYP	$5.51 \cdot 10^{-5}$	LC-BLYP	$1.40 \cdot 10^{-5}$	LC-wPBE	$7.70 \cdot 10^{-6}$
B97	$8.23 \cdot 10^{-5}$	wB97M-V	$5.53 \cdot 10^{-5}$	BHH	$1.40 \cdot 10^{-5}$	wB97X-D3	$7.70 \cdot 10^{-6}$
wB97X-V	$8.49 \cdot 10^{-5}$	RevTPSS	$5.57 \cdot 10^{-5}$	SOGGA11-X	$1.41 \cdot 10^{-5}$	wB97	$7.72 \cdot 10^{-6}$
B1LYP	$8.78 \cdot 10^{-5}$	B97	$5.69 \cdot 10^{-5}$	wB97X-D3	$1.45 \cdot 10^{-5}$	wB97X	$7.76 \cdot 10^{-6}$
wB97X-D3	$8.90 \cdot 10^{-5}$	LC-wPBE	$5.90 \cdot 10^{-5}$	wB97	$1.45 \cdot 10^{-5}$	CAMB3LYP	$7.90 \cdot 10^{-6}$
B3LYP	$8.93 \cdot 10^{-5}$	wB97	$6.15 \cdot 10^{-5}$	B3LYP	$1.50 \cdot 10^{-5}$	SPW92	$8.25 \cdot 10^{-6}$
B1PW91	$9.06 \cdot 10^{-5}$	wB97X	$6.23 \cdot 10^{-5}$	wB97X	$1.51 \cdot 10^{-5}$	SVWN5	$8.26 \cdot 10^{-6}$
wB97X	$9.50 \cdot 10^{-5}$	SPW92	$6.28 \cdot 10^{-5}$	wB97X-D	$1.53 \cdot 10^{-5}$	wB97X-V	$8.66 \cdot 10^{-6}$
SOGGA11-X	$9.94 \cdot 10^{-5}$	SVWN5	$6.32 \cdot 10^{-5}$	PBE0	$1.59 \cdot 10^{-5}$	wB97X-D	$8.72 \cdot 10^{-6}$
wB97M-V	$1.01 \cdot 10^{-4}$	wB97X-D3	$6.41 \cdot 10^{-5}$	B1PW91	$1.68 \cdot 10^{-5}$	PBE0	$9.11 \cdot 10^{-6}$
PBE0	$1.06 \cdot 10^{-4}$	B1LYP	$6.61 \cdot 10^{-5}$	wB97M-V	$1.70 \cdot 10^{-5}$	B1LYP	$1.03 \cdot 10^{-5}$
BLYP	$1.15 \cdot 10^{-4}$	B3LYP	$6.64 \cdot 10^{-5}$	LC-wPBE	$1.74 \cdot 10^{-5}$	B3LYP	$1.05 \cdot 10^{-5}$
wB97X-D	$1.28 \cdot 10^{-4}$	TPSSh	$6.96 \cdot 10^{-5}$	SPW92	$1.93 \cdot 10^{-5}$	B1PW91	$1.05 \cdot 10^{-5}$
LC-wPBE	$1.36 \cdot 10^{-4}$	wB97X-D	$7.34 \cdot 10^{-5}$	SVWN5	$1.93 \cdot 10^{-5}$	PBE	$1.22 \cdot 10^{-5}$
PBE	$1.39 \cdot 10^{-4}$	TPSS	$7.51 \cdot 10^{-5}$	BLYP	$2.17 \cdot 10^{-5}$	wB97M-V	$1.23 \cdot 10^{-5}$
wB97	$1.57 \cdot 10^{-4}$	PBE0	$7.82 \cdot 10^{-5}$	PBE	$2.49 \cdot 10^{-5}$	BLYP	$1.33 \cdot 10^{-5}$
B97-D	$3.34 \cdot 10^{-4}$	BLYP	$8.03 \cdot 10^{-5}$	B97-D	$2.88 \cdot 10^{-5}$	VSXC	$1.95 \cdot 10^{-5}$
MN15	$3.81 \cdot 10^{-4}$	B1PW91	$8.12 \cdot 10^{-5}$	B97M-V	$3.16 \cdot 10^{-5}$	B97-D	$1.95 \cdot 10^{-5}$
N12	$4.03 \cdot 10^{-4}$	N12-SX	$9.01 \cdot 10^{-5}$	TPSSh	$3.53 \cdot 10^{-5}$	B97M-V	$2.22 \cdot 10^{-5}$
N12-SX	$4.70 \cdot 10^{-4}$	PBE	$9.96 \cdot 10^{-5}$	RevTPSS	$3.76 \cdot 10^{-5}$	N12-SX	$2.30 \cdot 10^{-5}$
B97M-V	$4.83 \cdot 10^{-4}$	PW91	$1.35 \cdot 10^{-4}$	N12-SX	$3.92 \cdot 10^{-5}$	RevTPSS	$2.98 \cdot 10^{-5}$
PW91	$4.87 \cdot 10^{-4}$	mPW91	$1.36 \cdot 10^{-4}$	N12	$4.15 \cdot 10^{-5}$	SOGGA11	$3.08 \cdot 10^{-5}$
mPW91	$4.99 \cdot 10^{-4}$	B97M-V	$1.80 \cdot 10^{-4}$	MN15	$4.17 \cdot 10^{-5}$	TPSSh	$3.28 \cdot 10^{-5}$
RevTPSS	$6.24 \cdot 10^{-4}$	B97-D	$1.82 \cdot 10^{-4}$	VSXC	$4.34 \cdot 10^{-5}$	PW91	$3.49 \cdot 10^{-5}$
TPSSh	$7.09 \cdot 10^{-4}$	MN15-L	$1.94 \cdot 10^{-4}$	TPSS	$4.41 \cdot 10^{-5}$	MN15-L	$3.63 \cdot 10^{-5}$
SOGGA11	$7.36 \cdot 10^{-4}$	VSXC	$2.09 \cdot 10^{-4}$	SOGGA11	$4.62 \cdot 10^{-5}$	N12	$3.65 \cdot 10^{-5}$
TPSS	$7.71 \cdot 10^{-4}$	M06	$3.00 \cdot 10^{-4}$	mPW91	$4.85 \cdot 10^{-5}$	TPSS	$3.67 \cdot 10^{-5}$
VSXC	$1.06 \cdot 10^{-3}$	N12	$3.17 \cdot 10^{-4}$	PW91	$5.38 \cdot 10^{-5}$	MN15	$3.73 \cdot 10^{-5}$
MN15-L	$1.53 \cdot 10^{-3}$	M11	$3.20 \cdot 10^{-4}$	MN15-L	$6.67 \cdot 10^{-5}$	mPW91	$4.41 \cdot 10^{-5}$
MN12-SX	$3.70 \cdot 10^{-3}$	M06-2X	$3.51 \cdot 10^{-4}$	M06	$1.73 \cdot 10^{-4}$	MN12-L	$9.84 \cdot 10^{-5}$
M06	$3.77 \cdot 10^{-3}$	MN15	$3.58 \cdot 10^{-4}$	MN12-L	$1.78 \cdot 10^{-4}$	MN12-SX	$1.50 \cdot 10^{-4}$
MN12-L	$4.08 \cdot 10^{-3}$	SOGGA11	$4.14 \cdot 10^{-4}$	MN12-SX	$1.93 \cdot 10^{-4}$	M06	$1.51 \cdot 10^{-4}$
M06-L	$4.58 \cdot 10^{-3}$	M06-L	$5.32 \cdot 10^{-4}$	M11	$2.22 \cdot 10^{-4}$	M11	$1.84 \cdot 10^{-4}$
M06-2X	$5.19 \cdot 10^{-3}$	MN12-L	$7.49 \cdot 10^{-4}$	M06-L	$2.37 \cdot 10^{-4}$	M06-L	$1.99 \cdot 10^{-4}$
M11	$5.19 \cdot 10^{-3}$	M06-HF	$8.36 \cdot 10^{-4}$	M06-2X	$2.44 \cdot 10^{-4}$	M06-2X	$2.09 \cdot 10^{-4}$
M11-L	$9.96 \cdot 10^{-3}$	M11-L	$1.13 \cdot 10^{-3}$	M11-L	$4.32 \cdot 10^{-4}$	M11-L	$2.29 \cdot 10^{-4}$
M06-HF	$1.23 \cdot 10^{-2}$	MN12-SX	$1.79 \cdot 10^{-3}$	M06-HF	$5.84 \cdot 10^{-4}$	M06-HF	$5.18 \cdot 10^{-4}$
SCAN0	$3.77 \cdot 10^{-2}$	SCAN0	$6.94 \cdot 10^{-3}$	SCAN0	$5.32 \cdot 10^{-3}$	SCAN0	$4.21 \cdot 10^{-3}$
SCAN	$5.02 \cdot 10^{-2}$	SCAN	$1.10 \cdot 10^{-2}$	SCAN	$9.70 \cdot 10^{-3}$	SCAN	$4.96 \cdot 10^{-3}$

Table S29: RMSE values for various exchange-correlation energy derivatives obtained with different functionals, averaged across all systems and grids.

DFA	$d^1E_{xc}/d\xi^1$	DFA	$d^2E_{xc}/d\xi^2$	DFA	$d^3E_{xc}/d\xi^3$	DFA	$d^4E_{xc}/d\xi^4$
BHH	$3.75 \cdot 10^{-5}$	BHH	$3.24 \cdot 10^{-5}$	BHHLYP	$1.60 \cdot 10^{-5}$	SPW92	$7.58 \cdot 10^{-5}$
BHHLYP	$5.97 \cdot 10^{-5}$	CAMB3LYP	$4.20 \cdot 10^{-5}$	B1LYP	$1.82 \cdot 10^{-5}$	SVWN5	$7.65 \cdot 10^{-5}$
LC-BLYP	$5.99 \cdot 10^{-5}$	BHHLYP	$5.14 \cdot 10^{-5}$	CAMB3LYP	$1.84 \cdot 10^{-5}$	CAMB3LYP	$8.51 \cdot 10^{-5}$
CAMB3LYP	$6.83 \cdot 10^{-5}$	LC-BLYP	$5.57 \cdot 10^{-5}$	LC-BLYP	$2.00 \cdot 10^{-5}$	BHH	$8.66 \cdot 10^{-5}$
PBE50	$7.21 \cdot 10^{-5}$	RevTPSS	$6.19 \cdot 10^{-5}$	BHH	$2.01 \cdot 10^{-5}$	LC-BLYP	$8.79 \cdot 10^{-5}$
SPW92	$7.44 \cdot 10^{-5}$	wB97X-V	$6.22 \cdot 10^{-5}$	B3LYP	$2.13 \cdot 10^{-5}$	BHHLYP	$8.81 \cdot 10^{-5}$
SVWN5	$7.45 \cdot 10^{-5}$	SPW92	$6.35 \cdot 10^{-5}$	SPW92	$2.58 \cdot 10^{-5}$	B3LYP	$9.06 \cdot 10^{-5}$
wB97X-V	$8.58 \cdot 10^{-5}$	SVWN5	$6.40 \cdot 10^{-5}$	SVWN5	$2.58 \cdot 10^{-5}$	B1LYP	$9.09 \cdot 10^{-5}$
B97	$8.62 \cdot 10^{-5}$	PBE50	$6.45 \cdot 10^{-5}$	BLYP	$2.78 \cdot 10^{-5}$	BLYP	$9.40 \cdot 10^{-5}$
B1LYP	$8.79 \cdot 10^{-5}$	B1LYP	$6.67 \cdot 10^{-5}$	RevTPSS	$1.07 \cdot 10^{-4}$	RevTPSS	$7.94 \cdot 10^{-4}$
B3LYP	$8.94 \cdot 10^{-5}$	B3LYP	$6.71 \cdot 10^{-5}$	PBE	$1.14 \cdot 10^{-4}$	PBE	$9.71 \cdot 10^{-4}$
B1PW91	$9.16 \cdot 10^{-5}$	LC-wPBE	$6.82 \cdot 10^{-5}$	PBE0	$1.17 \cdot 10^{-4}$	PW91	$9.73 \cdot 10^{-4}$
wB97X-D3	$9.43 \cdot 10^{-5}$	TPSSh	$7.96 \cdot 10^{-5}$	LC-wPBE	$1.17 \cdot 10^{-4}$	mPW91	$1.02 \cdot 10^{-3}$
wB97X	$1.00 \cdot 10^{-4}$	BLYP	$8.09 \cdot 10^{-5}$	B1PW91	$1.21 \cdot 10^{-4}$	LC-wPBE	$1.08 \cdot 10^{-3}$
wB97M-V	$1.03 \cdot 10^{-4}$	wB97M-V	$8.10 \cdot 10^{-5}$	PBE50	$1.22 \cdot 10^{-4}$	PBE0	$1.09 \cdot 10^{-3}$
SOGGA11-X	$1.03 \cdot 10^{-4}$	SOGGA11-X	$8.32 \cdot 10^{-5}$	B97M-V	$1.29 \cdot 10^{-4}$	B1PW91	$1.11 \cdot 10^{-3}$
PBE0	$1.07 \cdot 10^{-4}$	TPSS	$8.47 \cdot 10^{-5}$	mPW91	$1.40 \cdot 10^{-4}$	TPSS	$1.16 \cdot 10^{-3}$
BLYP	$1.15 \cdot 10^{-4}$	PBE0	$8.75 \cdot 10^{-5}$	PW91	$1.41 \cdot 10^{-4}$	TPSSh	$1.20 \cdot 10^{-3}$
wB97X-D	$1.35 \cdot 10^{-4}$	B1PW91	$9.09 \cdot 10^{-5}$	TPSSh	$1.45 \cdot 10^{-4}$	PBE50	$1.20 \cdot 10^{-3}$
LC-wPBE	$1.37 \cdot 10^{-4}$	PBE	$1.08 \cdot 10^{-4}$	TPSS	$1.50 \cdot 10^{-4}$	B97M-V	$2.08 \cdot 10^{-3}$
PBE	$1.40 \cdot 10^{-4}$	B97	$1.11 \cdot 10^{-4}$	wB97X-V	$2.05 \cdot 10^{-4}$	wB97X-V	$4.96 \cdot 10^{-3}$
wB97	$1.63 \cdot 10^{-4}$	wB97X	$1.22 \cdot 10^{-4}$	SOGGA11-X	$3.41 \cdot 10^{-4}$	M11	$5.96 \cdot 10^{-3}$
B97-D	$3.41 \cdot 10^{-4}$	wB97X-D3	$1.31 \cdot 10^{-4}$	wB97M-V	$4.65 \cdot 10^{-4}$	SOGGA11-X	$6.28 \cdot 10^{-3}$
MN15	$3.88 \cdot 10^{-4}$	wB97	$1.39 \cdot 10^{-4}$	M11	$7.13 \cdot 10^{-4}$	MN15	$8.59 \cdot 10^{-3}$
N12	$4.11 \cdot 10^{-4}$	PW91	$1.44 \cdot 10^{-4}$	MN15	$8.13 \cdot 10^{-4}$	MN12-SX	$1.34 \cdot 10^{-2}$
N12-SX	$4.75 \cdot 10^{-4}$	mPW91	$1.45 \cdot 10^{-4}$	SOGGA11	$8.52 \cdot 10^{-4}$	MN15-L	$1.39 \cdot 10^{-2}$
B97M-V	$4.84 \cdot 10^{-4}$	N12-SX	$1.49 \cdot 10^{-4}$	N12-SX	$9.38 \cdot 10^{-4}$	MN12-L	$1.43 \cdot 10^{-2}$
PW91	$4.88 \cdot 10^{-4}$	wB97X-D	$1.74 \cdot 10^{-4}$	M06-2X	$1.08 \cdot 10^{-3}$	wB97M-V	$1.43 \cdot 10^{-2}$
mPW91	$5.00 \cdot 10^{-4}$	B97M-V	$1.88 \cdot 10^{-4}$	B97-D	$1.09 \cdot 10^{-3}$	M11-L	$1.65 \cdot 10^{-2}$
RevTPSS	$6.25 \cdot 10^{-4}$	B97-D	$2.48 \cdot 10^{-4}$	B97	$1.32 \cdot 10^{-3}$	SOGGA11	$1.93 \cdot 10^{-2}$
TPSSh	$7.10 \cdot 10^{-4}$	MN15-L	$3.09 \cdot 10^{-4}$	MN12-SX	$1.35 \cdot 10^{-3}$	M06-2X	$2.74 \cdot 10^{-2}$
SOGGA11	$7.45 \cdot 10^{-4}$	M11	$3.64 \cdot 10^{-4}$	MN15-L	$1.38 \cdot 10^{-3}$	N12-SX	$3.05 \cdot 10^{-2}$
TPSS	$7.72 \cdot 10^{-4}$	M06-2X	$3.98 \cdot 10^{-4}$	MN12-L	$1.51 \cdot 10^{-3}$	B97-D	$3.51 \cdot 10^{-2}$
VSXC	$1.10 \cdot 10^{-3}$	N12	$4.12 \cdot 10^{-4}$	M11-L	$1.67 \cdot 10^{-3}$	VSXC	$4.03 \cdot 10^{-2}$
MN15-L	$1.54 \cdot 10^{-3}$	MN15	$4.24 \cdot 10^{-4}$	wB97X	$1.69 \cdot 10^{-3}$	B97	$5.00 \cdot 10^{-2}$
MN12-SX	$3.71 \cdot 10^{-3}$	SOGGA11	$4.91 \cdot 10^{-4}$	wB97	$1.82 \cdot 10^{-3}$	M06-HF	$6.03 \cdot 10^{-2}$
M06	$3.81 \cdot 10^{-3}$	VSXC	$5.77 \cdot 10^{-4}$	wB97X-D3	$1.95 \cdot 10^{-3}$	SCAN0	$6.11 \cdot 10^{-2}$
MN12-L	$4.09 \cdot 10^{-3}$	M06-L	$6.88 \cdot 10^{-4}$	M06-L	$2.41 \cdot 10^{-3}$	SCAN	$6.21 \cdot 10^{-2}$
M06-L	$4.59 \cdot 10^{-3}$	M06	$7.16 \cdot 10^{-4}$	M06-HF	$2.75 \cdot 10^{-3}$	M06-L	$7.03 \cdot 10^{-2}$
M06-2X	$5.19 \cdot 10^{-3}$	MN12-L	$8.70 \cdot 10^{-4}$	wB97X-D	$2.91 \cdot 10^{-3}$	M06	$7.23 \cdot 10^{-2}$
M11	$5.19 \cdot 10^{-3}$	M06-HF	$9.83 \cdot 10^{-4}$	N12	$3.02 \cdot 10^{-3}$	wB97	$7.82 \cdot 10^{-2}$
M11-L	$9.97 \cdot 10^{-3}$	M11-L	$1.24 \cdot 10^{-3}$	VSXC	$3.98 \cdot 10^{-3}$	wB97X	$9.26 \cdot 10^{-2}$
M06-HF	$1.23 \cdot 10^{-2}$	MN12-SX	$1.90 \cdot 10^{-3}$	M06	$4.93 \cdot 10^{-3}$	wB97X-D3	$1.11 \cdot 10^{-1}$
SCAN0	$3.77 \cdot 10^{-2}$	SCAN0	$6.98 \cdot 10^{-3}$	SCAN0	$6.31 \cdot 10^{-3}$	N12	$1.61 \cdot 10^{-1}$
SCAN	$5.02 \cdot 10^{-2}$	SCAN	$1.10 \cdot 10^{-2}$	SCAN	$1.07 \cdot 10^{-2}$	wB97X-D	$1.63 \cdot 10^{-1}$

Table S30: Maximum absolute RMSE values for DFAs per molecular family. Molecular family A comprises $HCN \cdot HF$, $HCN \cdot HCl$, $OC \cdot HF$ and $N_2 \cdot HF$; B comprises $HCN \cdot BrF$, and C comprises Ar_2.

	A		B		C	
**DFA**	E_C	E_X	E_C	E_X	E_C	E_X
BHH	$1.27 \cdot 10^{-5}$	$4.59 \cdot 10^{-3}$	$2.04 \cdot 10^{-5}$	$6.16 \cdot 10^{-6}$	$6.92 \cdot 10^{-4}$	$3.34 \cdot 10^{-10}$
SPW92	$2.04 \cdot 10^{-6}$	$9.17 \cdot 10^{-3}$	$1.31 \cdot 10^{-5}$	$8.29 \cdot 10^{-6}$	$7.67 \cdot 10^{-4}$	$3.86 \cdot 10^{-10}$
SVWN5	$2.12 \cdot 10^{-6}$	$9.17 \cdot 10^{-3}$	$1.32 \cdot 10^{-5}$	$8.17 \cdot 10^{-6}$	$7.72 \cdot 10^{-4}$	$2.90 \cdot 10^{-10}$
BLYP	$5.83 \cdot 10^{-6}$	$1.33 \cdot 10^{-2}$	$1.87 \cdot 10^{-5}$	$2.10 \cdot 10^{-5}$	$6.96 \cdot 10^{-4}$	$1.11 \cdot 10^{-9}$
B1LYP	$5.11 \cdot 10^{-6}$	$1.03 \cdot 10^{-2}$	$1.60 \cdot 10^{-5}$	$1.00 \cdot 10^{-5}$	$6.96 \cdot 10^{-4}$	$8.92 \cdot 10^{-10}$
B3LYP	$4.25 \cdot 10^{-6}$	$1.05 \cdot 10^{-2}$	$1.94 \cdot 10^{-5}$	$1.21 \cdot 10^{-5}$	$7.35 \cdot 10^{-4}$	$1.44 \cdot 10^{-9}$
BHHLYP	$5.14 \cdot 10^{-6}$	$7.03 \cdot 10^{-3}$	$1.67 \cdot 10^{-5}$	$8.47 \cdot 10^{-6}$	$6.99 \cdot 10^{-4}$	$9.07 \cdot 10^{-10}$
LC-BLYP	$8.65 \cdot 10^{-6}$	$6.95 \cdot 10^{-3}$	$1.76 \cdot 10^{-5}$	$1.16 \cdot 10^{-5}$	$6.93 \cdot 10^{-4}$	$4.53 \cdot 10^{-10}$
CAMB3LYP	$4.13 \cdot 10^{-6}$	$7.90 \cdot 10^{-3}$	$1.58 \cdot 10^{-5}$	$9.88 \cdot 10^{-6}$	$6.97 \cdot 10^{-4}$	$5.58 \cdot 10^{-10}$
PBE	$4.59 \cdot 10^{-5}$	$1.11 \cdot 10^{-2}$	$3.17 \cdot 10^{-5}$	$1.79 \cdot 10^{-5}$	$9.80 \cdot 10^{-3}$	$6.74 \cdot 10^{-10}$
PBE0	$4.61 \cdot 10^{-5}$	$8.63 \cdot 10^{-3}$	$2.05 \cdot 10^{-5}$	$1.01 \cdot 10^{-5}$	$1.12 \cdot 10^{-2}$	$4.35 \cdot 10^{-10}$
PBE50	$4.18 \cdot 10^{-5}$	$5.99 \cdot 10^{-3}$	$1.02 \cdot 10^{-5}$	$7.48 \cdot 10^{-6}$	$1.24 \cdot 10^{-2}$	$4.68 \cdot 10^{-10}$
LC-wPBE	$4.42 \cdot 10^{-5}$	$7.93 \cdot 10^{-3}$	$1.17 \cdot 10^{-5}$	$1.05 \cdot 10^{-5}$	$1.11 \cdot 10^{-2}$	$2.55 \cdot 10^{-9}$
TPSS	$5.40 \cdot 10^{-5}$	$1.22 \cdot 10^{-2}$	$2.20 \cdot 10^{-5}$	$6.65 \cdot 10^{-5}$	$1.17 \cdot 10^{-2}$	$8.68 \cdot 10^{-9}$
TPSSh	$5.33 \cdot 10^{-5}$	$1.12 \cdot 10^{-2}$	$1.86 \cdot 10^{-5}$	$5.81 \cdot 10^{-5}$	$1.22 \cdot 10^{-2}$	$6.22 \cdot 10^{-9}$
RevTPSS	$4.67 \cdot 10^{-5}$	$9.00 \cdot 10^{-3}$	$2.11 \cdot 10^{-5}$	$5.07 \cdot 10^{-5}$	$7.57 \cdot 10^{-3}$	$1.26 \cdot 10^{-9}$
PW91	$7.82 \cdot 10^{-5}$	$2.47 \cdot 10^{-2}$	$1.42 \cdot 10^{-5}$	$1.12 \cdot 10^{-4}$	$9.75 \cdot 10^{-3}$	$2.06 \cdot 10^{-8}$
mPW91	$6.59 \cdot 10^{-5}$	$2.54 \cdot 10^{-2}$	$2.97 \cdot 10^{-5}$	$1.12 \cdot 10^{-4}$	$1.02 \cdot 10^{-2}$	$2.45 \cdot 10^{-8}$
B1PW91	$4.77 \cdot 10^{-5}$	$1.07 \cdot 10^{-2}$	$3.40 \cdot 10^{-5}$	$1.25 \cdot 10^{-5}$	$1.16 \cdot 10^{-2}$	$4.18 \cdot 10^{-10}$
M11	$4.81 \cdot 10^{-4}$	$8.07 \cdot 10^{-2}$	$9.50 \cdot 10^{-5}$	$2.17 \cdot 10^{-4}$	$4.62 \cdot 10^{-2}$	$1.04 \cdot 10^{-7}$
M11-L	$5.31 \cdot 10^{-4}$	$1.79 \cdot 10^{-1}$	$1.66 \cdot 10^{-4}$	$4.26 \cdot 10^{-4}$	$1.36 \cdot 10^{-1}$	$1.91 \cdot 10^{-7}$
MN12-L	$4.39 \cdot 10^{-4}$	$9.38 \cdot 10^{-2}$	$7.71 \cdot 10^{-5}$	$1.19 \cdot 10^{-4}$	$1.45 \cdot 10^{-1}$	$3.25 \cdot 10^{-8}$
MN12-SX	$4.00 \cdot 10^{-4}$	$2.13 \cdot 10^{-1}$	$2.33 \cdot 10^{-5}$	$1.22 \cdot 10^{-4}$	$1.26 \cdot 10^{-1}$	$3.97 \cdot 10^{-8}$
B97	$3.94 \cdot 10^{-3}$	$6.36 \cdot 10^{-3}$	$8.10 \cdot 10^{-4}$	$6.97 \cdot 10^{-6}$	$3.44 \cdot 10^{-1}$	$4.37 \cdot 10^{-10}$
B97-D	$2.77 \cdot 10^{-3}$	$2.12 \cdot 10^{-2}$	$6.90 \cdot 10^{-4}$	$2.02 \cdot 10^{-5}$	$2.42 \cdot 10^{-1}$	$2.59 \cdot 10^{-9}$
wB97	$4.98 \cdot 10^{-3}$	$7.84 \cdot 10^{-3}$	$2.02 \cdot 10^{-3}$	$1.05 \cdot 10^{-5}$	$6.04 \cdot 10^{-1}$	$2.29 \cdot 10^{-9}$
wB97X	$6.75 \cdot 10^{-3}$	$7.49 \cdot 10^{-3}$	$3.62 \cdot 10^{-3}$	$1.26 \cdot 10^{-5}$	$6.86 \cdot 10^{-1}$	$8.10 \cdot 10^{-10}$
wB97X-D	$9.99 \cdot 10^{-3}$	$9.03 \cdot 10^{-3}$	$6.93 \cdot 10^{-3}$	$1.22 \cdot 10^{-5}$	$1.20 \cdot 10^{0}$	$8.53 \cdot 10^{-10}$
wB97X-D3	$7.23 \cdot 10^{-3}$	$7.82 \cdot 10^{-3}$	$4.40 \cdot 10^{-3}$	$1.24 \cdot 10^{-5}$	$8.23 \cdot 10^{-1}$	$4.57 \cdot 10^{-10}$
wB97X-V	$2.55 \cdot 10^{-4}$	$5.80 \cdot 10^{-3}$	$3.22 \cdot 10^{-5}$	$2.68 \cdot 10^{-5}$	$3.56 \cdot 10^{-2}$	$5.37 \cdot 10^{-10}$
wB97M-V	$3.19 \cdot 10^{-4}$	$1.19 \cdot 10^{-2}$	$2.33 \cdot 10^{-4}$	$1.49 \cdot 10^{-5}$	$9.94 \cdot 10^{-2}$	$4.93 \cdot 10^{-10}$
B97M-V	$4.79 \cdot 10^{-4}$	$2.13 \cdot 10^{-2}$	$1.43 \cdot 10^{-4}$	$4.00 \cdot 10^{-5}$	$1.51 \cdot 10^{-2}$	$4.96 \cdot 10^{-9}$
SCAN	$3.45 \cdot 10^{-2}$	$1.04 \cdot 10^{0}$	$3.80 \cdot 10^{-3}$	$1.53 \cdot 10^{-2}$	$3.59 \cdot 10^{-1}$	$2.45 \cdot 10^{-6}$
SCAN0	$2.97 \cdot 10^{-2}$	$7.82 \cdot 10^{-1}$	$3.02 \cdot 10^{-3}$	$1.15 \cdot 10^{-2}$	$3.63 \cdot 10^{-1}$	$1.53 \cdot 10^{-6}$
M06	$3.26 \cdot 10^{-3}$	$7.04 \cdot 10^{-2}$	$3.26 \cdot 10^{-4}$	$1.73 \cdot 10^{-4}$	$6.22 \cdot 10^{-1}$	$7.35 \cdot 10^{-8}$
M06-L	$4.94 \cdot 10^{-3}$	$9.52 \cdot 10^{-2}$	$2.60 \cdot 10^{-4}$	$4.65 \cdot 10^{-4}$	$4.86 \cdot 10^{-1}$	$1.14 \cdot 10^{-7}$
M06-2X	$2.67 \cdot 10^{-3}$	$9.08 \cdot 10^{-2}$	$6.38 \cdot 10^{-4}$	$2.57 \cdot 10^{-4}$	$1.86 \cdot 10^{-1}$	$1.05 \cdot 10^{-7}$
M06-HF	$4.75 \cdot 10^{-3}$	$2.32 \cdot 10^{-1}$	$9.62 \cdot 10^{-4}$	$6.02 \cdot 10^{-4}$	$4.66 \cdot 10^{-1}$	$2.62 \cdot 10^{-7}$
VSXC	$4.80 \cdot 10^{-4}$	$2.24 \cdot 10^{-2}$	$8.62 \cdot 10^{-5}$	$1.83 \cdot 10^{-5}$	$4.53 \cdot 10^{-1}$	$1.12 \cdot 10^{-8}$
MN15	$6.73 \cdot 10^{-4}$	$3.94 \cdot 10^{-2}$	$2.53 \cdot 10^{-5}$	$3.86 \cdot 10^{-5}$	$8.16 \cdot 10^{-2}$	$1.50 \cdot 10^{-9}$
MN15-L	$3.42 \cdot 10^{-4}$	$2.66 \cdot 10^{-2}$	$4.85 \cdot 10^{-5}$	$4.16 \cdot 10^{-5}$	$1.40 \cdot 10^{-1}$	$1.95 \cdot 10^{-8}$
SOGGA11	$1.31 \cdot 10^{-3}$	$5.80 \cdot 10^{-2}$	$6.88 \cdot 10^{-4}$	$4.62 \cdot 10^{-5}$	$1.15 \cdot 10^{-1}$	$6.78 \cdot 10^{-9}$
SOGGA11-X	$4.12 \cdot 10^{-4}$	$2.99 \cdot 10^{-3}$	$6.85 \cdot 10^{-5}$	$9.29 \cdot 10^{-6}$	$4.06 \cdot 10^{-2}$	$9.23 \cdot 10^{-10}$
N12	$1.35 \cdot 10^{-2}$	$3.94 \cdot 10^{-2}$	$5.68 \cdot 10^{-3}$	$4.65 \cdot 10^{-5}$	$1.25 \cdot 10^{0}$	$1.34 \cdot 10^{-8}$
N12-SX	$3.46 \cdot 10^{-3}$	$1.06 \cdot 10^{-2}$	$3.93 \cdot 10^{-4}$	$3.83 \cdot 10^{-5}$	$1.87 \cdot 10^{-1}$	$1.34 \cdot 10^{-8}$

S4 The role of integration grids

In this section, we analyze the performance of various schemes of numerical integration in density functional theory. In particular, we consider the radial numerical quadratures and the nuclear weighting schemes detailed in Table S31. The combination Becke-Stratmann (Becke's radial integration grid with Stratmann's weighting scheme) is the default used in Gaussian,[9] and produces oscillations of similar magnitude[44] to those reported in this paper with Handy-Becke integration (which is the default in QCHEM 5.1).[8] As we can see in Figure S25 for SCAN and Figures S27-S30 for other DFAs, employing other schemes such as Becke-Becke, Treutler-Treutler, Becke-Treutler increases the magnitude of the spurious oscillations compared to Becke-Stratmann. On the other hand, using Ochsenfeld's extension[45] of Becke's nuclear weighting scheme (SBecke) does show a significant improvement for SCAN, by reducing the magnitude of some oscillations (see Figures S25 and S26) for some segments of the property profile. These results can be extrapolated to all DFAs (compare Figure S31 to Figures S27-S30), where still we find spurious oscillations for many popular functionals such as ωB97X-D. The SBecke scheme was already shown to eliminate spurious oscillations in the binding curves of noncovalently bound systems,[45] however, in the present context it does not help avoid spurious oscillations in Ar_2 or He_2. Indeed, for some parts of the property profile, the Treutler-SBecke scheme presents similar oscillations to those of more conventional grids (see Figure S26). Hence, the latter is recommended whenever available (it is implemented in Psi4),[46–48] although it is not, in general, expected to get rid of spurious oscillations.

Table S31: Grid schemes tested. See Table S32 for the references and description of these grids.

Radial int. grid	Nuclear weighting
Becke	Becke
Treutler	Treutler
Becke	Treutler
Treutler	SBecke
Becke	Stratmann

Table S32: Information about the most common grid schemes in DFT calculations..

	Usual Name	Reference
Radial integration grid scheme	Becke	49
	Euler-Maclaurin (a.k.a. Handy)	50
	Ahlrichs (a.k.a. Treutler)	51
	Log (a.k.a Mura)	52
Nuclear weighting	Becke	49
	Ahlrichs (a.k.a. Treutler)	51
	Stratmann	53
	Sbecke	45

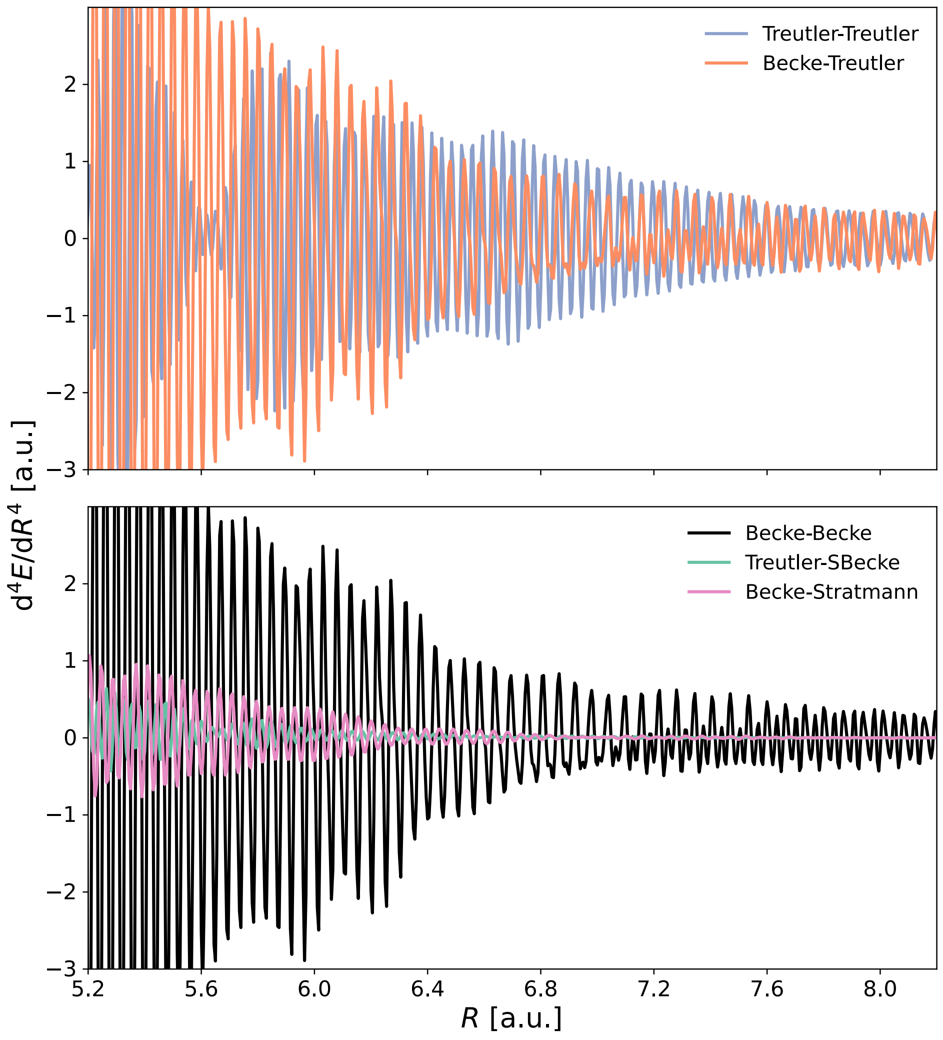

Figure S25: Spurious oscillations in the fourth derivative of total energy of Ar_2, obtained using SCAN and the (750,974) integration grid for the different grid schemes as defined in S31. The grid choices are represented as *Radial Grid Scheme - Nuclear weighting*.

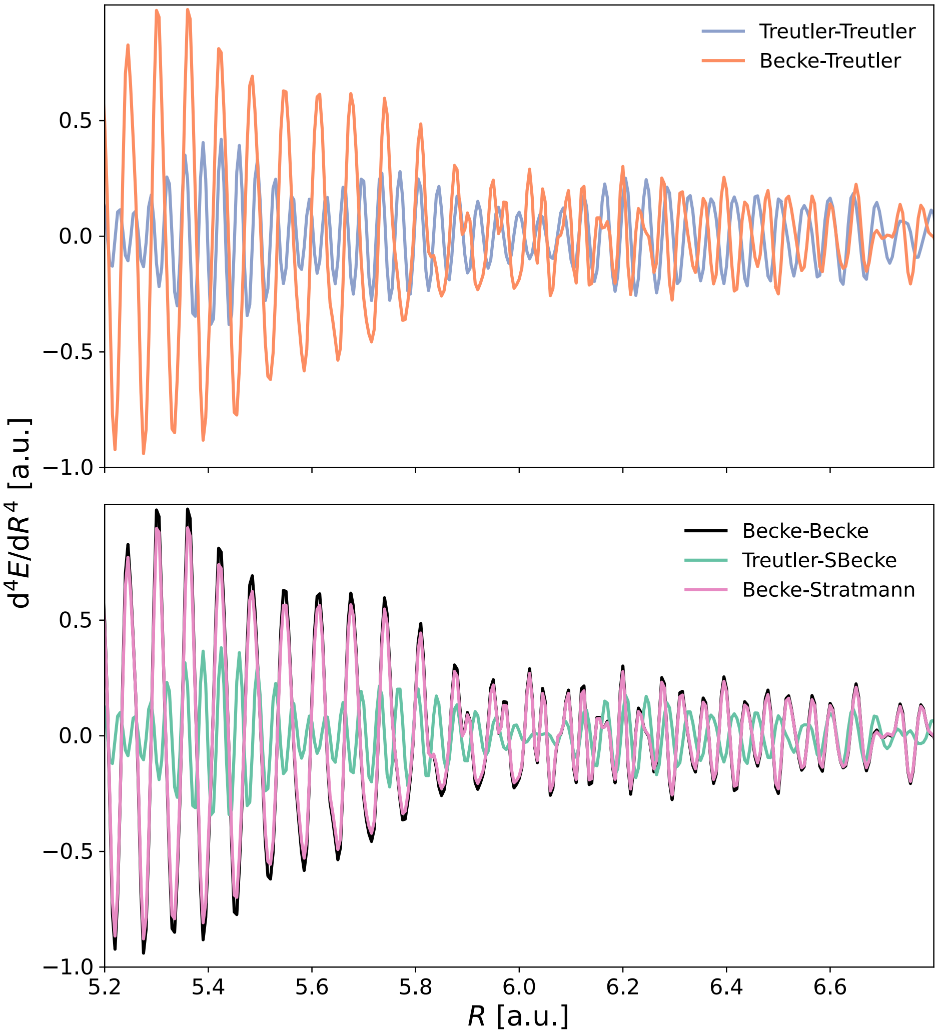

Figure S26: Spurious oscillations in the fourth derivative of total energy of He_2, obtained using SCAN and the (750,974) integration grid for the different grid schemes as defined in S31. The grid choices are represented as *Radial Grid Scheme - Nuclear weighting*.

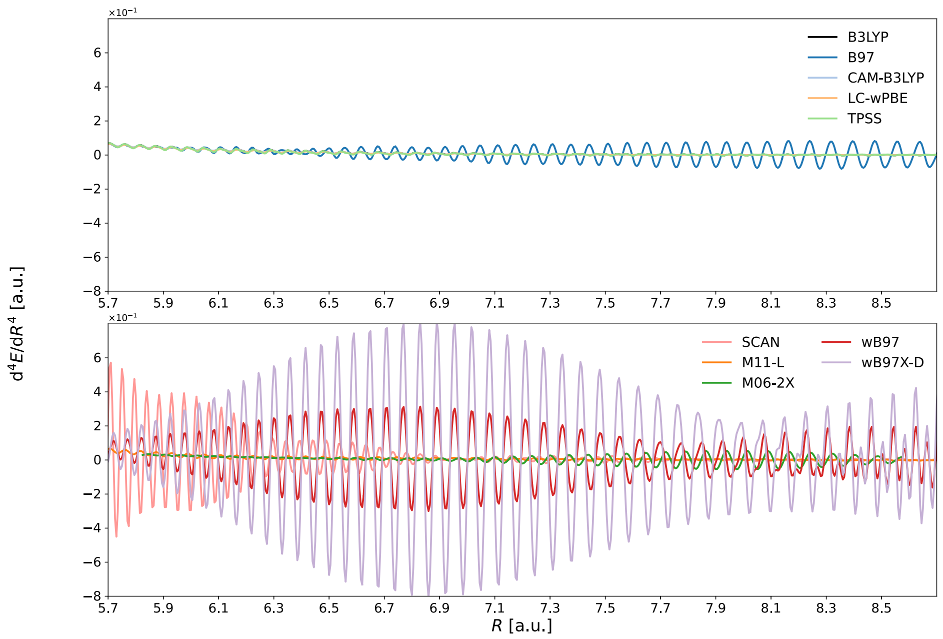

Figure S27: Spurious oscillations in the fourth derivative of total energy of Ar_2, obtained using several functionals and the (750,974) integration grid. The grid scheme is *Becke-Stramann*.

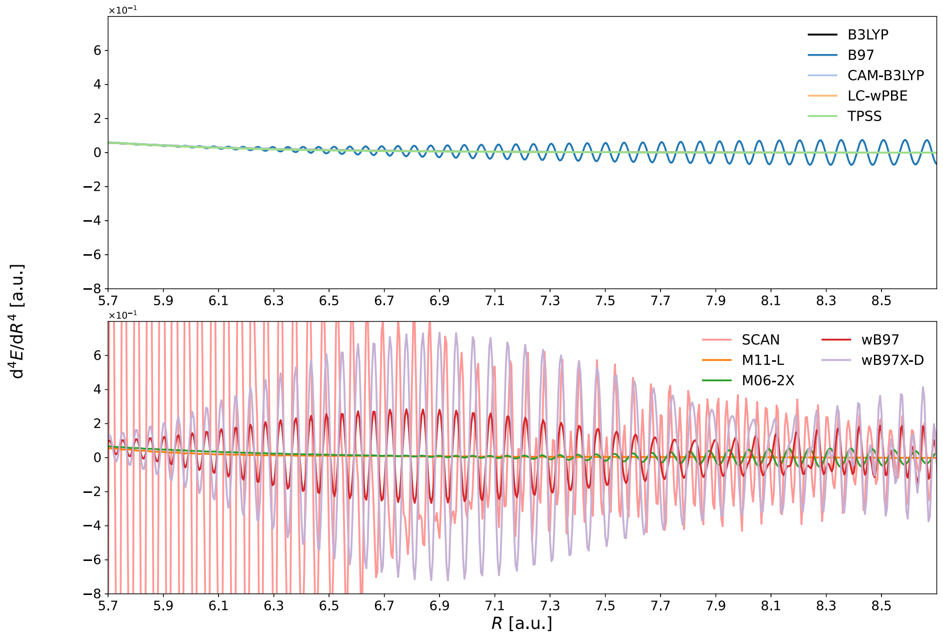

Figure S28: Spurious oscillations in the fourth derivative of total energy of Ar_2, obtained using several functionals and the (750,974) integration grid. The grid scheme is *Becke-Becke*.

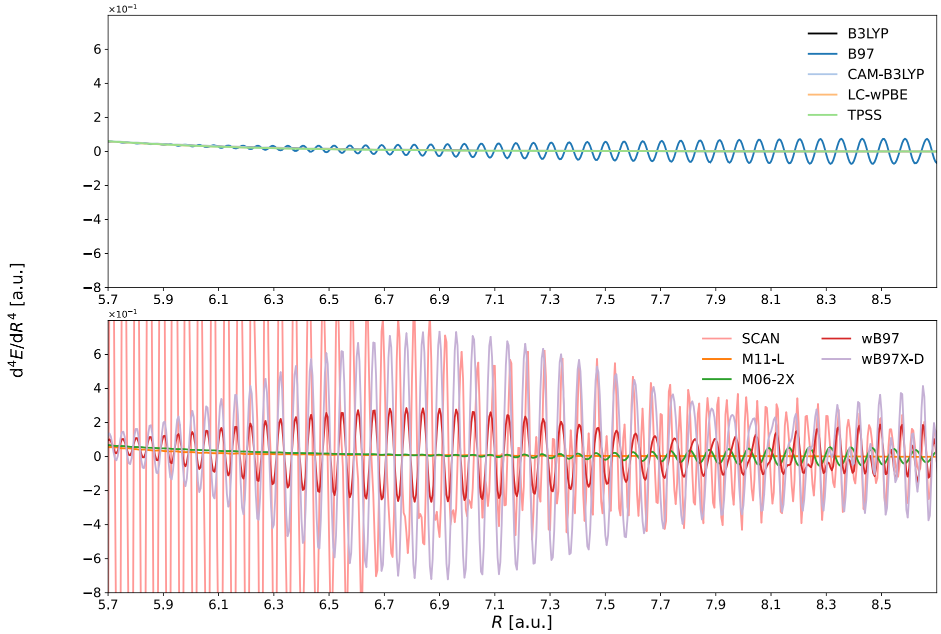

Figure S29: Spurious oscillations in the fourth derivative of total energy of Ar_2, obtained using several functionals and the (750,974) integration grid. The grid scheme is *Becke-Treutler*.

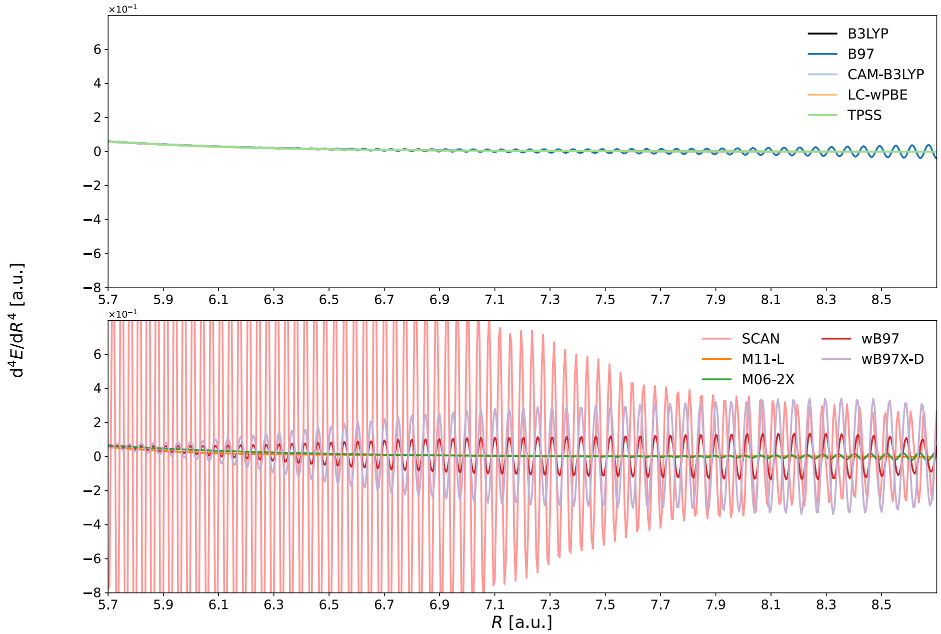

Figure S30: Spurious oscillations in the fourth derivative of total energy of Ar_2, obtained using several functionals and the (750,974) integration grid. The grid scheme is *Treutler-Treutler*.

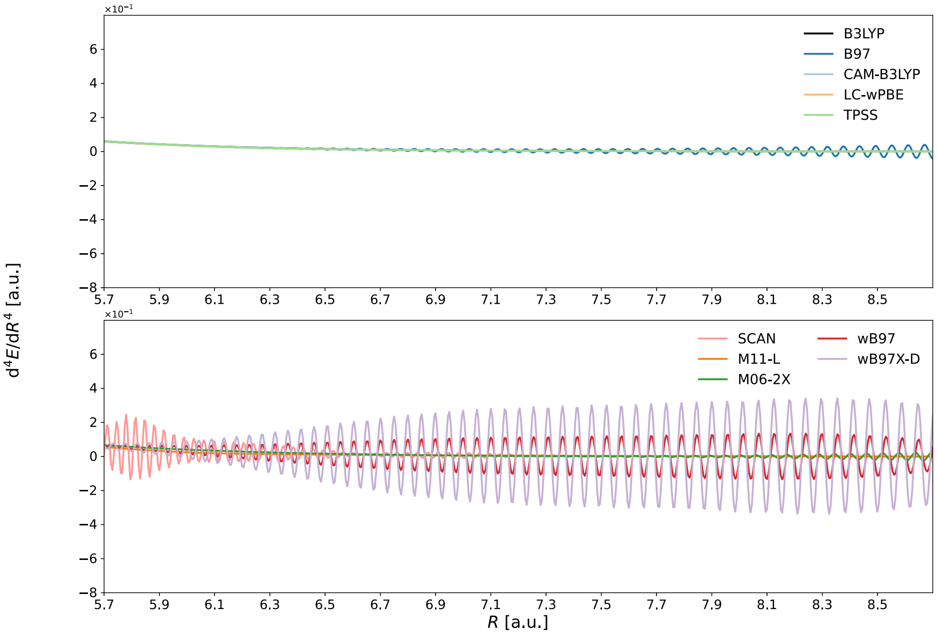

Figure S31: Spurious oscillations in the fourth derivative of total energy of Ar_2, obtained using several functionals and the (750,974) integration grid. The grid scheme is *Treutler-SBecke*.

S5 SCAN density functional approximations

SCAN[42] has long been known to be numerically unstable, which prompted the appearance of regularized variants, such as a rSCAN[54] and r2SCAN.[55] The latter variants of SCAN converge much faster to the grid limit and, hence, could be less prone to present spurious oscillations. While these variants are indeed well-behaved in some instances,[56] they have been also found to be numerically ill-behaved[57] in other situations. In this section, we study the proneness of rSCAN and r2SCAN to present spurious oscillations.

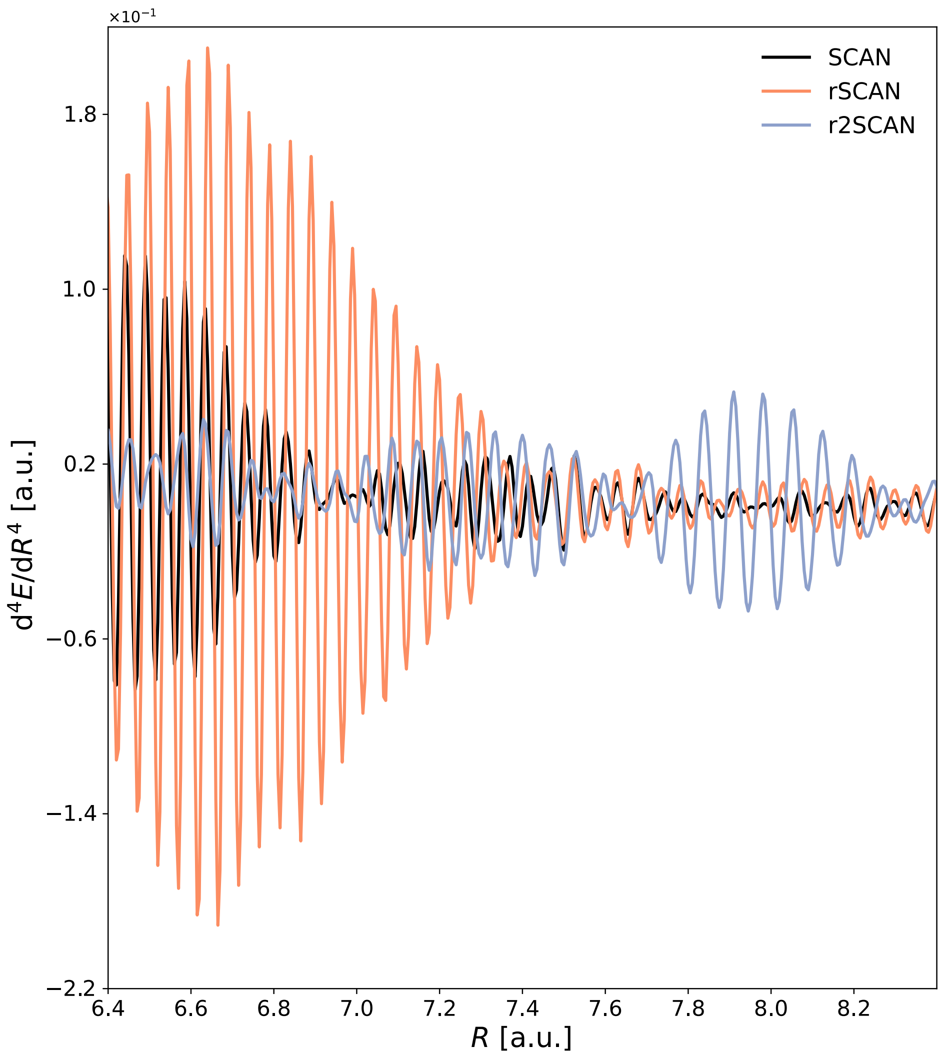

Figure S32: Spurious oscillations in the fourth derivative of total energy of Ar_2, obtained using SCAN, rSCAN and r2SCAN and the (750,974) integration grid. The grid scheme is *Becke-Stramann*.

rSCAN shows larger oscillations than native SCAN. However, we see a significant reduction in the magnitude of the oscillations using r2SCAN in some parts of the property profile (see Figure S32). Unfortunately, the magnitude of the oscillations of r2SCAN is still quite large compared to other DFAs (see Figure S27), and even larger than SCAN and rSCAN at large interatomic separations (see Figure S32). In this sense, this renormalized version of SCAN should still be used with caution, as is prone to present spurious oscillations in many cases (for a comparison, see Figure 4 in Ref. 44, where functionals such as LC-ωPBE that outperform r2SCAN in the present case were

classified as *highly grid dependent*).

As evident from Figure S33, the combined use of Ochsenfeld's extension[45] of Becke's nuclear weighting scheme (SBecke) with r2SCAN further reduces the spurious oscillations but it does not eliminate them.

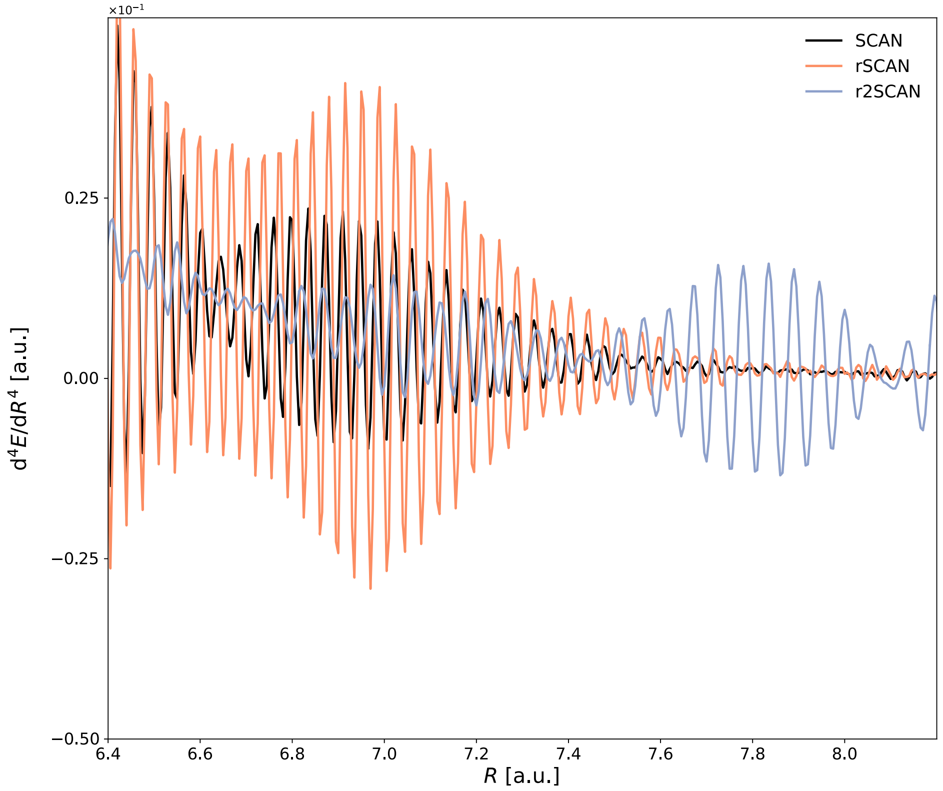

Figure S33: Spurious oscillations in the fourth derivative of total energy of Ar_2, obtained using SCAN, rSCAN and r2SCAN and the (750,974) integration grid. The grid scheme is *Treutler-SBecke*.

References

(1) Becke, A. D. Density-functional exchange-energy approximation with correct asymptotic behavior. *Phys. Rev. A* **1988**, *38*, 3098–3100.

(2) Lee, C.; Yang, W.; Parr, R. G. Development of the Colle-Salvetti correlation-energy formula into a functional of the electron density. *Phys. Rev. B* **1988**, *37*, 785–789.

(3) Perdew, J. P.; Burke, K.; Ernzerhof, M. Generalized gradient approximation made simple. *Phys. Rev. Lett.* **1996**, *77*, 3865–3868.

(4) Adamo, C.; Barone, V. Toward reliable adiabatic connection models free from adjustable parameters. *Chem. Phys. Lett.* **1997**, *274*, 242–250.

(5) Adamo, C.; Barone, V. Toward reliable density functional methods without adjustable parameters: The PBE0 model. *J. Chem. Phys.* **1999**, *110*, 6158–6170.

(6) Becke, A. D. Density-functional thermochemistry. III. The role of exact exchange. *J. Chem. Phys.* **1993**, *98*, 5648–5652.

(7) Stephens, P. J.; Devlin, F. J.; Chabalowski, C. F.; Frisch, M. J. Ab initio calculation of vibrational absorption and circular dichroism spectra using density functional force fields. *J. Phys. Chem.* **1994**, *98*, 11623–11627.

(8) Epifanovsky, E.; Gilbert, A. T. B.; Feng, X.; Lee, J.; Mao, Y.; Mardirossian, N.; Pokhilko, P.; White, A. F.; Coons, M. P.; Dempwolff, A. L. et al. Software for the frontiers of quantum chemistry: An overview of developments in the Q-Chem 5 package. *J. Chem. Phys.* **2021**, *155*, 084801.

(9) Frisch, M. J.; Trucks, G. W.; Schlegel, H. B.; Scuseria, G. E.; Robb, M. A.; Cheeseman, J. R.; Scalmani, G.; Barone, V.; Petersson, G. A.; Nakatsuji, H. et al. Gaussian 16 Revision C.01. 2016; Gaussian Inc. Wallingford CT.

(10) Rohrdanz, M. A.; Herbert, J. M. Simultaneous benchmarking of ground- and excited-state properties with long-range-corrected density functional theory. *J. Chem. Phys.* **2008**, *129*, 034107.

(11) Van Voorhis, T.; Scuseria, G. E. A novel form for the exchange-correlation energy functional. *J. Chem. Phys.* **1998**, *109*, 400–410.

(12) Tawada, Y.; Tsuneda, T.; Yanagisawa, S.; Yanai, T.; Hirao, K. A long-range-corrected time-dependent density functional theory. *J. Chem. Phys.* **2004**, *120*, 8425–8433.

(13) Becke, A. D. Density-functional thermochemistry. V. Systematic optimization of exchange-correlation functionals. *J. Chem. Phys.* **1997**, *107*, 8554–8560.

(14) Yanai, T.; Tew, D. P.; Handy, N. C. A new hybrid exchange–correlation functional using the Coulomb-attenuating method (CAM-B3LYP). *Chem. Phys. Lett.* **2004**, *393*, 51–57.

(15) Grimme, S. Semiempirical GGA-type density functional constructed with a long-range dispersion correction. *J. Comput. Chem.* **2006**, *27*, 1787–1799.

(16) Zhao, Y.; Truhlar, D. The M06 suite of density functionals for main group thermochemistry, thermochemical kinetics, noncovalent interactions, excited states, and transition elements: two new functionals and systematic testing of four M06-class functionals and 12 other functionals. *Theor. Chem. Account* **2008**, *120*, 215–241.

(17) Chai, J.-D.; Head-Gordon, M. Systematic optimization of long-range corrected hybrid density functionals. *J. Chem. Phys.* **2008**, *128*, 084106.

(18) Zhao, Y.; Truhlar, D. G. A new local density functional for main-group thermochemistry, transition metal bonding, thermochemical kinetics, and noncovalent interactions. *J. Chem. Phys.* **2006**, *125*, 194101.

(19) Zhao, Y.; Truhlar, D. G. Density functional for spectroscopy: no long-range self-interaction

error, good performance for Rydberg and charge-transfer states, and better performance on average than B3LYP for ground states. *J. Phys. Chem. A* **2006**, *110*, 13126–13130.

(20) Chai, J.-D.; Head-Gordon, M. Long-range corrected hybrid density functionals with damped atom–atom dispersion corrections. *Phys. Chem. Chem. Phys.* **2008**, *10*, 6615–6620.

(21) Lin, Y.-S.; Li, G.-D.; Mao, S.-P.; Chai, J.-D. Long-range corrected hybrid density functionals with improved dispersion corrections. *J. Chem. Theory Comput.* **2013**, *9*, 263–272.

(22) Peverati, R.; Zhao, Y.; Truhlar, D. G. Generalized gradient approximation that recovers the second-order density-gradient expansion with optimized across-the-board performance. *J. Phys. Chem. Lett.* **2011**, *2*, 1991–1997.

(23) Mardirossian, N.; Head-Gordon, M. Mapping the genome of meta-generalized gradient approximation density functionals: The search for B97M-V. *J. Chem. Phys.* **2015**, *142*, 074111.

(24) Peverati, R.; Truhlar, D. G. Communication: A global hybrid generalized gradient approximation to the exchange-correlation functional that satisfies the second-order density-gradient constraint and has broad applicability in chemistry. *J. Chem. Phys.* **2011**, *135*, 191102.

(25) Mardirossian, N.; Head-Gordon, M. ωB97M-V: A combinatorially optimized, range-separated hybrid, meta-GGA density functional with VV10 nonlocal correlation. *J. Chem. Phys.* **2016**, *144*, 214110.

(26) Peverati, R.; Truhlar, D. G. Improving the accuracy of hybrid meta-GGA density functionals by range separation. *J. Phys. Chem. Lett.* **2011**, *2*, 2810–2817.

(27) Mardirossian, N.; Head-Gordon, M. ωB97X-V: A 10-parameter, range-separated hybrid, generalized gradient approximation density functional with nonlocal correlation, designed by a survival-of-the-fittest strategy. *Phys. Chem. Chem. Phys.* **2014**, *16*, 9904–9924.

(28) Peverati, R.; Truhlar, D. G. M11-L: a local density functional that provides improved accuracy for electronic structure calculations in chemistry and physics. *J. Phys. Chem. Lett.* **2012**, *3*, 117–124.

(29) Tao, J.; Perdew, J. P.; Staroverov, V. N.; Scuseria, G. E. Climbing the density functional ladder: nonempirical meta–generalized gradient approximation designed for molecules and solids. *Phys. Rev. Lett.* **2003**, *91*, 146401.

(30) Peverati, R.; Truhlar, D. G. An improved and broadly accurate local approximation to the exchange–correlation density functional: The MN12-L functional for electronic structure calculations in chemistry and physics. *Phys. Chem. Chem. Phys.* **2012**, *14*, 13171–13174.

(31) Perdew, J. P.; Ruzsinszky, A.; Csonka, G. I.; Constantin, L. A.; Sun, J. Workhorse semilocal density functional for condensed matter physics and quantum chemistry. *Phys. Rev. Lett.* **2009**, *103*, 026403.

(32) Peverati, R.; Truhlar, D. G. Screened-exchange density functionals with broad accuracy for chemistry and solid-state physics. *Phys. Chem. Chem. Phys.* **2012**, *14*, 16187–16191.

(33) Staroverov, V. N.; Scuseria, G. E.; Tao, J.; Perdew, J. P. Comparative assessment of a new nonempirical density functional: molecules and hydrogen-bonded complexes. *J. Chem. Phys.* **2003**, *119*, 12129–12137.

(34) Peverati, R.; Truhlar, D. G. Exchange–Correlation Functional with Good Accuracy for Both Structural and Energetic Properties while Depending Only on the Density and Its Gradient. *J. Chem. Theory Comput.* **2012**, *8*, 2310–2319.

(35) Dirac, P. A. M. Note on exchange phenomena in the Thomas atom. *Math. Proc. Cambridge Philos. Soc.* **1930**, *26*, 376–385.

(36) Perdew, J. P.; Wang, Y. Accurate and simple analytic representation of the electron-gas correlation energy. *Phys. Rev. B* **1992**, *45*, 13244–13249.

(37) Vosko, S. H.; Wilk, L.; Nusair, M. Accurate spin-dependent electron liquid correlation energies for local spin density calculations: a critical analysis. *Can. J. Phys.* **1980**, *58*, 1200–1211.

(38) Yu, H. S.; He, X.; Li, S. L.; Truhlar, D. G. MN15: A Kohn–Sham global-hybrid exchange–correlation density functional with broad accuracy for multi-reference and single-reference systems and noncovalent interactions. *Chem. Sci.* **2016**, *7*, 5032–5051.

(39) Perdew, J. P.; Chevary, J. A.; Vosko, S. H.; Jackson, K. A.; Pederson, M. R.; Singh, D. J.; Fiolhais, C. Atoms, molecules, solids, and surfaces: applications of the generalized gradient approximation for exchange and correlation. *Phys. Rev. B* **1992**, *46*, 6671–6687.

(40) Yu, H. S.; He, X.; Truhlar, D. G. MN15-L: a new local exchange-correlation functional for Kohn–Sham density functional theory with broad accuracy for atoms, molecules, and solids. *J. Chem. Theory Comput.* **2016**, *12*, 1280–1293.

(41) Adamo, C.; Barone, V. Exchange functionals with improved long-range behavior and adiabatic connection methods without adjustable parameters: the mPW and mPW1PW models. *J. Chem. Phys.* **1998**, *108*, 664–675.

(42) Sun, J.; Ruzsinszky, A.; Perdew, J. P. Strongly constrained and appropriately normed semilocal density functional. *Phys. Rev. Lett.* **2015**, *115*, 036402.

(43) Hui, K.; Chai, J.-D. SCAN-based hybrid and double-hybrid density functionals from models without fitted parameters. *J. Chem. Phys.* **2016**, *144*, 044114.

(44) Sitkiewicz, S. P.; Zaleśny, R.; Ramos-Cordoba, E.; Luis, J.; Matito, E. How reliable are modern density functional approximations to simulate vibrational spectroscopies? *J. Phys. Chem. Lett.* **2022**, *13*, 5963–5968.

(45) Laqua, H.; Kussmann, J.; Ochsenfeld, C. An improved molecular partitioning scheme for numerical quadratures in density functional theory. *J. Chem. Phys.* **2018**, *149*.

(46) Turney, J. M.; Simmonett, A. C.; Parrish, R. M.; Hohenstein, E. G.; Evangelista, F. A.; Fermann, J. T.; Mintz, B. J.; Burns, L. A.; Wilke, J. J.; Abrams, M. L. et al. Psi4: an open-source ab initio electronic structure program. *WIREs, Comput. Mol. Sci.* **2012**, *2*, 556–565.

(47) Parrish, R. M.; Burns, L. A.; Smith, D. G.; Simmonett, A. C.; DePrince III, A. E.; Hohenstein, E. G.; Bozkaya, U.; Sokolov, A. Y.; Di Remigio, R.; Richard, R. M. et al. Psi4 1.1: an open-source electronic structure program emphasizing automation, advanced libraries, and interoperability. *J. Chem. Theory Comput.* **2017**, *13*, 3185–3197.

(48) Smith, D. G.; Burns, L. A.; Sirianni, D. A.; Nascimento, D. R.; Kumar, A.; James, A. M.; Schriber, J. B.; Zhang, T.; Zhang, B.; Abbott, A. S. et al. Psi4NumPy: an interactive quantum chemistry programming environment for reference implementations and rapid development. *J. Chem. Theory Comput.* **2018**, *14*, 3504–3511.

(49) Becke, A. D. A multicenter numerical integration scheme for polyatomic molecules. *J. Chem. Phys.* **1988**, *88*, 2547–2553.

(50) Murray, C. W.; Handy, N. C.; Laming, G. J. Quadrature schemes for integrals of density functional theory. *Mol. Phys.* **1993**, *78*, 997–1014.

(51) Treutler, O.; Ahlrichs, R. Efficient molecular numerical integration schemes. *J. Chem. Phys.* **1995**, *102*, 346–354.

(52) Mura, M. E.; Knowles, P. J. Improved radial grids for quadrature in molecular density-functional calculations. *J. Chem. Phys.* **1996**, *104*, 9848–9858.

(53) Stratmann, R. E.; Scuseria, G. E.; Frisch, M. J. Achieving linear scaling in exchange-correlation density functional quadratures. *Chem. Phys. Lett.* **1996**, *257*, 213–223.

(54) Bartók, A. P.; Yates, J. R. Regularized SCAN functional. *J. Chem. Phys.* **2019**, *150*, 161101.

(55) Furness, J. W.; Kaplan, A. D.; Ning, J.; Perdew, J. P.; Sun, J. Accurate and numerically

efficient r2SCAN meta-generalized gradient approximation. *J. Phys. Chem. Lett.* **2020**, *11*, 8208–8215.

(56) Lehtola, S. Meta-GGA density functional calculations on atoms with spherically symmetric densities in the finite element formalism. *J. Chem. Theory Comput.* **2023**, *19*, 2502–2517.

(57) Lehtola, S.; Marques, M. A. Reproducibility of density functional approximations: how new functionals should be reported. *J. Chem. Phys.* **2023**, *159*, 114116.
